# Parallel subgenome structure and divergent expression evolution of allo-tetraploid common carp and goldfish

**DOI:** 10.1038/s41588-021-00933-9

**Published:** 2021-09-30

**Authors:** Jiong-Tang Li, Qi Wang, Mei-Di Huang Yang, Qing-Song Li, Ming-Shu Cui, Zai-Jie Dong, Hong-Wei Wang, Ju-Hua Yu, Yu-Jie Zhao, Chen-Ru Yang, Ya-Xin Wang, Xiao-Qing Sun, Yan Zhang, Ran Zhao, Zhi-Ying Jia, Xi-Yin Wang

**Affiliations:** 1 Key Laboratory of Aquatic Genomics, Ministry of Agriculture and Rural Affairs, Beijing Key Laboratory of Fishery Biotechnology, Chinese Academy of Fishery Sciences, Beijing, China; 2 College of Fisheries and Life Science, Shanghai Ocean University, Shanghai, China; 3 Chinese Academy of Agricultural Sciences, Beijing, China; 4 Key Laboratory of Freshwater Fisheries and Germplasm Resources Utilization, Freshwater Fisheries Research Center of Chinese Academy of Fishery Sciences, Ministry of Agriculture and Rural Affairs, Wuxi, China; 5 Heilongjiang River Fisheries Research Institute, Chinese Academy of Fishery Sciences, Harbin, China; 6 North China University of Science and Technology, Tangshan, China

**Keywords:** Genomics, Gene expression, Zoology

## Abstract

How two subgenomes in allo-tetraploids adapt to coexistence and coordinate through structure and expression evolution requires extensive studies. In the present study, we report an improved genome assembly of allo-tetraploid common carp, an updated genome annotation of allo-tetraploid goldfish and the chromosome-scale assemblies of a progenitor-like diploid *Puntius tetrazona* and an outgroup diploid *Paracanthobrama guichenoti*. Parallel subgenome structure evolution in the allo-tetraploids was featured with equivalent chromosome components, higher protein identities, similar transposon divergence and contents, homoeologous exchanges, better synteny level, strong sequence compensation and symmetric purifying selection. Furthermore, we observed subgenome expression divergence processes in the allo-tetraploids, including inter-/intrasubgenome *trans-*splicing events, expression dominance, decreased expression levels, dosage compensation, stronger expression correlation, dynamic functionalization and balancing of differential expression. The potential disorders introduced by different progenitors in the allo-tetraploids were hypothesized to be alleviated by increasing structural homogeneity and performing versatile expression processes. Resequencing three common carp strains revealed two major ecotypes and uncovered candidate genes relevant to growth and survival rate.

## Abstract (web summary)

Genomic analysis of allo-tetraploid common carp and goldfish identifies parallel subgenome structure and divergent expression processes.

## Main

Compared with plants^1^, few polyploidization events are observed in animals^2^ except in fish^3^ and frogs^4^. The Cyprininae fish include diploids (2*n*: 50 or 48), tetraploids (2*n*:100), hexaploids (2*n*: 150) and higher polyploids (2*n*: 417–470)^5^. The genomes of Cyprininae fish of different ploidies help to study how the subgenomes coordinate to coexist in the same cell. The divergent structure evolution mechanisms underlying subgenome adaptation in polyploids include decreased sequence identity^6^, relaxed purifying selection^7^, transposon expansion^8^, gene fractionation^7^ and loss of genes and/or conserved genomic elements^6^. However, whether the polyploid subgenomes underwent similar structure evolution was studied less.

Both common carp (*Cyprinus carpio*) and goldfish (*Carassius auratus*) in the Cyprininae superfamily, Cyprinidae family, Cyprinoidei suborder are allo-tetraploid (2*n*: 100). The tetraploidization event was hypothesized to result from an interspecific hybridization of two diploids (2*n*: 50)^9^, with one progenitor hypothesized to originate from a diploid Barbinae fish^10^. Allo-tetraploids are used to investigate the allo-polyploidization and rediploidization processes in vertebrates^11,12^. Although five common carp genome assemblies are available^13–15^, they are either extremely fragmented with a small contig N50 size and low chromosome anchoring ratio^14^ or of low genome coverage^13^. Three goldfish genome assemblies were generated but the annotated gene numbers ranged from 43,144 (ref. ^16^) and 56,251 (ref. ^17^) to 80,065 (ref. ^6^). The genomes of zebrafish (*Danio rerio*, 2*n*: 50, Danionidae family, Cyprinoidei suborder) and grass carp (2*n*: 48, Xenocyprididae family, Cyprinoidei suborder) were used as references to study allo-tetraploid genome evolution^16,17^. However, zebrafish are phylogenetically distant from Cyprinidae (~60 million years ago (Ma)^13^) and the grass carp genome has undergone one chromosome fusion^18^. Neither chromosome-level genome assemblies nor transcriptome resources of a diploid progenitor-like fish or a close outgroup fish are available for study. If reference-quality genomes of common carp, goldfish and close diploids were available, we could extensively examine the adaptive and coordinative mechanisms of the tetraploid subgenomes. Besides, a high-quality genome assembly would help to study the breeding of the common carp, which has a variety of domesticated strains^19,20^ with elite phenotypic improvements.

In the present study, we describe the genomes of *P. tetrazona* (2*n*: 50, Barbinae subfamily, Cyprinidae family, Cyprinoidei suborder) and *P. guichenoti* (2*n*: 50, Sarcocheilichthyinae subfamily, Gobionidae family, Cyprinoidei suborder), a high-quality common carp genome and an improved annotation of the goldfish genome. We found evidence for parallel subgenome structure evolution and versatile expression divergence processes in these tetraploids. Resequencing 93 individuals uncovered the geographical genome architecture and domestication of common carp.

## Results

### High-quality genome assemblies and annotations

**Fig. 1:**
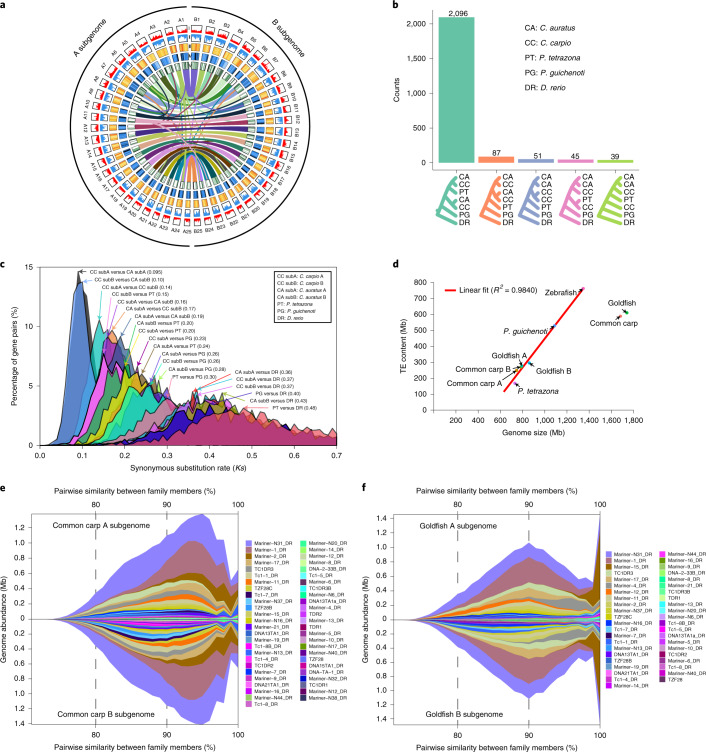
The components and phylogenetic evolution of the common carp genome. **a**, Multidimensional display of genomic components of common carp A and B subgenomes. The density was calculated per 1 Mb. From the outer ring: I, gene densities in the plus strand; II, gene densities in the minus strand; III, heatmap of interspersed repeat content; IV, heatmap of simple sequence repeat content; and V, heatmap of LTR content. The lines link the syntenic gene pairs from different genomic loci. **b**, The top five frequent phylogenetic tree topologies in the 3,171 ‘1:1:1:2:2’ gene families. **c**, The *Ks* distribution is shown among all combination comparisons. The peaks of the *Ks* distribution for each comparison are marked with arrows and text. **d**, Genome size expansion notably correlates with TE bursts. The red line shows the linear relationship between genome size and TE content. **e**, An expansion of TcMar-Tc1 transposons ongoing with a major peak at an average of 96% similarity between family members in the common carp A (upper panel) and B subgenome (bottom panel). **f**, A major peak at an average of 90% during the expansion of TcMar-Tc1 transposons in the goldfish A (upper panel) and B subgenome (bottom panel).

With 185.7-fold sequencing coverage (Supplementary Table 1), we generated an error-corrected new assembly of common carp var. ‘Songpu’ (SP strain; Supplementary Figs. 1a and 2) capturing 1.68 Gb of sequences with a contig N50 size of 1.55 Mb (Table 1); 91.1% of all bases spanning 1.53 Gb were ordered and oriented into 50 pseudo-chromosomes (Table 1 and Fig. 1a). With 188-fold sequencing coverage, the assembled genome size of *P. guichenoti* (Supplementary Fig. 1b) was 1.09 Gb with a contig N50 size of 1.97 Mb, covering 96.9% of the estimated size (Table 1 and Supplementary Fig. 3a). In total, 88.6% of all bases were anchored to 25 pseudo-chromosomes with an average size of 38.56 Mb (Supplementary Fig. 4). With 185-fold sequencing coverage, the assembled genome size of *P. tetrazona* (four-banded strain; Supplementary Fig. 1c) was 730 Mb, accounting for 97.9% of the estimated size (Supplementary Fig. 3b). The contig N50 size was 1.42 Mb and almost 85.9% of all bases were anchored into 25 pseudo-chromosomes with an average length of 25.08 Mb (Supplementary Fig. 5).

**Table 1:** Summary of genome assemblies of three species

		*C. carpio*	*P. guichenoti*	*P. tetrazona*
Genome assembly	Estimated genome size (Mb)	1,830 (ref. ^14^)	1,125	745
	Assembly size (Mb)	1,681	1,088	730
	Contig N50 size (kb)	1,554	1,966	1,423
	Longest contig (Mb)	13	14.2	9.9
	Anchored sequences (Mb)	1,531	964	627
	Average chromosome size (Mb)	30.62	38.56	25.08
	GC content (%)	37.11	39.13	38.24
Repeats	Retrotransposons (Mb)	117.8	111.9	41.9
	DNA transposons (Mb)	193.6	213.9	46.2
	Others (Mb)	362.5	266.7	122.8
	Total (Mb)	673.9	592.5	210.9
Protein-coding genes		47,924	24,284	21,943

The genome coverage and contiguity of each assembly were evaluated. The Illumina genome-seq reads to the genomes of common carp, *P. guichenoti* and *P. tetrazona* had alignment ratios of 99.4%, 99.6% and 98.9%, respectively. The average alignment ratios of RNA-sequencing (RNA-seq) reads were >92% to each assembly (Supplementary Table 2). The indicators suggest high genome coverage in these species. The insert size distributions of paired-end/mate-pair libraries were consistent with the estimated sizes (Supplementary Fig. 6). The high-throughput chromosome conformation capture (Hi-C) data clustered on the diagonals showed strong contact signals (Supplementary Figs. 7–9). For common carp, the physical distances of the genetic markers had Pearson’s correlation with the genetic distances, ranging from 0.931 to 0.994 (Supplementary Fig. 10). All data illustrate good contiguity of each assembly. Although the current common carp assembly showed high synteny with the previous assemblies (Supplementary Fig. 11), it had substantial improvements (Supplementary Result 1), reflected by higher genome completeness (Supplementary Table 3), higher coverage (Supplementary Fig. 12), more aligned RNA-seq reads (Supplementary Table 4) and more anchored sequences.

The common carp, *P. guichenoti* and *P. tetrazona* genomes contained 47,924, 24,284 and 21,943 protein-coding genes, respectively. Among them, at least 90.1% and 99.3% of genes were anchored to chromosomes and given functional annotations, respectively (Supplementary Table 5). Evaluating the gene completeness of each species with BUSCO^21^ found a missing rate of ~2.0–4.7% (Supplementary Table 6), suggesting sufficient quality of the gene models.

We predicted 48,857 protein-coding genes in the most contiguous goldfish genome^17^ (Supplementary Table 7 and Supplementary Fig. 13). Compared with the previous gene models^6,16,17^, the complete rate increased from 92.3% to 97.8% and the missing rate decreased from 3.3% to 0.8% in the new set (Supplementary Table 8). In total, 94.7% and 98.6% of genes were in 50 chromosomes and given functional annotations, respectively (Supplementary Table 5).

### Parallel subgenome sequence and structure evolution

Based on the phylogenetic analysis of individual genes, diploid Barbinae species were closely related to the progenitor of one tetraploid subgenome and *P. guichenoti* was an outgroup of these allo-tetraploids^10,22^. The assemblies and transcriptome of *P. guichenoti* and *P. tetrazona* would facilitate studying the allo-tetraploid structure evolution and expression divergence processes.

#### Division of the tetraploid subgenomes

The proteins from zebrafish, *P. guichenoti*, *P. tetrazona*, common carp and goldfish were clustered into 24,070 protein families. There were 3,171 heptad families, each having a single gene in each diploid, 2 common carp genes and 2 goldfish genes (1:1:1:2:2). These 3,171 families comprised 207 phylogenetic topologies, the most frequent of which (2,096 families, Fig. 1b) grouped 1 *P. tetrazona* gene, 1 common carp gene and 1 goldfish gene (considered as the ‘subB’ genes). Among the 2,096 families retained in the following analysis (red distribution in Supplementary Fig. 14), at least 93.3% of all branches had bootstrap values >50, suggesting the reliabilities. A species tree generated from 2,096 gene trees confirmed the previous phylogenetic relationship among these fish^10,22^. In the remaining 1,075 families, we are unable to differentiate the subA and subB genes because of the complex topologies (Fig. 1b).

On enrichment of ‘subB’ genes on one subgenome, we divided 50 chromosomes of each allo-tetraploid into 25 homoeologous chromosome pairs (Supplementary Tables 9 and 10). In each tetraploid, two subgenomes had almost equivalent sizes (Supplementary Fig. 15). The subgenome divisions were validated by the biased alignment of *P. tetrazona* genome-seq reads toward two B subgenomes (Supplementary Figs. 16a and 17a, and Supplementary Table 11). Supplementary Table 12 shows the relationship between the subgenomes in our study and other studies^16,17,23^. As expected, the numbers of *P. guichenoti* reads mapped to two subgenomes in each allo-tetraploid were, overall, similar (Supplementary Figs. 18–20) because of its outgroup relationship.

**Fig. 2:**
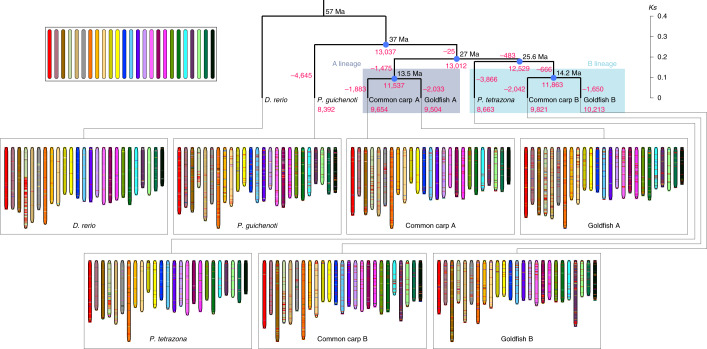
The karyotype evolution in the CDG fish. The figure depicts a model for the distribution of CDG ARs in the studied genomes. Twenty-five ancestral chromosomes reconstructed by using whole-genome multiple alignments are represented by the colored bars (the upper left). Each chromosome of modern fish consists of ARs, indicated with different colors. In general, the modern fish chromosomes and the ancestral chromosomes have similar components. The losses of families including the AGs at different branches were indicated by the negative numbers. Numbers on the nodes or leaves indicate the retained gene families. The AG family retention rate in each subgenome was significantly higher than that in the *P. tetrazone* (χ^2^ test *P* values: common carp A subgenome: 3.45 × 10^−41^; common carp B subgenome: 2.65 × 10^−56^; goldfish A subgenome: 8.11 × 10^−30^; and goldfish B subgenome: 1.19 × 10^−102^).

We defined *P. tetrazona* genome and two B subgenomes as B-lineage genomes and designated two A subgenomes as A-lineage genomes (Fig. 2). The synonymous substitution rate (*Ks*) distributions of the orthologous pairs and the tetraploid homoeologous pairs from 2,096 heptads suggested the speciation time and the tetraploidization time (Fig. 1c). The orthologous pairs between the A- and B-lineage genomes had a mode of *Ks* (0.19) corresponding to a divergence time of 27 Ma. The divergence time between *P. tetrazona* and the ancestor of two B subgenomes was 25.6 Ma (*Ks* mode: 0.18). Two B subgenomes and two A subgenomes (*Ks* modes: 0.1 and 0.095) diverged 14.2 Ma and 13.5 Ma, respectively. We hypothesized that the tetraploidization most probably occurred 13.5–25.6 Ma. Although the diploid progenitor-like fish of the A subgenomes has not been found, we inferred the divergence time of the A progenitor from the B lineage to be 13.5–27.0 Ma. We also used different molecular evolution time to estimate the time of speciation and tetraploidization (Supplementary Fig. 21).

#### Sequence similarity between the subgenomes

The exon size, exon number and protein length per gene were equivalent among the homoeologs and orthologs (Supplementary Fig. 22). It is reasonable that the messenger RNA identities of orthologs between the *P. tetrazona* and the tetraploid subB (Supplementary Fig. 23a) were notably higher than the tetraploid homoeologous pairs, because the A-lineage subgenomes were the outgroup of the B-lineage subgenomes. Remarkably, the protein identities of 2,096 common carp homoeologous pairs (median value: 91.3%; *P* values in Supplementary Fig. 23b) were significantly higher than the orthologous pairs between the common carp subB and *P. tetrazona* (90.6%), and were similarly observed in goldfish.

The repeat components and proportions in the subgenomes displayed high consistency (Supplementary Tables 13 and 14). First, the repeat proportion of each subgenome was approximately equivalent (38.5–39.4%), but higher than *P. tetrazona* (28.86%) and lower than *P. guichenoti* (54.46%; Supplementary Table 15). The contents of all repeats, DNA transposons and retrotransposons correlated with the genome sizes of the studied fish (*r*^2^: 0.984, 0.90 and 0.9699; Fig. 1d and Supplementary Fig. 24), suggesting repeats as driving forces to the diploid Cyprinoidei genome expansion. Second, few transposons except four types (Supplementary Fig. 25) were exclusively distributed on one subgenome, indicating no major transposon expansion in two progenitors after their speciation and before hybridization. Third, similar transposon divergence distributions (19.5%) were observed among four subgenomes (Supplementary Fig. 26a), equivalent to the *P. tetrazona* genome but higher than *P. guichenoti* (18.8%) and zebrafish (13.7%), indicating more ancient transposon expansions in the former five genomes than the latter two genomes. Taking all repeats into consideration, we also observed similar divergence distributions between two subgenomes of each tetraploid (Supplementary Fig. 26b). The repeat expansions in the tetraploids occurred before 27 Ma and after the speciation between Cyprinidae and Gobionidae (37 Ma). The TcMar-Tc1 and LINE/L2 superfamily, the major type of DNA transposons and retrotransposons, also had similar divergence distributions between two subgenomes in each tetraploid (Fig. 1e,f, Supplementary Result 2 and Supplementary Figs. 27–40).

#### Homoeologous exchanges and synteny between subgenomes

We identified the homoeologous exchanges (HEs; Supplementary Fig. 41) by tracing their distributions in two subgenomes. Except the homoeologs in the scaffolds, most common carp subA genes (97%) were distributed in the A subgenome and most subB genes in the B subgenome (97.2%; Supplementary Table 9). However, 3% of common carp subB genes were exchanged to the A subgenome (Supplementary Fig. 42a), as were their counterparts to the B subgenome (Supplementary Fig. 42b). The occurrence of HEs was also observed in the goldfish subgenomes (Supplementary Fig. 42c,d). As *P. tetrazona* was phylogenetically close to the B subgenomes, more *P. tetrazona* genomic-seq reads were expected to be aligned to the exchanged subB genes than the hosted subA genes in the A subgenomes with fewer *P. tetrazona* genomic-seq reads to the exchanged subA genes than the hosted subB genes in the B subgenomes. Although a few low-confidence phylogenetic branches might lead to artificial HE events, the above expectations were observed in both tetraploids, validating the HE events (Supplementary Figs. 16b,c and 17b,c). Besides the HE events identified in the 2,096 families, we detected more HE events in each tetraploid by comparing the *P. tetrazona* read numbers (Supplementary Fig. 43).

The (sub)genome syntenies were represented with ancestral regions (ARs) and protein collinear blocks. In total 275,928 ARs were identified. The ancestral chromosome components of Cyprinidae, Danionidae and Gobionidae (CDG) were reconstructed at 1-kb resolution. The seven (sub)genomes have preserved the ancestral CDG genomic structure without major interchromosomal rearrangements for more than 57 Ma, except many translocation events on chromosome 4 (chr4) occurring in the genomes (Fig. 2 and Supplementary Result 3). Although the A subgenomes were phylogenetically distant from the B subgenomes, the fractions of translocated ARs in the A subgenomes were not significantly different from the B subgenomes (χ^2^ test *P* values: 0.83 and 0.95 in common carp and goldfish, respectively; Supplementary Table 16), but significantly lower than the *P. tetrazona* genome (χ^2^ test *P* values: 0.002 and 0.0009).

The common carp subgenomes shared 15,883 collinear gene pairs (Supplementary Fig. 44a and Supplementary Table 17). The goldfish subgenomes shared 14,401 collinear gene pairs (Supplementary Fig. 45a). The *P. tetrazona* genome had better collinearity with the subgenomes than the *P. guichenoti* genome (Supplementary Figs. 44b,c and 45b,c). Unexpectedly, the B subgenomes shared better preservation of gene collinearity and order consistency with the A subgenomes than with the *P. tetrazona* genome (χ^2^ test *P* values: 1.04 × 10^−25^ and 4.0 × 10^−75^, respectively; Supplementary Table 17). The translocation events on chr4 occurring in the different (sub)genomes possibly resulted in different conservation levels in chr4 between two tetraploids and between two lineages (Supplementary Table 18 and Supplementary Fig. 46). Only 6.9–26.4% of exchanged homoeologs were not covered by the collinear blocks (Supplementary Table 19), suggesting that most HE events were driven by the rearrangements. Both the comparisons of the ARs and of the protein collinear blocks support better subgenome synteny after the tetraploidization event. Using *P. tetrazona* as a proxy, the common carp and the goldfish homoeolog retention rates were 70.8% and 65.2%, respectively.

#### Subgenome-specific genomic retention and loss patterns

We analyzed the retention and loss patterns of duplicated regions at two scales: ARs and ancestral genes (AGs). The common carp AR retention rate and loss rate of the A subgenome (271.3 AR retention and 99.1 loss per Mb) were not significantly different from the B subgenome (269 retentions and 82.5 loss per Mb, χ^2^ *P* value: 0.352). The AR retention rate and loss rates were also not significantly different between two goldfish subgenomes (260.4 AR retention and 87.6 loss per Mb in the A subgenome, and 261.1 AR retention and 59.7 loss per Mb in the B subgenome; χ^2^ *P* value: 0.051).

Of the ARs, 17.7% were lost in *P. tetrazona*, lower than in the common carp B subgenome (23.5%, χ^2^ *P* = 0) and the goldfish B subgenome (18.6%, χ^2^ *P* = 4.68 × 10^−19^; Supplementary Table 20), suggesting increased AR loss in each B subgenome after the speciation between *P. tetrazona* and the diploid progenitor of the B subgenomes (Supplementary Fig. 47). However, the complete loss ratios of ARs in the common carp and goldfish were only 2.5% and 1.5%, respectively. The subgenome-specific AR loss would retain as many as ARs in each tetraploid after the hybridization, that is, sequence compensation (Supplementary Fig. 48) decreased the AR loss.

After separation from the ancestor of the A and B lineages (13,012 AG families), the tetraploid subgenome reserved the AG families at rates from 73.04% to 78.48%, notably higher than that in *P. tetrazona* (66.57%; Fig. 2). The AG retention rates in each subgenome ranged from 59.9% to 63.4%, significantly higher than that in *P. tetrazona* (53.0%; *P* values in Supplementary Table 21). These data suggested slow gene loss in the tetraploids or fast gene loss in *P. tetrazona*. The retained AGs in two A subgenomes shared 95 gene ontology (GO) biological processes and the retained AGs in two B subgenomes had 118 processes (Supplementary Fig. 49), indicating that the subgenomes from the same lineage might retain the functions of each progenitor. The retained AGs in two subgenomes had not only subgenome-specific processes but also many common processes (Supplementary Fig. 50).

#### Symmetric purifying selection on the subgenomes

**Fig. 3:**
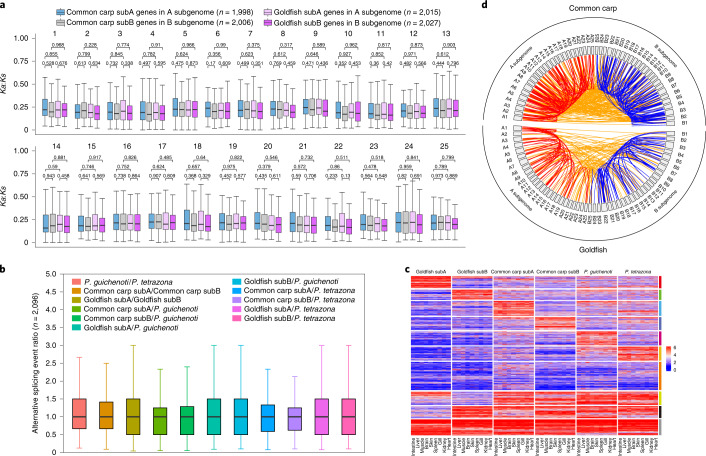
Purifying selection and splicing pattern of the tetraploid homoeologs. **a**, Boxplots of the *Ka*:*Ks* ratio distribution of homoeologs from 2,096 septuplets in each tetraploid (except the genes in the scaffolds or having no *Ka*:*Ks* ratios). The blue, gray, pink and purple boxplots indicate *Ka*:*Ks* distributions from the common-carp-hosted subA genes in the A subgenome, common-carp-hosted subB genes in the B subgenome, goldfish-hosted subA genes in the A subgenome and goldfish-hosted subB genes in the B subgenome, respectively. The *P. guichenoti* orthologs were served as references. The boxplots show the 25th, 50th and 75th percentiles; the upper and lower whiskers correspond to the third quartile + 1.5× the interquartile ratio and the first quartile + ­1.5× the interquartile ratio, respectively. The *P* values were calculated using the two-sided Mann–Whitney *U*-test. The *n* values in the brackets represent the gene numbers of four types on the chromosomes. **b**, The distribution of AS ratios of 2,096 pairs (listed in the brackets) between two compared genomes. The definitions of the boxplots and whiskers were the same as those in **a**. **c**, Heatmap of 2,096 sextuplets, clustered into 10 groups based on the AS number. The color bar at the right indicates the different clusters. **d**, Circos plot distribution of all TS events in two tetraploid genomes. Red lines and blue lines represent intra-A subgenome and intra-B subgenome TS events, respectively. Orange lines join two separate mRNAs from the A and B subgenomes.

In many tetraploids, stronger purifying selection is usually observed in one subgenome^24,25^. Using *P. guichenoti* (Fig. 3a) and *P. tetrazona* (Supplementary Fig. 51) as references, in each tetraploid the *Ka*:*Ks* values (*Ka* is the nonsynonymous substitution rate) of 2,096 homoeologs in the A chromosomes were not significantly different from their counterparts in the B chromosomes (*P* values in Supplementary Table 22), consistent with a study in goldfish^16^ but opposite to a claim of relaxed purifying selection on the common carp A subgenome^13^. The *Ka*:*Ks* ratio distributions in the chromosomes of four subgenomes were not significantly different from their *P. tetrazona* orthologous chromosomes (*P* values in Supplementary Table 22). Together with the deeper insight into the *Ka*:*Ks* ratios of the hosted and exchanged genes in the subgenomes (Supplementary Result 4 and Supplementary Tables 23–27), all data support two subgenomes in each tetraploid under the symmetric purifying selection.

Our sequence and structure analysis is evidence of subgenome parallel structure evolution, including higher protein identities, similar repeat component and divergence, homoeologous exchanges, better syntenies, strong sequence compensation and symmetric purifying selection.

### Expressional divergence of the allo-tetraploid subgenomes

The recent tetraploidization event in the common carp and goldfish enabled analysis of how the homoeologs underwent rediploidization via expression divergence and how two subgenomes coordinated during adaptation to the aquaculture environment. We compared the expression levels across nine tissues shared in these fish and investigated the expression levels across nine conditions in each tetraploid.

#### Alternative splicing balance

Alternative splicing (AS) events generate different isoforms in one gene and increase the transcriptome complexity^26^. The homoeologs and orthologs in 2,096 sextuplets had unbiased AS events either in all tissues (Fig. 3b) or in each tissue (Supplementary Fig. 52). The 2,096 sextuplets were clustered into ten groups according to their AS numbers (Fig. 3c), including six species/subgenome-specific high-frequency groups (56.1%, possibly originating after the speciation and the tetraploidization event). In another two groups the sextuplets had either high-frequency AS events (AS number per gene ≥5, 12.21%) or low-frequency events (AS number per gene ≤3, 15.37%), hinting at conserved AS events in all six genomes.

#### Inter-/intrasubgenome *trans-*splicing preference

The *trans-*splicing (TS) event of low-frequency^27^ fuses segments from two transcripts of different genes to generate a new transcript^28^. Few of the 2,096 sextuplets or of all genes participated in TS (0.52–3.96%; Supplementary Fig. 53 and Supplementary Table 28), supporting the rarity of TS events. The TS events show tissue bias (Supplementary Figs. 54–58) and species bias (Supplementary Table 28). With the long transcriptome reads (Supplementary Fig. 59), 79 TS events in the common carp and 111 in the goldfish were validated (Supplementary Table 29), >91% of which were predicted to have coding potentials. More long RNA-seq reads would be beneficial for validating more TS events.

The intersubgenome TS events (225) in the common carp were more than the intrasubgenome events (124 in the A subgenome and 112 in the B subgenome; Fig. 3d). Likewise, in the goldfish more intersubgenome TS events (81) were found than intrasubgenome events (56 in the A subgenome and 66 in the B subgenome). The intra-/intersubgenome TS events also showed tissue preferences. Besides homoeologous exchanges, TS events create another layer of genome crosstalk and increase the tetraploid genome complexity.

#### Expression divergence in tissues

**Fig. 4:**
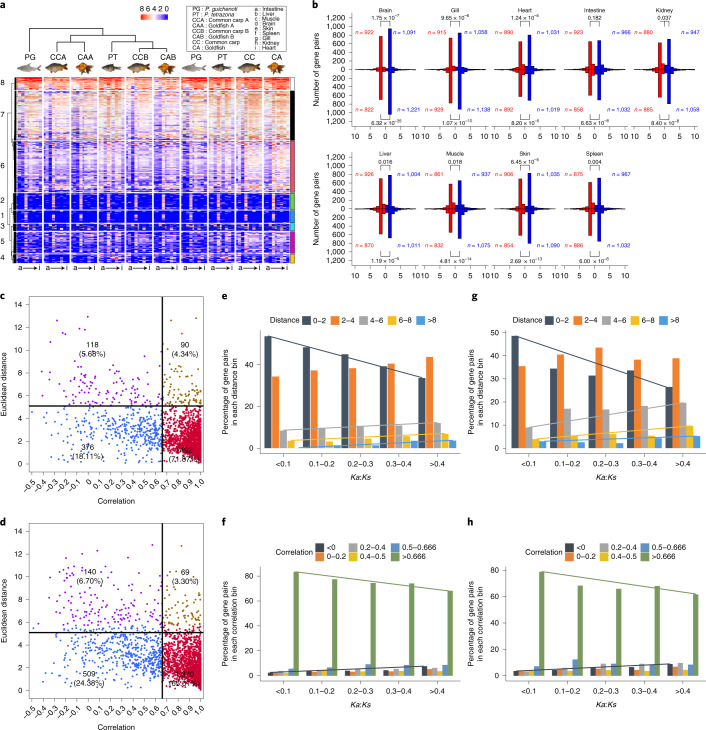
Homoeologous expression divergence across tissues. **a**, Sextuplets, 2,096, clustered into 8 groups based on their expression levels and patterns across 9 tissues, revealing the expression dosage compensation effect in 2 tetraploids. **b**, Expression histograms of 2,096 homoeologous pairs in 9 tissues (upper panels: common carp; bottom panels: goldfish). The *n* values indicate the numbers of the dominant subA genes (red bars) and subB genes (blue bars). The *P* values were computed using the two-sided χ^2^ test. **c**, Dotplot of the expression correlation (*x* axis) and the Euclidean distance (*y* axis) between common carp homoeologs. The upper left box (purple) groups the homoeologous pairs with high Euclidean distance (more than or equal to threshold for the highest 10% of the Euclidean distances) and low correlation (≤0.66). The lower left box (blue) clusters the pairs with low Euclidean distance and low correlation. The upper right box (brown) includes the pairs with high Euclidean distance and high correlation. The pairs in the lower right box (red) have low Euclidean distance and high correlation. Each box lists the number and percentage of homoeologous pairs in one group. **d**, Dotplot of the expression correlation (*x* axis) and the Euclidean distance (*y* axis) between goldfish homoeologs. The definitions of the plots of different colors were consistent with those in **c**. **e**, Euclidean distance distribution in different *Ka*:*Ks* groups in common carp. More conserved complementary DNA sequence is indicated by a shorter distance between two genes. **f**, Expression correlation distribution in different *Ka*:*Ks* groups in common carp. A more conserved cDNA sequence is associated with more closely correlated gene expression. **g**, Euclidean distance distribution in different *Ka*:*Ks* groups in goldfish. **h**, Expression correlation distribution in different *Ka*:*Ks* groups in goldfish.

In all tissues of both tetraploids, the homoeolog expression levels were notably lower than the *P. guichenoti* orthologs (Supplementary Fig. 60 and Supplementary Table 30). In each tissue, the expression levels of 2,096 pseudo-ancestral genes in the common carp and goldfish increased, close to or higher than the diploid orthologs, hinting at the dosage compensation (Fig. 4a and Supplementary Fig. 60). In total, 1,451 (69.2%) pairs and 1,916 (91.4%) of the 2,096 common carp homoeologs were cotranscribed in 9 tissues and in at least 3 tissues, respectively (Supplementary Table 31). The proportions of cotranscribed goldfish homoeologous pairs approximated to those in common carp. These data suggest that the homoeologs were subject to cotranscription to maintain the dosage compensation.

Despite the lowered expression of the tetraploid homoeologs, the coexistence of similar expression and differentiated expression patterns was observed in two tetraploids. In each tetraploid, 4,192 homoeologs in 2,096 pairs were clustered into 8 groups based on their expression patterns (Supplementary Fig. 61a,b). The common carp homoeologs in 1,218 pairs that have similar expression patterns were distributed in the same groups, with the remaining homoeologs in different groups (Supplementary Fig. 61c). Most of the common carp homoeologous pairs with or without similar expression patterns tended to have significantly correlated expression (Pearson’s correlation >0.66, *P* < 0.05; 992 of 1,218 in the same groups and 610 of 878 in different groups; Supplementary Fig. 61d).

The expression dominance toward one subgenome, observed in many polyploids^7^, was confirmed. A large number of 2,096 common carp homoeologous pairs had dominant subB genes in each tissue except the intestine (Fig. 4b). The global expression levels of the subB genes were higher than in their subA copies (Supplementary Fig. 62a). The global expression levels of all genes in the B subgenome were also higher than those in the A subgenome (Supplementary Fig. 62b). Likewise, the expression dominance toward the goldfish B subgenome was observed (Fig. 4b), consistent with previous studies^16,17^.

In total, 929 common carp subA, 982 common carp subB, 981 goldfish subA and 1,030 goldfish subB genes in 2,096 sextuplets showed conserved expression profiles with their *P. guichenoti* orthologs (Supplementary Figs. 63 and 64). More common carp homoeologous pairs had significant expression correlation (1,582 of 2,096; Fig. 4c) than the orthologous pairs between the common carp and *P. tetrazona* (Supplementary Fig. 63c,d; χ^2^ *P* values: 1 × 10^−113^ and 1.3 × 10^−116^). The goldfish homoeologous pairs with significant expression correlation (1,439 of 2,096; Fig. 4d) were more than the orthologous pairs between the goldfish and *P. tetrazona* (Supplementary Fig. 64c,d; χ^2^ *P* values: 6.97 × 10^−76^ and 1.38 × 10^−62^). These data indicated the increased expression correlation between subgenomes after the tetraploidization event.

The nondivergent common carp homoeologous pair numbers, measured by the Euclidean distance^29^ and correlation^30^, were 1,868 and 1,582, respectively. In total, 1,492 common carp pairs were considered nondivergent by both methods (Fig. 4c,d). The percentage of common carp divergent homoeologous pairs increased as *Ka*:*Ks* increased (Fig. 4e, gray, yellow and blue lines; Fig. 4f, black and green lines) whereas the trend was reversed for less divergent gene pairs (Fig. 4e, black line). These two trends also existed in the goldfish homoeologous pairs (Fig. 4g,h). The tetraploid homoeologous pairs were less divergent than the orthologous pairs between the tetraploid subB and *P. tetrazona* (Supplementary Figs. 63c,d and 64c,d). The data suggested the lowered expression divergence of the homoeologs after the tetraploidization events.

The purifying selection plays a role in the homoeolog functionalization. Four functionalization mechanisms were classified as follows: coexpression of two homoeologs, nonfunctionalization (non-F)^31^, subfunctionalization (sub-F)^32^ and neofunctionalization (neo-F)^33^. The 2,096 common carp pairs were classified into 1,205 coexpressed pairs, 572 neo-F pairs, 273 sub-F pairs and 43 non-F pairs (Supplementary Table 32). The last three groups showed significantly lower purifying selection than the coexpressed groups (*P* values in Supplementary Fig. 65a). The significantly differentiated purifying selection was also observed in the goldfish subgenomes (*P* values in Supplementary Fig. 66a).

Our expression analysis confirmed previous reports on expression dominance^13^, high correlation^11^ and purifying selection on functionalization in the tetraploids^11^. Our data uncovered additional divergent expression processes, including expression level decreases, dosage compensation, coexistence of similar and different expression patterns and increased expression correlation.

#### Expression divergence in different conditions

The homoeologs were subject to cotranscription in different conditions (Supplementary Table 7). Among 2,096 common carp homoeologous pairs, 1,459 (69.6%) and 1,916 (91.4%) were cotranscribed in all conditions and at least three conditions, respectively (Supplementary Table 31). The proportions of goldfish that cotranscribed homoeologous pairs in all conditions and at least three conditions were also the majority.

Despite the lack of compared conditions in the diploids, we observed the coexistence of similar and differentiated patterns of homoeologous pairs in these conditions (Supplementary Fig. 67). The notable expression correlations were observed in most homoeologous pairs with similar (72.4% and 74.8% in the common carp and goldfish, respectively) and differentiated patterns (55.4% in common carp and 61.6% in goldfish; Supplementary Fig. 67). Expression dominance toward the B subgenomes was also in evidence (Supplementary Figs. 68 and 69). The nondivergent homoeologous pairs, measured with the Euclidean distance and the correlation, were still in the majority (≥60.58%; Supplementary Figs. 70a and 71a).

The effects of the purifying selection on the expression divergence and the homoeolog functionalization in multiple conditions were proven. The percentage of divergent homoeologous pairs increased as *Ka*:*Ks* increased (Supplementary Figs. 70b,c and 71b,c), whereas the trend was reversed for less divergent homoeologous pairs. The *Ka*:*Ks* values in the non-F, sub-F and neo-F groups (Supplementary Table 32) were significantly higher than those in the coexpressed group (*P* values in Supplementary Figs. 65b and 66b). A comparison of the four types of homoeologous pairs in tissues and conditions showed dynamic functionalization. In total, 1,206 (57.5%) common carp homoeologous pairs and 1,275 goldfish pairs were consistent in two comparisons (Supplementary Fig. 72 and Supplementary Table 32). Among the remaining inconsistent pairs, the conversion from the coexpressed type to the other three types was dominant (671 of 890 common carp pairs and 812 of 821 goldfish pairs; Supplementary Table 33). The homoeologs might employ functional conversion as the first strategy of expression plasticity in response to different conditions.

We identified the common carp differentially expressed genes (DEGs) by comparing both expression profiles in hypoxia-, CyHV-3- and *Aeromonas hydrophila*-treated groups and corresponding controls, and profiles among different skin colorations (Supplementary Fig. 73). In all comparisons the percentages of differentially expressed subA and subB genes in the 2,096 pairs were not significantly different (*P* values in Supplementary Table 34), but the majority (50–79%) of the 2,096 pairs had only one differentially expressed homoeolog (Supplementary Table 35, except the comparison between black and red colorations). These data indicated a balance of differential expression, also observed in the genome-wide distribution of all DEGs between two subgenomes (Supplementary Table 36) and in the goldfish (Supplementary Fig. 74 and Supplementary Tables 34–36). The differential expression balance in response to different conditions could dampen the stimulus impact and possibly serve as the second strategy of expression plasticity.

The DEGs in each subgenome were enriched in specific biological processes (Supplementary Figs. 75–84). In response to hypoxia, except for 87 GO terms enriched by both common carp subgenomes, the DEGs in the A subgenome were enriched in the macromolecule biosynthetic process, protein binding and RNA biosynthetic process (Supplementary Fig. 75b) whereas the DEGs in the B subgenome preferred the regulation of signaling, response to stimulus and regulation of cell communication (Supplementary Fig. 75c). Functional divergences between two subgenomes were also observed in other comparisons of conditions and in goldfish. The subgenome-specific biological processes in different conditions could reduce the perturbation of gene expression on exposure to stress or phenotypes as the third strategy of expression plasticity.

The expression analysis across conditions revealed the third layer of divergence processes, including dynamic functionalization, differential expression balance and subgenome-specific biological processes in response to variable stresses.

### The domestication of common carp

The common carp is widely spread around the world, having numerous geographical populations. Domestication accelerated its phenotypic diversity (Supplementary Result 5). Genome resequencing of 93 individuals from 3 common carp strains, including the SP strain, domesticated FR (Furui) strain and YR (Yellow river) strain, was performed to compare their genomic diversities and uncover the domestication mechanisms.

#### Phylogeny analysis of three common carp strains

**Fig. 5:**
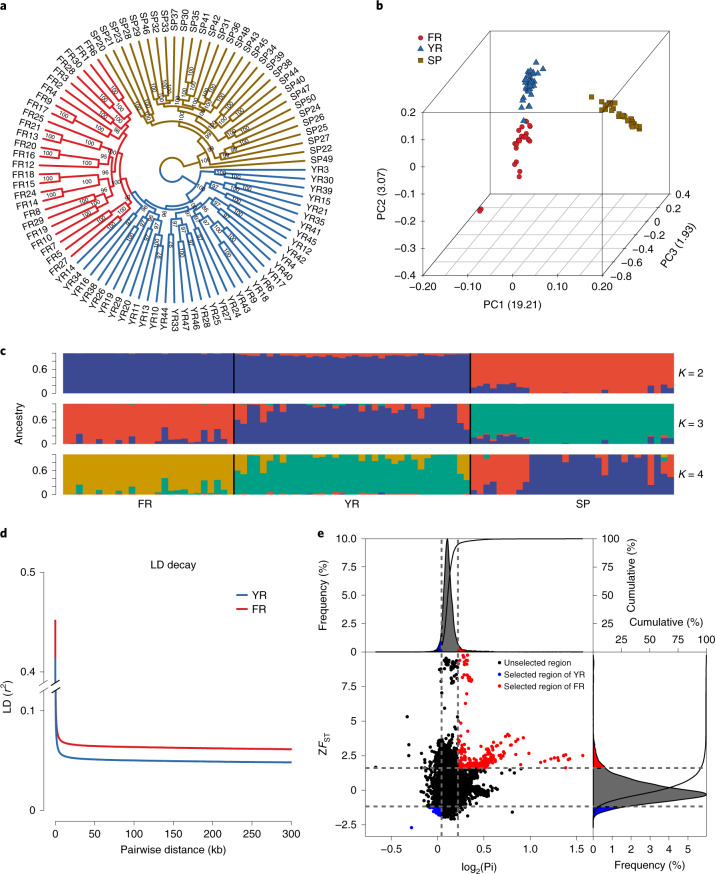
Population analysis of three common carp strains. **a**, Maximum likelihood phylogenetic tree of common carp individuals inferred from whole-genome SNPs. Red, blue and brown colors represent the FR, YR and SP strains. **b**, PCA plots of the first 3 components for 93 samples. **c**, Population structure of the three carp strains estimated with different cluster numbers (*K* = 2–4). Each color represents one ancestral population. Each sample is symbolized by a bar, in which the length of each colored segment shows the proportion of the ancestral population. **d**, LD decay patterns in the FR and YR strains. **e**, Selective sweep regions of the FR and YR strains identified the *Z*-transformed *F*_ST_ (*ZF*_ST_) and π statistics. Red points in the upper right panel are the artificially selected regions in the FR strain. Blue points in the lower left panel are the naturally selected regions in the YR strain.

Maximum likelihood phylogenetic analysis with the core set of 57,049,657 SNPs (Supplementary Result 6) reveals two major clades: the European clade (the SP strain) and the Asian clade (the YR and FR strains; Fig. 5a). In the Asian clade, the domesticated FR strain formed an independent subspecies to the YR strain. Principal component analysis (PCA) based on the core SNPs confirmed the clade classification. The first three principal components (PCs) explained 19.21%, 3.07% and 1.93% of all genetic variances, respectively (Fig. 5b). PC1 separated the Asian strains from the European strain. PC2 and PC3 evidently separated the YR individuals from the FR samples. These findings were supported by the population admixture analysis (Fig. 5c). Some SP strains had genetic components from the YR and FR strains, and vice versa (*K*: 2 and 3), suggesting that these two clades experienced genetic exchange.

#### Low genetic diversity and selection signatures in the domesticated FR strain

Characterization of the linkage disequilibrium (LD, expressed as *r*^2^) pattern is crucial to forward genetics studies. LD decayed to its half-maximum within 128.8 kb in the YR strain, but smaller than that in the FR strain (145.6 kb; Fig. 5d). The FR stain had 21.1% less polymorphism diversity (π = 1.57 × 10^−3^; s.d. = 8.9 × 10^−4^) than the YR stain (π = 1.99 × 10^−3^; s.d. = 1.07 × 10^−3^), as shown with the other four statistics (Supplementary Table 37). Both the lower LD decay rate and the lower diversity in the FR strain indicate a stronger bottleneck^34^ during domestication or a founder effect^35^.

The improved traits by artificial and/or natural selection decreases variations and skews allele frequency, both of which are used to identify potential selected genes (PSGs) in plants^36^ and animals^37,38^. Integrated methods with π (top 5%, π ratio ≥ 1.165) and *ZF*_ST_ (top 5%, *ZF*_ST_ ≥ 1.61) identified 1,057 PSGs in the FR stain compared with the YR stain (Fig. 5e). These PSGs were enriched in the positive regulation of cellular catabolic process, heat shock protein (HSP) binding, regulation of translation and metabolism-related processes (Supplementary Fig. 85a). Many HSPs were reported to function in the survival of cells^39^ and animals^40^. Enrichment of HSP binding might explain the improved survival rate of the FR strain. We also identified 737 YR PSGs enriched in the ion channel-related activity, dioxygenase activity and ubiquitin–protein transferase activity (Supplementary Fig. 85b).

## Discussion

It is essential for allo-tetraploids to alleviate conflicts derived from different progenitor genomes in the same cell. In the common carp and goldfish, the tetraploid subgenomes underwent parallel structural evolution rather than divergent structural evolution. The structural parallelism might be explained by slow evolution between two subgenomes after the tetraploidization or by fast evolution in *P. tetrazona* after its speciation. It is also important for two subgenomes to coordinate their expression during development and in response to environmental changes. We found much more versatile expression divergence strategies in these two tetraploids than the previous studies^4,14,16,17^, which improved expression plasticity and functional flexibility. The strong correlation between two subgenomes might result from either the restricted changes in expression regulations in the tetraploids or the extensive changes in the diploids. As few losses of different-scale genome sequences occurred, expression divergence made greater contributions to adaptation than the structure rediploidization. Comparison of the tetraploidization events in vertebrates (Supplementary Result 7) indicates that common carp and goldfish are valuable for the study of early subgenome structure evolution in polyploid vertebrates.

## Methods

### Genome sequencing and assembly

#### Genome sequencing and read filtration

The welfare and use of animals in this study were done following the recommendations for scientific purposes set up by the Animal Care and Use Committee of the Chinese Academy of Fishery Sciences. We collected a female common carp var. ‘Songpu’ at the hatchery station of Chinese Academy of Fishery Sciences in Beijing, China. Farmed mature *P. guichenoti* and *P. tetrazona* were sampled at Taihu Lake, Wuxi, Jiang Su Province and Beijing, respectively. For common carp, we performed whole-genome sequencing using SMRT sequencing technology and Nanopore technology. Sequencing libraries with 20-kb DNA inserts were sequenced using a Pacific Biosciences Sequel instrument. The Nanopore libraries were sequenced on R9.4 flow cells. We performed whole-genome sequencing for *P. guichenoti* and *P. tetrazona* using Nanopore technology and Illumina platform. All genome-seq reads from Illumina libraries were cleaned using Trimmomatic v.0.35 (ref. ^41^) and SolexaQA v.3.7.1 (ref. ^42^). The sequencing information is described in Supplementary Methods 1.

#### Hybrid assembly

For common carp, de novo contig assembly was developed using raw PacBio reads and Nanopore reads using wtdbg2 (ref. ^43^). The contigs were error corrected with long reads using racon v.1.3.1 (ref. ^44^) and polished with cleaned Illumina reads using pilon v.1.22 (ref. ^45^). The contigs of wtdbg2 assembly and previously published assembly^14^ were assembled into longer contigs using quickmerge^46^. The contigs were scaffolded using the mate-pair libraries with SSPACE v.3.0 (ref. ^47^) and Platanus v.1.2.4 (ref. ^48^). The gaps in the scaffolds were closed with reads from the paired-end libraries using Platanus v.1.2.4 and further filled with long reads using LR_Gapcloser v.1.0 (ref. ^49^). The assembly pipelines for *P. guichenoti* and *P. tetrazona* are described in Supplementary Methods 2.

#### Three-dimensional chromatin conformation capture sequencing

We prepared a Hi-C library for the pseudo-chromosome assembly of each species^50^. Muscle was fixed with fresh formaldehyde, creating DNA–protein bonds. The DNA was digested into fragments using the restriction enzyme *MboI*, and a biotinylated residue was added to the 5′-end of each fragment. During fixation, the fragments adjacent to each other were ligated. After shearing by sonication into smaller fragments, they were pulled down with streptavidin beads. The Hi-C library was sequenced on the Illumina platform with 150-bp PE mode.

#### Integrating Hi-C data and a high-density genetic map

Common carp pseudo-chromosomes were constructed by the integration of Hi-C data and a high-density linkage map^51^ with 14,619 markers. The Hi-C reads were mapped to the polished scaffolds using Bowtie 2 (v.2.3.5.1)^52^ and HiCUP v.0.6.1 (ref. ^53^) was used to filter the Hi-C reads. Based on HiCUP-filtered valid pairs, we clustered scaffolds into pseudo-chromosomes using Lachesis^54^ with a pseudo-chromosome number of 50. We constructed an interaction matrix with cleaned Hi-C reads using HiC-Pro v.2.11.1 (ref. ^55^) (Ligation_site = GATC). The genome was divided into bins of an equal size of 500 kb and the number of contacts between each bin pair was determined. Two linked pins were separated if they had few contacts, as determined by manual checks, resulting in the separation of the pseudo-chromosomes into super-scaffolds, which were anchored to the linkage map. We used BLAT (v.35X1)^56^ (with alignment length coverage of >70%) to align the genetic markers to the super-scaffolds. Only markers with a unique location were used for anchoring and orienting scaffolds, with a string of 100 Ns representing the gap between two adjacent scaffolds. The pseudo-chromosomes of *P. guichenoti* and *P. tetrazona* were generated with valid Hi-C data using Lachesis^54^.

#### Quality evaluation of assemblies

The genome coverage of each assembly was assessed by aligning the cleaned Illumina genome-seq reads with BWA v.0.7.17 (ref. ^57^) and aligning the cleaned Illumina RNA-seq reads to the genome using HISAT 2 (v.2.1.0)^58^. The genome contiguity was measured based on insert size distribution and Hi-C contact signals. We compared the actual insert size distribution with the estimated insert size of each paired-end/mate-pair library, determined by aligning reads to the genome using BWA v.0.7.17. The genome contiguity was confirmed by mapping Hi-C data using HiCPlotter^59^. The common carp genetic markers were aligned to the assembly using BLAT (v.35X1), and the correlation between sequence distance and genetic distance was used to estimate the assembly contiguity. The assessment of common carp assembly improvement was described in Supplementary Methods 3.

### Genome annotation

#### Annotation of repeat elements

A two-step strategy was applied to repeat identification in each assembly. First, species-specific repeat families were de novo identified using RepeatModeler v.1.0.11 (ref. ^60^). RepeatMasker v.4.0.7 (ref. ^61^) was used to search the species-specific families in each genome. Subsequently, each assembly was masked against the Repbase teleost repeat library with RepeatMasker. LTR_finder v.1.07 (ref. ^62^) scanned for full-length long terminal repeat (LTR) retrotransposons and simple sequence repeat markers were detected using MISA^63^. Using the same strategy, we predicted the repeats in the goldfish genome.

#### RNA-seq and read filtration

For each species, nine tissues (brain, gill, heart, intestine, kidney, liver, muscle, skin and spleen) from six individuals were collected. The total RNA from each tissue was extracted and the genomic DNA was removed. For each tissue, three RNA-seq libraries with an insert size of 300 bp were sequenced on the Illumina platform with 150-bp PE mode. We also collected the published RNA-seq data from the same nine tissues of goldfish for comparative analysis (Supplementary Table 7).

#### Prediction and annotation of protein-coding genes

Using the repeat-masked assembly, we generated gene models by integrating predictions from scratch, homolog prediction and RNA-seq models. Fgenesh v.3.1.1 (ref. ^64^) was used to construct de novo gene models. We aligned all fish proteins in the Ensembl database^65^ to the assembly using BLAT (v.35X1)^56^. Proteins with alignments with >70% coverage were realigned to the assembly using GeneWise v.2.4.1 (ref.^66^) to produce accurately spliced alignments. The clean RNA-seq reads from nine tissues trimmed by Trimmomatic v.0.35 (ref. ^41^) and SolexaQA v.3.7.1 (ref. ^42^) were aligned to the assembly using HISAT 2 (v.2.1.0)^58^, followed by StringTie v.1.3.5 (ref. ^67^) to predict RNA-seq-based transcripts. These model sets were combined to produce consensus genes using StringTie v.1.3.5 with the parameter of ‘–merge’. The longest transcript was selected to represent one gene model and inputted into TransDecoder v.5.5.0 (ref. ^68^) to predict the protein sequence. We aligned all proteins against Swiss-Prot, TrEMBL and NR databases with blastp (*e* value: 10^−5^) to identify homologs. Each gene was assigned a KEGG biological pathway and GO terms using KOBAS v.2.0 (ref. ^69^) and Blast2GO v.5.2 (ref. ^70^), respectively. We reannotated the protein-coding genes in the goldfish genome assembly (Supplementary Methods 4). The gene completeness of each fish was assessed with BUSCO v.3.1.0 (ref. ^21^) against the actinopterygii dataset.

### Phylogenetic analysis

#### Identification of ‘subA’ and ‘subB’ genes and subgenomes

Orthologous gene families among common carp, goldfish and three diploid species (*2n*: 50, zebrafish, *P. guichenoti* and *P. tetrazona*) were identified using Orthofinder v.2.3.11 (ref. ^71^) with the protein sequences in five species. In total, 3,171 gene families with a 1:1:1:2:2 relationship (1 zebrafish gene, 1 *P. guichenoti* gene, 1 *P. tetrazona* gene, 2 common carp genes and 2 goldfish genes) were selected to identify the homoeologous pairs derived from the tetraploidization event. We constructed gene trees for these families. In each family, a multiple amino acid alignment was generated using Mafft v.7.453 (ref. ^72^) and converted into a codon alignment using pal2nal v.14 (ref. ^73^). The codon alignment was used to construct a gene tree using IQ-tree v.1.6.12 (ref. ^74^) (maximum likelihood method, 1,000 bootstrap replicates and the best model detected by Modelfinder^75^). In total, 2,096 ‘1:1:1:2:2’ families satisfied the top frequent topology in Fig. 1b. In each such family, a tetraploid gene phylogenetically closer to its *P. tetrazona* ortholog was a subB gene. Otherwise, it was a subA gene.

If a tetraploid chromosome encoded more ‘subB’ genes than ‘subA’ genes, it belonged to the B subgenome. Otherwise, it was derived from the A subgenome (Supplementary Methods 5). We also validated the accuracy of the subgenome division in each tetraploid following the strategy of Chen et al.^17^ (Supplementary Methods 6). In cases when a subA gene was located in a B subgenome chromosome or one subB gene was distributed in the A subgenome, an HE event occurred. We validated these HEs by comparing the aligned *P. tetrazona* read numbers between the exchanged genes and the hosted genes in the same subgenome (Supplementary Methods 6). On the homoeologous regions generated by whole-genome alignments between two subgenomes (Reconstruction of ancestral chromosome components), we compared the *P. tetrazona* read numbers between two homoeologous regions and detected potential HE events (Supplementary Methods 7).

#### Dating speciation

With Phybase package^76^, the 2,096 ‘1:1:1:2:2’ gene trees were used to reconstruct a species tree including zebrafish, *P. guichenoti*, *P. tetrazona* and four tetraploid subgenomes. For each family, we calculated the pairwise nonsynonymous (*Ka*) and synonymous substitution (*Ks*) rates using KaKs_calculator v.2.0 (ref. ^77^) with the YN model^78^. We applied a *Ks* molecular clock of 3.51 × 10^−9^ substitutions per synonymous site per year^12^ to estimate the speciation time of two selected species on the basis of the *Ks* distribution in all pairs between them (Supplementary Methods 8).

### Genome structure analysis

#### The historical activities of L2 and TcMar-Tc1 families

We scanned the subgenome-specific transposon elements (TEs) by adopting the TE distribution analysis^4,23^ (Supplementary Methods 9). LINE/L2 and TcMar-Tc1 are the most abundant retrotransposon and DNA transposon, respectively, in *P. guichenoti*, *P. tetrazona*, common carp and goldfish. To estimate the historical activity of TcMar-Tc1 transposons in these genomes, all copies from each TcMar-Tc1 subfamily were extracted and pairwise alignment between each two copies was performed using blastn. The distribution of pairwise percentage identity, a proxy for divergence time, between members of a family was used to analyze the temporal dynamics of transposon activity. To estimate the historical activity of LINE/L2 elements, the same analysis was performed on each LINE/L2 family.

#### Reconstruction of ancestral chromosome components

To deduce the evolution of the ancestral chromosomes in these fish during speciation and after tetraploidization, we used an optimal approach (Supplementary Methods 10) to detect ancestral CDG chromosome components and their orientation based on the multiple alignments of seven genomes (zebrafish, *P. guichenoti*, *P. tetrazona*, carp A, carp B, goldfish A and goldfish B). Following the strategy of Chen et al.^6^, we performed all-to-all pairwise genomic alignments using Lastz v.1.02.00, axtToChain, chainToAxt and axtToMaf. Using roast, we transformed all pairwise mutation annotation format (MAF) files into multiple alignment mutation annotation format files. In each multiple alignment region (longer than 1 kb), if sequences from at least four genomes belonged to the same homoeologous chromosome, this region was an AR from one ancestral chromosome. The orientation of this AR was considered to be that with more sequence supports than the other orientation. We analyzed the distributions of the ARs in two subgenomes and studied the retention and loss patterns in each subgenome (Supplementary Methods 11). After identifying the AGs in the ARs, we computed the loss and retention of families including AGs in the nodes or leaves of the species tree (Supplementary Methods 12).

#### Detection of rearrangements

The syntenies among diploid genomes and tetraploid subgenomes were studied by scanning ARs and protein collinear blocks. First, using the ARs and their orientations as references, we calculated the fractions involved in the translocations and inversions, for all modern descendants. If one AR was located in a modern chromosome different from the ancestor chromosome, a translocation occurred. We estimated the translocation fraction by dividing the translocated ARs by all ARs. If one AR was located in a modern chromosome originating from the ancestor chromosome, but the orientation of the modern chromosome was opposed to the ancestor chromosome, an inversion occurred. The inversion fraction was estimated by dividing the number of inversed ARs by all ARs. Second, among the genomes of *P. guichenoti*, *P. tetrazona* and four subgenomes, we performed pairwise analyses of protein collinearity using MCScanX^79^ with the parameters of at least five syntenic genes and an *e* value of 1 × 10^−5^.

### Homoeolog feature analysis

#### Comparison of gene structure and identity

In each of 2,096 families, we conducted pairwise comparisons of the gene structures (exon number, exon size and protein length) and sequence identity (mRNA and protein). The mRNA identity and protein identity between two genes were analyzed using blastn and blastp, respectively.

#### Alternative splicing and TS analysis

For each species, the RNA-seq alignments were input to StringTie (v.1.3.5)^67^ to assemble alternative splicing variants using the reference gene models as guides. To study the TS events, RNA-seq reads from each tissue were mapped to the corresponding genome using STAR v.2.7.3 (ref. ^80^) and STAR-fusion v.1.7.0 (ref. ^81^) was used to identify candidate fusion transcripts on the basis of the STAR alignments. Long RNA-seq reads and de novo assembled transcripts were used to validate the TS events (Supplementary Methods 13).

#### Expression divergence analysis in tissue profiling

To study the tissue expression profiles of the tetraploid homoeologs, RNA-seq alignments of each tissue were used to quantify the gene expression level using StringTie v.1.3.5 (ref. ^67^). The level was represented with the value of transcripts per million. The gene expression levels of *P. guichenoti* and *P. tetrazona* in each tissue were also calculated. We calculated the transcripts per million for a tetraploid pseudo-ancestral gene as the sum of the transcripts per million of both homoeologs. To investigate decreased expression levels and dosage compensation, 2,096 sextuplets based on the expression levels of all components in 9 tissues were clustered using the ‘Average method’ and visualized using the R (v.3.5.2) function ‘heatmap’. Furthermore, in each tetraploid we performed the following analysis:

1. Expression clustering: 4,192 homoeologs from 2,096 pairs were clustered using the above method to determine whether 2 homoeologs in 1 pair had similar expression patterns.
2. Dominance analysis: if the expression level of one tetraploid homoeolog was higher than that of its counterpart in one tissue, this pair was defined as a dominant gene pair, and the former was the dominant gene.
3. Conservative analysis: with the strategy of Lien et al.^25^, a tetraploid gene was defined as conserved if Pearson’s correlation between it and its outgroup (*P. guichenoti*) ortholog was >0.66 (*P* ≤ 0.05) across nine shared tissues and diverged if the correlation was <0.66 (*P* > 0.05).
4. Divergence analysis: the Euclidean distance and correlation-based distance of two genes with their expression profiles reflected the uniform divergence and concerted changes, respectively. We used these two indicators to measure the expression divergence of two homoeologs. These two distances were calculated using the R functions of ‘heatmap’ and ‘correl’: the higher the divergence, the larger the Euclidean distance, but the smaller the correlation. If one pair had the highest 10% Euclidean distance or correlation values <0.66, then the expression of this pair was divergent. Five groups of the homoeologous pairs in each tetraploid were divided according to their *Ka*:*Ks* values.
5. Functionalization analysis: based on the clustering information in (1) and conservation information in (3), we identified the subfunctionalized and neofunctionalized homoeologous pairs following the strategy of Lien et al.^25^. The remaining pairs were classified as coexpressed (two homoeologs in the same cluster) and nonfunctionalized (at least one homoeolog was not expressed in all tissues).

#### Expression divergence analysis in condition profiling

To study how two subgenomes coordinate for adaptation to the aquaculture environment after tetraploidization, we collected RNA-seq data related to multiple conditions for common carp and goldfish (Supplementary Table 7). Expression clustering, dominance analysis, divergence analysis and functionalization analysis were carried out as in tissue profiling. We performed differential expression analysis. The DEGs with fold-change (FC) ≥2 and false discovery rate ≤0.05 were identified using DESeq2 (v.1.30.0)^82^ in the comparisons between treatments and controls. We used TBtools v.1.046 (ref. ^83^) to detect GO terms overrepresented in DEGs with an adjusted *P* < 5%.

### Population genome analysis

#### Resequencing and SNP calling

Of the common carp SP strain (*n* = 31), YR strain (*n* = 36) and domesticated FR strain (*n* = 26), 93 individuals were chosen for DNA-resequencing. The three strains were sampled at Beijing, Dong Er (Shandong Province) and Wuxi (Jiang Su Province), respectively. A 150-bp paired-end library was generated for each individual. All libraries were sequenced on the Illumina platform. The raw reads from 93 individuals were filtered using Trimmomatic v.0.35 (ref. ^41^) and mapped to the reference genome using BWA v.0.7.17 (ref. ^57^). Variants were called using HaplotypeCaller and GenotypeGVCFs in the Genome Analysis Tool Kit (GATK, v.3.8)^84^. The variants were classified as SNPs and indels using SelectVariants and filtered using VariantFiltration in GATK. The genomic distribution analysis and functional annotation of variants were performed using ANNOVAR (v.2015Dec14)^85^.

#### Population structure analysis

A phylogenetic tree of 93 individuals was constructed using IQ-tree v.1.6.12 (maximum likelihood method, 1,000 bootstraps and auto-model detected) using the filtered SNPs. PCA was performed using EIGENSOFT v.7.2.1 (ref. ^86^) and the first three eigenvectors were plotted. Population structure was analyzed using Admixture v.1.3.0 (ref. ^87^) with *K* from 2 to 4. For each strain, LD was calculated using PopLDdecay v.3.40 (ref. ^88^). The pairwise *r*^2^ values within and between different chromosomes were calculated.

#### Genetic diversity and selection sweep analysis

We estimated the genetic diversities of the FR and YR strains using five indices (π^89^, θw^90^, Tajima’D^91^, FuLi’D and FuLi’F^92^). For each 100-kb window with a 50-kb step, we calculated the pairwise nucleotide diversity (π) and the average fixation index (*F*_ST_) by using VCFtools v.0.1.16 (ref. ^93^), to identify signals of selection sweep. The *F*_*ST*_ values were converted to *ZF*_ST_ scores following the *Z*-transformation method^37^. The putative candidate regions with the highest 5% π and *ZF*_ST_ were selected as selection signals in the FR strain, whereas the regions with the lowest 5% π and *ZF*_ST_ were defined as natural selection signals in the YR strain. GO enrichment analysis for genes in the selective sweep regions was performed using TBtools v.1.046 (ref. ^83^).

### Reporting Summary

Further information on research design is available in the Nature Research Reporting Summary linked to this article.

## Online content

Any methods, additional references, Nature Research reporting summaries, supplementary information, acknowledgements, peer review information; details of author contributions and competing interests; and statements of data and code availability are available at 10.1038/s41588-021-00933-9.

## Supplementary information

Supplementary Information Supplementary Methods 1–13, Supplementary Results 1–7, Supplementary Figs. 1–89.

Reporting Summary

Supplementary Tables Supplementary Tables 1–37.

Supplementary Data 1 Source data for Fig. 4a–h.

## Data Availability

The genome and transcriptome sequencing data of three species were deposited in the Genome Sequence Archive (GSA)^94^ in the BIG Data Center^95^ (accession nos. CRA002435, CRA002449 and CRA002464) and the Sequence Read Archive (SRA) database (accession nos. PRJNA684670, PRJNA684766 and PRJNA684636), respectively. The genome resequencing data of three common carp strains were available in both the GSA (accession nos. CRA002466, CRA002415 and CRA002463) and the SRA (accession nos. PRJNA684795, PRJNA684797 and PRJNA684676). The assemblies of three genomes were available in both the Genome Warehouse^95^ in the BIG data Center (accession nos. GWHALNJ00000000, GWHACFJ00000000 and GWHACFI00000000) and the Bioproject database (accession nos. PRJNA682709, PRJNA686690 and PRJNA683758). The mRNA sequences, protein sequences and function annotations of four fish are available at figshare (10.6084/m9.figshare.13886912). Genome assembly and RNA-seq data of goldfish were downloaded from the SRA database with accession nos. shown in Supplementary Tables 3 and 7.

## Code availability

The Bioinformatic tools used are all published or publicly available and are described in Methods.
