# Supplementary material for: Parallel subgenome structure and divergent expression evolution of allo-tetraploid common carp and goldfish
Source: Nat Genet. 2021 Sep 30;53(10):1493–503. doi: 10.1038/s41588-021-00933-9 (PMC8492472; doi:10.1038/s41588-021-00933-9)
Supplement: Supplementary file 1 — Supplementary Methods 1–13, Supplementary Results 1–7, Supplementary Figs. 1–89. [file 41588_2021_933_MOESM1_ESM.pdf]

nature portfolio

https://doi.org/10.1038/s41588-021-00933-9

Supplementary information

Parallel subgenome structure and divergent expression evolution of allo-tetraploid common carp and goldfish

In the format provided by the authors and unedited

Supplementary information for

Parallel subgenome structure and divergent expression evolution of allo-tetraploid common carp and goldfish

TABLE OF CONTENTS

Supplementary Methods 7

1. Sequence the genomes of *P. guichenoti* and *P. tetrazona* 7

2. Assemble the genomes of *P. guichenoti* and *P. tetrazona* 7

3. Assess the improvement of the common carp genome assembly 7

4. Re-predict protein-coding genes of goldfish (*Carassius auratus*) 8

5. Name the chromosome number in the genomes of four species 8

6. Validate the subgenome division and the homoeologous exchanges 8

7. Detect the HE events using different depths of reads from *P. tetrazona* aligned to two subgenomes 9

8. Estimate the speciation time and the tetraploidization time 9

9. Identify the subgenome-specific TEs 10

10. Determine the chromosome components of CDG ancestor 10

11. Estimate the AR retention and loss in different genomes 11

12. Compute the loss and retention of families including AGs 11

13. Validating the *trans*-splicing events using Pacbio Iso-seq reads, 454 reads, and TSA sequences 12

14. Definitions of terms 12

Supplementary Results 14

1. Comparing the quality of the new common carp assembly 14

2. Analyzing the repeat contents in seven (sub)genomes 14

3. Studying the syntenies of chr4 in seven (sub)genomes 15

4. The *Ka/Ks* distributions of four types of homoeologues 15

5. Domestication of common carp 15

6. Genomic variations in three common carp strains 16

7. Comparing the tetraploidization events in vertebrates 16

Supplementary Figures 18

Supplementary Fig. 1. The photos of *P. guichenoti*, *P. tetrazona*, and three sequenced common carp strains 18

Supplementary Fig. 2. Sampling sites of the sequenced fish 19

Supplementary Fig. 3. K-mer distribution of reads to estimate the genome sizes of *P. guichenoti* and *P. tetrazona* 20

Supplementary Fig. 4. Genome landscape of *P. guichenoti* 21

Supplementary Fig. 5. Genome landscape of *P. tetrazona* 22

Supplementary Fig. 6. Mate-pair/Paired-end insert distribution of *P. guichenoti*, *P. tetrazona*, and common carp 23
Supplementary Fig. 7. HiC chromosome contact map of common carp 24
Supplementary Fig. 8. HiC chromosome contact map of *P. guichenoti* 25
Supplementary Fig. 9. HiC chromosome contact map of *P. tetrazona* 26
Supplementary Fig. 10. The correlation between the chromosome-level-assembly and the common carp genetic map 27
Supplementary Fig. 11. MUMmer dotplots of previous five assemblies compared to the current assembly 28
Supplementary Fig. 12. Insert size distributions of common carp BAC end sequences in the current assembly and previous assemblies 29
Supplementary Fig. 13. Genome landscape of goldfish 30
Supplementary Fig. 14. The distributions of bootstrap values and branch lengths in 3,171 heptad families 31
Supplementary Fig. 15. Chromosome size comparisons among genomes of the common carp A, common carp B, goldfish A, goldfish B, *P. guichenoti*, and *P. tetrazona* 32
Supplementary Fig. 16. The numbers of *P. tetrazona* reads mapped to the common carp subgenomes and exchanged gene regions 33
Supplementary Fig. 17. The numbers of *P. tetrazona* reads mapped to the goldfish subgenomes and exchanged gene regions 34
Supplementary Fig. 18. The numbers of *P. guichenoti* reads mapped to the common carp subgenomes 35
Supplementary Fig. 19. The numbers of *P. guichenoti* reads mapped to the goldfish subgenomes 36
Supplementary Fig. 20. Ratio distributions of mean read numbers between the homoeologous chromosomes in each tetraploid 37
Supplementary Fig. 21. Estimated speciation time using different molecular evolution rates 38
Supplementary Fig. 22. Exon number, exon size, and protein length comparisons among orthologs and homoeologues in 2,096 pairs 39
Supplementary Fig. 23. mRNA identity and protein identity comparisons among orthologs and homoeologues 40
Supplementary Fig. 24. Expansions of DNA transposons and retrotransposons in Cyprinidae fish 41
Supplementary Fig. 25. Distributions of the subgenome-specific transposons on each tetraploid chromosome 42
Supplementary Fig. 26. Repeat divergence distributions in seven genomes 43
Supplementary Fig. 27. Abundances and pairwise similarities of TcMar-Tc1 DNA transposons in the common carp genome 44
Supplementary Fig. 28. Abundances and pairwise similarities of TcMar-Tc1 DNA transposons in the goldfish genome

....45
Supplementary Fig. 29. Abundances and pairwise similarities of TcMar-Tc1 DNA transposons in the *P. guichenoti* genome 46
Supplementary Fig. 30. Abundances and pairwise similarities of TcMar-Tc1 DNA transposons in the *P. tetrazona* genome 47
Supplementary Fig. 31. Abundances and pairwise similarities of TcMar-Tc1 DNA transposons in the common carp A and B subgenomes 48
Supplementary Fig. 32. Abundances and pairwise similarities of TcMar-Tc1 DNA transposons in the goldfish A and B subgenomes..49
Supplementary Fig. 33. Abundances and pairwise similarities of L2 retrotransposons in the common carp genome...50
Supplementary Fig. 34. Abundances and pairwise similarities of L2 retrotransposons in the goldfish genome 51
Supplementary Fig. 35. Abundances and pairwise similarities of L2 retrotransposons in the *P. guichenoti* genome 52
Supplementary Fig. 36. Abundances and pairwise similarities of L2 retrotransposons in the *P. tetrazona* genome......53
Supplementary Fig. 37. Abundances and pairwise similarities of L2 retrotransposons in the common carp A subgenome 54
Supplementary Fig. 38. Abundances and pairwise similarities of L2 retrotransposons in the common carp B subgenome...55
Supplementary Fig. 39. Abundances and pairwise similarities of L2 retrotransposons in the goldfish A subgenome ...56
Supplementary Fig. 40. Abundances and pairwise similarities of L2 retrotransposons in the goldfish B subgenome...57
Supplementary Fig. 41. Schematic figure for the homoeologous exchange 58
Supplementary Fig. 42. In both common carp and goldfish, homoeologue exchanges in the A and B subgenomes....59
Supplementary Fig. 43. Identification and classification of homoeologous exchanges by comparing *P. tetrazona* read numbers between two homoeologous regions in the tetraploids...60
Supplementary Fig. 44. Gene collinearity among the genomes of common carp A, common carp B, *P. guichenoti*, and *P. tetrazona* 61
Supplementary Fig. 45. Gene collinearity among the genomes of goldfish A, goldfish B, *P. guichenoti*, and *P. tetrazona* 62
Supplementary Fig. 46. Gene collinearity among chr4 in different subgenomes 63
Supplementary Fig. 47. The AR loss ratios in five Cyprinidae (sub)genomes...64
Supplementary Fig. 48. Schematic figure for the sequence compensation between subgenomes 65
Supplementary Fig. 49. The venn diagram of the enriched GO terms by the retained AGs in four subgenomes..........66

Supplementary Fig. 50. The enriched GO terms by the retained AGs in the tetraploid subgenomes 67

Supplementary Fig. 51. *Ka/Ks* distribution of four types of homoeologues of common carp and goldfish using the *P. tetrazona* as a reference 68

Supplementary Fig. 52. Alternative splicing number comparison of 2,096 sextuplet pairs among the common carp subA, common carp subB, goldfish subA, goldfish subB, *P. guichenoti*, and *P. tetrazona* in nine tissues 69

Supplementary Fig. 53. *Trans*-splicing events of the homoeologous genes and orthologous genes from 2,096 pairs across nine tissues 70

Supplementary Fig. 54. *Trans*-splicing maps of *P. guichenoti* 71

Supplementary Fig. 55. *Trans*-splicing maps of *P. tetrazona* 72

Supplementary Fig. 56. *Trans*-splicing maps of common carp 73

Supplementary Fig. 57. *Trans*-splicing maps of goldfish 74

Supplementary Fig. 58. *Trans*-splicing event numbers in nine tissues of all genes from the common carp A, common carp B, goldfish A, goldfish B, *P. guichenoti*, and *P. tetrazona* 75

Supplementary Fig. 59. Sequence identities and coverages of long reads and de novo assembled transcripts used to validate TS events 76

Supplementary Fig. 60. Dosage compensation effect of the tetraploid homoeologues 77

Supplementary Fig. 61. The expression correlation between homoeologues across nine tissues in the common carp and goldfish 78

Supplementary Fig. 62. The expression dominance towards the tetraploid B subgenomes in nine tissues 79

Supplementary Fig. 63. Expression correlations and distances between the common carp genes and their *P. guichenoti* (*P. tetrazona*) orthologs across nine tissues 80

Supplementary Fig. 64. Expression correlation and distance between the goldfish homoeologues and their *P. guichenoti* (*P. tetrazona*) orthologs across nine tissues 81

Supplementary Fig. 65. *Ka/Ks* levels of the co-expressed group, sub-functionalized group, non-functionalized group, and neo-functionalized group across tissues and conditions in common carp 82

Supplementary Fig. 66. *Ka/Ks* levels of the co-expressed group, sub-functionalized group, non-functionalized group, and neo-functionalized group across tissues and conditions in goldfish 83

Supplementary Fig. 67. The expression correlation between the homoeologues across nine conditions in common carp and goldfish 84

Supplementary Fig. 68. Expression dominance towards the subB genes in nine conditions of common carp 85

Supplementary Fig. 69. Expression dominance towards the subB genes in nine conditions of goldfish 86

Supplementary Fig. 70. Expression correlations and distances between the common carp homoeologues across nine conditions 87
Supplementary Fig. 71. The expression correlations and distances between the goldfish homoeologues across nine conditions 88
Supplementary Fig. 72. Statistics of co-expressed, sub-functionalized, neo-functionalized, and non-functionalized groups of 2,096 homoeologous pairs in nine tissues and nine conditions of common carp and goldfish 89
Supplementary Fig. 73. Genome-wide distribution of common carp DEGs in different experiments comparisons 90
Supplementary Fig. 74. Genome-wide distribution of goldfish DEGs in different condition comparisons 91
Supplementary Fig. 75. GO enrichment of common carp DEGs in hypoxia treatment 92
Supplementary Fig. 76. GO enrichment of common carp DEGs in CyHV-3 infection 93
Supplementary Fig. 77. GO enrichment of common carp DEGs in *A. hydrophila* infection 94
Supplementary Fig. 78. GO enrichment of common carp DEGs in comparison of red skin and white skin 95
Supplementary Fig. 79. GO enrichment of common carp DEGs in the other two skin comparisons 96
Supplementary Fig. 80. GO enrichment of goldfish DEGs in bisphenol treatment 97
Supplementary Fig. 81. GO enrichment of goldfish DEGs in gyrodactylus infection 98
Supplementary Fig. 82. GO enrichment of goldfish DEGs in different FCE groups 99
Supplementary Fig. 83. GO enrichment of goldfish DEGs in SNa treatment group 100
Supplementary Fig. 84. GO enrichment of goldfish DEGs between SNa treatment group and SKF treatment group 101
Supplementary Fig. 85. GO enrichment of the PSGs of two common carp strains identified by using π and ZF_{ST} 102
Supplementary Fig. 86. The re-sequencing mapping ratio, depth, and coverage of three common carp trains 103
Supplementary Fig. 87. The venn diagrams of SNPs/Indels identified in three strains 104
Supplementary Fig. 88. SNP patterns in the common carp genome 105
Supplementary Fig. 89. Indels distribution in the common carp genome 106
References 107

Supplementary Methods

1. Sequence the genomes of *P. guichenoti* and *P. tetrazona*

Genomic DNA was extracted from muscle of *P. guichenoti* and *P. tetrazona*, respectively. For each species, a Nanopore genome-seq library and a paired-end Illumina library with an insert size of 250 bp were produced and then sequenced on the corresponding platforms, respectively. The genome-seq reads from Illumina libraries were trimmed to remove the adapters and filter the low-quality bases.

2. Assemble the genomes of *P. guichenoti* and *P. tetrazona*

We estimated the genome size of each species using the k-mer analysis based on filtered reads from the paired-end Illumina library using Jeffyfish[1] with the C-setting. The genome sizes were estimated to be 1.12 Gb and 745 Mb for *P. guichenoti* and *P. tetrazona*, respectively. For each species, two *de novo* contig assemblies were generated using raw Nanopore with wtdbg2[2] and clean Illumina reads with Platanus v1.2.4[3], respectively. The wtdbg2 contigs were error-corrected using the long reads with racon v1.3.1[4] and further polished by cleaned Illumina reads with pilon v1.22[5]. The contigs of wtdbg2 assembly and Platanus assembly were assembled to longer contigs using quickmerge[6]. Using the alignments of HiC reads to the longer contigs using Bowtie 2 (v2.3.5.1)[7], HiCUP v0.6.1[8] removed invalid pairs. Based on the refined alignments, we ordered and oriented contigs into 25 chromosomes using Lachesis v1.0[9] since the chromosome numbers of both *P. guichenoti* and *P. tetrazona* were 25[10,11].

3. Assess the improvement of the common carp genome assembly

We compared the new assembly with previous five common carp assemblies (GCA_000951615.2[12], GCA_001270105.1[13], GCA_004011575.1[14], GCA_004011595.1[14], and GCA_004011555.1[14]) with the following indicators. (1) Contig synteny. The contigs from each previous assembly were aligned to the new assembly using Mashmap 2[15] (mapping segment length = 500 Kb and percentage-identity = 80). (2) Genome completeness was measured with the indicators including genome size without gaps, contig N50, and gap number. (3) Genome coverage was estimated with the proportion of BAC ends[16] in the same sequences to all BAC ends and the RNA-seq alignment ratio. Ends from 34,932 BAC clones were aligned to each assembly using BLAT (v35X1)[17] and the best alignment was selected for each end. Then we estimated the number of BAC clones of which two ends were aligned to the same sequence and calculated the insert size distributions of paired ends. The RNA-seq reads from nine tissues were mapped to each assembly using HISAT 2 (v2.1.0)[18]. (4) Total chromosome size was the sum of bases anchored in 50 chromosomes.

4. Re-predict protein-coding genes of goldfish (*Carassius auratus*)

There were three genome assemblies of goldfish available. Nevertheless the corresponding protein-coding gene number greatly varied, ranging from 43,144 and 56,251 to 80,065. The quality of annotated genes might hinder the comparative genome studies. Therefore, it is necessary to re-annotate the goldfish genome. The most contiguous genome of goldfish (GenBank: GCA_014332655.1)[19] anchoring 95.75% of contigs into 50 pseudo-chromosomes was used as the reference genome. We collected all gene-model transcripts from these three genome assemblies. The PacBio Iso-Seq raw reads of goldfish (Supplementary Table 7) were collected to generated circular consensus sequences using CCS command in the SMRTLink package (6.0.0.47841) with the default parameters. The consensus sequences and gene-model transcripts were aligned to this reference genome using GMAP[20] with –n=1, --max-intronlength-middle=50 kb, --min-trimmed-coverage=90, and --min-identity=90. The clean RNA-seq reads from nine tissues and nine conditions (Supplementary Table 7) trimmed by Trimmomatic v0.35[21] and SolexaQA v3.7.1[22] were aligned to the assembly using HISAT 2 (v2.1.0)[18], followed by StringTie v1.3.5[23] to predict RNA-seq-based transcripts. All the alignments were merged into a consensus gene set using StringTie v1.3.5 with the parameter of '–merge'. Then following the pipelines applied into functional annotation of common carp genes, the proteins of goldfish consensus genes was predicted, aligned against Swiss-Prot, TrEMBL and NR database, and finally assigned with KEGG biological pathways and Gene Ontology terms. We also re-predicted repeats in the goldfish genome using the same two-step strategy applied to the common carp genome.

5. Name the chromosome number in the genomes of four species

To facilitate the following comparative analysis, we identified the orthologous chromosomes based on 2,096 1:1:1:2:2 heptads among zebrafish, *P. guichenoti*, *P. tetrazona*, common carp, and goldfish. If most single-copy genes on a *P. guichenoti* chromosome had orthologs on a zebrafish chromosome, this chromosome was named with zebrafish chromosome number. Each tetraploid genome was partitioned into two subgenomes. The same manipulation was applied to naming the chromosomes of other (sub)genomes.

6. Validate the subgenome division and the homoeologous exchanges

According to Chen *et al.*'s method[19], we utilized the alignment read number from close diploid fish to validate the accuracy of subgenome division. The genome sequencing reads of *P. tetrazona* and *P. guichenoti* were aligned to the common carp and goldfish genomes by BWA[24] with default parameter, respectively. The number of aligned reads was counted in 1 kb non-overlapping windows across the entire subgenome. *P. tetrazona* is

phylogenetically close to two B subgenomes and it is reasonably that the *P. tetrazona* reads aligned to two B subgenomes were more than the reads to two A subgenomes (Supplementary Figs. 16 and 17). Since *P. guichenoti* is the outgroup of four tetraploid subgenomes, the read number distributions of *P. guichenoti* reads against each homoeologous chromosome pair were expectedly equivalent (Supplementary Figs. 18 and 19).

We validated these exchange events by assessing the read numbers of *P. tetrazona* in these exchanged regions. Suppose in each tetraploid the 'hosted-subA-D', 'exchanged-subB-D', 'hosted-subB-D', and 'exchanged-subA-D' represent the distributions of read numbers in 1 kb non-overlapping windows in the hosted subA genes in the A genome, the exchanged subB genes in the A genome, the hosted subB genes in the B genome, and the exchanged subA genes in the B genome, respectively. If the subB genes were exchanged from the B subgenome to the A subgenome, then the 'exchanged-subB-D' is significantly higher than the 'hosted-subA-D' (Mann-Whitney *U* test one-tailed P value < 0.05). If the subA genes were exchanged from the A subgenome to the B subgenome, then the 'exchanged-subA-D' is significantly lower than the 'hosted-subB-D' (Mann-Whitney *U* test one-tailed P value < 0.05).

7. Detect the HE events using different depths of reads from *P. tetrazona* aligned to two subgenomes

Besides using the tree topologies to identify the HE events occurring in the genic regions, we further detected the HE events occurring in the homologous regions between two subgenomes by comparing the *P. tetrazona* read numbers in these regions. The homologous regions were generated by all-to-all pairwise whole genomic alignments between two subgenomes, described in the section 'Reconstruction of ancestral chromosome components' in the main text. The chromosomes in the B subgenome had more aligned *P. tetrazona* reads than their homoeologous chromosomes in the A subgenome, with mean read number ratios ranging from 1.352 to 3.854. The mean ratios of *P. guichenoti* reads mapped to the homoeologous chromosomes between two subgenomes ranged from 0.678 to 1.557 (Supplementary Fig. 20). To decrease the false positive rate of potential HE regions, if the *P. tetrazona* read number in one region in the A subgenome was 1.73 times more than that its homologous region in the B subgenome, we considered a HE event occurring in these two regions. The potential HE regions were further classified into three groups based on their genomic locations: intergenic, exonic, and intronic HE regions.

8. Estimate the speciation time and the tetraploidization time

The speciation time between zebrafish and the ancestor of Cyprinidae and Gobionidae (CG) was on the basis of the mode (0.4) of all *Ks* values between zebrafish and CG fish. The mode (0.26) of all *Ks* values between *P.*

guichenoti and Cyprinidae fish (*P. tetrazona* and four tetraploid subgenomes) was used to estimate the speciation time between *P. guichenoti* and the Cyprinidae ancestor. The speciation time of A lineage genomes from B lineage genomes was based on the mode (0.19) of all *Ks* values between tetraploid subA genes and their *P. tetrazona* orthologs and between tetraploid homoeologues. The mode (0.18) of all *Ks* values between tetraploid subB genes and their *P. tetrazona* orthologs was used to estimate the speciation time between *P. tetrazona* and the B progenitor. The divergence time between two B subgenomes was on the basis of the *Ks* mode (0.1) between the common carp subB genes and corresponding goldfish orthologs. The divergence time between two A subgenomes was on the basis of the *Ks* mode (0.095) between the common carp subA genes and corresponding goldfish orthologs. We applied a *Ks* molecular clock of approximately 3.51×10^{-9} substitutions per synonymous site per year[25] to estimate the above speciation time. Besides the above molecular evolution time, we also using another two divergence times (the divergence time between Danionidae and Cyprinidae of 106 Mya[26], and the speciation time between zebrafish and the last common ancestor of *C. carpio* and *C. auratus* of 32.81~36.84 Mya[19]) as calibration points to estimate each speciation time (Supplementary Fig.21).

9. Identify the subgenome-specific TEs

The TEs specific to each progenitor were used to differentiate two subgenomes in the allotetraploid frog *X. laevis*[27]. According to the same strategy, we calculated two subgenome-biased indexes (SBI) for each of 2,208 types of TE: SBI-A = A/(A + B) − B/(A + B), and SBI-B = B/(A + B) − A/(A + B), where A is the number of the TEs in the A genome and B is the number of the TEs in the B genome. If SBI-A ≥ 0.6 and (A > B) is observed in at least 20 chromosomes of the A subgenome, the TE is regarded to be exclusive on the A subgenome. Likewise, if SBI-B ≥ 0.6 and (B > A) is observed in at least 20 chromosomes of the B subgenome, the TE is regarded to be exclusive on the B subgenome.

10. Determine the chromosome components of CDG ancestor

Since all studied fish from Cyprinidae, Danionidae, and Gobionidae had 25 pairs of chromosomes, we deduced that the ancestor of Cyprinidae, Danionidae, and Gobionidae (CDG ancestor) had 25 pairs of chromosomes. An optimal approach was used to detect ancestral chromosome components and their orientation based on the multiple alignments among seven genomes. In each multiple alignment block, denoted as (A), sequences of seven genomes were derived from N different ancestral chromosomes (N ≤ 7). C_N was the sequence number derived from each ancestral chromosome. An optimal ancestral chromosome N of the block A satisfied the following conditions:

$L(N)$=Maximal (C_1, C_2,……, C_N) and $L(N) \geq 50\% \times$ Sum (C_1, C_2,……, C_N).

All sequences from seven genomes in this block might originate from this ancestral chromosome N. If one block had no optimal chromosome satisfying the above conditions, this block was discarded. If one orientation of an ancestral component having more sequence supports than the other orientation, the former was the ancestral orientation. If two orientations had the same number of sequence supports, this block was defined to have no clear orientation. Only the block having the clear ancestral origin and orientation was retained in the following analysis.

11. Estimate the AR retention and loss in different genomes

In each multiple alignment block, we counted the number of lost ancestral regions (ARs) in each tetraploid subgenome as follows:

Single-copy AR loss in the A subgenome: (ZF/PG/PT/CCA/CCB/CAA/CAB >=4) and A=0 and B=1;

Single-copy AR loss in the B subgenome: (ZF/PG/PT/CCA/CCB/CAA/CAB >=4) and A=1 and B=0;

Double-copies loss: (ZF/PG/PT/CCA/CCB/CAA/CAB >=4) and A=0 and B=0;

Double-copies retention: (ZF/PG/PT/CCA/CCB/CAA/CAB >=4) and A=1 and B=1.

‘ZF/PG/PT/CCA/CCB/CAA/CAB >=4’ meant that this region was covered by at least four genomes. ‘A=0’ and ‘A=1’ meant the AR retention and loss in the A subgenome, respectively. Zebrafish (ZF), *P. guichenoti* (PG), and *P. tetrazona* (PT), *C. carpio* A subgenome (CCA), *C. carpio* B subgenome (CCB), *C. auratus* A subgenome (CAA), and *C. auratus* B subgenome (CAB).

12. Compute the loss and retention of families including AGs

Adopting Chen *et al.*’s strategy[28], gene families containing the AGs from PG, PT, CCA, CCB, CAA, and CAB, were used to count the numbers of gene loss and retention on each branch, where N(.) is the gene family number.

The number of the remained families in the CG ancestor: N(PG or PT or CCA or CCB or CAA or CAB >=1)

The number of the remained families in the C ancestor: N(PT or CCA or CCB or CAA or CAB >=1)

The number of the remained families in the ancestor of A lineage: N(CCA or CAA >= 1)

The number of the remained families in the ancestor of B lineage: N(PT or CCB or CAB >= 1)

The number of the remained families in the common ancestor of two B subgenomes from common carp and goldfish: N(CCB or CAB >= 1)

The number of the remained families in each species: N(X >=1) (X: each species)

Loss at each node was derived from the remained family number at the direct parent node minus the remained number at the current node.

13. Validating the *trans*-splicing events using Pacbio Iso-seq reads, 454 reads, and TSA sequences

To back up the existence of *trans*-splicing events in common carp and goldfish, we collected Pacbio Iso-Seq reads and 454 RNA-seq reads from SRA database and *de novo* assembled transcripts from TSA database (Supplementary Table 7). The long reads and transcripts had high sequence identities and coverages to the corresponding genomes by aligning them with using GMAP[20] with –n=1, --max-intronlength-middle=50 kb, --min-trimmed-coverage=90, and --min-identity=90. The RNA-seq reads supporting the TS events were aligned to all long reads and transcripts with BWA v0.7.17[24]. Then the aligned long reads and transcripts were inputted into TransDecoder v5.5.0[29] to predict coding potentials.

14. Definitions of terms

Higher protein identities. In each tetraploid, the protein identities of 2,096 homoeologous pairs were higher than the identities of 2,096 pairs between the tetraploid subB homoeologues and their *P. tetrazona* orthologs (Supplementary Fig. 23b). Since the subA genes were the outgroups of the subB homoeologues and their *P. tetrazona* orthologs, we considered the protein identities of homoeologues to be relatively raised compared with the *P. tetrazona* orthologs.

Protein collinear blocks. Chromosomal regions with conserved sequence and order of protein shared by two and more of genomes, identified with MCScanX[30]. Compared to the ARs, mainly consisting of small conserved DNA segments, protein collinear blocks are larger in scale. Moreover, the definition emphasized the sequence conservation, orders, and orientations of the adjacent protein-coding genes.

Better syntenies. The synteny level between two genomes was measured with the consistent collinear gene number. Each tetraploid B subgenome is supposed to have better syntenies with *P. tetrazona* genome than the other (sub)genomes because the B subgenomes and *P. tetrazona* are phylogenetically close. Unexpectedly, in each tetraploid it was observed that the chromosomes in the B subgenome had higher synteny levels with their homoeologous counterparts in the A subgenomes than the orthologous *P. tetrazona* chromosomes (Supplementary Table 17).

Sequence compensation. The loss of one homologous genomic region in a diploid always occurred after the speciation. In tetraploids one AR had two corresponding homologous regions. Even if one homologous region

was preferentially lost in one subgenome, the other subgenome might preserve the homologous region, that is, a sequence compensation mechanism to maintain the ancestral functions (Supplementary Fig. 48). By the sequence compensation mechanism, only 2.5% and 1.5% of ARs were lost in the genomes of common carp and goldfish, respectively.

Ancestral gene (AG). Genes located in one AR shared by multiple genomes were considered to originate from one same ancestral gene.

Stronger expression correlation. The subB genes are hypothesized to have higher expression correlation with their *P. tetrazona* orthologues than their subA homoeologues because the A lineage genomes diverged from the B lineage genomes. In contrast, in each tetraploid it was observed that the subB genes have stronger expression correlation with their subA homoeologues than their *P. tetrazona* orthologues.

Dynamic functionalization. We classified 2,096 homoeologous pairs into four types on the basis of the expression clustering and conservation in nine tissue profiles: coexpression of two homoeologues, non-functionalization (non-F, loss of ancestral gene functions in one homoeologue)[31], sub-functionalization (sub-F, retaining subsets of ancestral gene functions in different homoeologues)[32], and neo-functionalization (neo-F, assigning a novel function to one homoeologue)[33]. Likewise, the 2,096 pairs were clustered into the same four types in condition profiles. If one pair was classified into two different functionalization types in tissue profiles and condition profiles, we considered this pair to be dynamically functionalized.

Supplementary Results

1. Comparing the quality of the new common carp assembly

The new assembly of common carp makes substantial improvements compared with the previous assemblies. First, it has higher genome completeness. The genome sizes without gaps in the previous assemblies are smaller than that in the current assembly. The contig N50 size increases from at most 94 kb in the early assemblies to 1,554 kb in the updated assembly, a 16.5~222-fold improvement (Supplementary Table 3). The gap number is significantly reduced from 479,190 to 13,694, a 4.23~35-fold decrease. Second, the updated assembly has higher genome coverage. With a standard normal distribution corresponding to the real BAC insertion size, the alignment proportion of both ends from 34,932 BAC clones[16] to the same sequences in the updated assembly is higher than in the previously assemblies (Supplementary Fig. 12). The alignment ratio of RNA-seq reads from nine tissues is improved from 85.32% to 92.79% in the updated assembly (Supplementary Table 4). Third, the current assembly integrates the most bases into the chromosomes. It anchors 1.53 Gb of sequence onto 50 chromosomes, a substantial improvement over the previously assemblies (0.87~1.3 Gb anchored). These results indicate that the updated common carp genome may supplant earlier assemblies as chromosome-scale references.

2. Analyzing the repeat contents in seven (sub)genomes

DNA transposon and retrotransposon contents correlated with the Cyprinoidei genome sizes (r^2 = 0.90 and 0.9699, Supplementary Fig. 24). Since DNA transposons account for higher proportions of genome content than retrotransposons, the former likely made larger contributions to genome expansion.

In the allo-tetraploid frog[27], the bursts of transposon activities were estimated at different time in two progenitors and distinct transposon families were observed to be specific to one subgenome. We adopted the TE distribution analysis[19,27,34] to identify the subgenome-specific ones out of 2,208 types of TEs. In the common carp and goldfish, only Mariner-12_DR and Mariner-N17_DR were almost exclusively located in the A subgenome (Supplementary Figs. 26a, b, e, and f) while TC1DR2 and Tc1-8_DR were enriched in the B subgenome (Supplementary Figs. 26c, d, g, and h).

We further examined the divergences of the TcMar-Tc1 superfamily and LINE/L2 superfamily are the major type of DNA transposon and retrotransposon in the tetraploid subgenomes, *P. guichenoti*, and *P. tetrazona* genomes (Supplementary Tables 13, 14, and 15). Although the major divergence peaks among members in the TcMar-Tc1 superfamily varied in these four fish (Supplementary Figs. 27-30), the major divergence peaks for TcMar-Tc1 in

two subgenomes of each tetraploid were equal with similar distributions (Figs. 1e-f). Furthermore, the proportions of the major TcMar-Tc1 subfamilies in two subgenomes of each tetraploid were also consistent (Supplementary Figs. 31a, 31b, 32a, and 32b). Likewise, although the species-specific divergence peaks and proportions of the L2 retrotransposon superfamily were observed (Supplementary Figs. 33-36), similar divergence and proportions were also observed in the L2 retrotransposon superfamilies in each tetraploid (Supplementary Figs. 37-40).

3. Studying the syntenies of chr4 in seven (sub)genomes

The long arm of chr4, the wild zebrafish sex chromosome[35,36], has little orthology to the genomes of common carp[12] and goldfish[19]. The long arms of two homoeologous chr4 (chrA4 and chrB4) in the goldfish subgenomes lack collinearity[19] with the total collinear gene pairs of 382 in two chr4 (Supplementary Fig. 46a and Supplementary Table 18). However, we found significantly more collinear pairs (600) between two homoeologous chr4 in common carp (Supplementary Fig. 46b) than in goldfish (Chi-square P value = 1.69×10^{-31}). Interestingly, two chrA4 had lower protein collinearity (399) than two chrB4 (551, Supplementary Fig. 46c, Chi-square P value = 3.19×10^{-15}). These two results suggest different conservation levels in chr4 between two tetraploids and between two lineages.

4. The *Ka/Ks* distributions of four types of homoeologues

In each tetraploid we classified 2,096 homoeologous pairs into four types: hosted subA genes in the A subgenome, exchanged subA genes in the B subgenome, hosted subB genes in the B subgenome, and exchanged subB genes in the A subgenome. First, the *Ka/Ks* distributions of the hosted genes in two subgenomes showed no significant difference (Fig. 3a and Supplementary Table 23). Second, the hosted genes in different chromosomes of the same subgenome were under variable purifying selections. For instance, *Ka/Ks* ratios ranged from 0.159 to 0.245 in the common carp A subgenome and from 0.161 to 0.226 in the B subgenome. Third, the exchanged genes and hosted genes in the same chromosomes were also under the symmetric purifying selection (Supplementary Tables 24-27).

5. Domestication of common carp

Population resequencing of multiple geographical populations suggested distinct origins of the European subspecies and Asian subspecies since the speciation of common carp[12]. Domestication has also accelerated common carp diversity, producing phenotypic changes in growth rate, temperature and hypoxia tolerance, body colour, scale pattern, and survival rate. Nevertheless, knowledge of the genome-wide genetic variations underlying common carp breeding remains limited. Bred from the ‘Yellow river’ (YR) strain since 2004, the ‘Furui’ (FR) strain had the improved growth rate (~39.2%) and survival rate (~8.9%)[37] compared with many local strains.

6. Genomic variations in three common carp strains

For each individual, genome resequencing data were generated with an average depth of ~8.2 × (Supplementary Fig. 86a). After filtering out the non-uniquely mapped reads, the mapping rate of the clean reads to the carp reference genome and the average genome coverage were 92.3% and 84.4%, respectively (Supplementary Figs. 86b and 86c).

We obtained a basic set of 59,404,579 SNPs (3.5% of genome size, one SNP per 28.3 bp), including 57,435,243 SNPs in the FR strain, 57,565,118 SNPs in the YR strain, and 57,627,050 SNPs in the SP strain. A core set of 57,049,657 SNPs was shared among the three strains (Supplementary Fig. 87a). A set of 15,208,494 short insertions and deletions (Indels) (0.9% of genome size, one Indel per 110 bp) were identified, including 14,700,154 Indels in the FR strain, 14,806,014 Indels in YR strain, and 14,919,769 Indels in the SP strain. A core set of 14,371,781 Indels was covered by all three strains (Supplementary Fig. 87b). The ratio of transition to transversion (Ts/Tv) in the basic SNP set was estimated to be 1.04. Approximately 51.1% of the SNPs were C/T transitions (Supplementary Fig. 88a). Nearly 90% of SNPs were located in intergenic regions or intronic regions, and only 3.19% were located in exonic regions (Supplementary Fig. 88b). The percentage of synonymous SNPs (52.24%) was greater than that of nonsynonymous SNPs (44.02%, Supplementary Fig. 88c). Indels of one or two bases accounted for 46.9% of all Indels (Supplementary Fig. 89a). Approximately 92.29% of Indels were located in intergenic regions or intronic regions, and 0.59% were located in exonic regions (Supplementary Fig. 89b). An estimated 60% of Indels in the exonic regions might cause frameshifts, and 2.8% of Indels might lead to stop gain or loss (Supplementary Fig. 89c).

7. Comparing the tetraploidization events in vertebrates

Two earliest tetraploidization events in vertebrates, including an auto-tetraploidization and then an allo-tetraploidization event, were estimated to precede the divergence of jawed vertebrates and lamprey and the divergence of cartilaginous and bony fish, respectively[38]. These two events were too ancient to compare the early subgenome structure evolution processes with those recently occurring in the common carp and goldfish. Therefore, we compared the subgenome structure parallelism in the allo-tetraploid common carp and goldfish with the observations in the other modern tetraploid vertebrates, including allo-tetraploid *Xenopus laevis*[27], auto-tetraploid salmonids[39,40], and auto-tetraploid sterlet sturgeon[41]. First, after the tetraploidization event, common carp and goldfish did not experience large genomic reorganization (including chromosome fusion or loss) occurring in the latter vertebrates[27,39-41]. Second, the retention rates of the homoeologues after the species-specific tetraploidization event in common carp (70.8%) and goldfish (65.2%) were higher than in the allo-tetraploid frog (56.4%, polyploidization event at 17~18 Mya)[27] and auto-tetraploid Salmonids (55% in Atlantic salmon[40] and 48% in rainbow trout[39], Salmon-specific tetraploidization event at 80~100 Mya) but equivalent to that in the auto-tetraploid sterlet[41] (70%, polyploidization event at ~180 Mya). Because of the high retention rate, the protein-coding gene number ratios between two subgenomes in both allo-tetraploid common carp

(0.958) and goldfish (0.899) were significantly higher than in the allo-tetraploid frog also having clearly divided subgenomes (0.743, Chi square P values of 2.35×10^{-56} and 1.22×10^{-32}, respectively). These data indicate common carp and goldfish to be unique models to study the early subgenome structure evolution in polyploid vertebrates.

Supplementary Figures

Supplementary Fig. 1. The photos of *P. guichenoti*, *P. tetrazona*, and three sequenced common carp strains

(a)

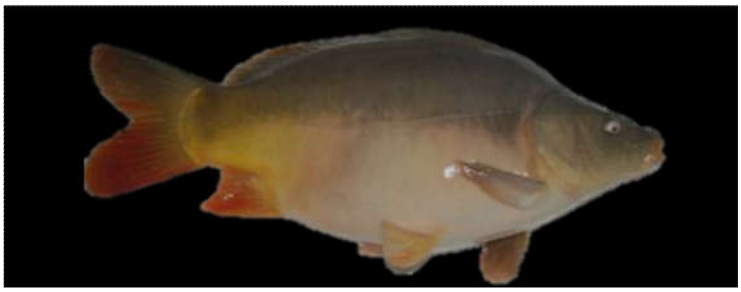

(b)

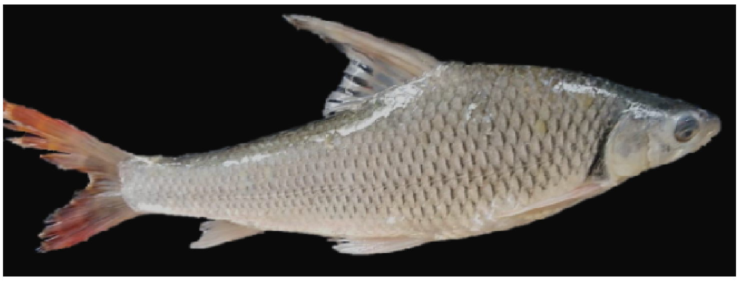

(c)

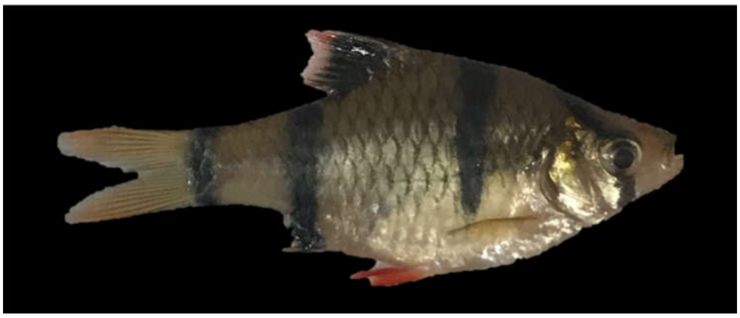

(d)

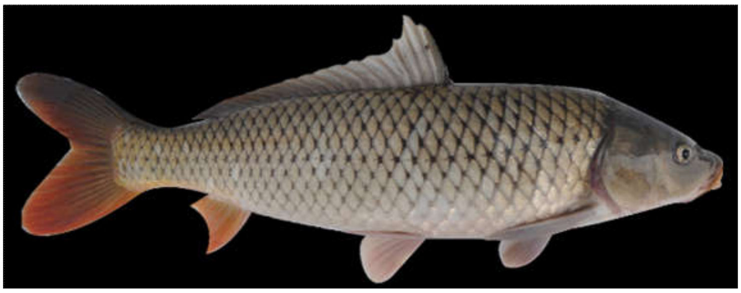

(e)

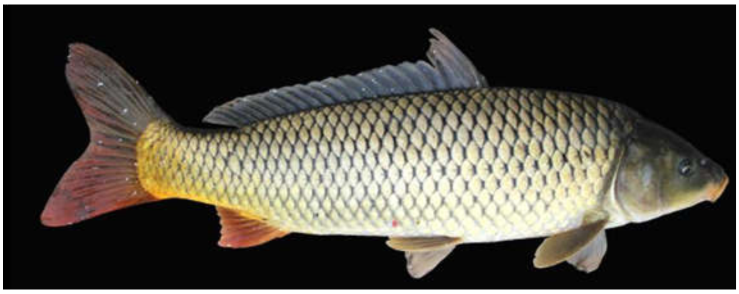

(a) common carp var. ‘Song pu’ (SP strain) photoed by Dr. Zhi Ying Jia; (b) *P. guichenoti* photoed by Dr. Ju-Hua Yu; (c) *P. tetrazona* photoed by Dr. Jiong-Tang Li; (d) common carp var. ‘Fu rui’ (FR strain) photoed by Dr. Zai Jie Dong; (e) common carp var. ‘Yellow river’ (YR strain) photyoed by Dr. Jiong-Tang Li.

Supplementary Fig. 2. Sampling sites of the sequenced fish

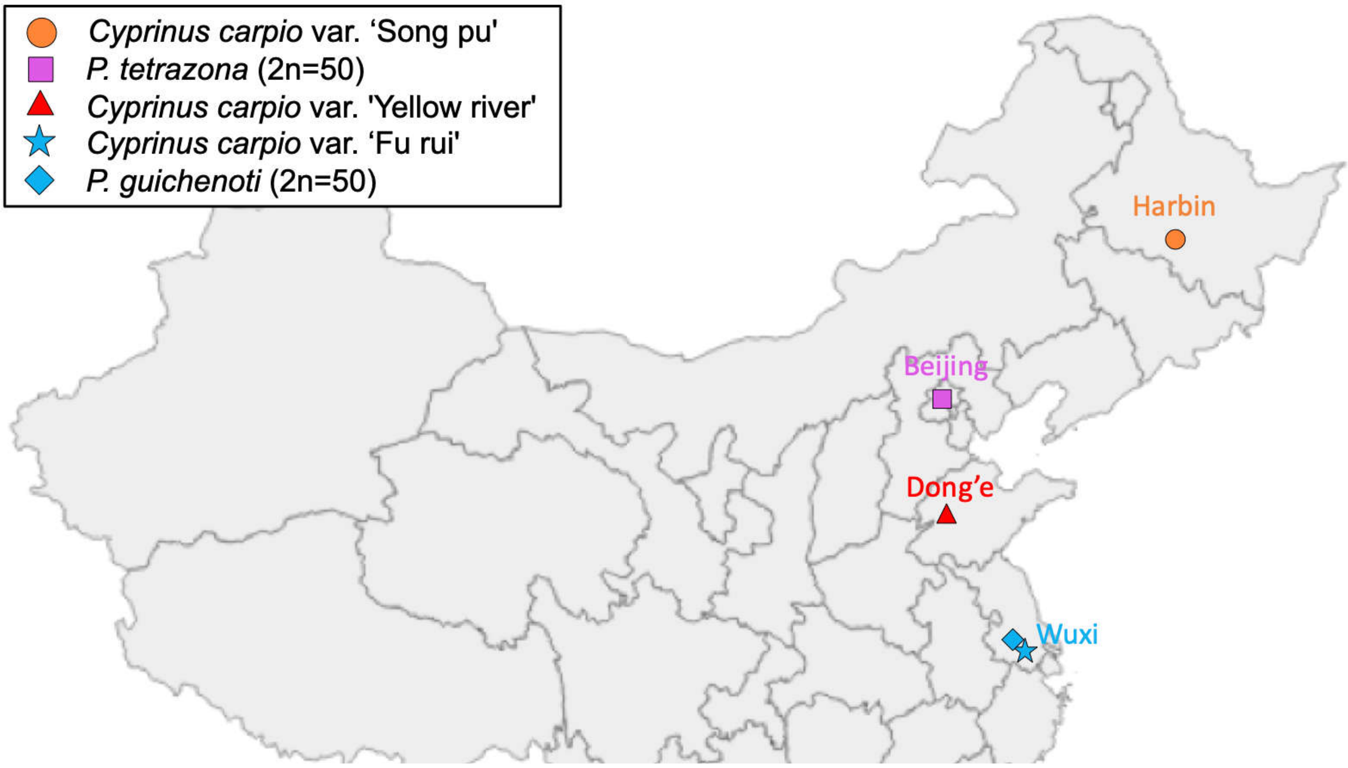

The map was generated with the python function of ‘pyechart’.

Supplementary Fig. 3. K-mer distribution of reads to estimate the genome sizes of *P. guichenoti* and *P. tetrazona*

(a)

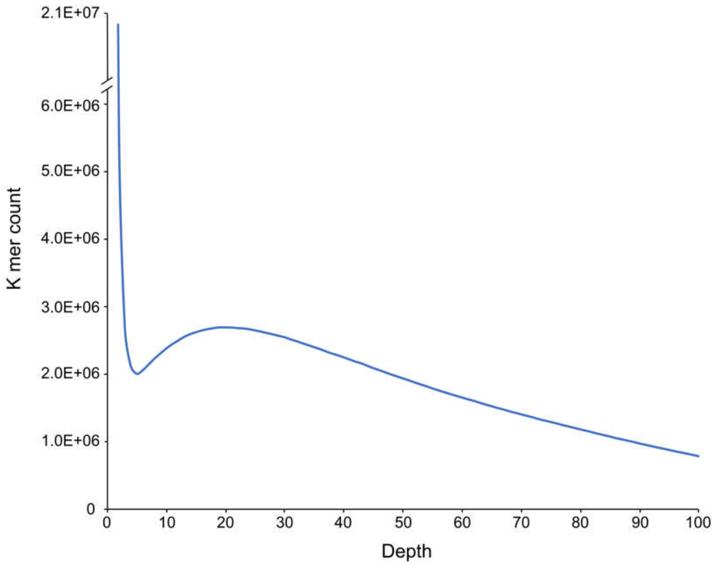

(b)

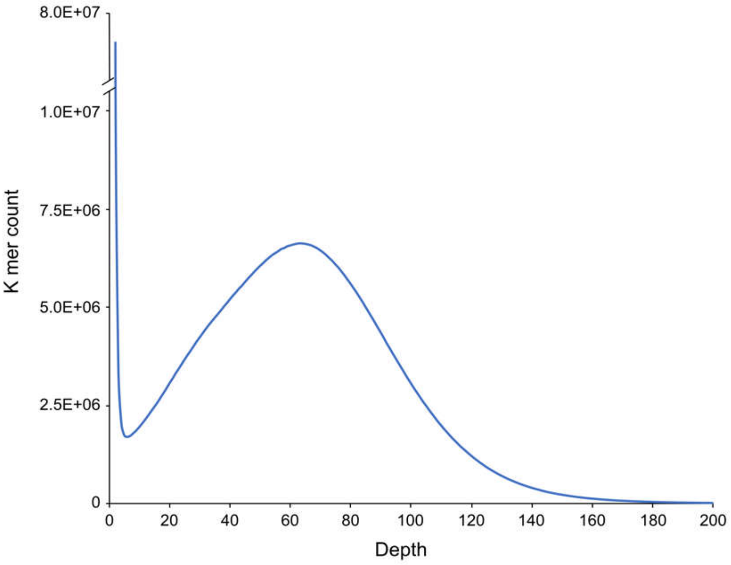

The k-mer distributions of Illumina paired-end reads based on k value of 21 in *P. guichenoti* (a) and *P. tetrazona* (b). K-mer occurrences (x axis) are plotted against their frequencies (y axis).

Supplementary Fig. 4. Genome landscape of *P. guichenoti*

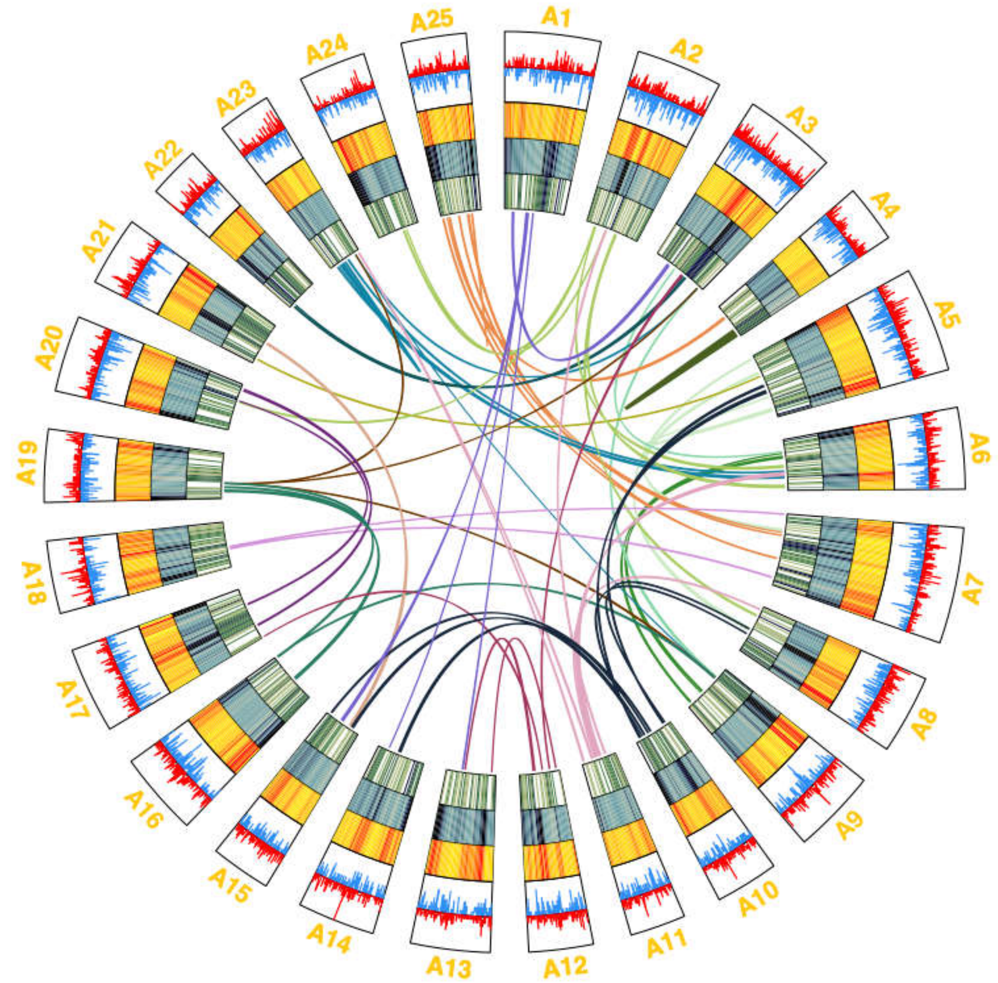

Multi-dimensional display of genomic components of *P. guichenoti.* The density was calculated per 1 Mb. The red and blue histograms show the gene densities in the plus and minus strand, respectively. The orange, indigo and green heatmaps plot the densities of interspersed repeats, SSRs, and LTRs, respectively. The intramural lines link different genomic loci to indicate the syntenic regions.

Supplementary Fig. 5. Genome landscape of *P. tetrazona*

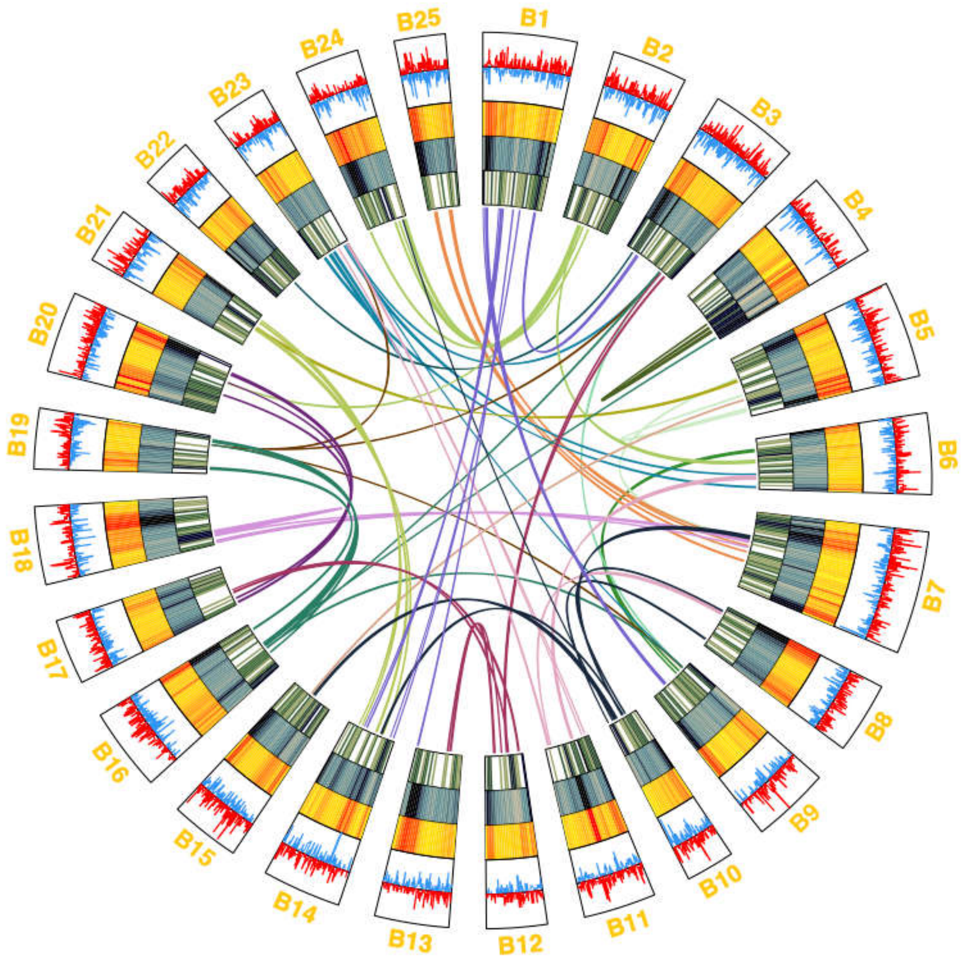

Multi-dimensional display of genomic components of *P. tetrazona.* The density was calculated per 1 Mb. The red and blue histograms show the gene densities in the plus and minus strand, respectively. The orange, indigo and green heatmaps plot the densities of interspersed repeats, SSRs, and LTRs, respectively. The intramural lines link different genomic loci to indicate the syntenic regions.

Supplementary Fig. 6. Mate-pair/Paired-end insert distribution of *P. guichenoti*, *P. tetrazona*, and common carp

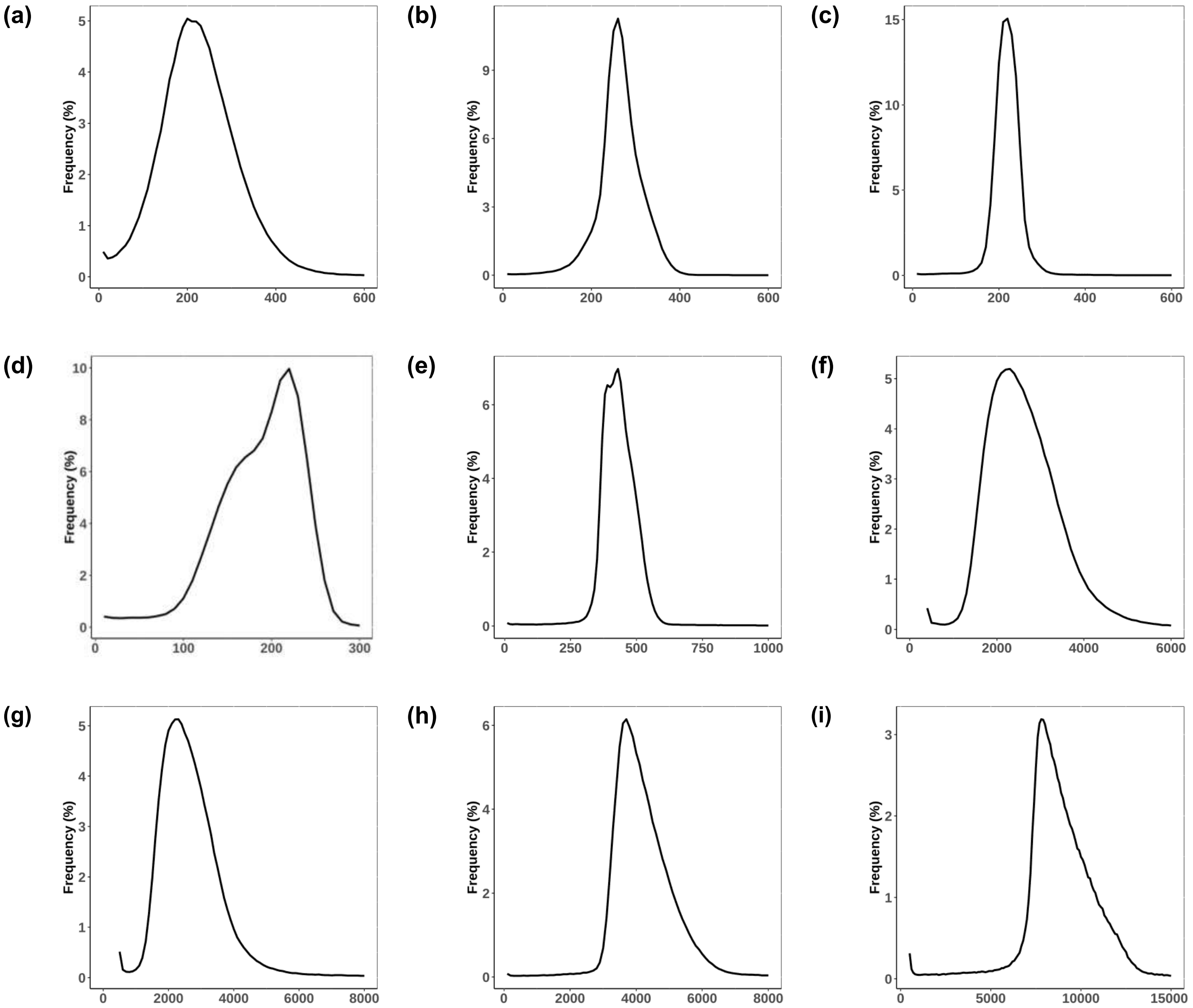

(a) The insert size distribution of *P. guichenoti* Illumina paired-end library. (b) The insert size distribution of *P. tetrazona* Illumina paired-end library. (c-i) The insert size distributions of seven published paired-end/mate-pair libraries in carp genome sequencing[12], listed in Supplementary Table 1.

Supplementary Fig. 7. HiC chromosome contact map of common carp

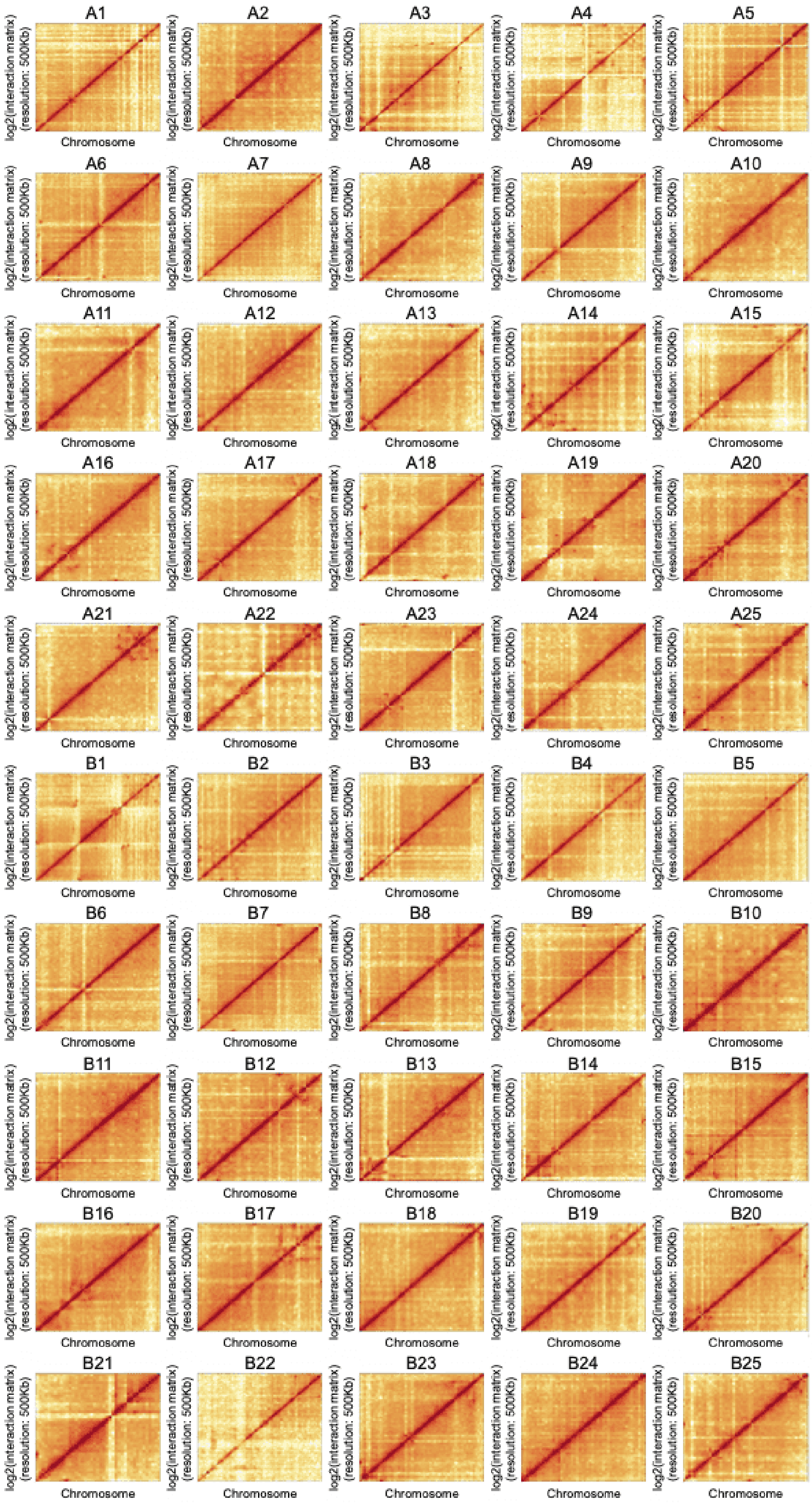

Each block represents a HiC contact between two genomic loci within a 500-kb window. The intensity of pixels represents the count of HiC links on a logarithmic scale. Darker color of a block indicates higher contact intensity.

Supplementary Fig. 8. HiC chromosome contact map of *P. guichenoti*

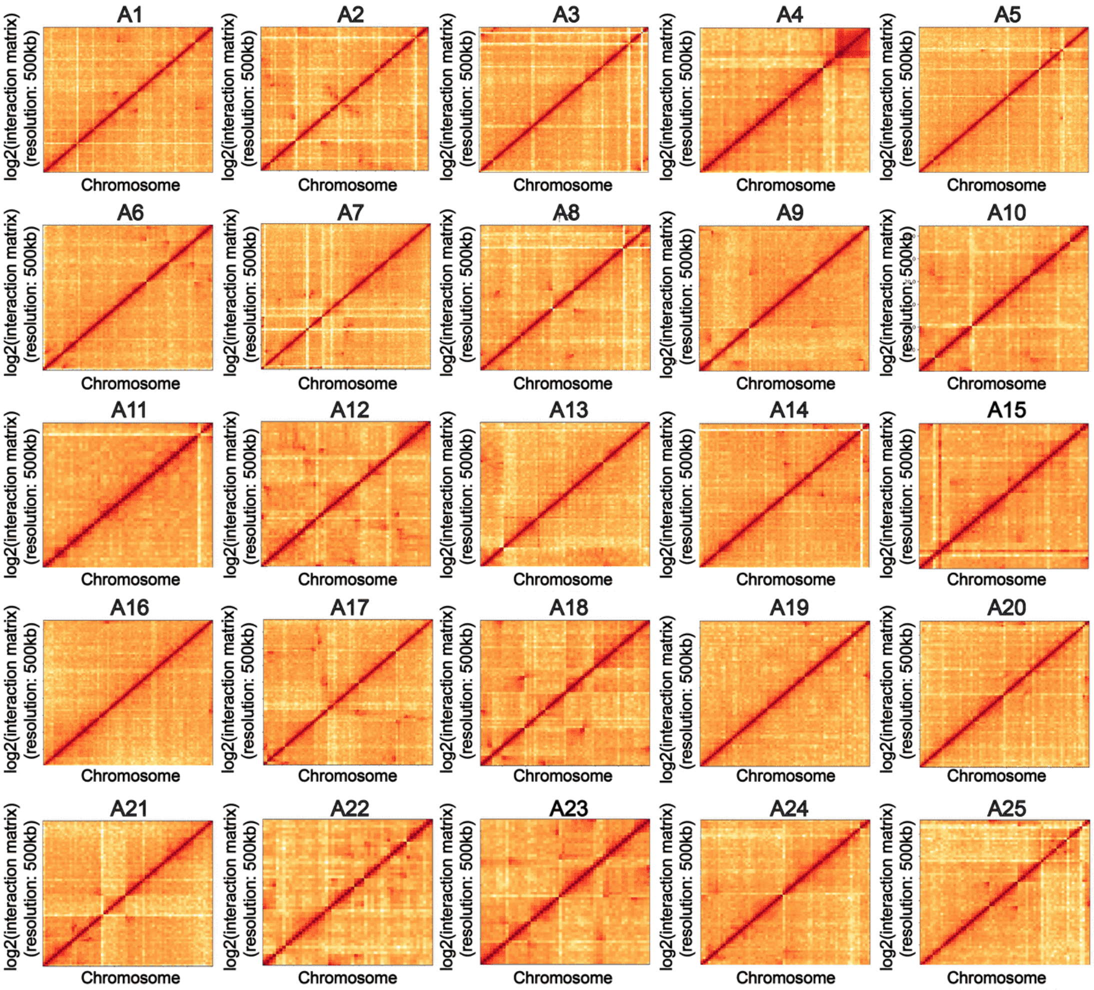

Each block represents a HiC contact between two genomic loci within a 500-kb window. The intensity of pixels represents the count of HiC links on a logarithmic scale. Darker color of a block indicates higher contact intensity.

Supplementary Fig. 9. HiC chromosome contact map of *P. tetrazona*

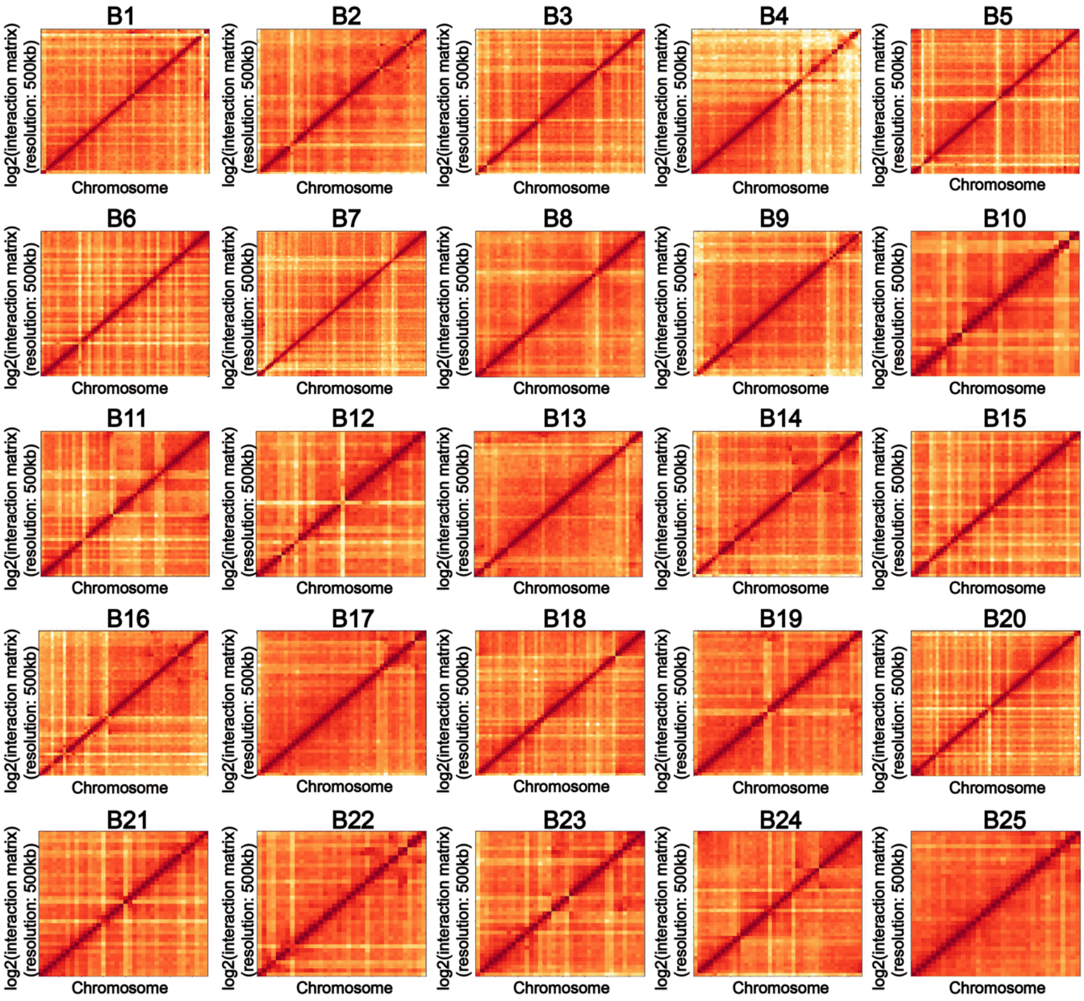

Each block represents a HiC contact between two genomic loci within a 500-kb window. The intensity of pixels represents the count of HiC links on a logarithmic scale. Darker color of a block indicates higher contact intensity.

Supplementary Fig. 10. The correlation between the chromosome-level-assembly and the common carp genetic map

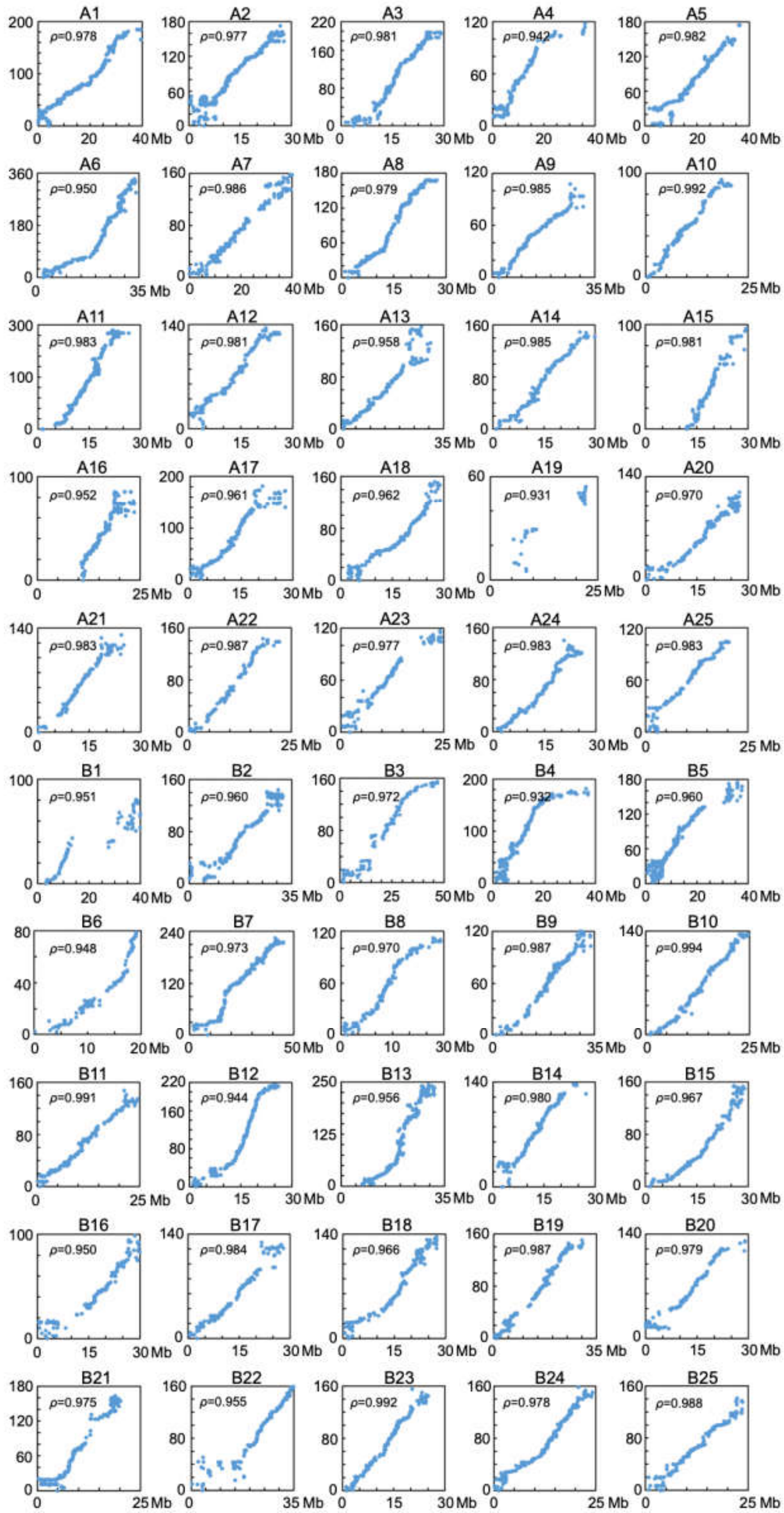

Scatter plots with dots represented the physical position on the chromosome (x axis) versus the genetic map position (y axis). The ρ is the Pearson correlation coefficient.

Supplementary Fig. 11. MUMmer dotplots of previous five assemblies compared to the current assembly

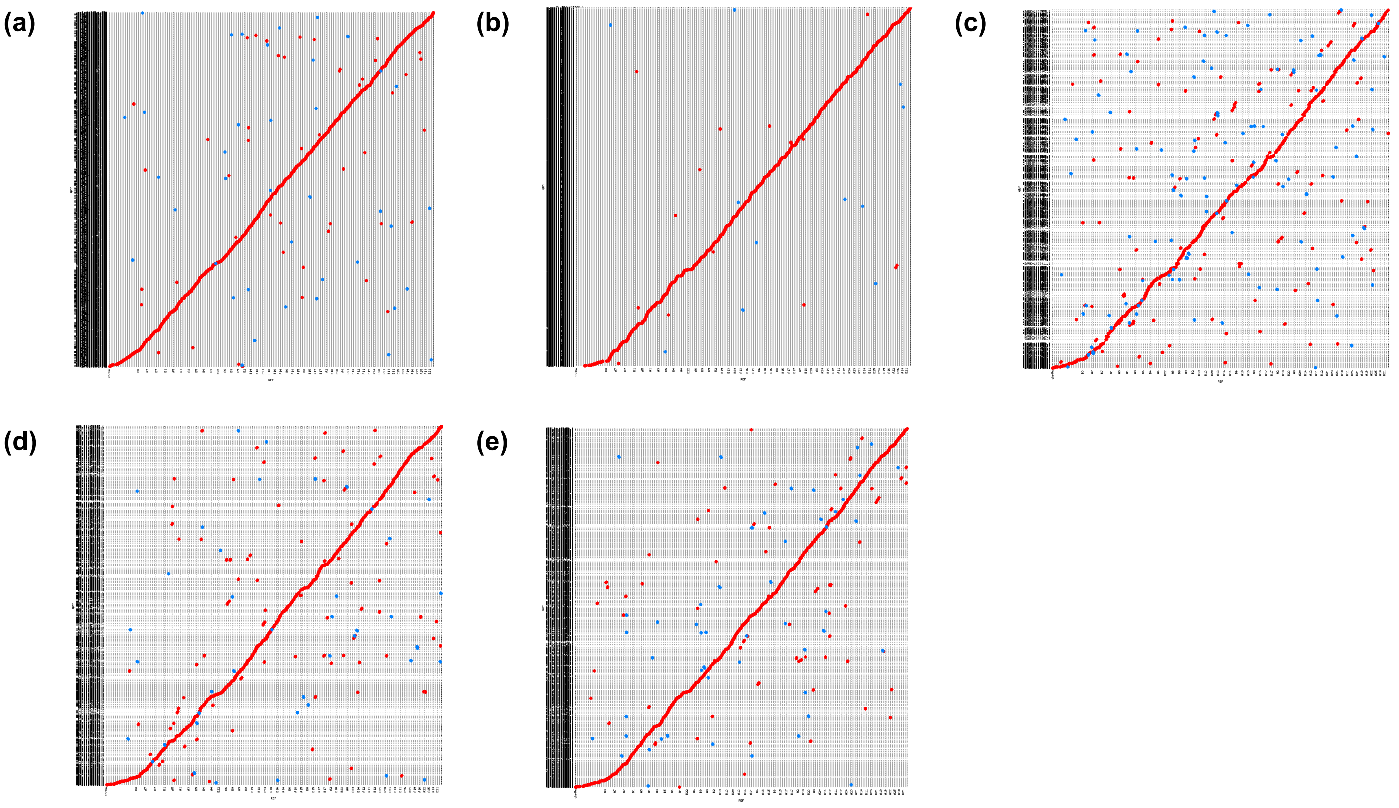

Alignment dotplots show the structural agreements between the previous assemblies (y axis, listed in Supplementary Table 3) and the current assembly (x axis). The panels showed the alignments with GCA_000951615.2[12] (a), GCA_001270105.1[13] (b), GCA_004011575.1[14] (c), GCA_004011595.1[14] (d), and GCA_004011555.1[14] (e), respectively. Boundaries of sequences from all assemblies are represented as dotted lines (vertical and horizontal, respectively). The red and blue points represent the forward-strand matches and reverse complement matches, respectively. Sequences are oriented and ordered to match the chromosomes using mummerplot[42] command with '--filter --layout' option.

Supplementary Fig. 12. Insert size distributions of common carp BAC end sequences in the current assembly and previous assemblies.

(a)

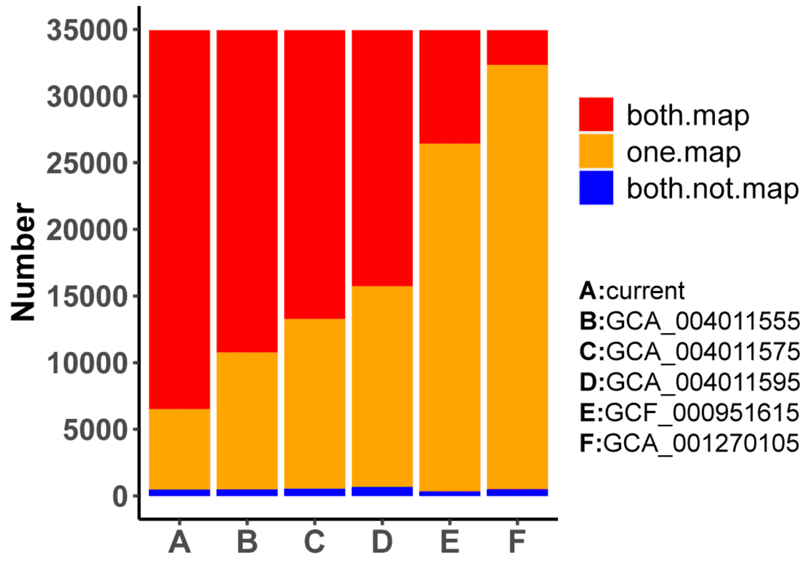

(b)

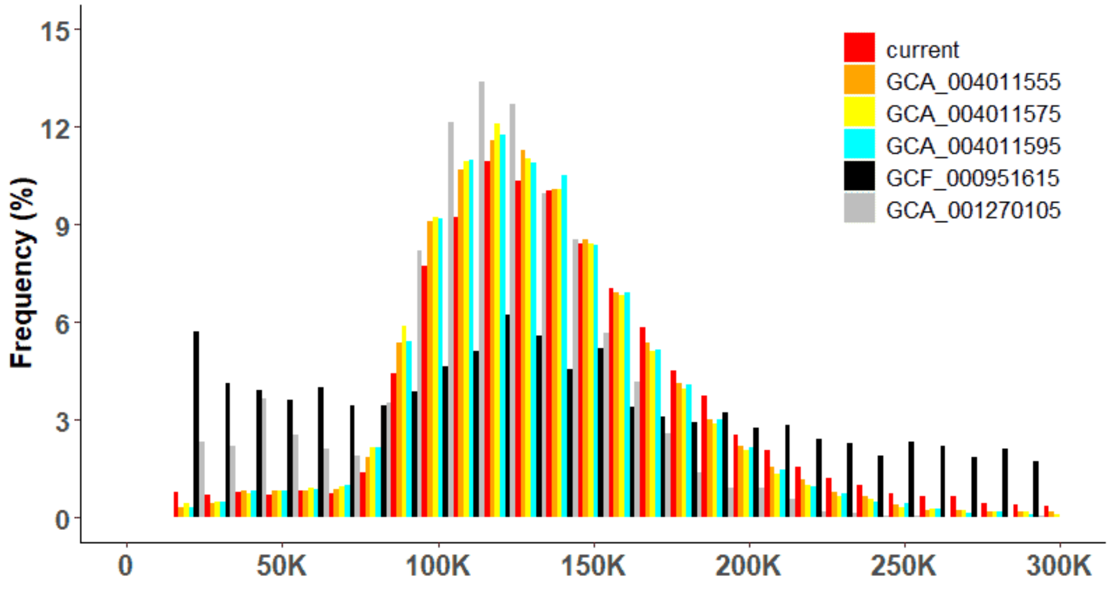

(a) The number of three types of aligned BAC ends in the carp genome. 'both.map' indicates that two ends of one BAC clone are aligned to the same genome sequence. 'one.map' means that only one end of a BAC clone is mapped to the carp genome. 'both.not.map' represents that both two ends of a BAC clone are not aligned to the genome. (b) The insert size distribution of BAC ends exhibited a standard normal distribution in the current assembly.

Supplementary Fig. 13. Genome landscape of goldfish

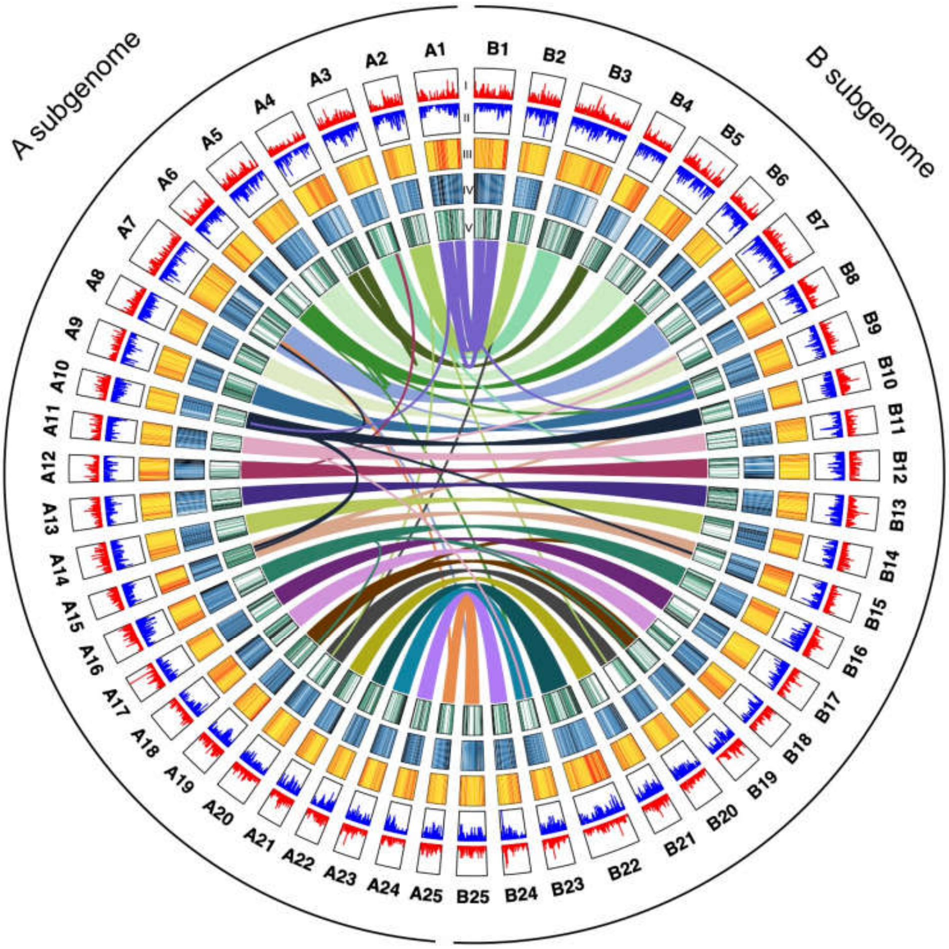

Multi-dimensional display of genomic components of goldfish. The density was calculated per 1 Mb. The red histograms and the blue histograms show the gene densities in the plus strand and the minus strand, respectively. The orange, indigo and green heatmaps plot the densities of interspersed repeats, SSRs, and LTRs, respectively. The intramural lines link different genomic loci to indicate the syntenic regions.

Supplementary Fig. 14. The distributions of bootstrap values and branch lengths in 3,171 heptad families

(a)

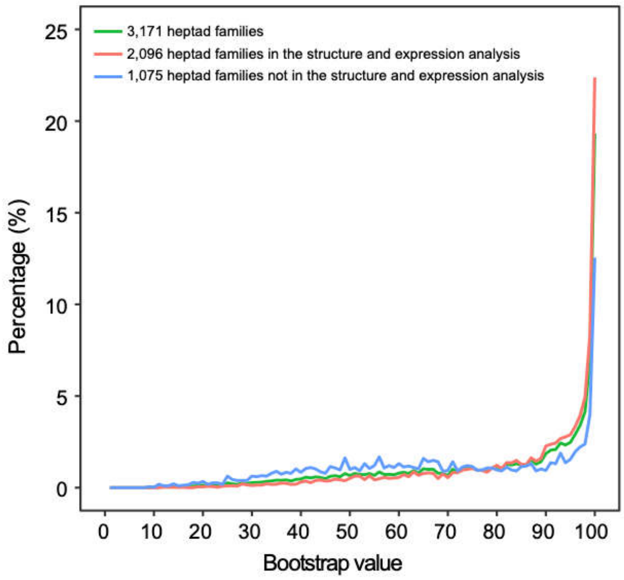

(b)

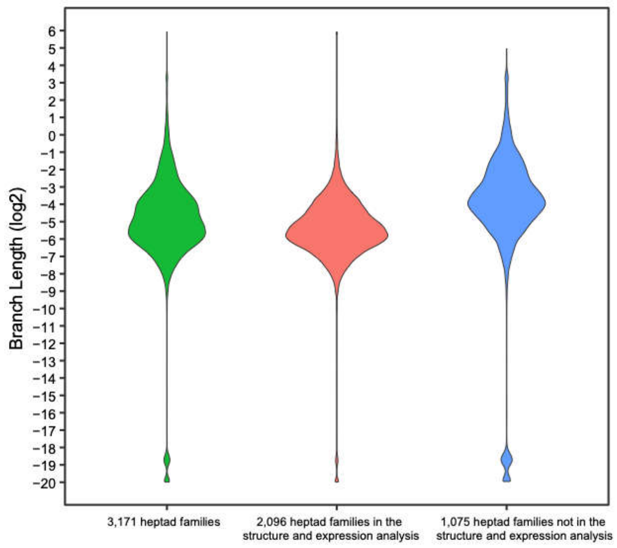

Each in the 3,171 heptad families (green distribution) comprises a single gene in each diploid fish, two common carp genes, and two goldfish genes. These families were classified into two groups, 2,096 families (red distribution) and 1,075 (blue distribution). In the 2,096 families, the common carp genes and goldfish genes were clearly differentiated into the subA and subB genes. On the contrary, the 1,075 families had no clearly differentiated subA and subB genes. Only the 2,096 families were used in the following structure and expression analysis including the identification of homoeologous exchange events. All bootstrap values and branch lengths of the above three types of families were shown in (a) and (b). At least 93.3% of all branches in the 2,096 families have bootstrap values over 50.

Supplementary Fig. 15. Chromosome size comparisons among genomes of the common carp A, common carp B, goldfish A, goldfish B, *P. guichenoti*, and *P. tetrazona*

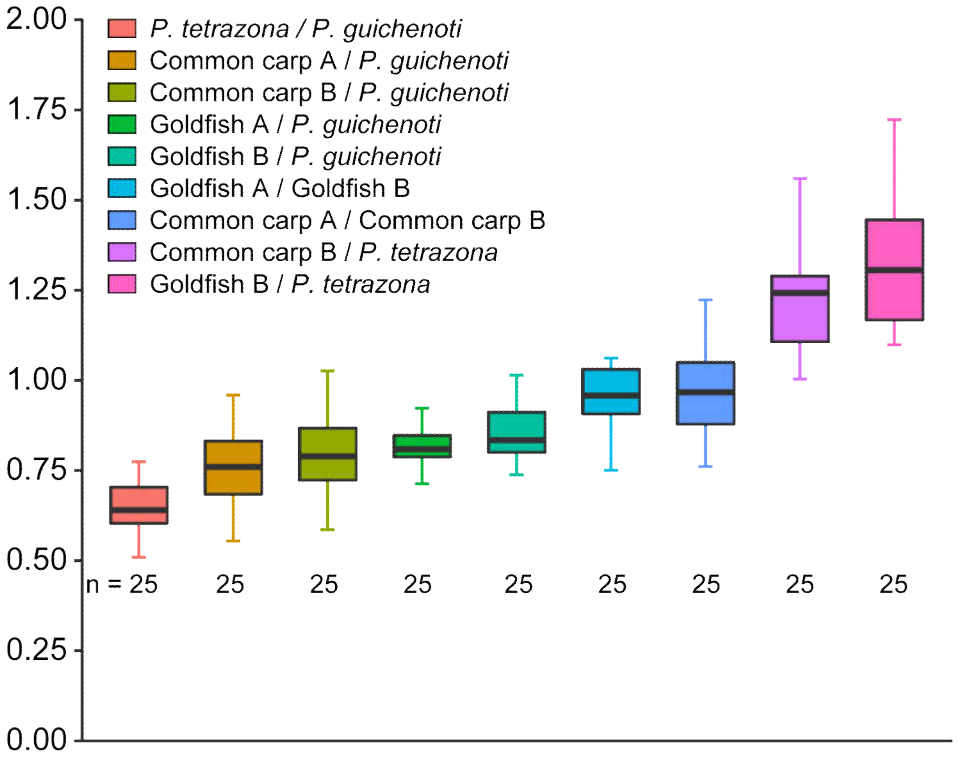

We defined the orthologous chromosomes in genomes of *P. guichenoti*, *P. tetrazona*, common carp A, common carp B, goldfish A, and goldfish B by comparison with zebrafish genome (detailed in Supplementary Methods 5). In two selected genomes, we calculated the size ratio of each orthologous chromosome pair or each homoeologous chromosome pair. The boxplots show the 25th, 50th and 75th percentiles. The upper and lower whiskers correspond to the third quartile + 1.5 × interquartile range (IQR) and the first quartile - 1.5 × IQR, respectively. The n values represent 25 pairs of the homoeologous chromosomes or the orthologous chromosomes.

Supplementary Fig. 16. The numbers of *P. tetrazona* reads mapped to the common carp subgenomes and exchanged gene regions

(a)

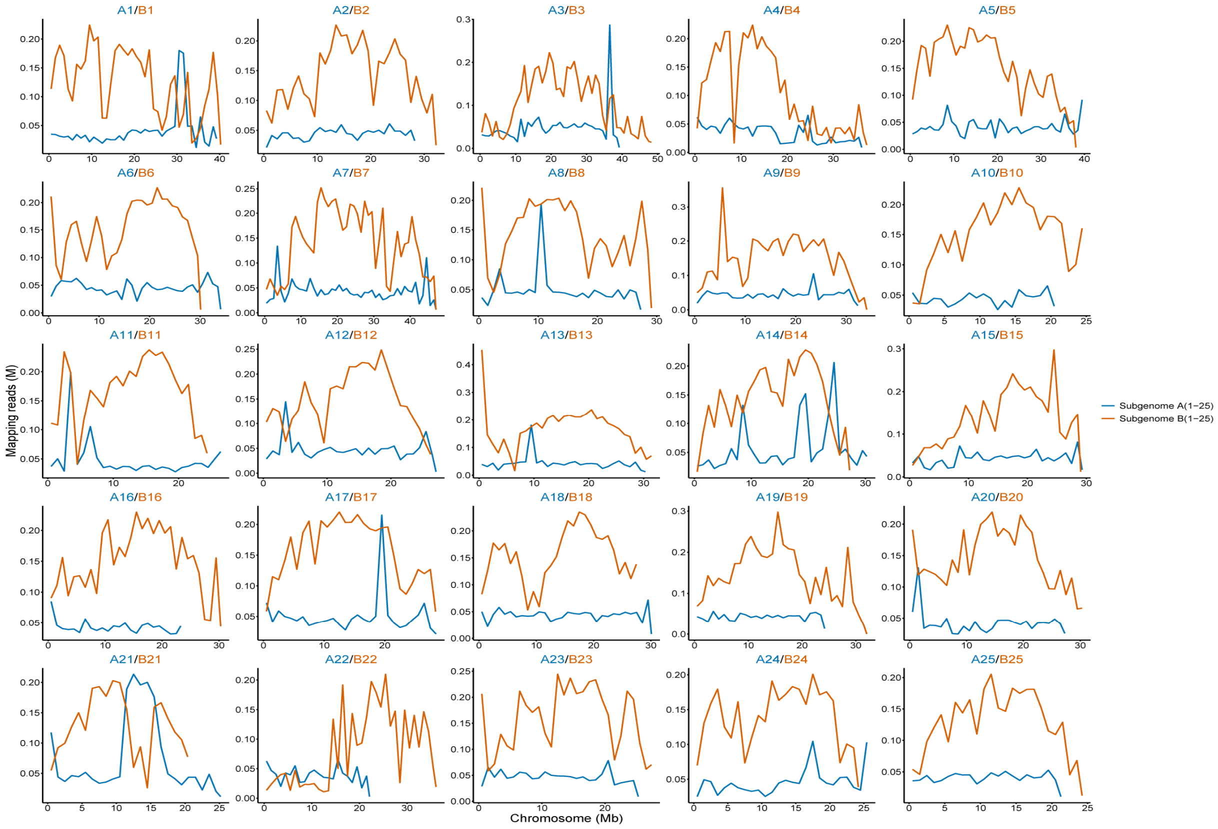

(b)

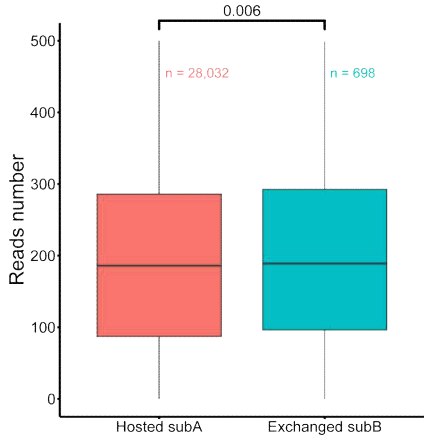

(c)

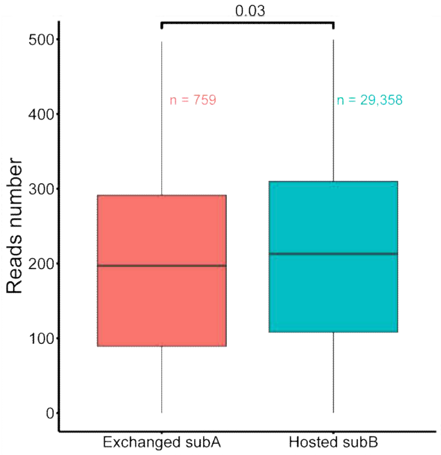

(a) Distribution of *P. tetrazona* read numbers mapped to 25 common carp homoeologous chromosome pairs (drawn in 1 Kb non-overlapping window). The reads mapped to the A and B subgenomes are colored with blue and orange lines, respectively. (b) Distribution of *P. tetrazona* read numbers in the hosted subA regions and the exchanged subB regions in the A subgenome. (c) Distribution of *P. tetrazona* read numbers in the hosted subB regions and the exchanged subA regions in the B subgenome. In (b) and (c), the numbers of the hosted and exchanged regions were represented as the n values. The definitions of the boxplots and the whiskers in (b) and (c) are consistent with those in Supplementary Fig. 15. The P values were computed with the one-sided Mann-Whitney *U* test.

Supplementary Fig. 17. The numbers of *P. tetrazona* reads mapped to the goldfish subgenomes and exchanged gene regions

(a)

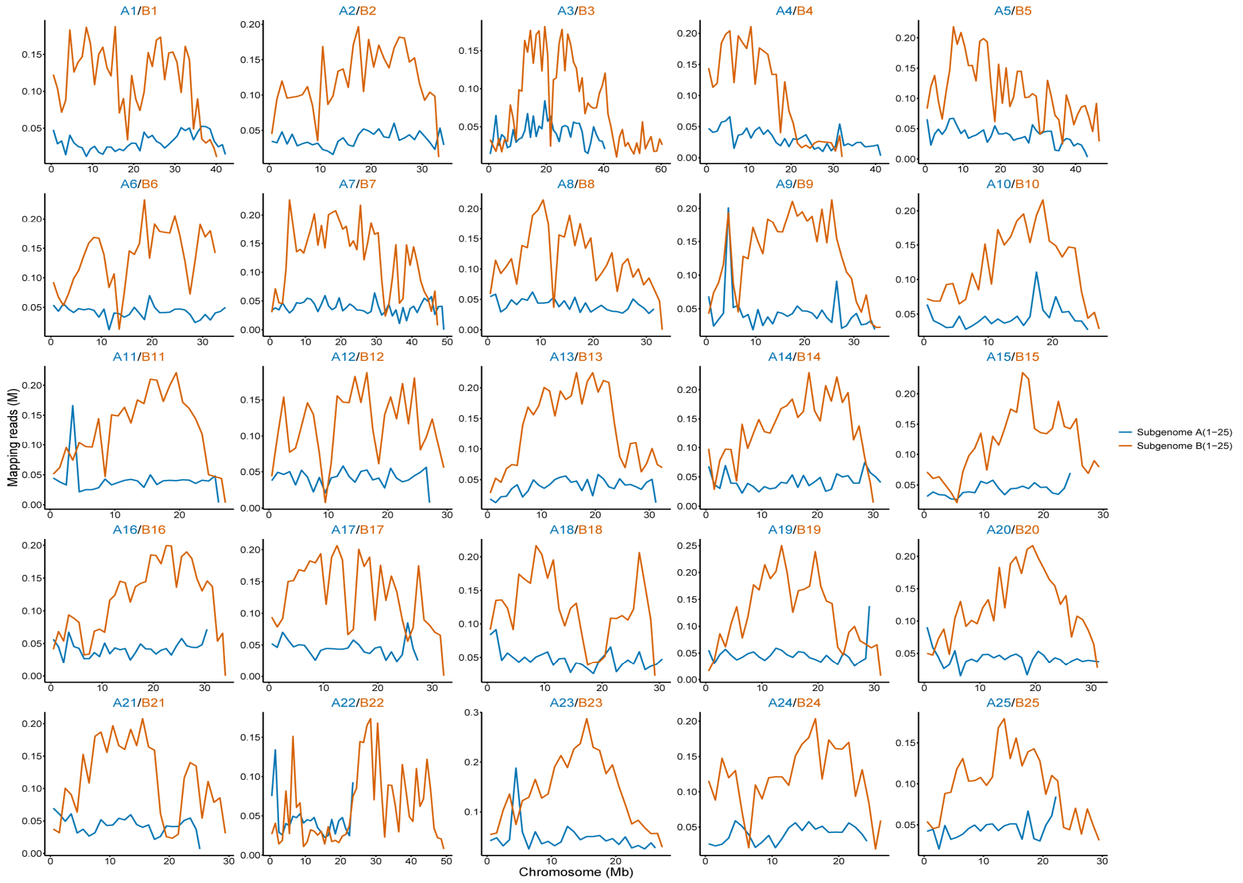

(b)

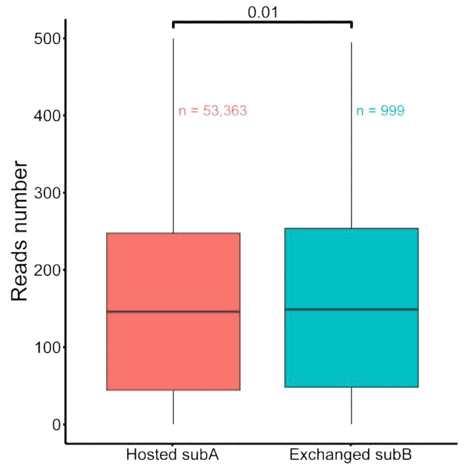

(c)

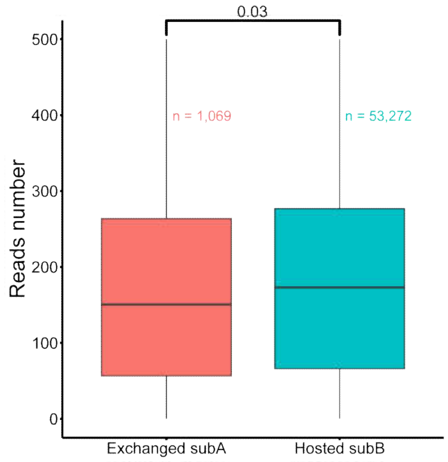

(a) Distribution of *P. tetrazona* read numbers mapped to 25 goldfish homoeologous chromosome pairs (drawn in 1 Kb non-overlapping window). The reads mapped to the A and B subgenomes are colored with blue and orange lines, respectively. (b) Distribution of *P. tetrazona* read numbers in the hosted subA regions and the exchanged subB regions in the A subgenome. (c) Distribution of *P. tetrazona* read numbers in the hosted subB regions and the exchanged subA regions in the B subgenome. The P values in (b) and (c) are computed with the one-sided Mann-Whitney *U* test. In (b) and (c), the numbers of the hosted and exchanged regions were represented as the n values. The definitions of the boxplots and whiskers are the same to those in Supplementary Fig. 15.

Supplementary Fig. 18. The numbers of *P. guichenoti* reads mapped to the common carp subgenomes

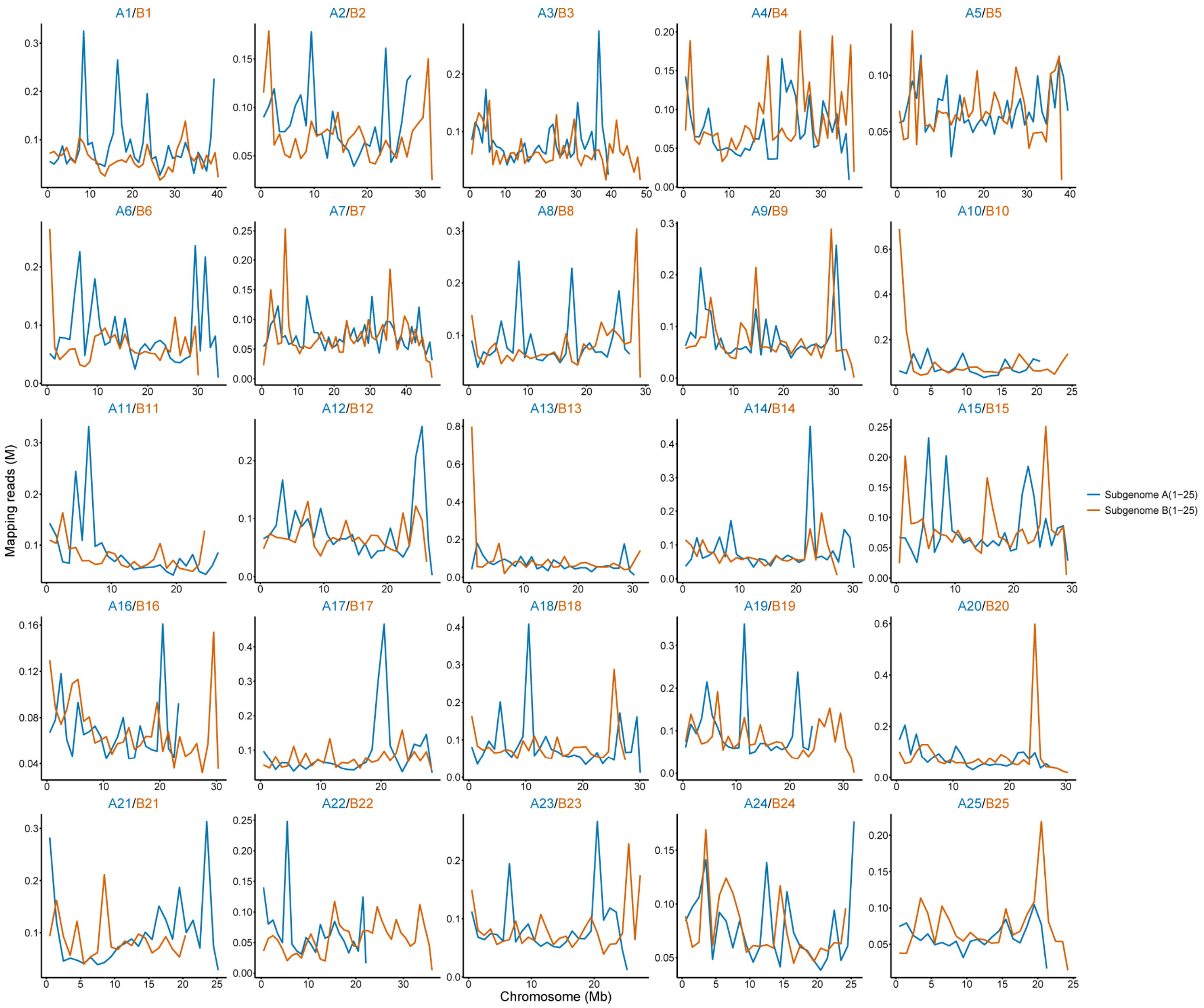

Distribution of *P. guichenoti* reads mapped to 25 common carp homoeologous chromosome pairs (drawn in 1 Mb non-overlapping window). The reads mapped to the A and B subgenomes are colored with blue and orange lines, respectively.

Supplementary Fig. 19. The numbers of *P. guichenoti* reads mapped to the goldfish subgenomes

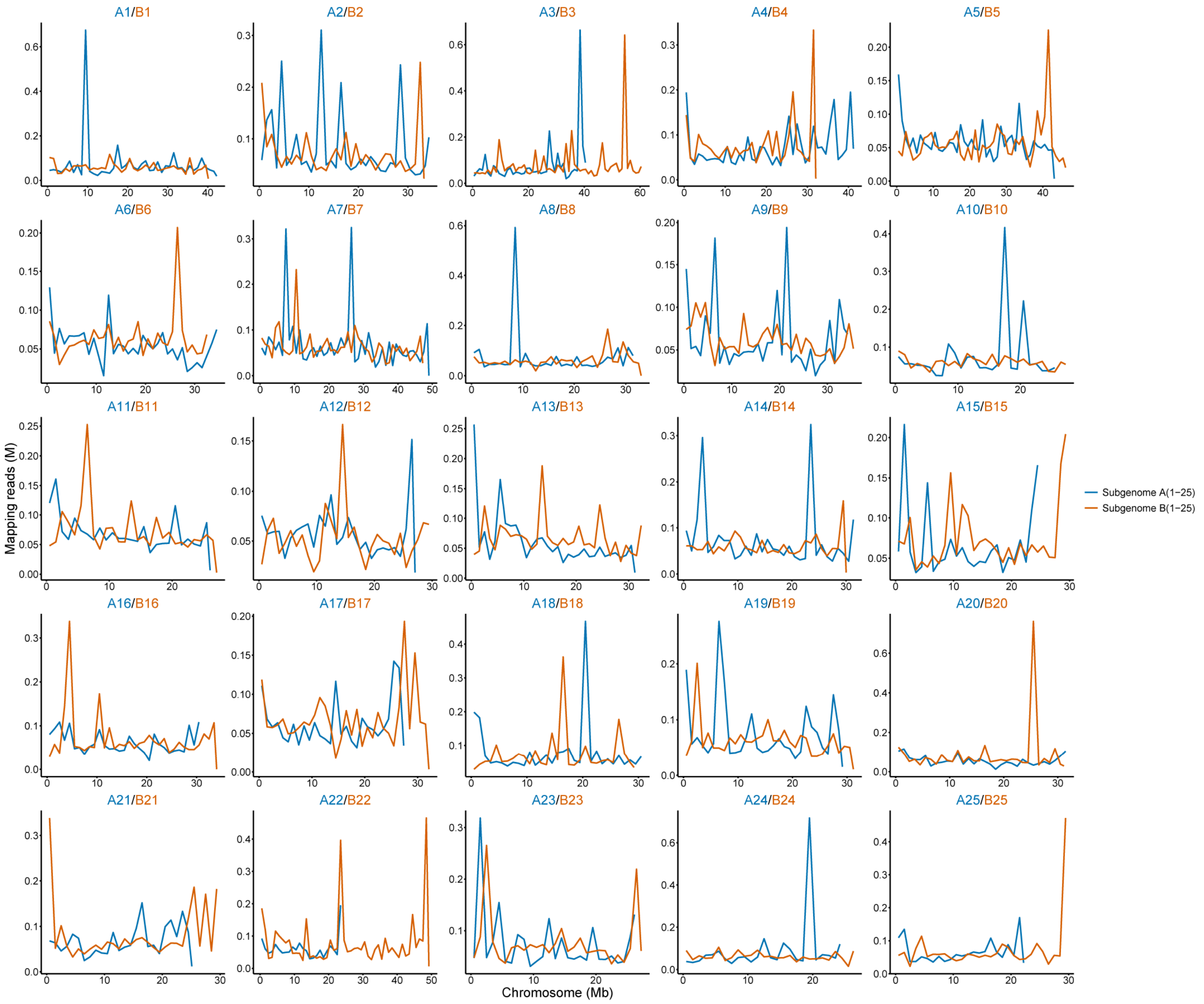

Distribution of *P. guichenoti* reads mapped to 25 goldfish homoeologous chromosome pairs (drawn in 1Mb non-overlapping window). The reads mapped to the A and B subgenomes are colored with blue and orange lines, respectively.

Supplementary Fig. 20. Ratio distributions of mean read numbers between the homoeologous chromosomes in each tetraploid

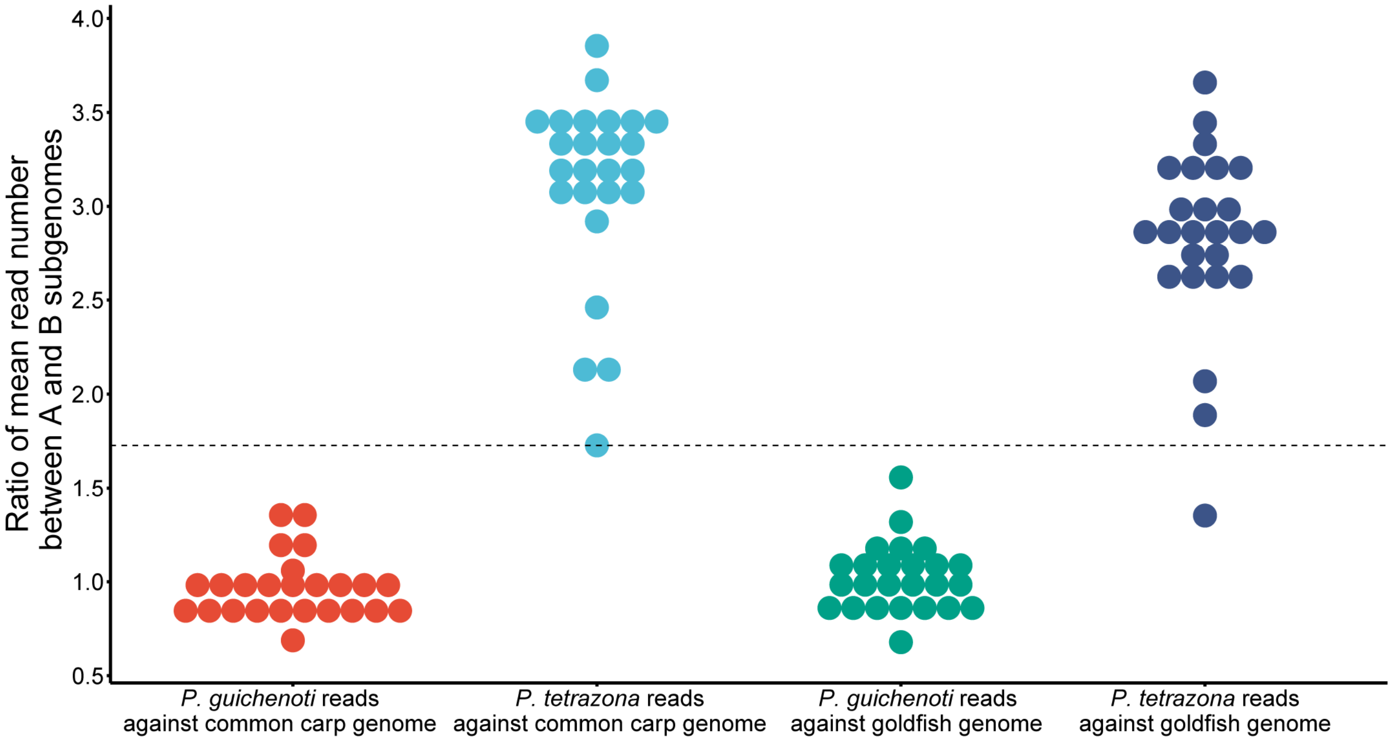

The dash line represents the read number ratio threshold of 1.73 to identify potential HE regions between two subgenomes.

Supplementary Fig. 21. Estimated speciation time using different molecular evolution rates

(a)

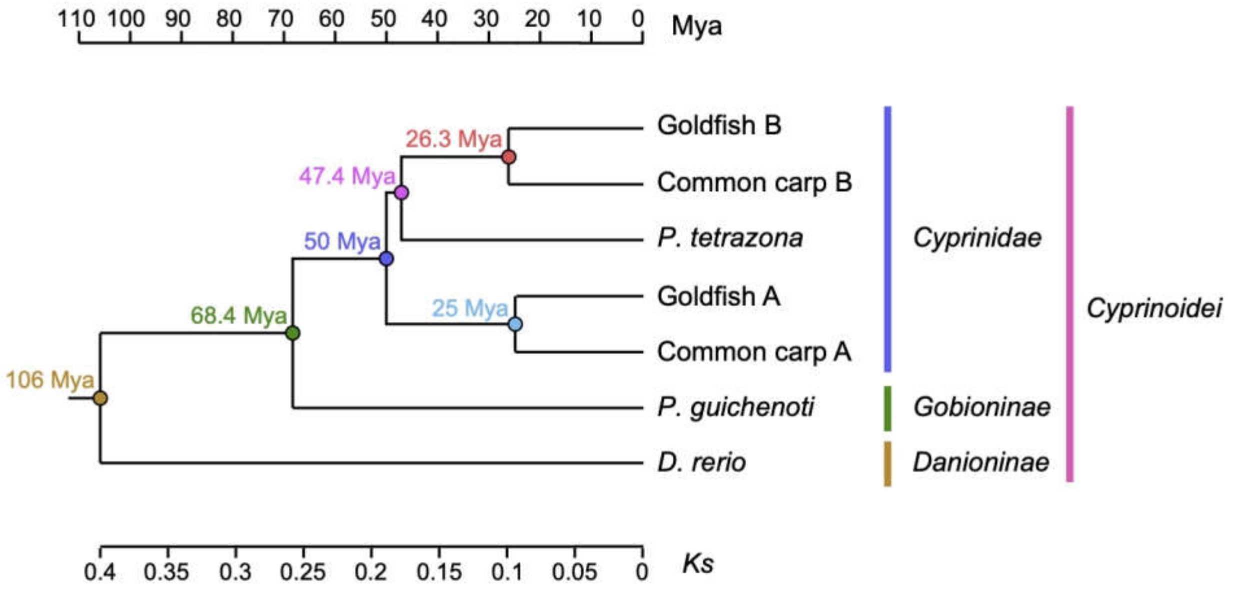

(b)

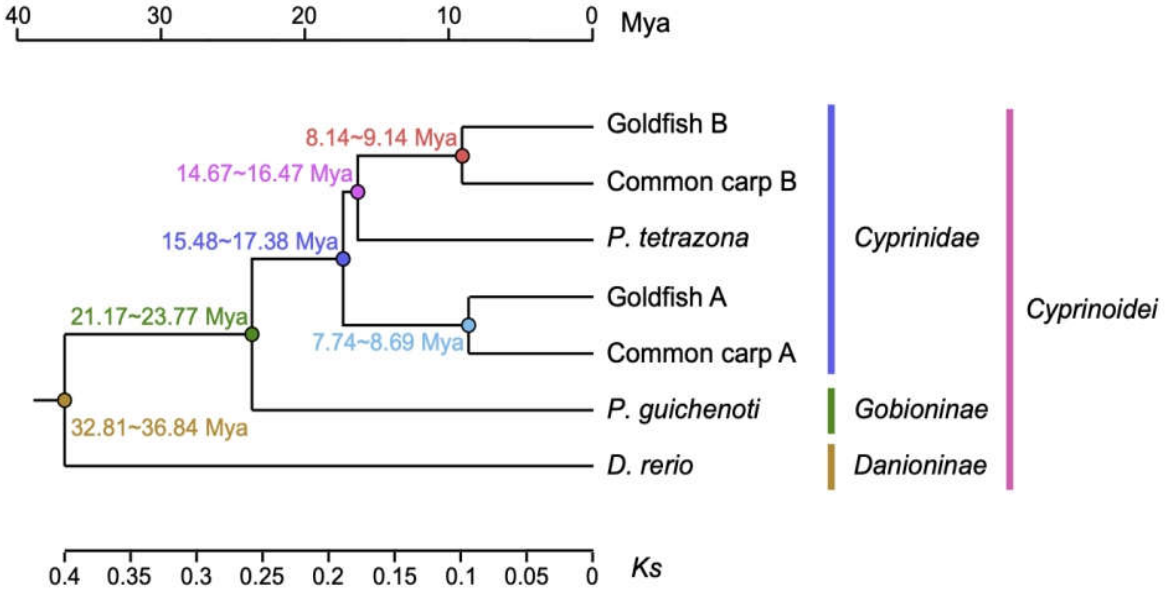

The divergence time between Danionidae and Cyprinidae of 106 Mya[26] (a), and the speciation time between zebrafish and the last common ancestor of the common carp and goldfish of 32.81~36.84 Mya[19] (b) as calibration time were used to estimate each speciation time in the species tree, respectively. The nodes with different colors represent the speciation events.

Supplementary Fig. 22. Exon number, exon size, and protein length comparisons among orthologs and homoeologues in 2,096 pairs

(a)

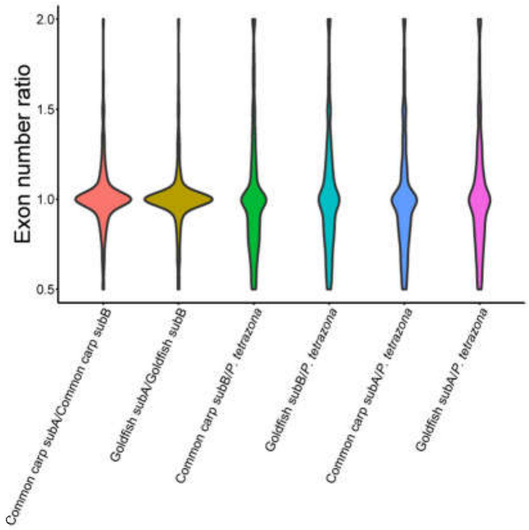

(b)

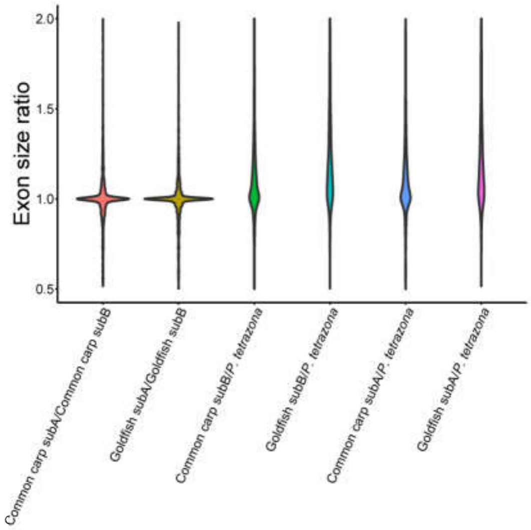

(c)

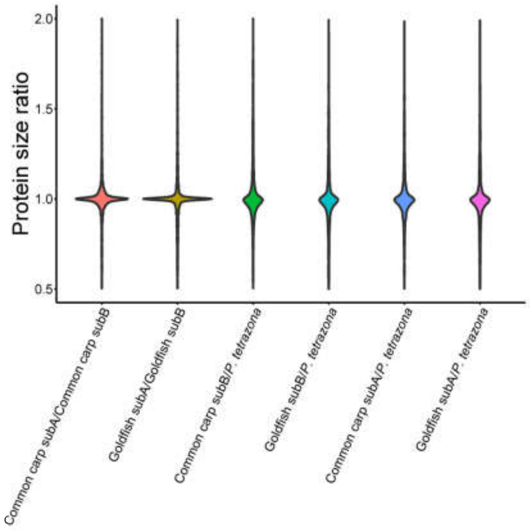

(a) Distribution of the ratio exon number of selected pairs from 2,096 septuplets. (b) Distribution of the exon size ratio. (c) Distribution of the protein length ratio.

Supplementary Fig. 23. mRNA identity and protein identity comparisons among orthologs and homoeologues

(a)

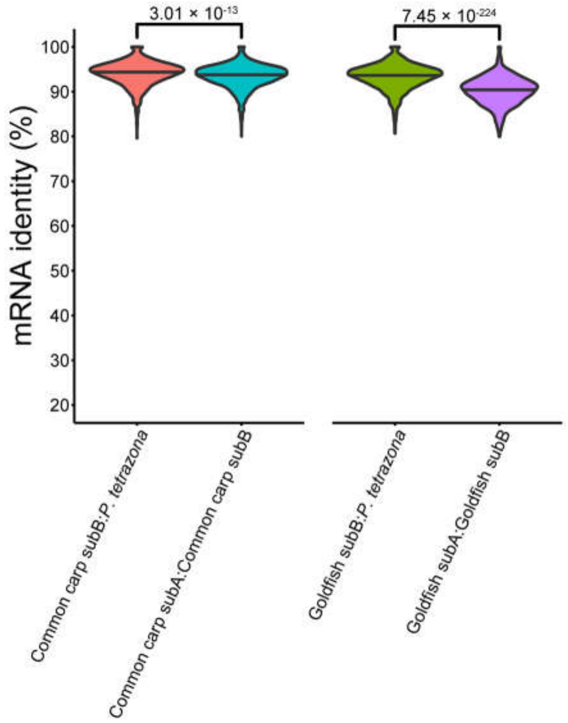

(b)

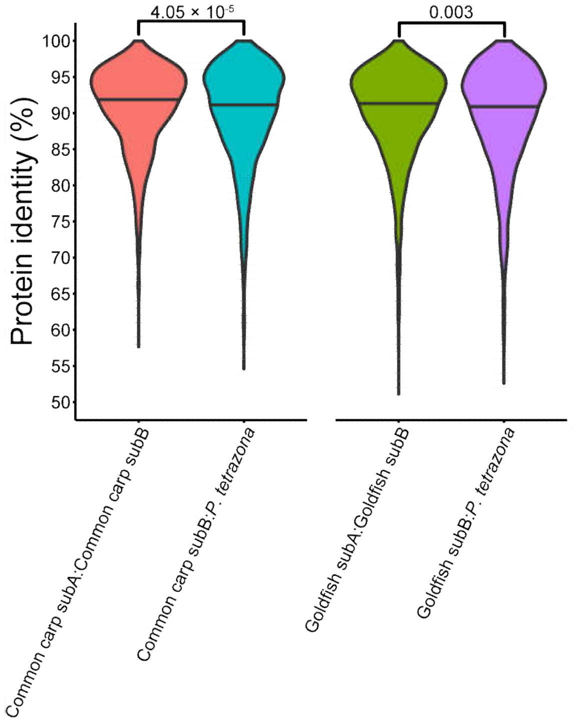

(a) The mRNA identities between the subB genes and their *P. tetrazona* orthologues are significantly higher than those between the homoeologues in each tetraploid fish (two-sided Mann Whitney *U* test). (b) The protein identities between the subB genes and their *P. tetrazona* orthologues are significantly lower than those between the homoeologues in each tetraploid fish (two-sided Mann Whitney *U* test).

Supplementary Fig. 24. Expansions of DNA transposons and retrotransposons in Cyprinidae fish

(a)

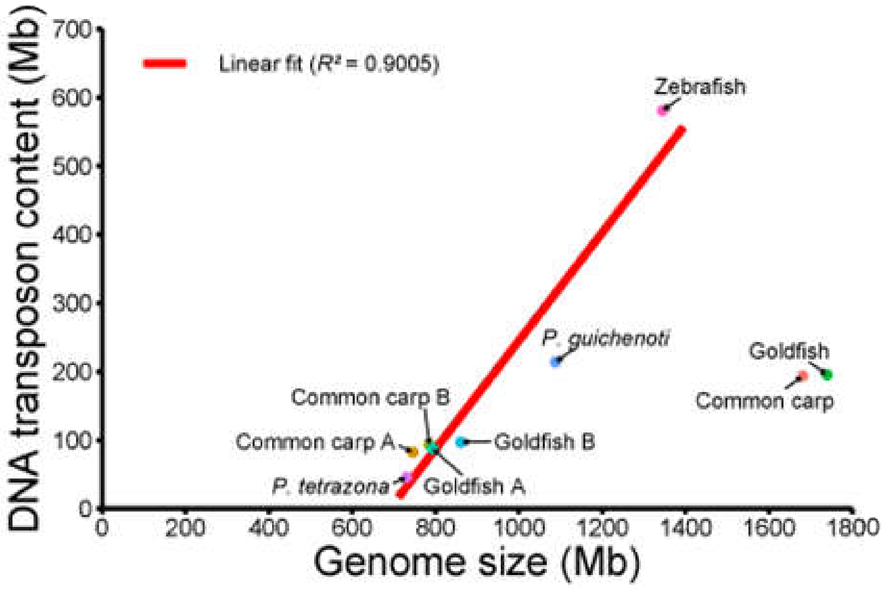

(b)

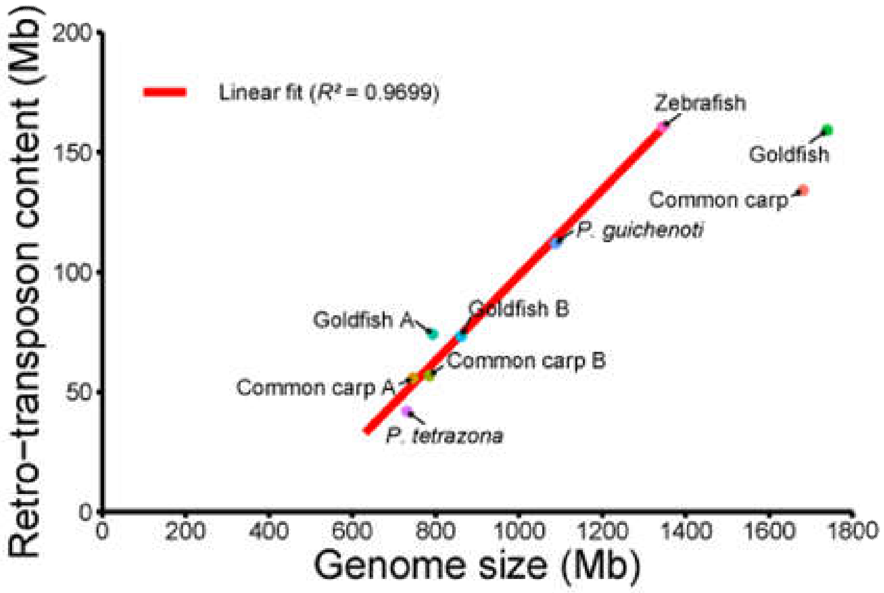

(a) The significant correlation of DNA transposon content and the genome size. (b) The significant correlation of retrotransposon content and the genome size.

Supplementary Fig. 25. Distributions of the subgenome-specific transposons on each tetraploid chromosome

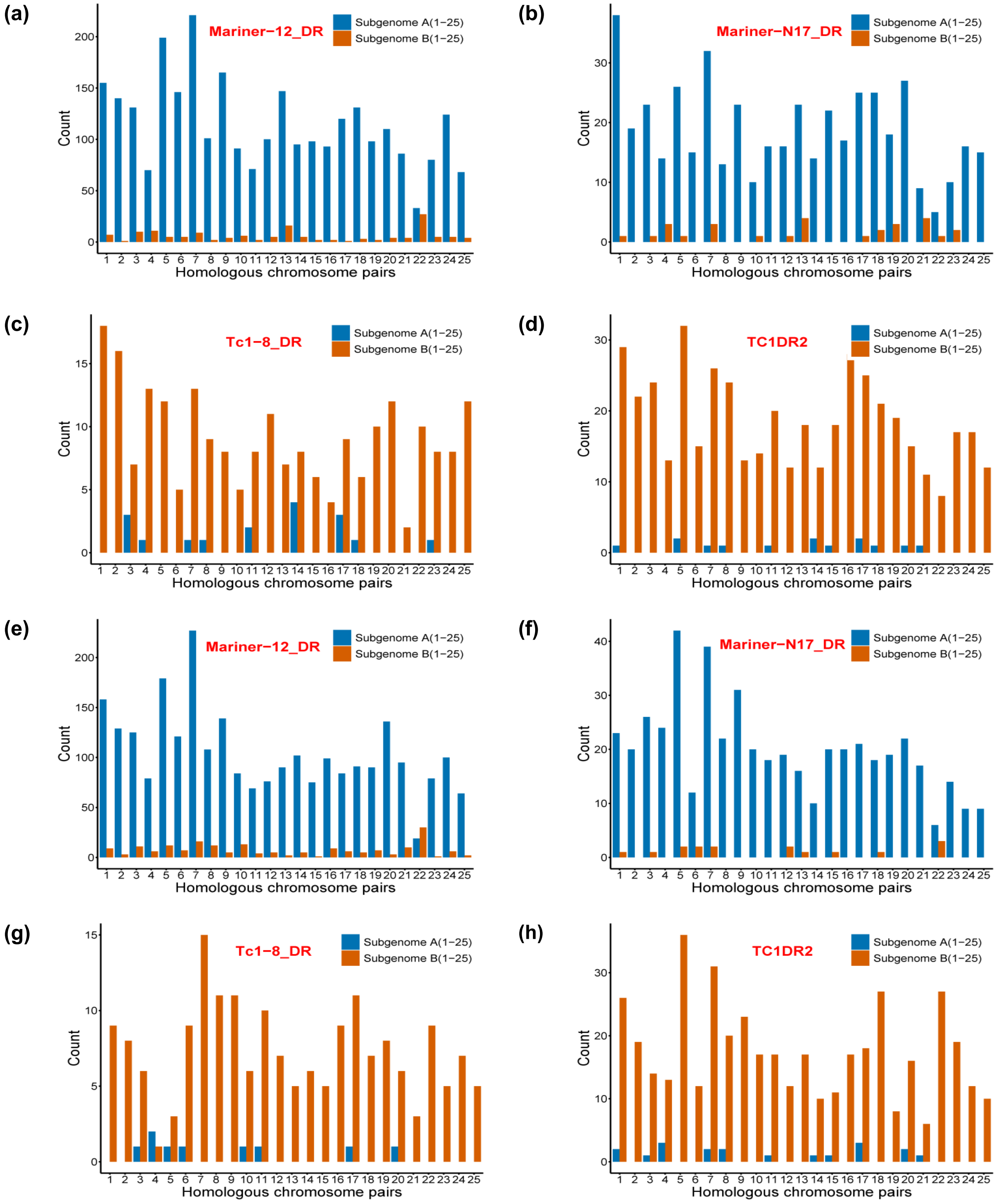

The figures (a)(b)(c)(d) show the distributions of Mariner-12_DR, Mariner-N17_DR, Tc1-8_DR, and TC1DR2 in the common carp subgenomes. The figures (e)(f)(g)(h) show the distributions of these four type of TEs in the goldfish subgenomes respectively.

Supplementary Fig. 26. Repeat divergence distributions in seven genomes

(a)

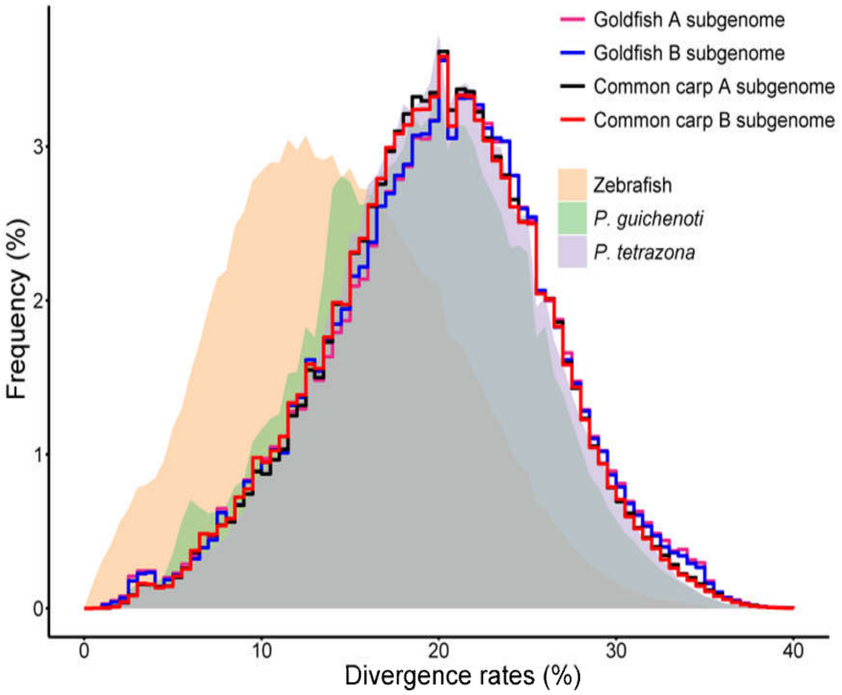

(b)

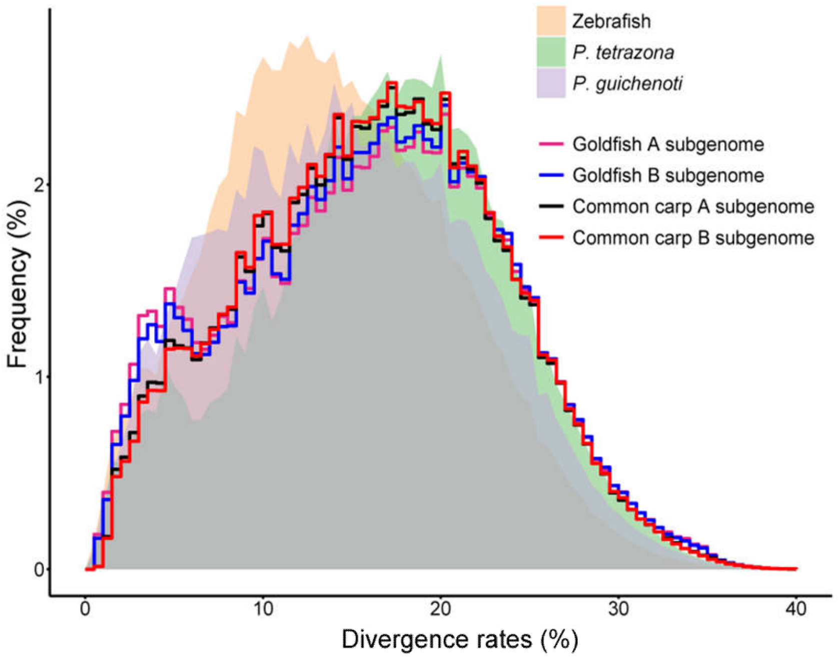

The sequence divergence distributions of transposons (a) and all repeats (b) in the genomes of common carp A, common carp B, goldfish A, goldfish B, *P. guichenoti*, *P. tetrazona*, and zebrafish. The sequence divergence distributions of *P. guichenoti*, *P. tetrazona*, and zebrafish are represented by different color shades. Four color curves mean the divergence distributions on the subgenomes of common carp and goldfish.

Supplementary Fig. 27. Abundances and pairwise similarities of TcMar-Tc1 DNA transposons in the common carp genome

(a)

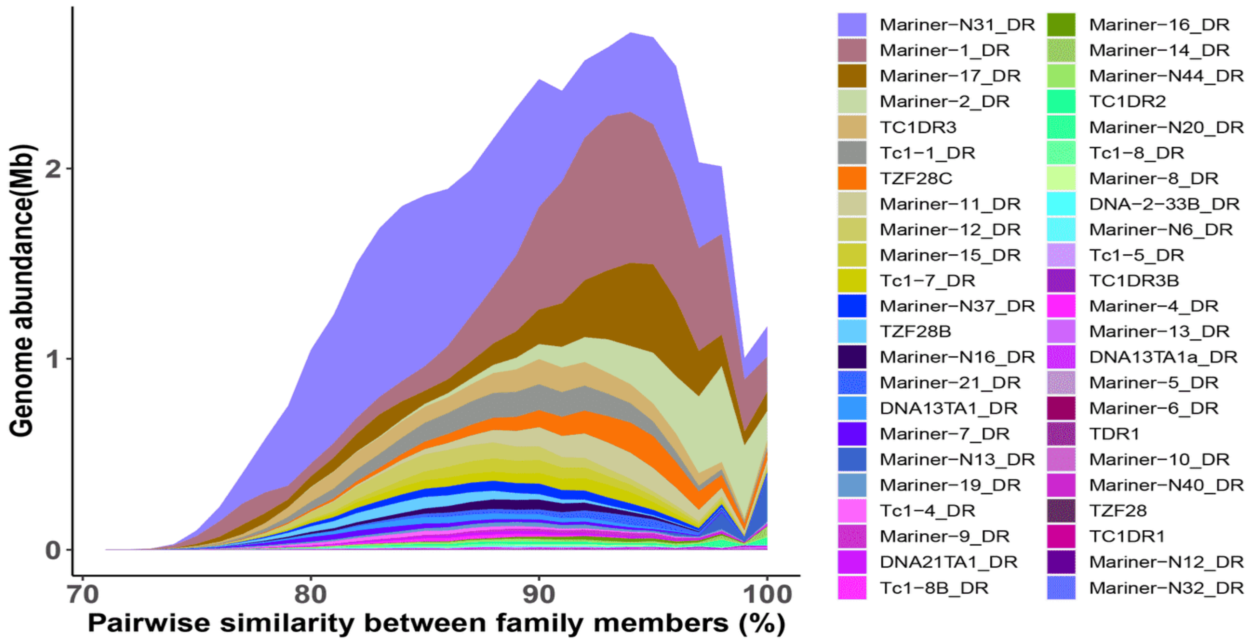

(b)

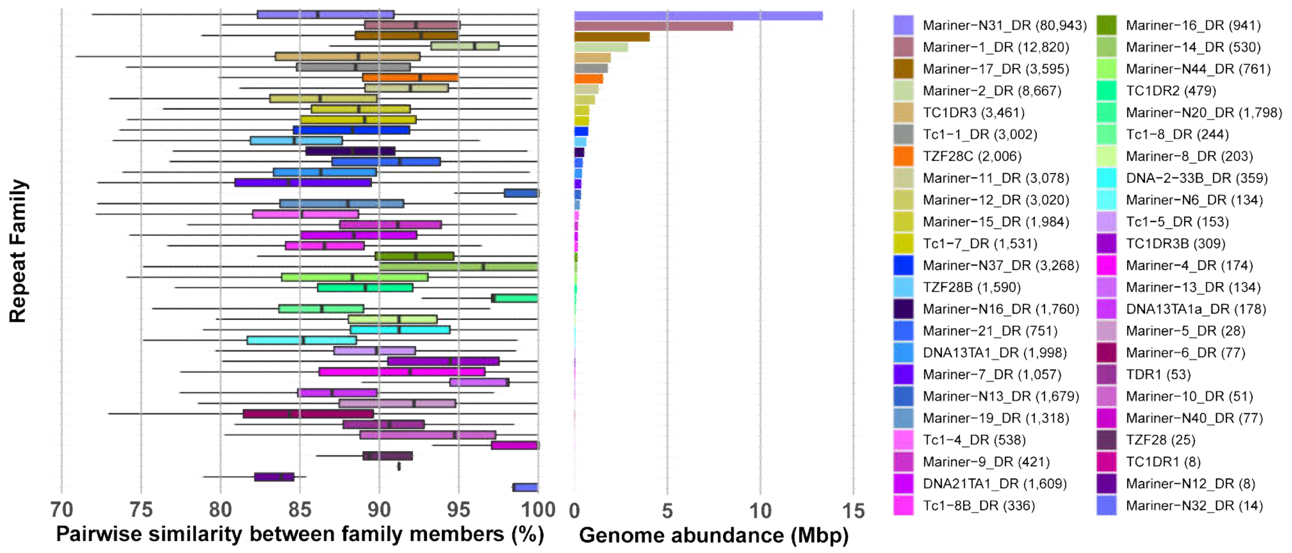

(a) In the common carp genome, an expansion of TcMar-Tc1 transposons was ongoing with a major peak at an average of 96% similarity between family members. (b) The boxplots and histograms display the pairwise similarity and abundance of each TcMar-Tc1 family. The boxplots show the 25th, 50th and 75th percentiles. The left and right whiskers correspond to the first quartile - 1.5 × IQR and the third quartile + 1.5 × IQR, respectively. The number of each compared TcMar-Tc1 transposon is indicated in the bracket.

Supplementary Fig. 28. Abundances and pairwise similarities of TcMar-Tc1 DNA transposons in the goldfish genome

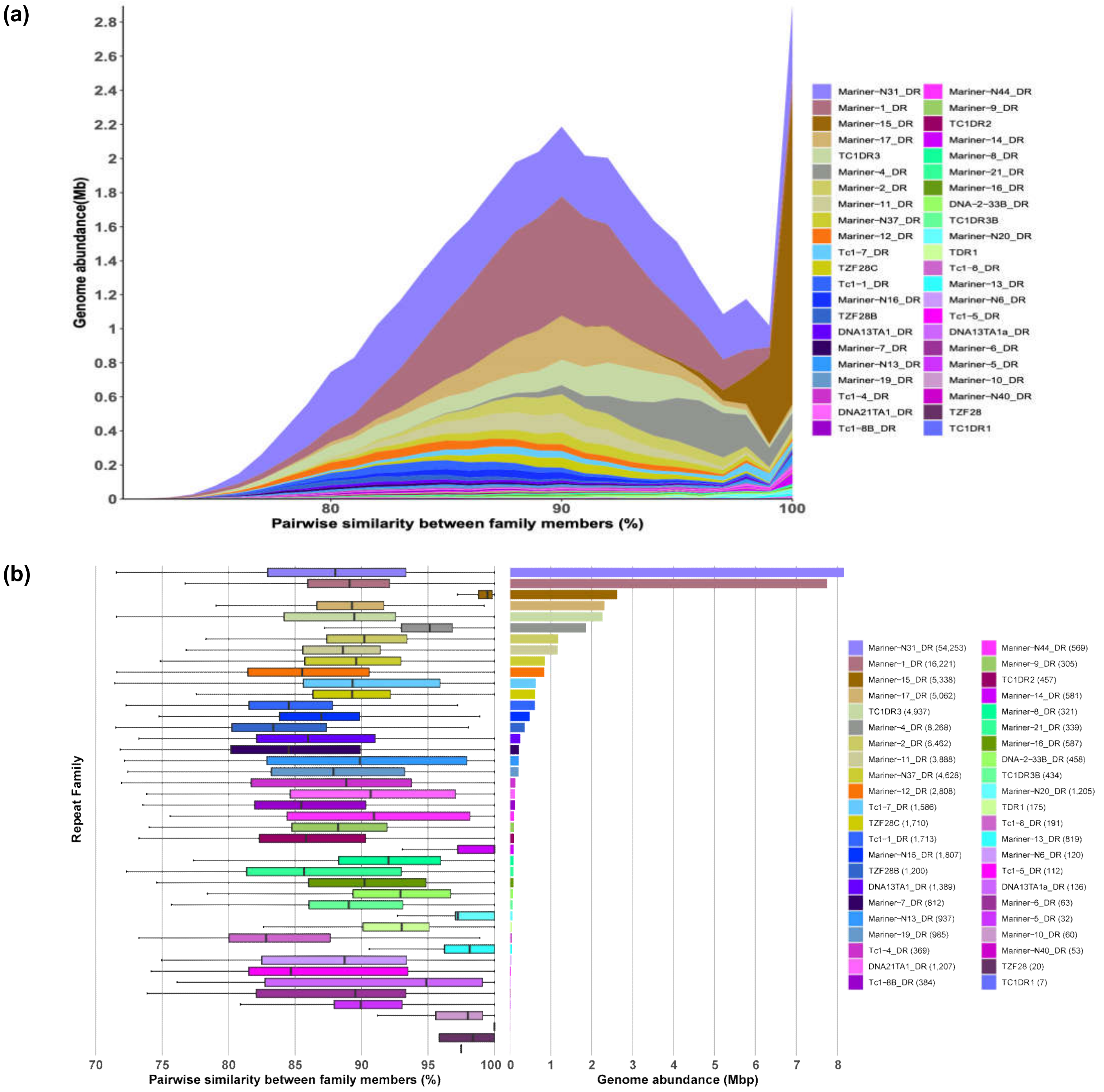

The figure (a) shows an expansion of the TcMar-Tc1 transposons in the goldfish genome. The boxplots and histograms (b) display the pairwise similarity and abundance of each TcMar-Tc1 family. The definitions of the boxplots, whiskers, and numbers in the brackets (b) are consistent with those in Supplementary Fig. 27 (b).

Supplementary Fig. 29. Abundances and pairwise similarities of TcMar-Tc1 DNA transposons in the *P. guichenoti* genome

(a)

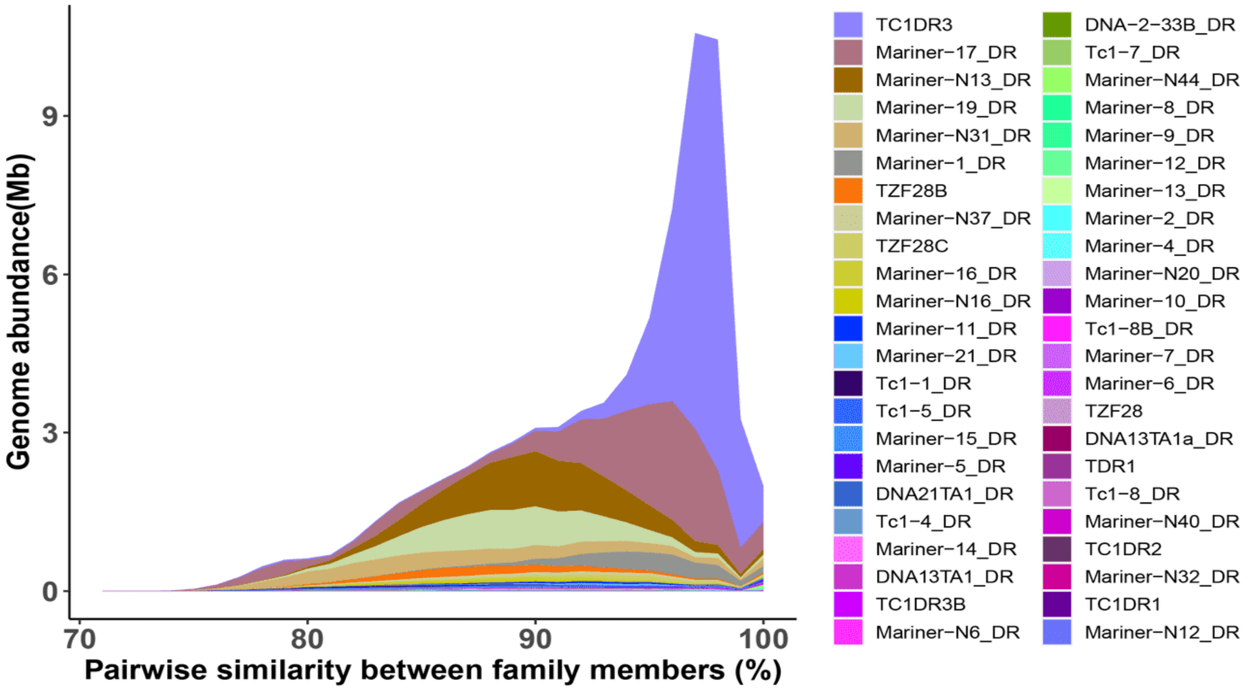

(b)

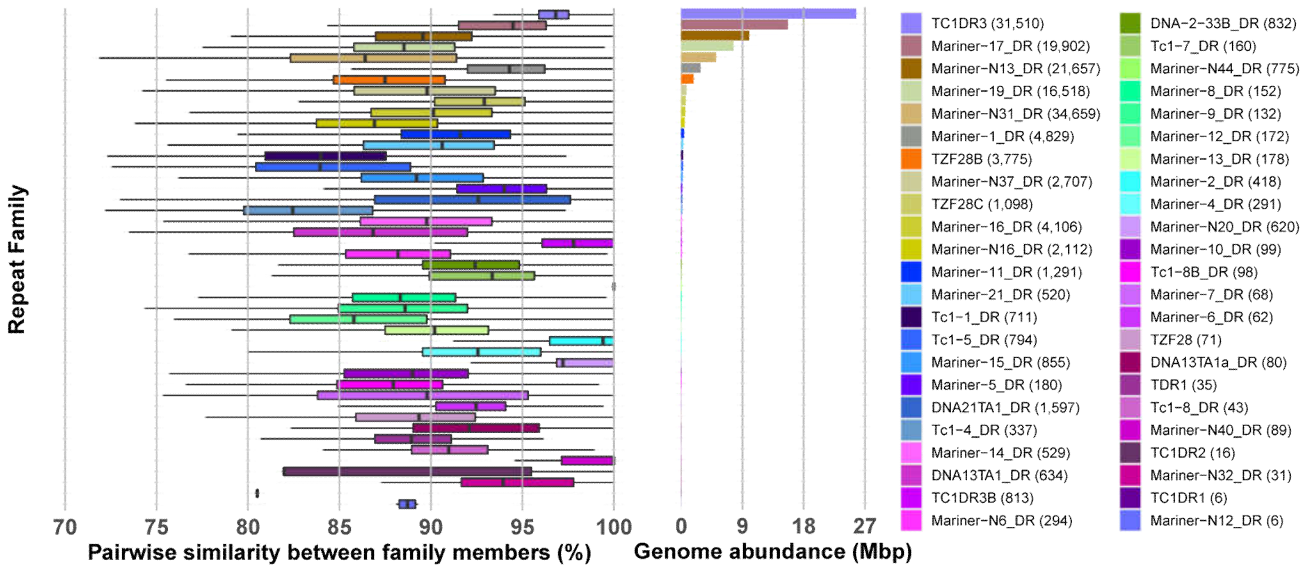

The figure (a) shows an expansion of the TcMar-Tc1 superfamily in the *P. guichenoti* genome. The boxplots and histograms in (b) display the pairwise similarity and abundance of each TcMar-Tc1 family. The definitions of the boxplots, whiskers, and numbers in the brackets (b) are consistent with those in Supplementary Fig. 27 (b).

Supplementary Fig. 30. Abundances and pairwise similarities of TcMar-Tc1 DNA transposons in the *P. tetrazona* genome

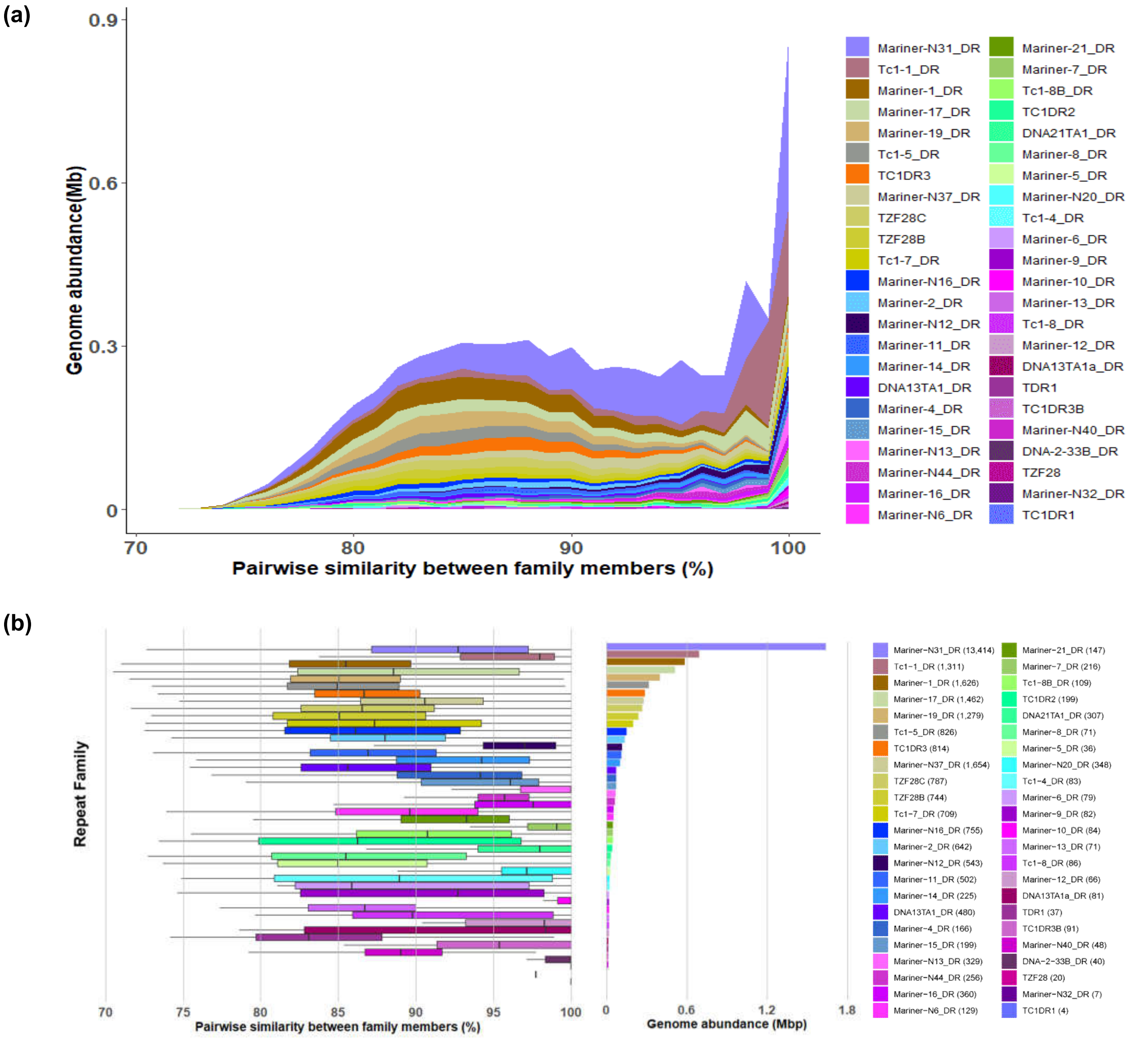

The figure (a) shows an expansion of the TcMar-Tc1 superfamily in the *P. tetrazona* genome. The boxplots and histograms (b) display the pairwise similarity and abundance of each TcMar-Tc1 family. The definitions of the boxplots, whiskers, and numbers in the brackets (b) are consistent with those in Supplementary Fig. 27 (b).

Supplementary Fig. 31. Abundances and pairwise similarities of TcMar-Tc1 DNA transposons in the common carp A and B subgenomes

(a)

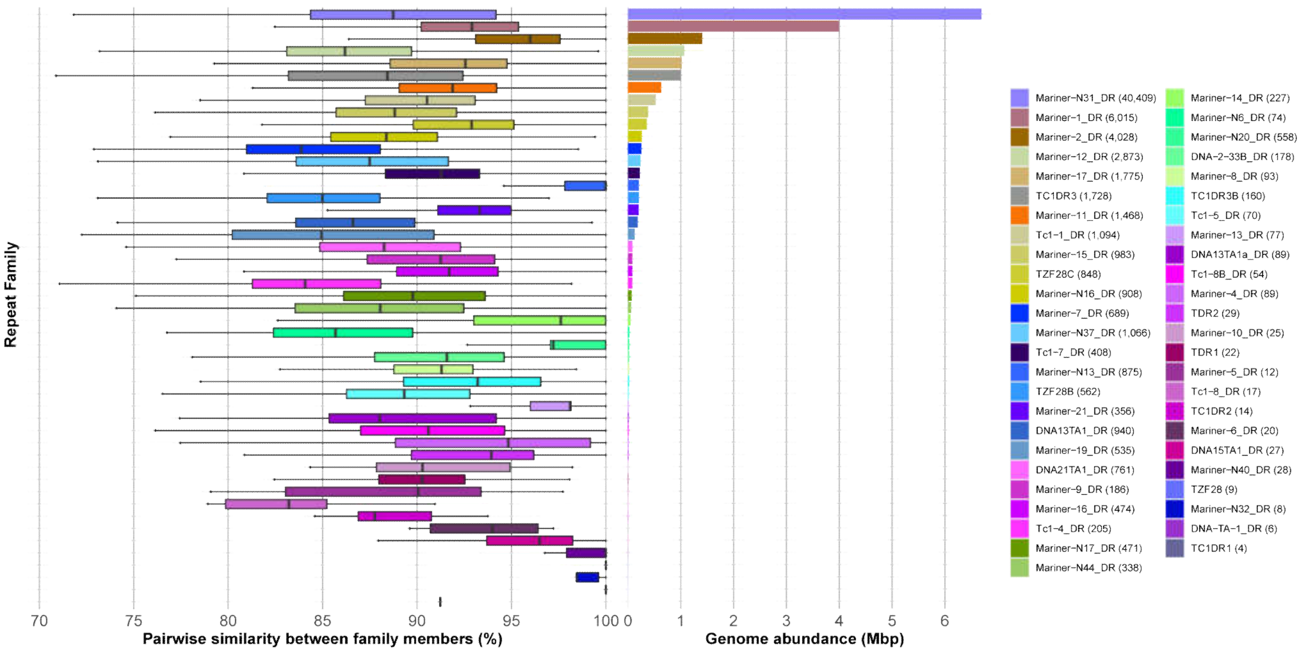

(b)

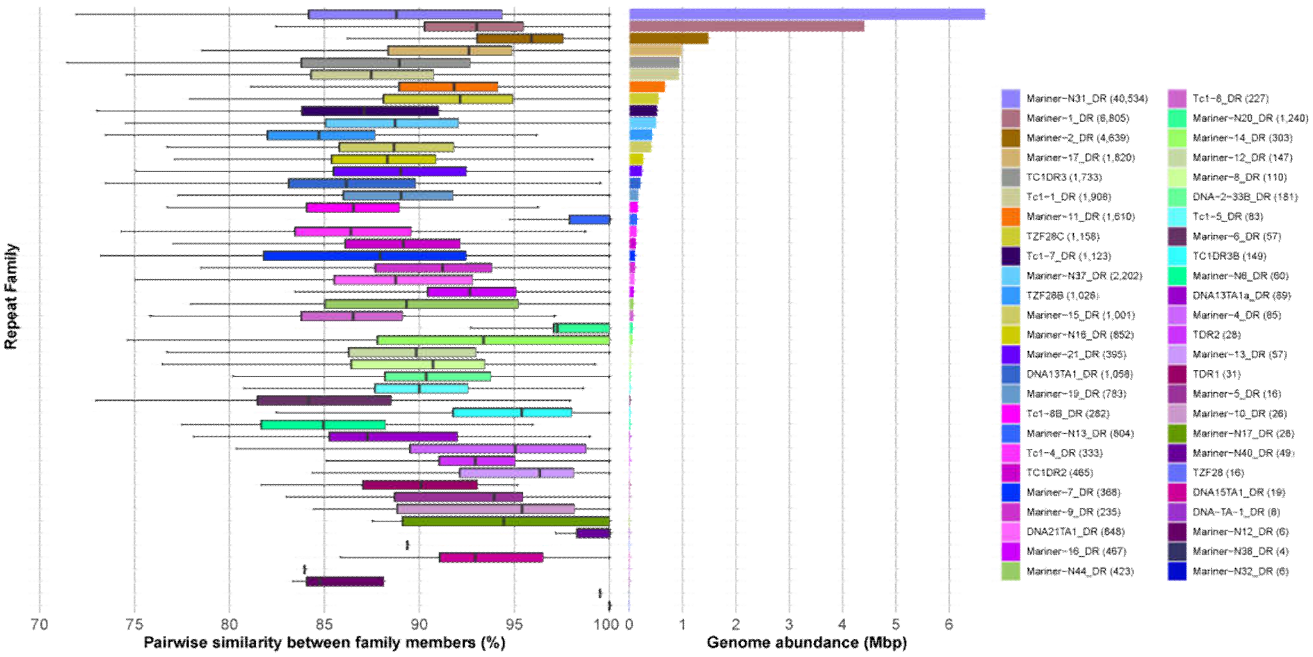

The boxplots and histograms displayed the pairwise similarity and abundance of each TcMar-Tc1 family in the common carp A subgenome (a) and B subgenome (b). The definitions of the boxplots, whiskers, and numbers in the brackets are consistent with those in Supplementary Fig. 27 (b).

Supplementary Fig. 32. Abundances and pairwise similarities of TcMar-Tc1 DNA transposons in the goldfish A and B subgenomes

(a)

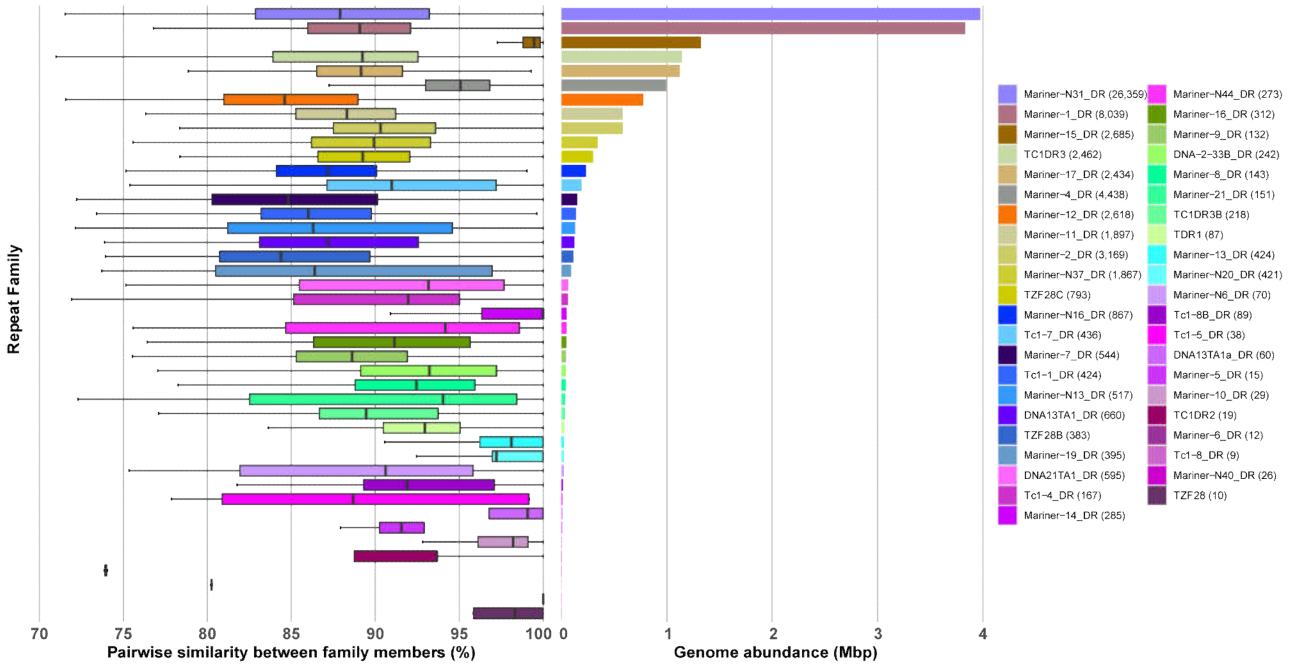

(b)

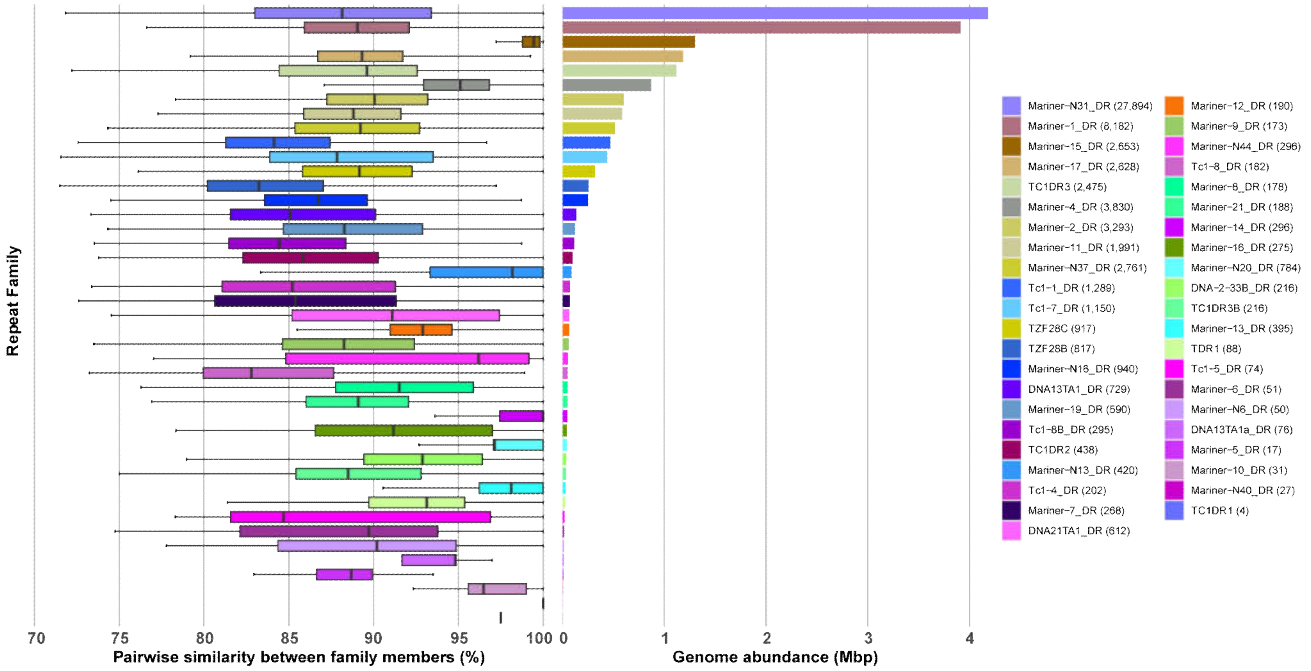

The boxplots and histograms display the pairwise similarity and abundance of each TcMar-Tc1 family in the goldfish A subgenome (a) and B subgenome (b). The definitions of the boxplots, whiskers, and numbers in the brackets are consistent with those in Supplementary Fig. 27 (b).

Supplementary Fig. 33. Abundances and pairwise similarities of L2 retrotransposons in the common carp genome

(a)

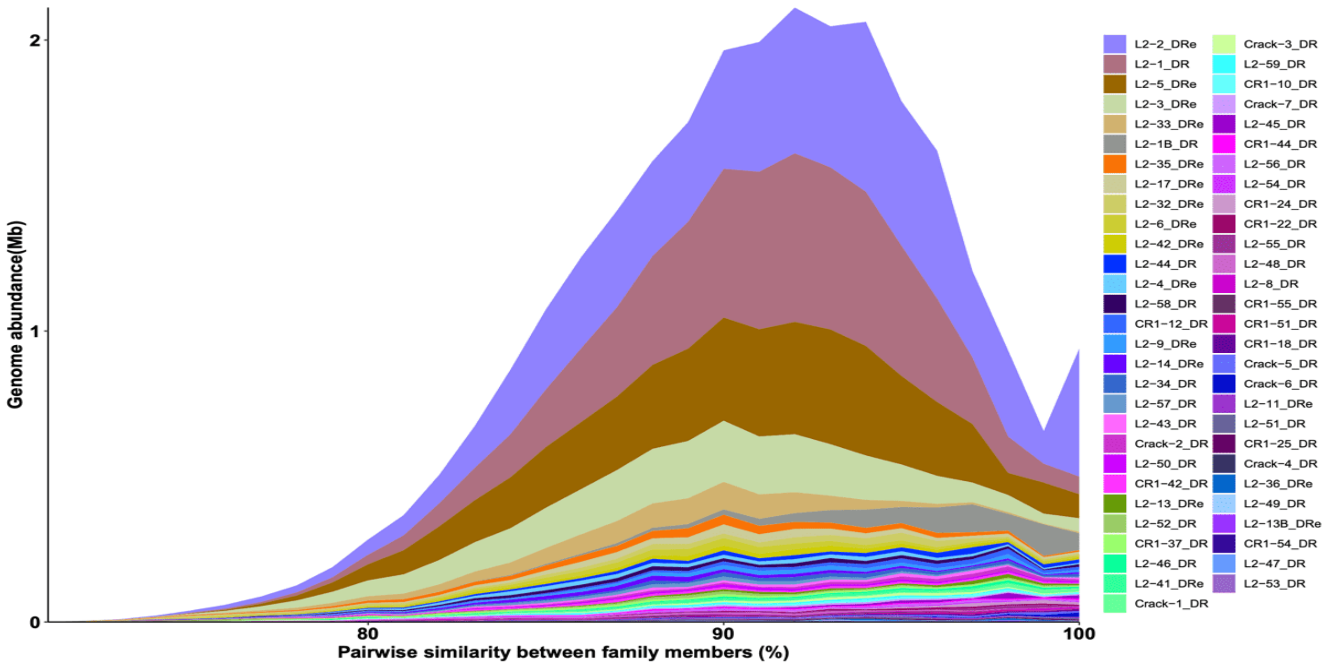

(b)

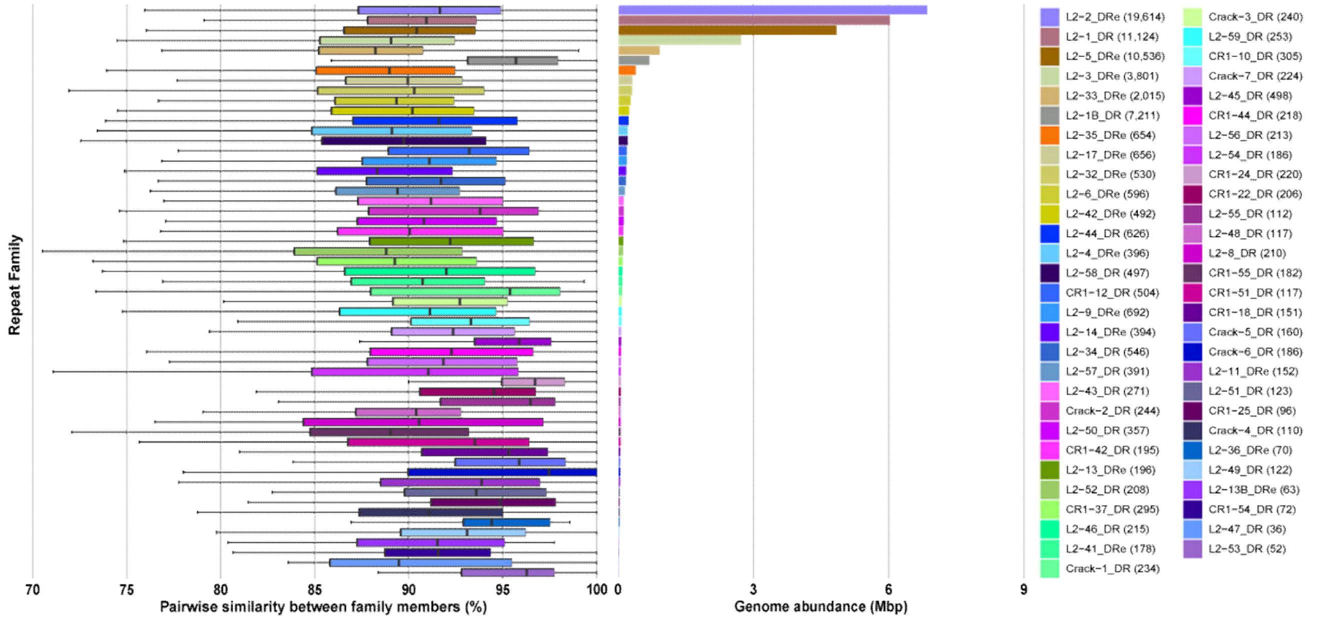

In the common carp genome, an expansion of L2 retrotransposons was ongoing with high similarity (a). The boxplots and histograms display the pairwise similarity and abundance of each L2 family (b). The definitions of the boxplots, whiskers, and numbers in the brackets are consistent with those in Supplementary Fig. 27 (b).

Supplementary Fig. 34. Abundances and pairwise similarities of L2 retrotransposons in the goldfish genome

(a)

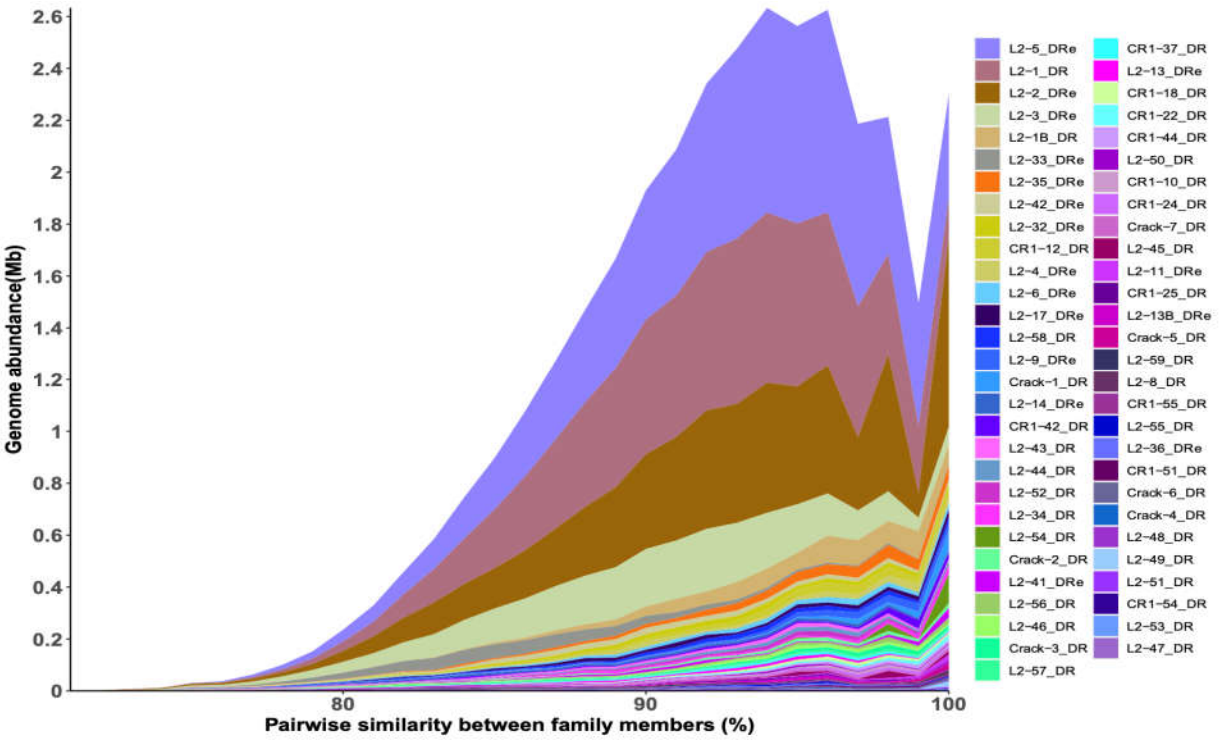

(b)

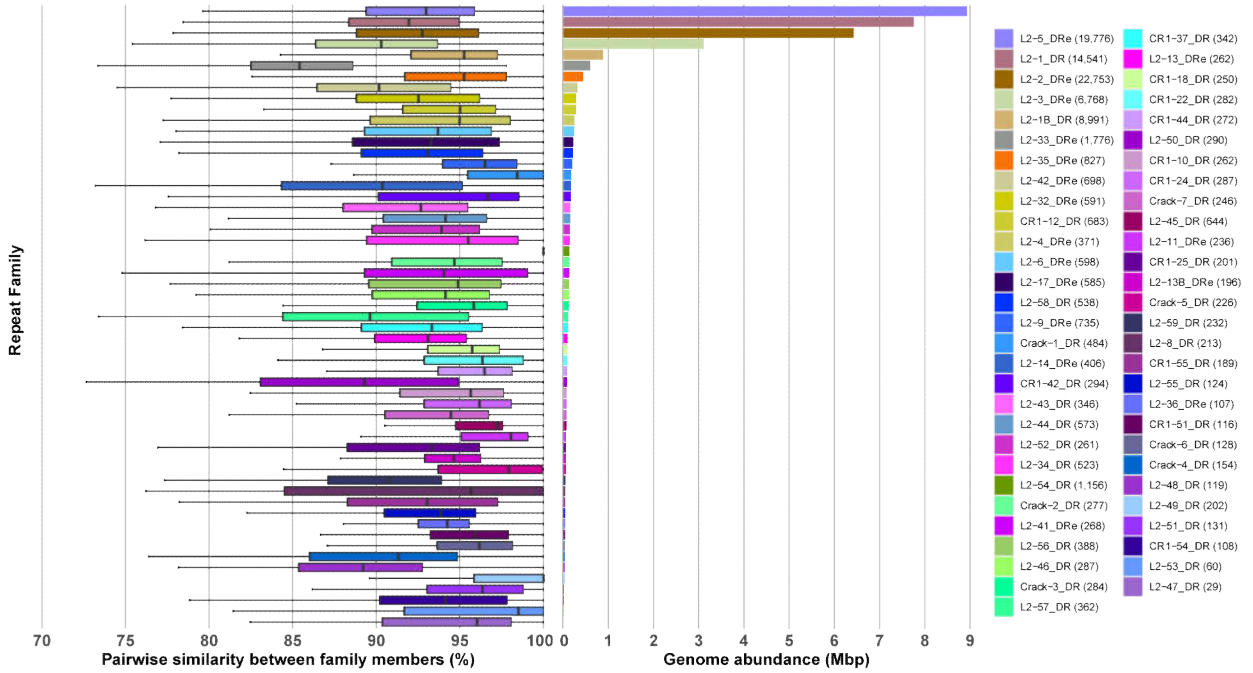

In the goldfish genome, an expansion of L2 retrotransposons was ongoing with high similarity (a). The boxplots and histograms display the pairwise similarity and abundance of each L2 family (b). The definitions of the boxplots, whiskers, and numbers in the brackets (b) are consistent with those in Supplementary Fig. 27 (b).

Supplementary Fig. 35. Abundances and pairwise similarities of L2 retrotransposons in the *P. guichenoti* genome

(a)

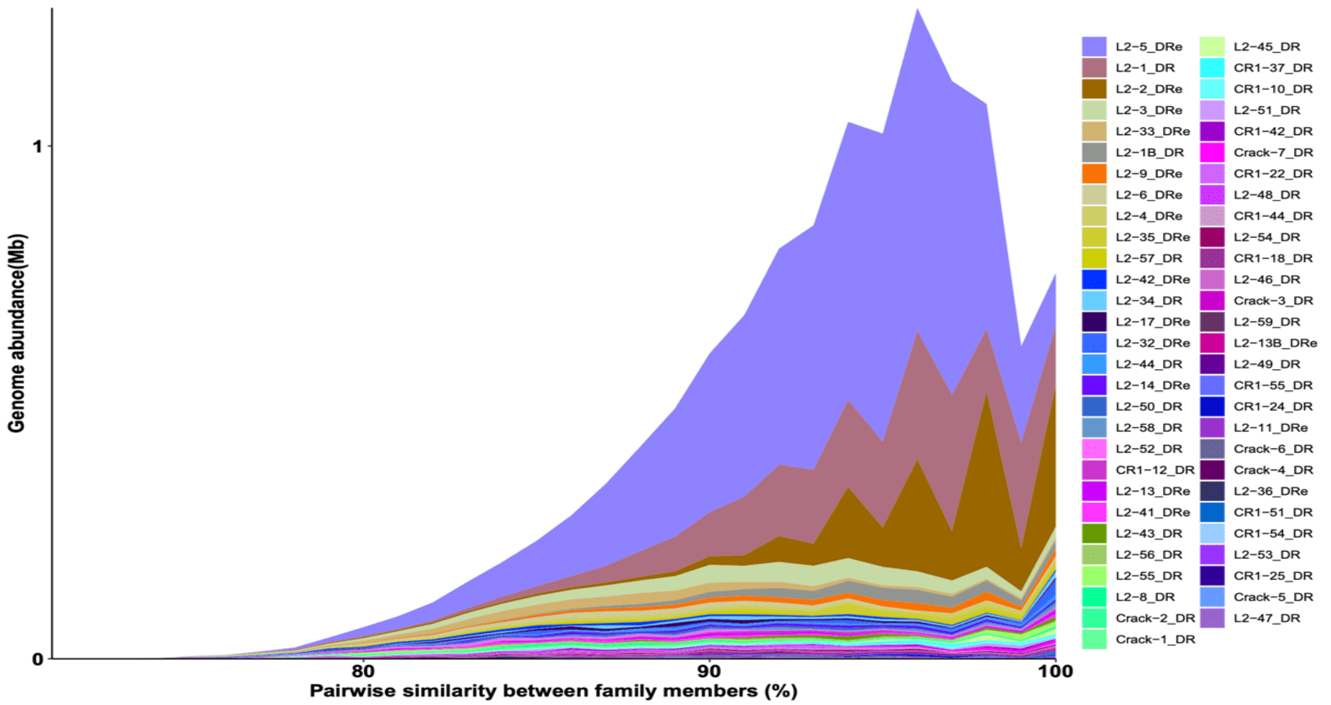

(b)

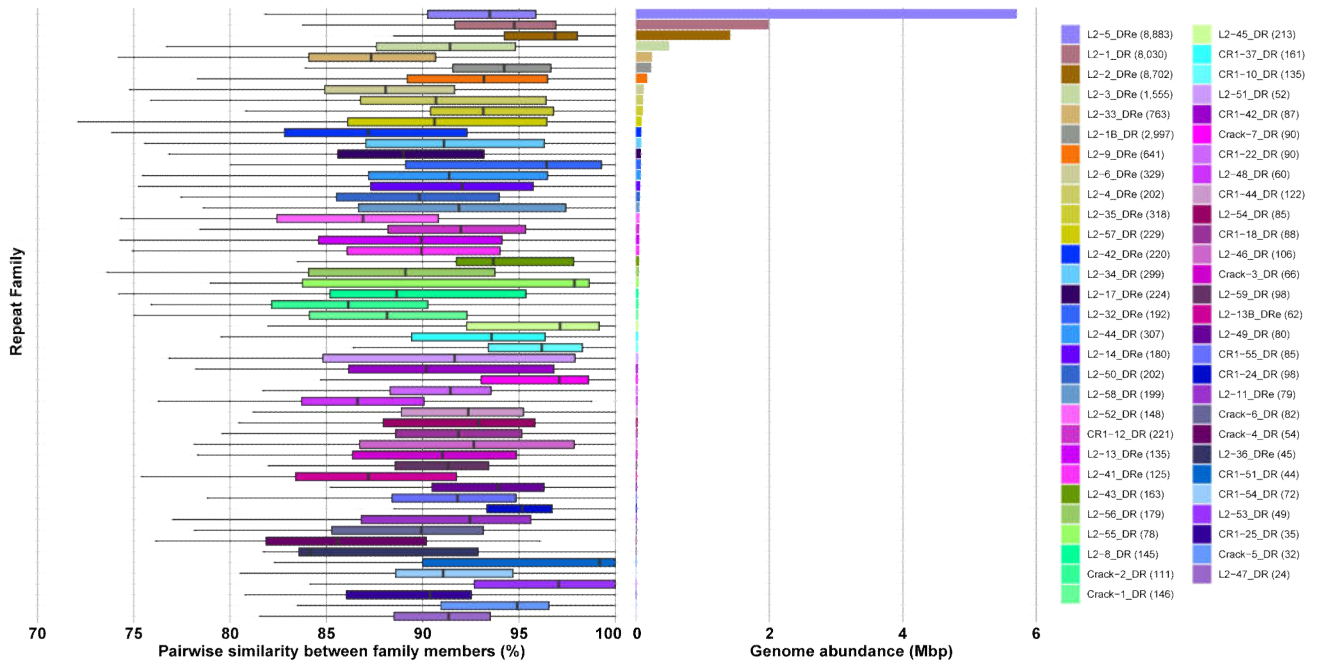

The figure (a) shows the expansion of L2 retrotransposons in *P. guichenoti g*enome and the boxplots and histograms in figure (b) display the pairwise similarity and abundance of each L2 family. The definitions of the boxplots, whiskers, and numbers in the brackets are consistent with those in Supplementary Fig. 27 (b).

Supplementary Fig. 36. Abundances and pairwise similarities of L2 retrotransposons in the *P. tetrazona* genome

(a)

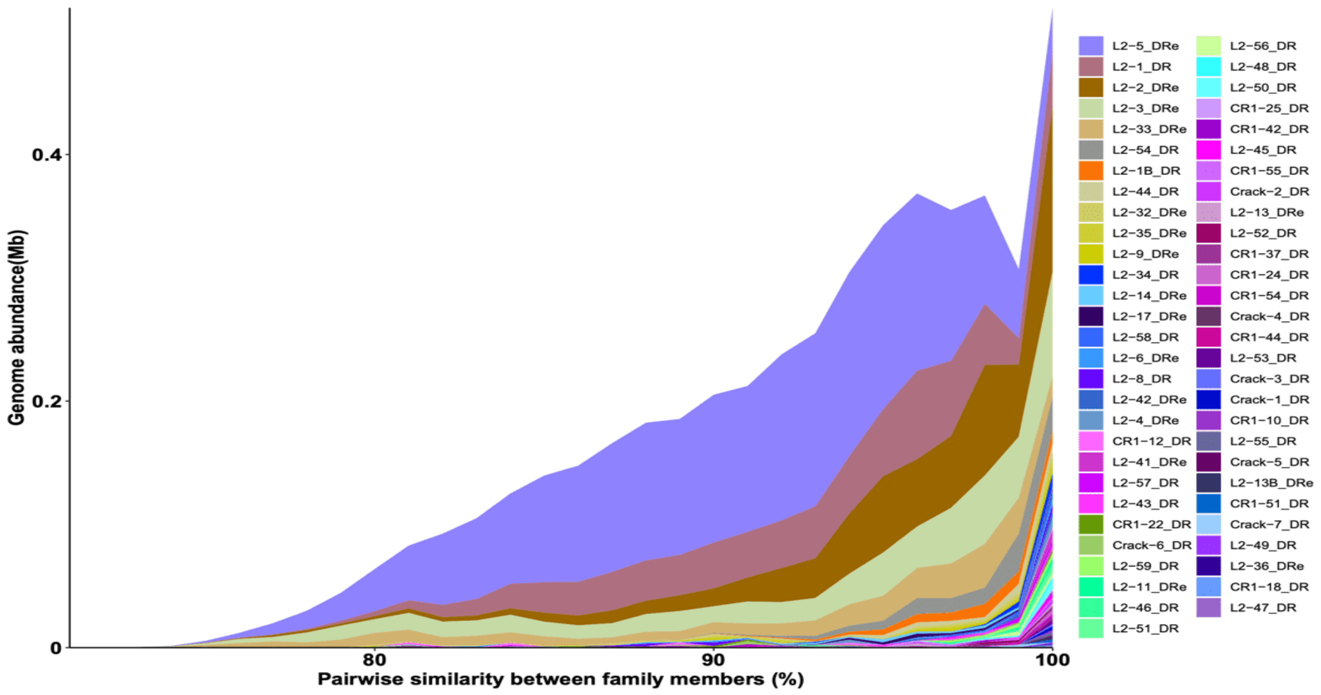

(b)

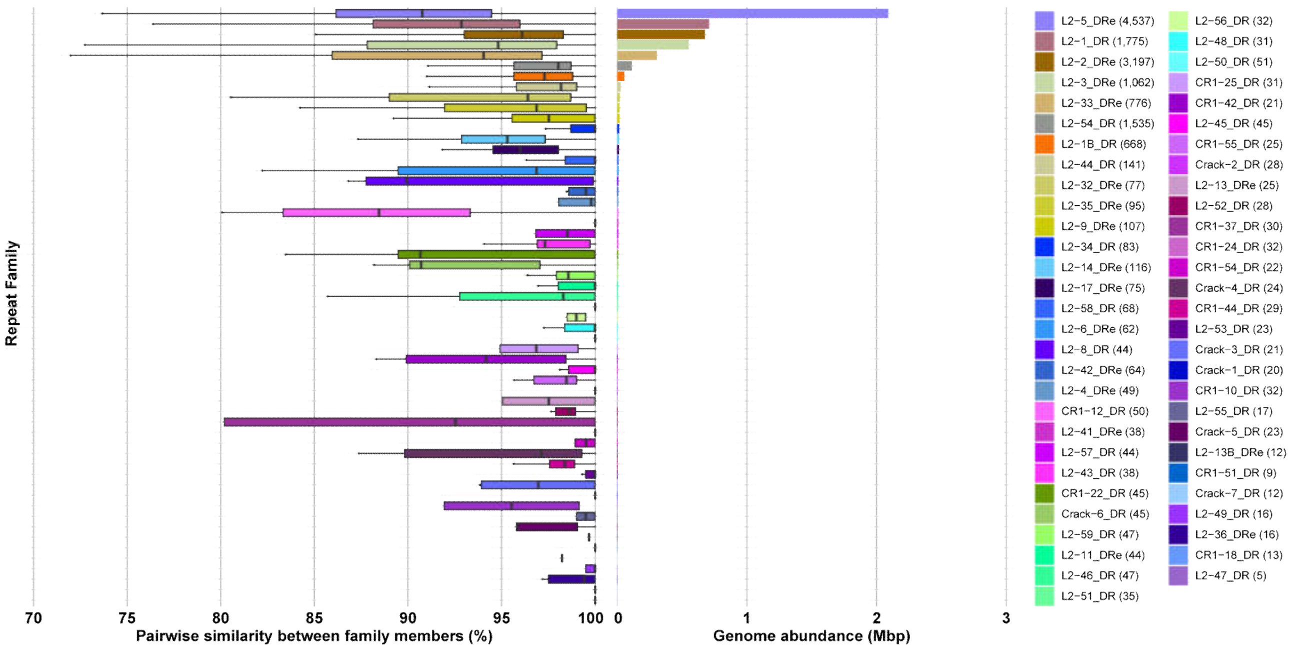

The figure (a) shows the expansion of L2 retrotransposons in *P. tetrazona* genome and the boxplots and histograms in figure (b) display the pairwise similarity and abundance of each L2 family. The definitions of the boxplots, whiskers, and numbers in the brackets are consistent with those in Supplementary Fig. 27 (b).

Supplementary Fig. 37. Abundances and pairwise similarities of L2 retrotransposons in the common carp A subgenome

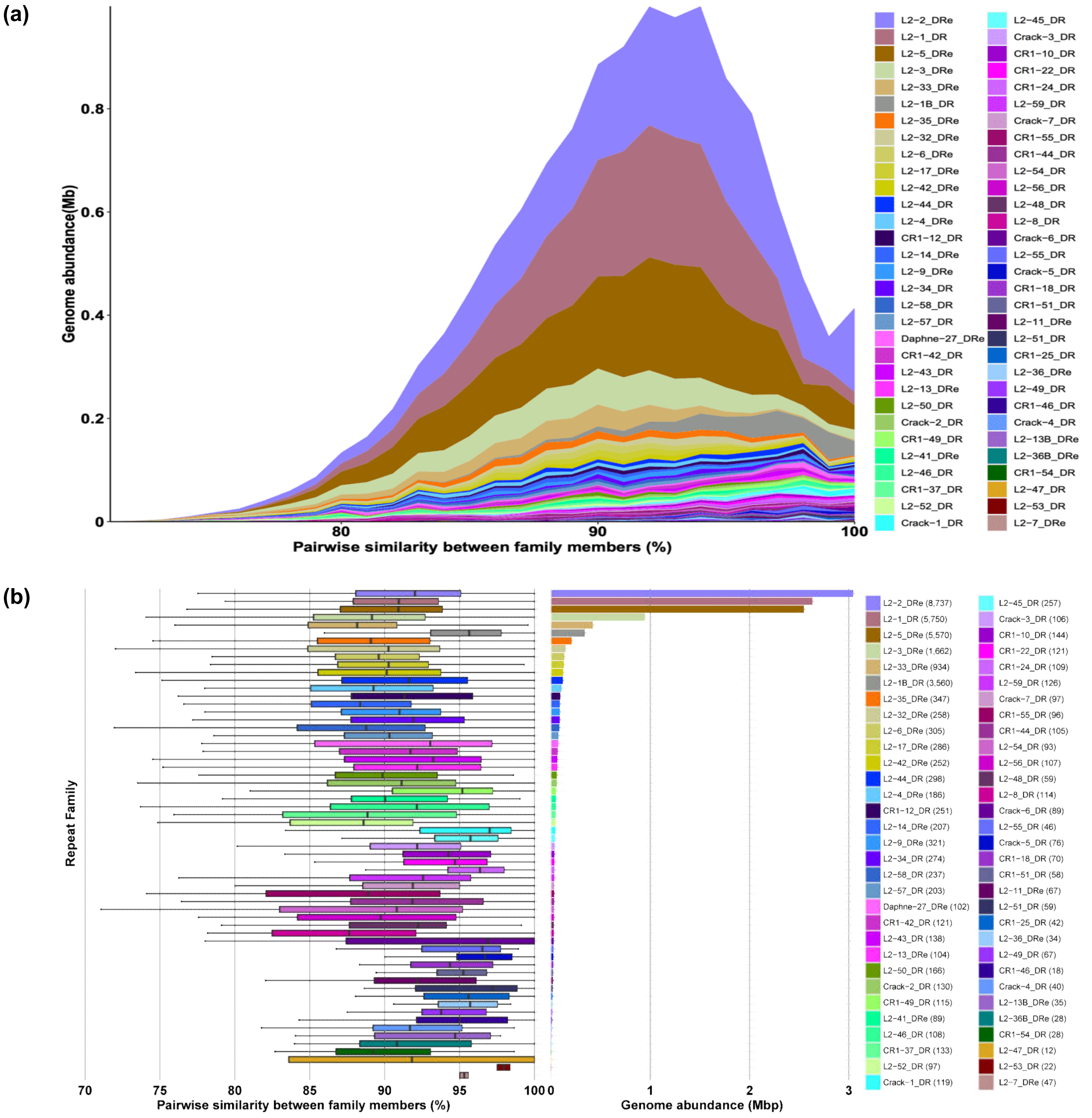

The figure (a) shows the expansion of L2 retrotransposons in the common carp A subgenome and the boxplots and histograms in figure (b) display the pairwise similarity and abundance of each L2 family. The definitions of the boxplots, whiskers, and numbers in the brackets are consistent with those in Supplementary Fig. 27 (b).

Supplementary Fig. 38. Abundances and pairwise similarities of L2 retrotransposons in the common carp B subgenome

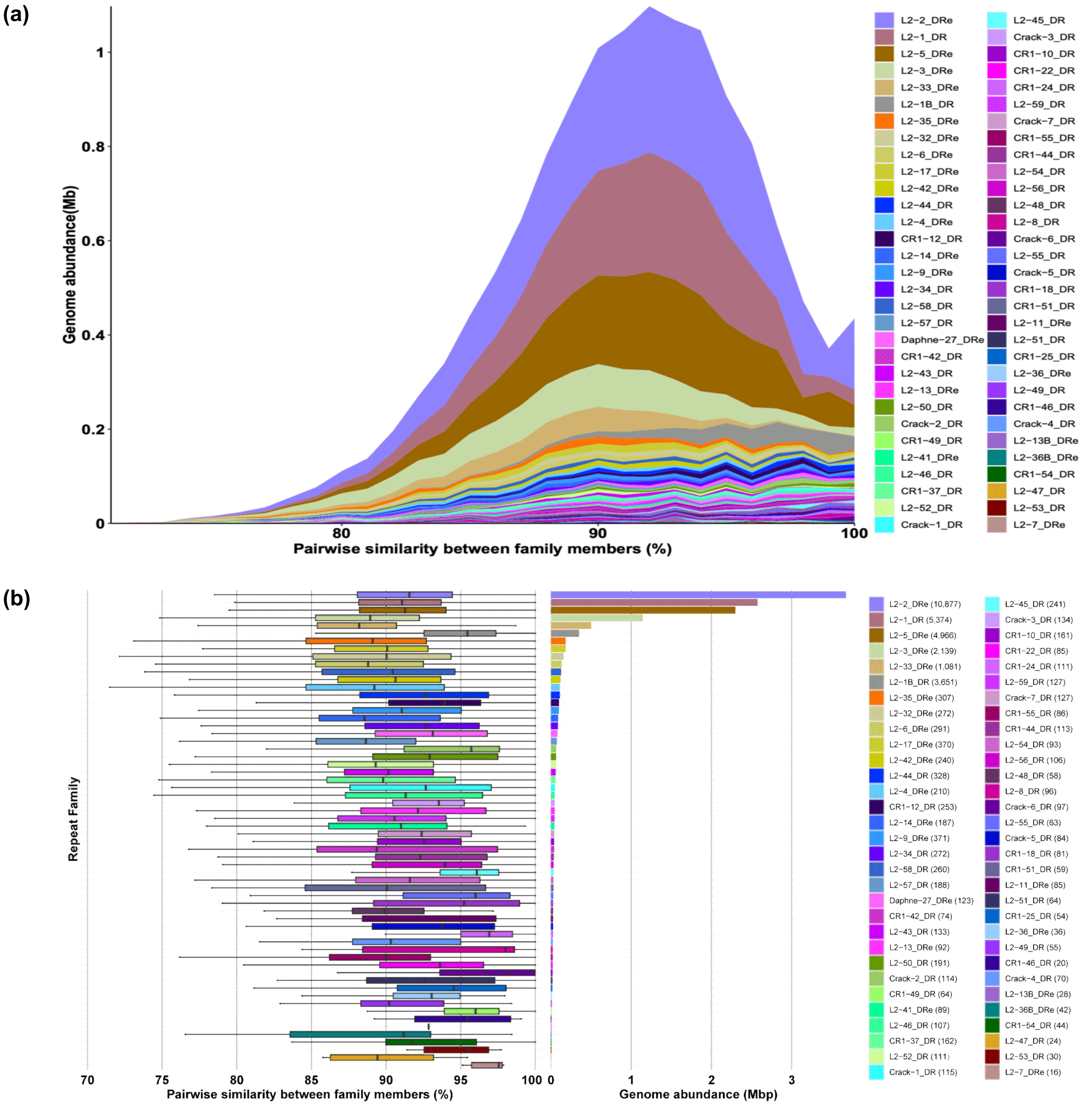

The figure (a) shows the expansion of L2 retrotransposons in the common carp B subgenome and the boxplots and histograms in figure (b) display the pairwise similarity and abundance of each L2 family. The definitions of the boxplots, whiskers, and numbers in the brackets are consistent with those in Supplementary Fig. 27 (b).

Supplementary Fig. 39. Abundances and pairwise similarities of L2 retrotransposons in the goldfish A subgenome

(a)

(b)

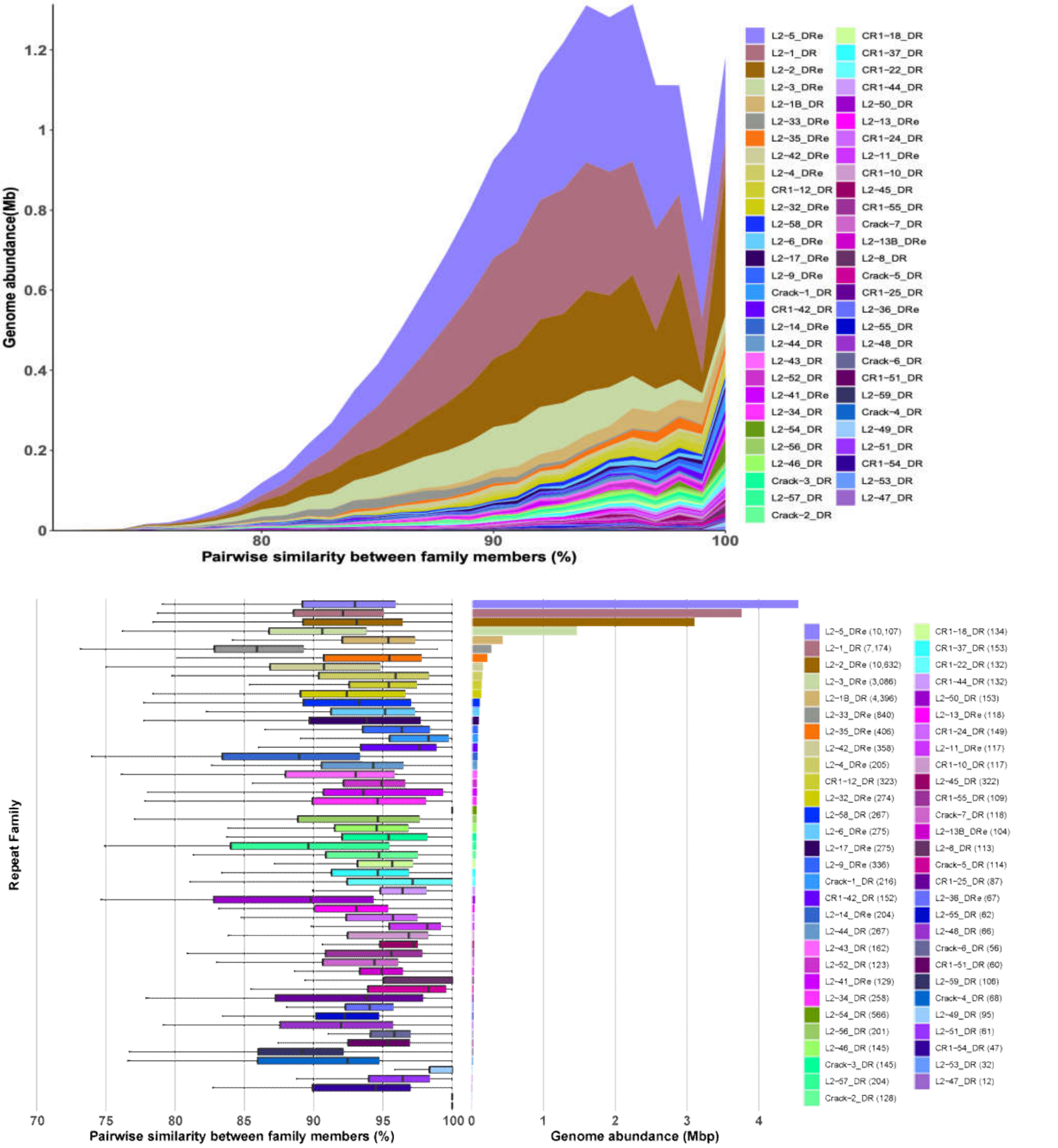

The figure (a) shows the expansion of L2 retrotransposons in the goldfish A subgenome and the boxplots and histograms in figure (b) display the pairwise similarity and abundance of each L2 family. The definitions of the boxplots, whiskers, and numbers in the brackets are consistent with those in Supplementary Fig. 27 (b).

Supplementary Fig. 40. Abundances and pairwise similarities of L2 retrotransposons in the goldfish B subgenome

(a)

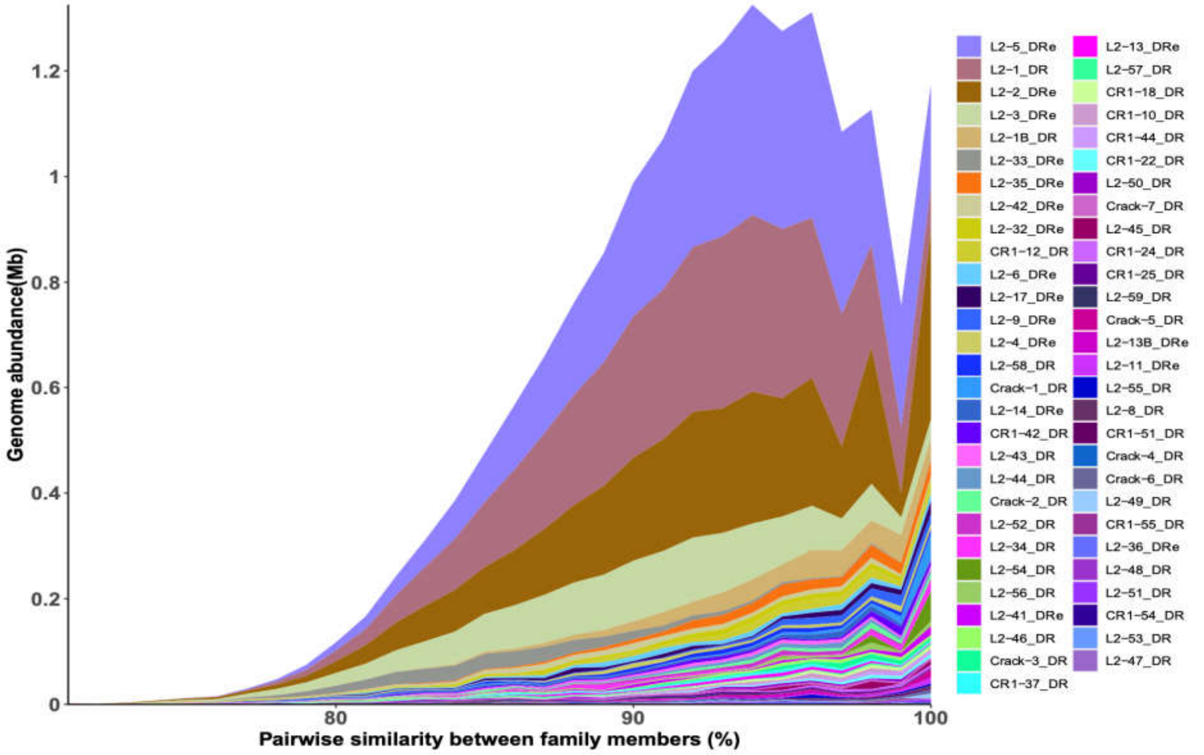

(b)

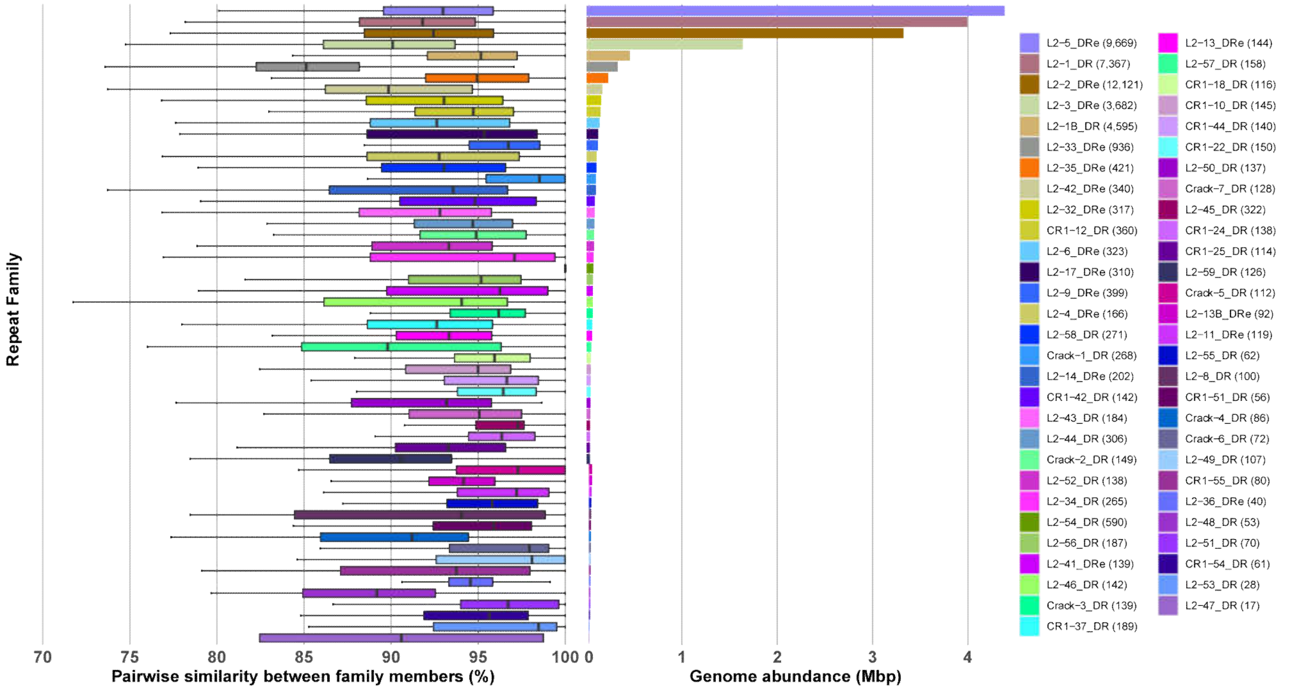

The figure (a) shows the expansion of L2 retrotransposons in the goldfish B subgenome and the boxplots and histograms in figure (b) display the pairwise similarity and abundance of each L2 family. The definitions of the boxplots, whiskers, and numbers in the brackets are consistent with those in Supplementary Fig. 27 (b).

Supplementary Fig. 41. Schematic figure for the homoeologous exchange

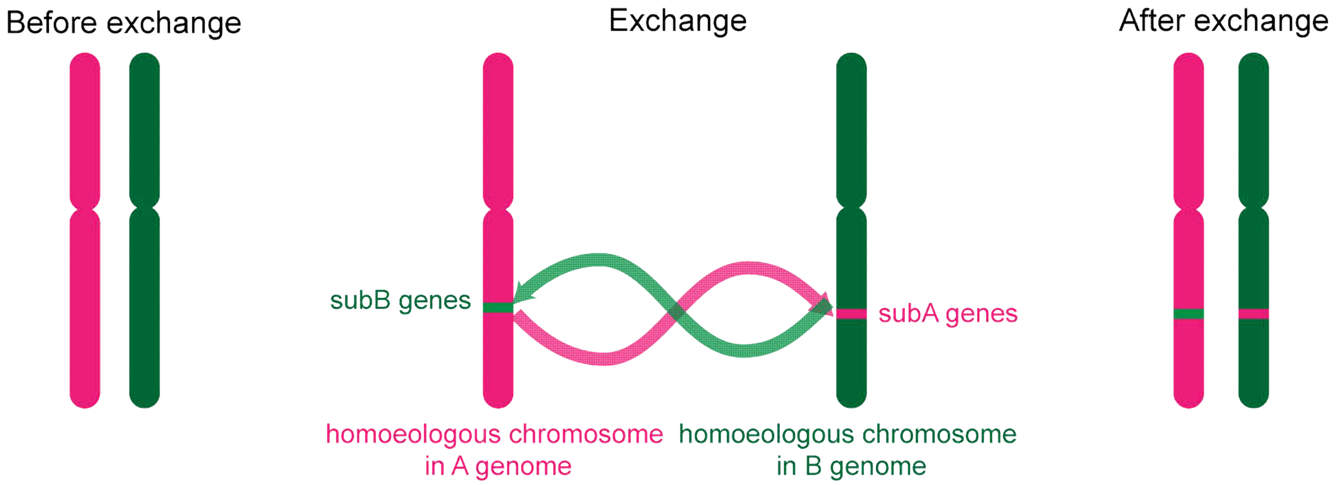

The pink bar and green bar represent the A chromosome and homoeologous B chromosome, respectively. The pink and green pieces stand for the exchanged subA and subB genes, respectively.

Supplementary Fig. 42. In both common carp and goldfish, homoeologue exchanges in the A and B subgenomes

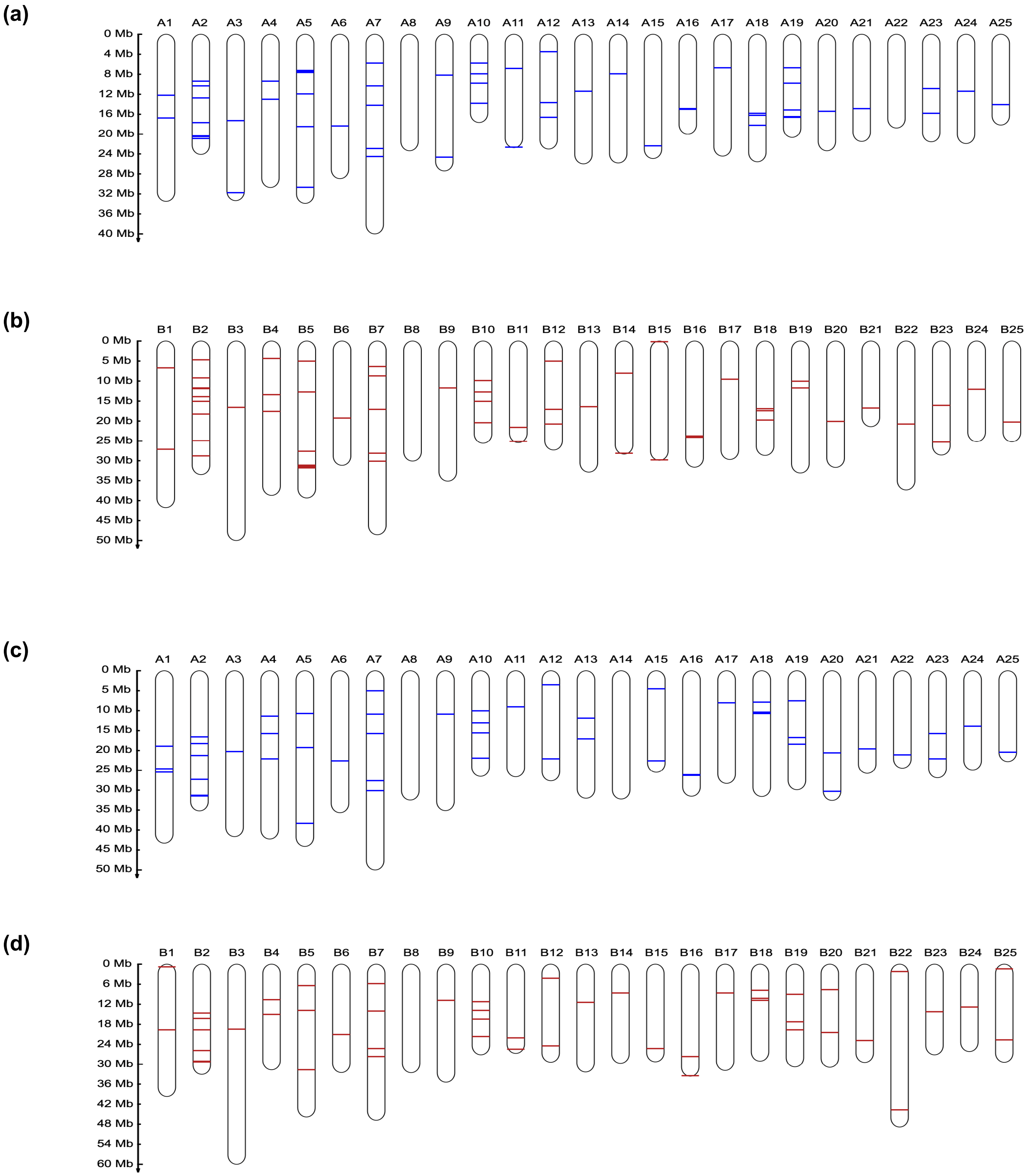

The distributions of the common carp subB genes in the A subgenome (a), subA genes in the B subgenome (b), goldfish sub genes in the A subgenome (c), goldfish subA genes in the B subgenome (d). The blue bars represent the exchanged subB genes to the A subgenome and the red bars show the exchanged subA genes to the B subgenome.

Supplementary Fig. 43. Identification and classification of homoeologous exchanges by comparing *P. tetrazona* read numbers between two homoeologous regions in the tetraploids

(a)

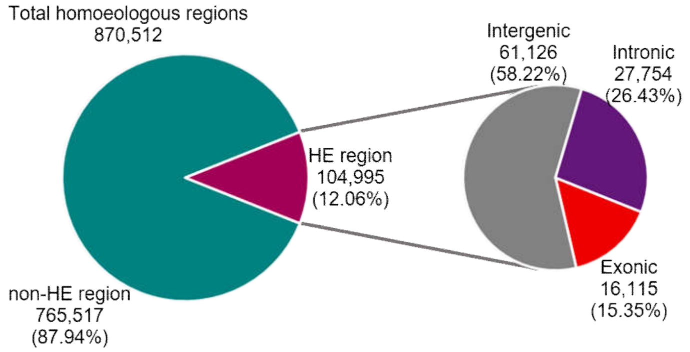

(b)

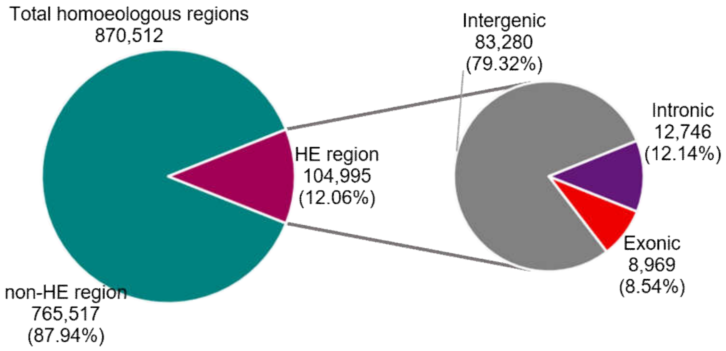

(c)

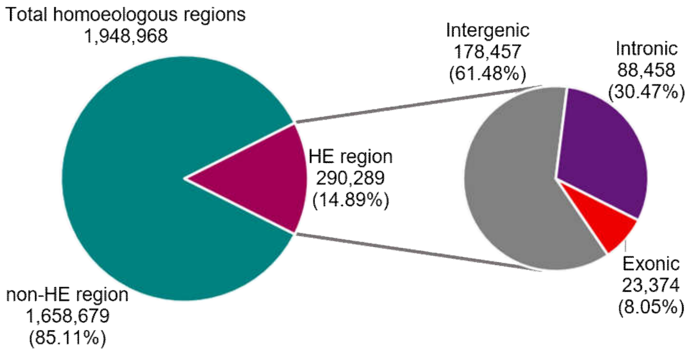

(d)

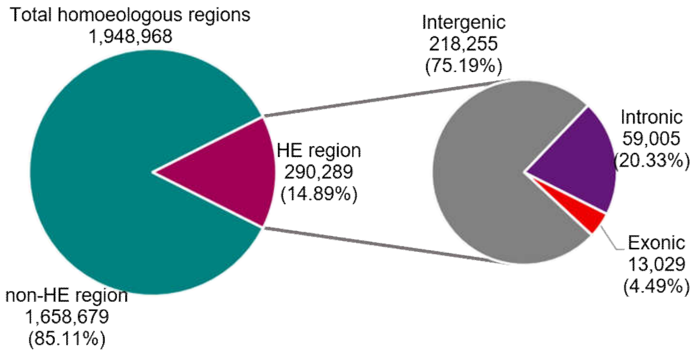

The figures (a) and (b) exhibit the HE events in the homoeologous regions in the common carp A and B subgenomes, respectively. (c) and (d) show the HE events in the homoeologous regions in the goldfish A and B subgenomes, respectively.

Supplementary Fig. 44. Gene collinearity among the genomes of common carp A, common carp B, *P. guichenoti*, and *P. tetrazona*

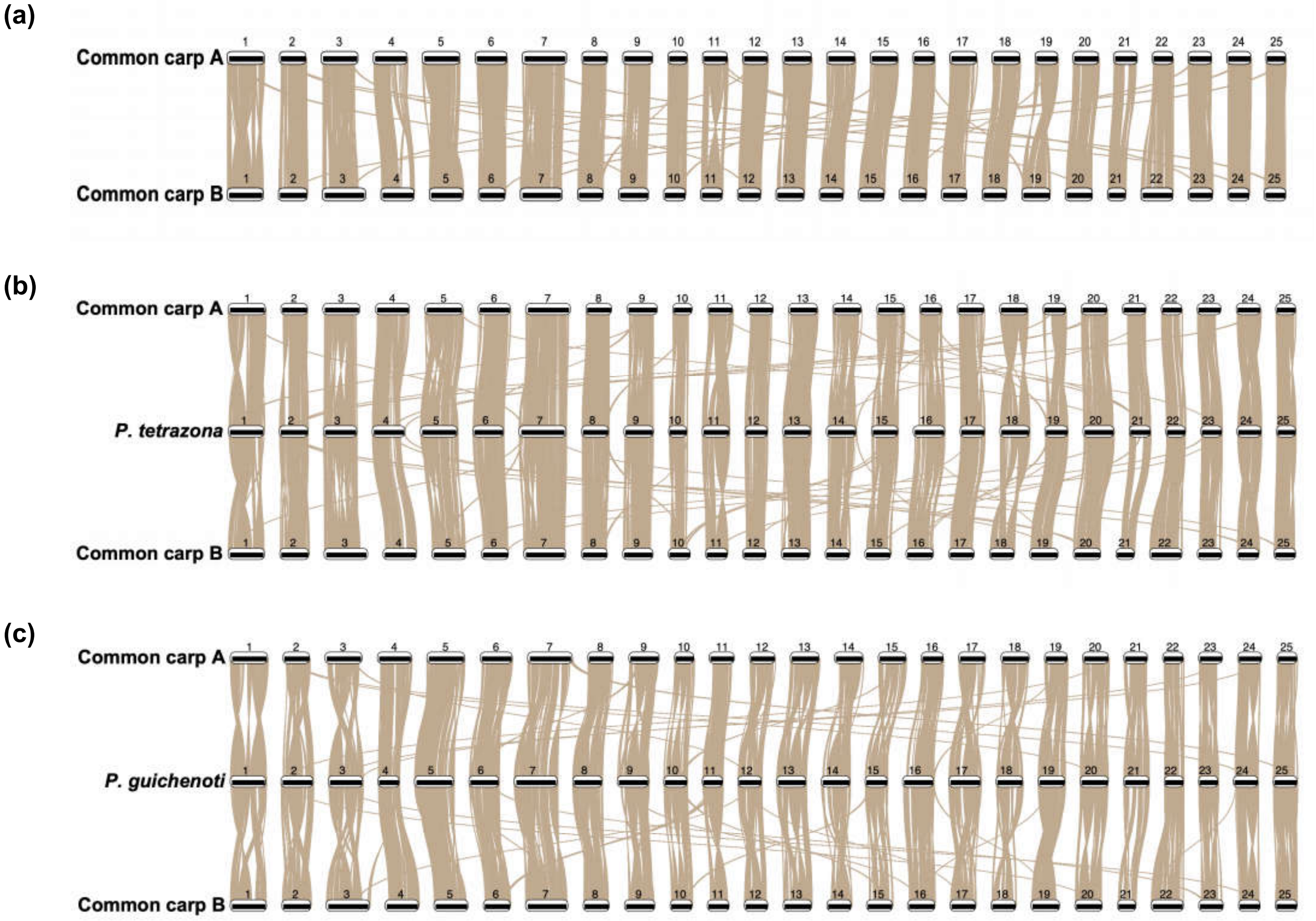

The figure (a) exhibits the 15,883 collinear gene pairs between the common carp subgenomes. In the figure (b), among 16,283 *P. tetrazona* genes having collinear common carp orthologues, 4,755 genes had common carp singletons, representing a homoeologue retention rate of 70.8% in the common carp genome. (c) Among 16,112 *P. guichenoti* genes having collinear common carp orthologues, 4,966 had common carp singletons, indicating a retention rate of 69.2% in the common carp genome.

Supplementary Fig. 45. Gene collinearity among the genomes of goldfish A, goldfish B, *P. guichenoti*, and *P. tetrazona*

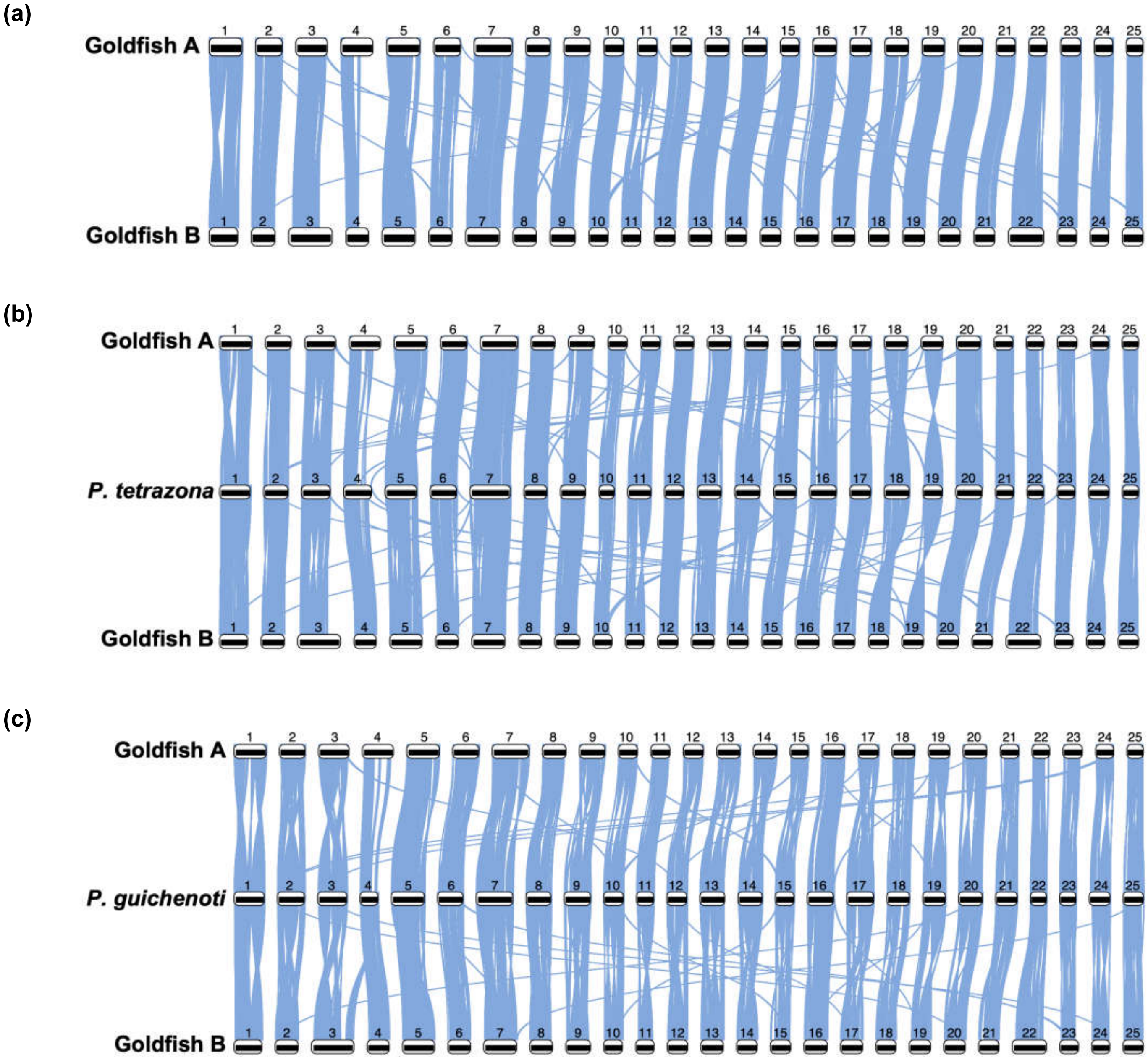

The goldfish subgenomes shared 14,401 collinear gene pairs (a). In the figure (b), among 16,241 *P. tetrazona* genes having collinear goldfish orthologues, 5,659 genes had goldfish singletons, representing a homoeologue retention rate of 65.2% in the goldfish genome. (c) Among 16,091 *P. guichenoti* genes having collinear goldfish orthologues, 5,835 had goldfish singletons, indicating a retention rate of 63.7% in the goldfish genome.

Supplementary Fig. 46. Gene collinearity among chr4 in different subgenomes

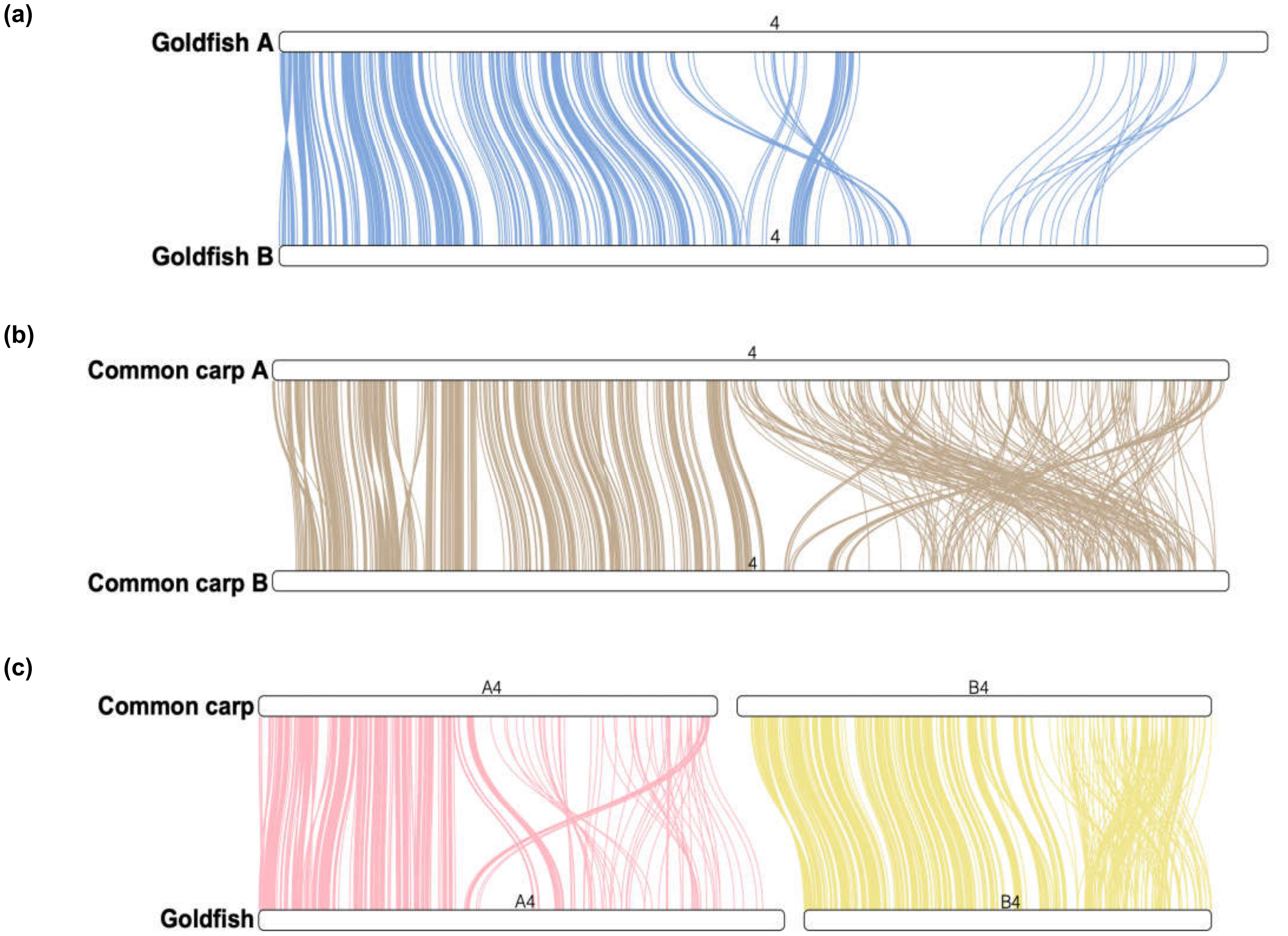

The collinear gene pairs (represented with color lines) between the goldfish homoeologous chr4 chromosomes (a) and between the common carp homoeologous chr4 pairs (b) show different conservation levels in chr4 between two tetraploids. In the figure (c), the pairs between two orthologous A4 chromosomes and between two orthologous B4 chromosomes showed different conservation levels in chr4 between two lineages.

Supplementary Fig. 47. The AR loss ratios in five Cyprinidae (sub)genomes

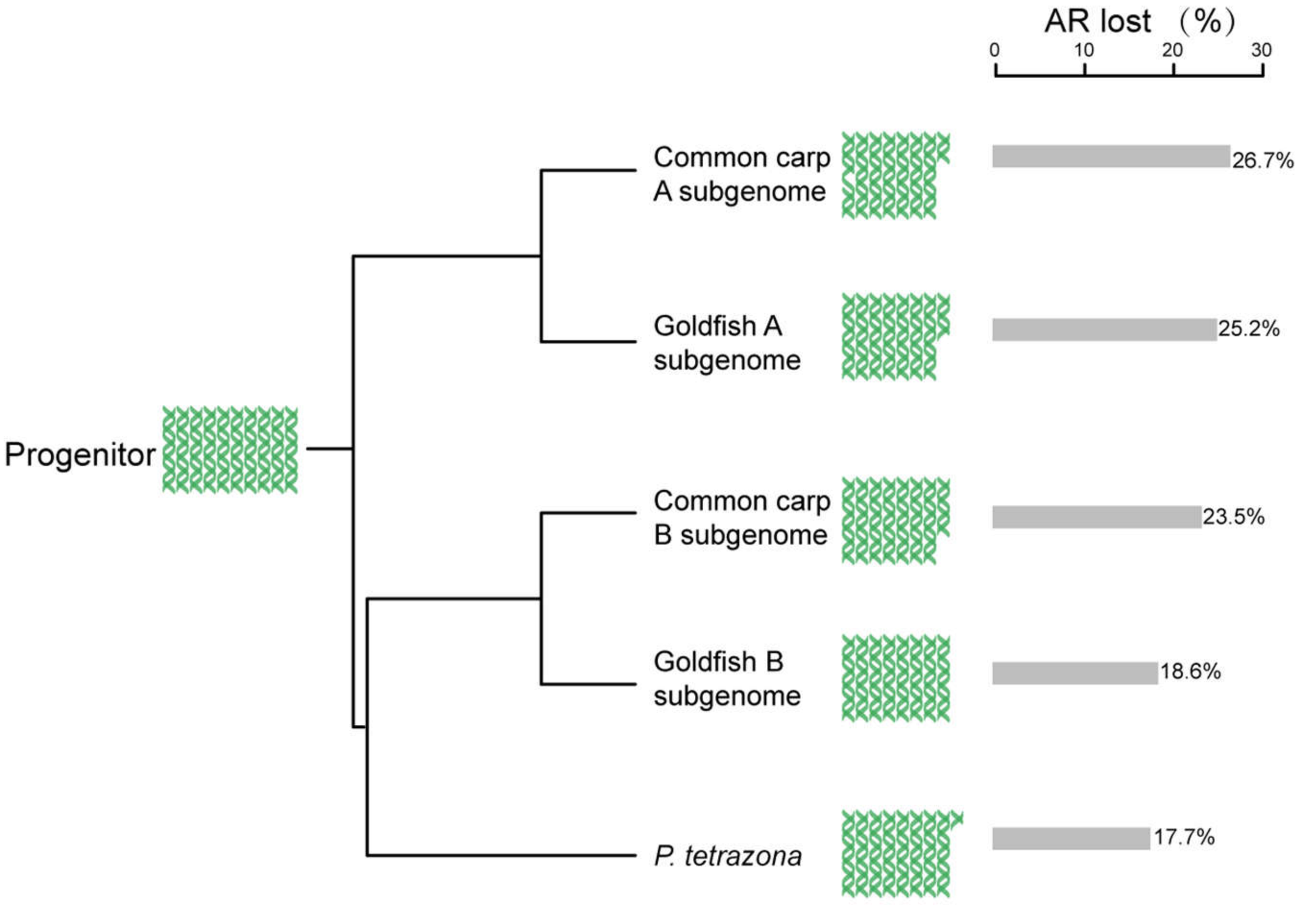

The green double helixes represent the ARs.

Supplementary Fig. 48. Schematic figure for the sequence compensation between subgenomes

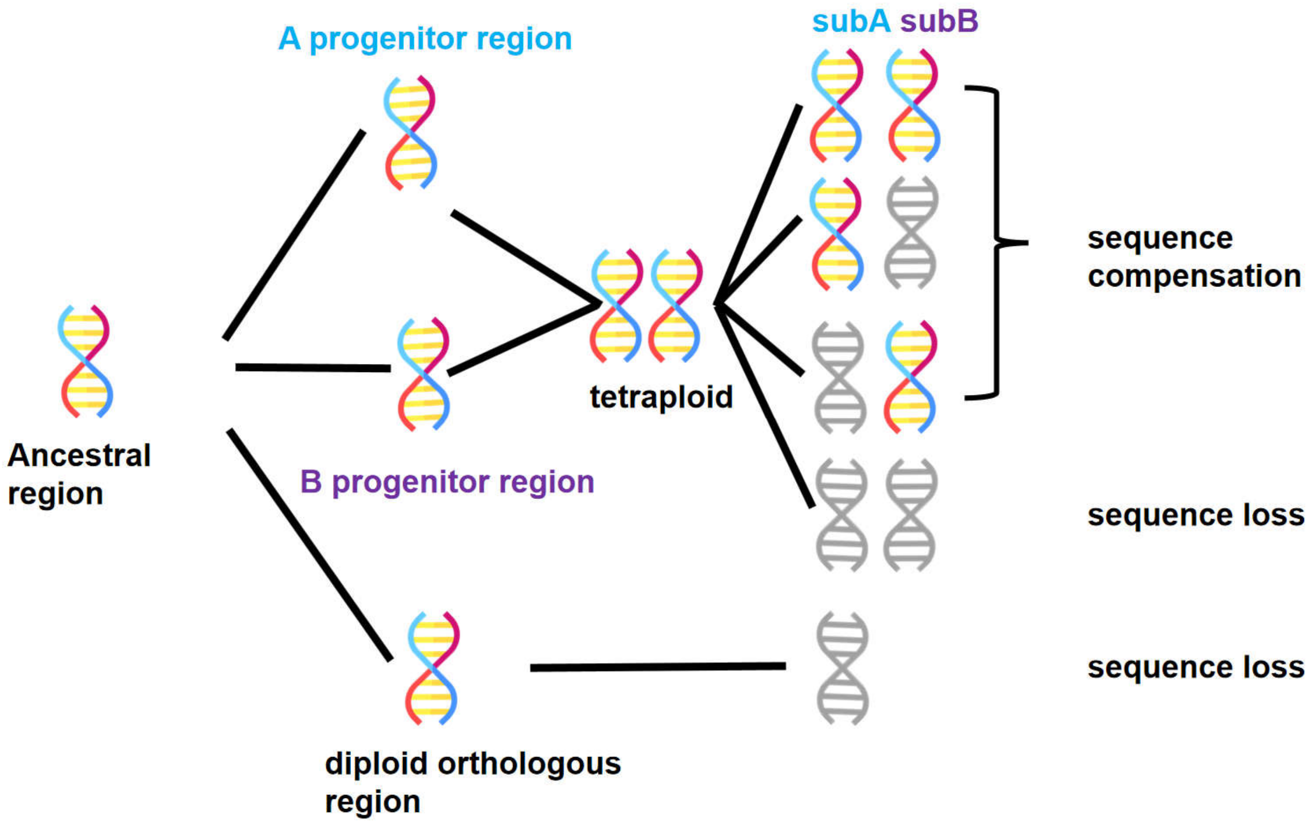

The grey DNA double helix represents a loss and the color one means a retention. For each AR, in each progenitor there existed one locus while the hybridization generated two loci in the tetraploid. The retention of at least one AR locus in the tetraploid produced the sequence compensation. The losses of two ARs in the tetraploid of one AR in the diploids result in the sequence loss.

Supplementary Fig. 49. The venn diagram of the enriched GO terms by the retained AGs in four subgenomes

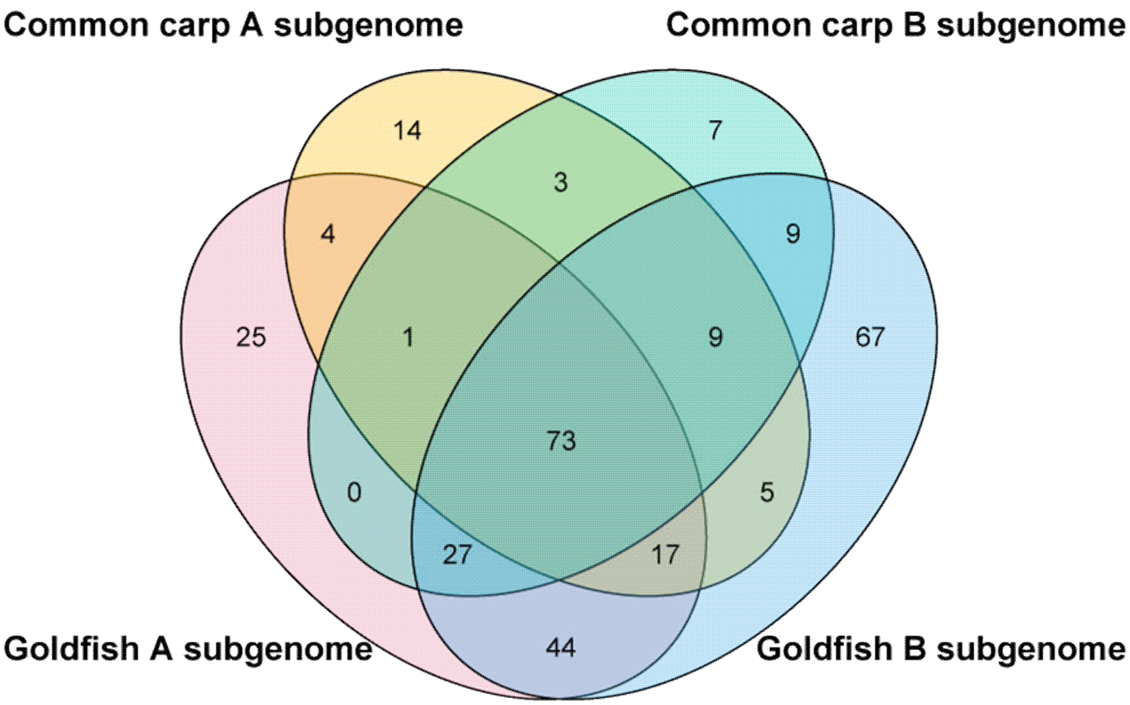

Supplementary Fig. 50. The enriched GO terms by the retained AGs in the tetraploid subgenomes

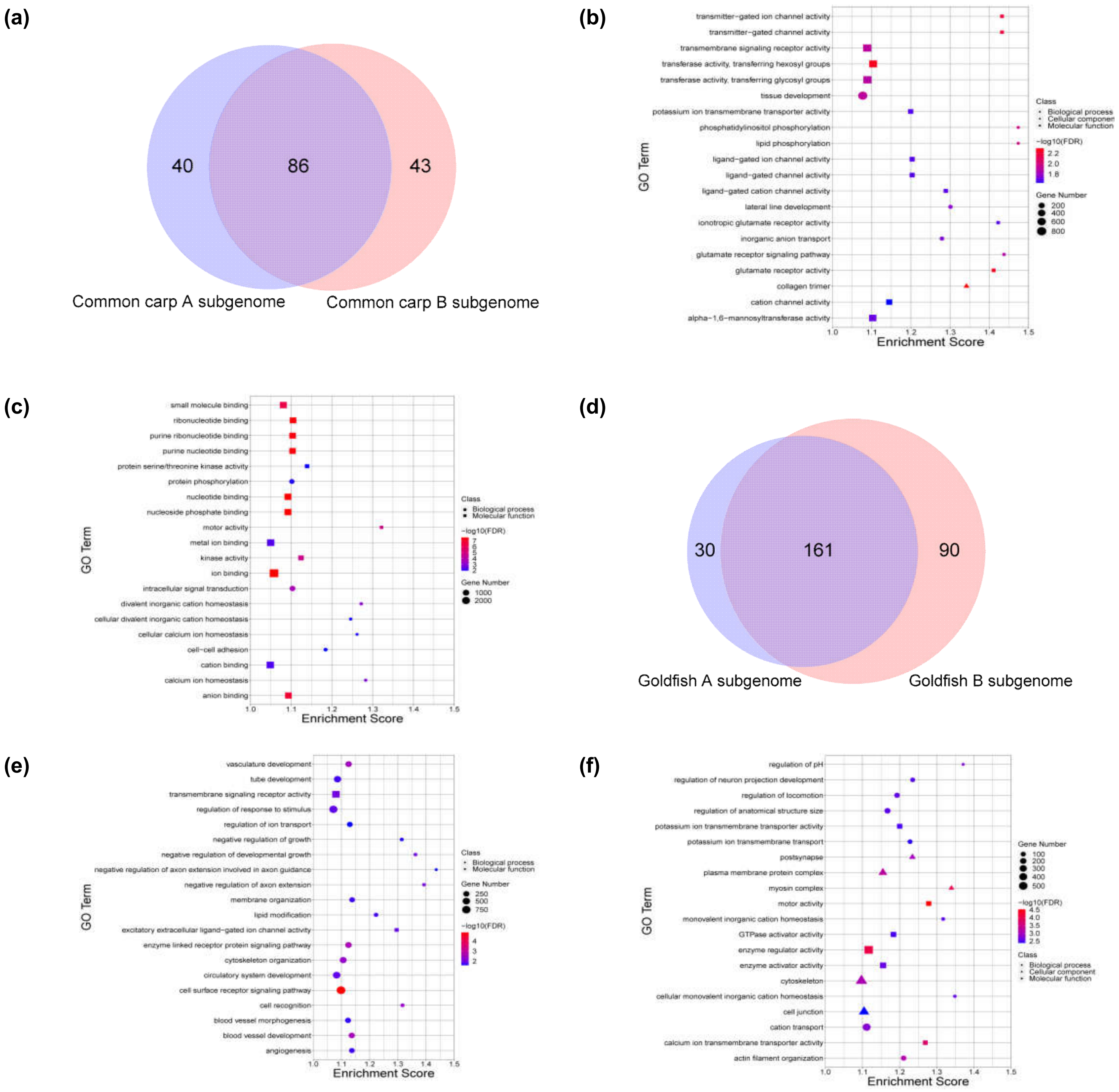

The venn diagram (a) shows the shared GO terms and specific GO terms enriched by the retained AGs in the common carp A and B subgenomes. The top 20 enriched GO terms in the common carp A subgenome and B subgenome are listed in (b) and (c), respectively. The venn diagram (d) shows the shared GO terms and specific GO terms enriched by the retained AGs in the goldfish A and B subgenomes. The top 20 enriched GO terms in the goldfish A subgenome and B subgenome are listed in (e) and (f), respectively.

Supplementary Fig. 51. *Ka/Ks* distribution of four types of homoeologues of common carp and goldfish using the *P. tetrazona* as a reference

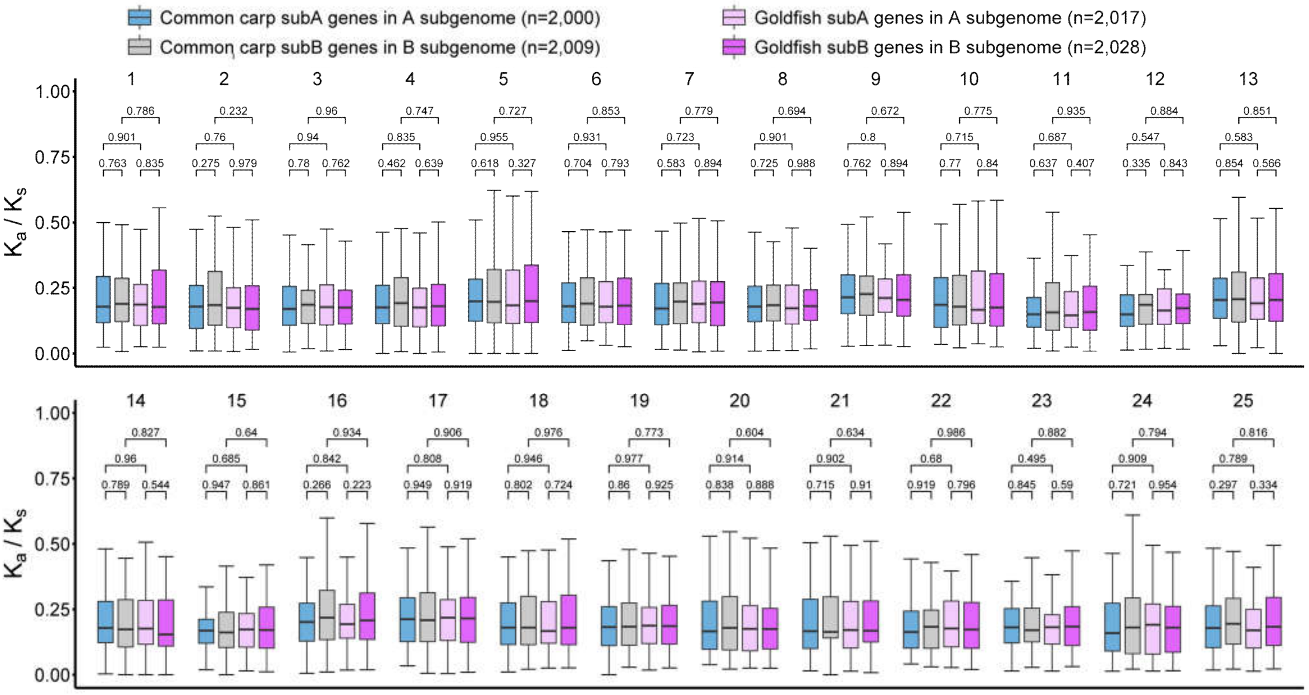

Boxplot of the *Ka/Ks* ratio distribution of homoeologues from 2,096 heptads in 25 homoeologous chromosome pairs in the common carp and goldfish (except the genes in the scaffolds or having no *Ka*/*Ks* ratios). The blue, grey, pink and purple boxplots indicate the *Ka/Ks* distributions of the hosted subA genes in the A subgenome, exchanged subA genes in the B subgenome, exchanged subB genes in the A subgenome, and hosted subB genes in the B subgenome, respectively. The definitions of the boxplots and whiskers are consistent with those in Fig. 3a. The P values are computed with two-sided Mann-Whitney *U* test. The n values in the brackets represent the gene numbers of four types on the chromosomes.

Supplementary Fig. 52. Alternative splicing number comparison of 2,096 sextuplet pairs among the common carp subA, common carp subB, goldfish subA, goldfish subB, *P. guichenoti*, and *P. tetrazona* in nine tissues

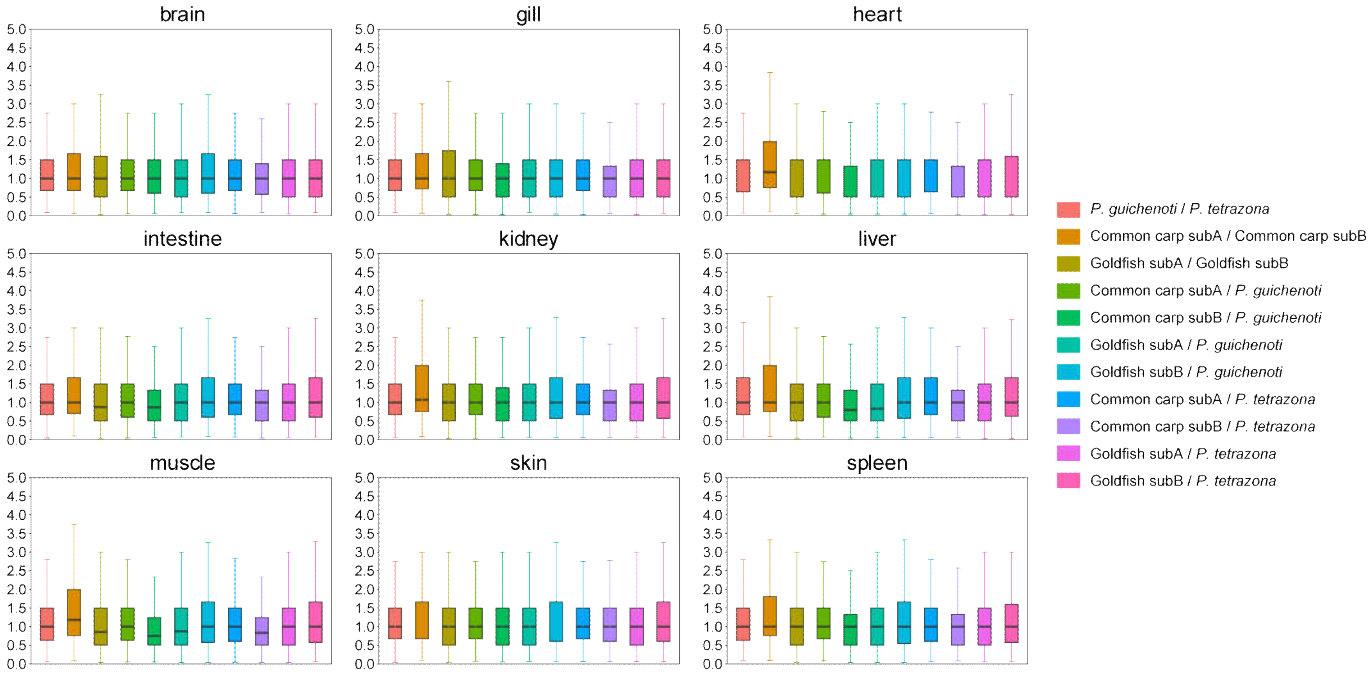

The definitions of the boxplots and whiskers are consistent with those in Fig. 3b. In each tissue, the n value is 2,096.

Supplementary Fig. 53. ***Trans*-splicing events of the homoeologous genes and orthologous genes from 2,096 pairs across nine tissues**

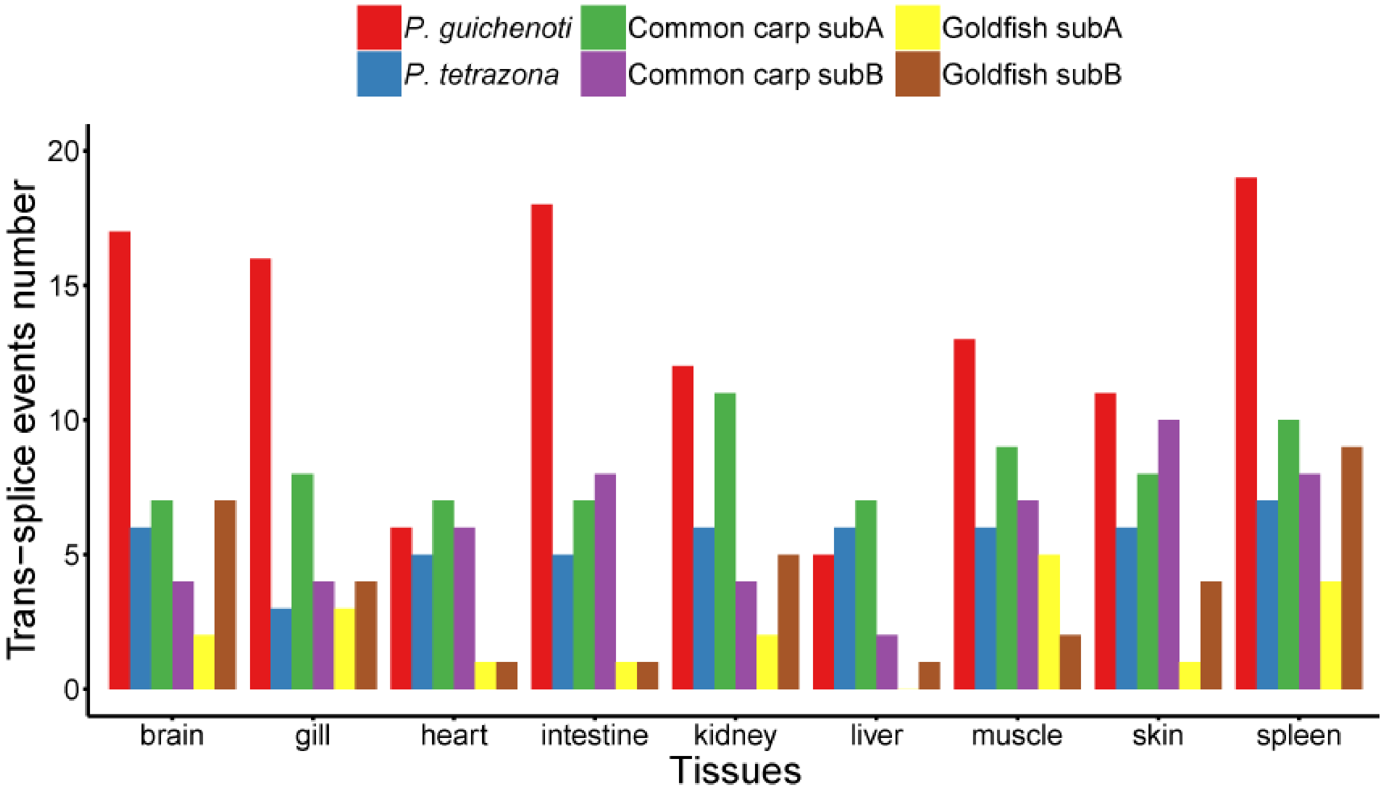

Supplementary Fig. 54. ***Trans*-splicing maps of *P. guichenoti***

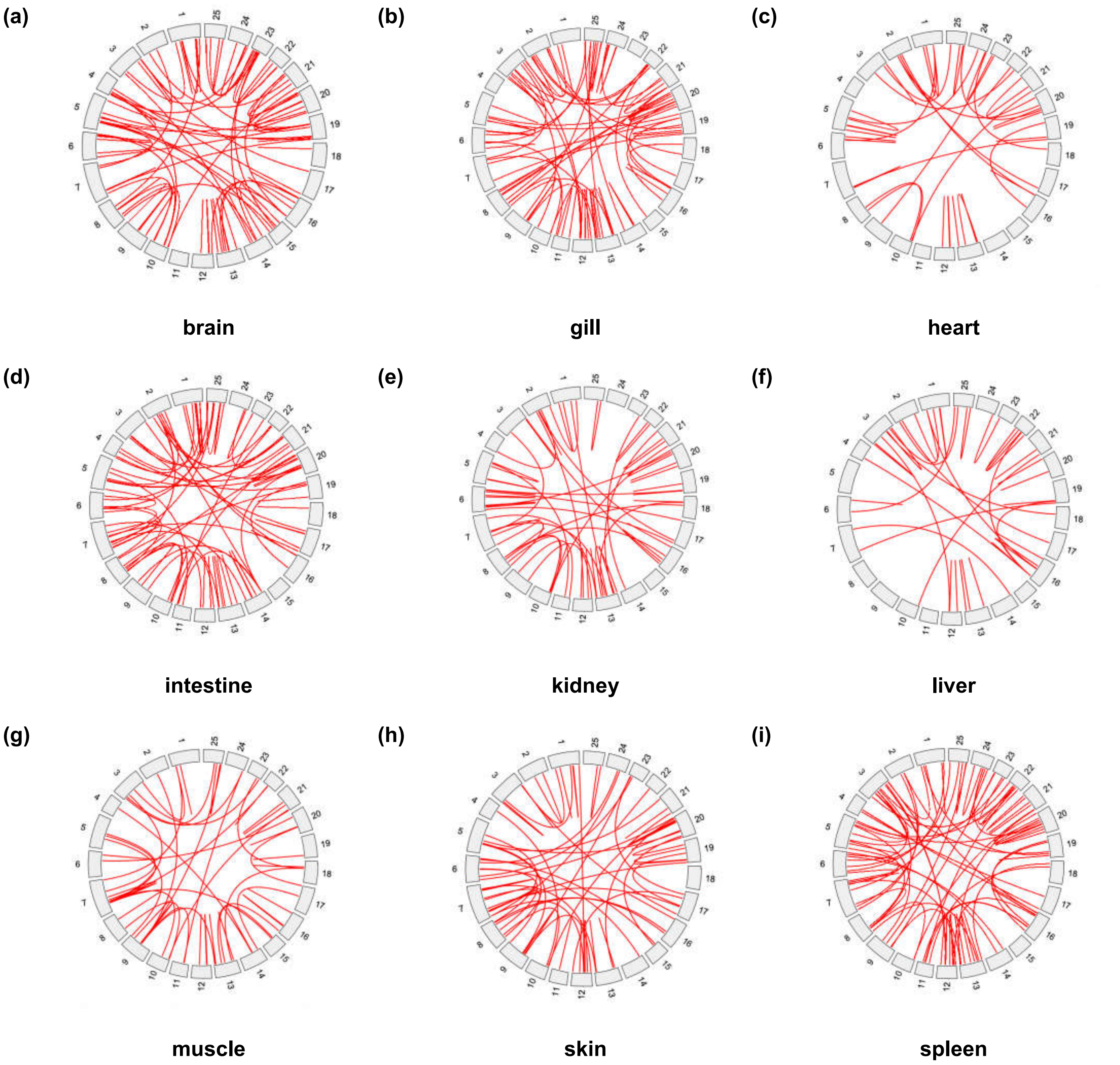

A red line links two reads from different genomic loci, representing a *trans*-splicing event.

Supplementary Fig. 55. *Trans*-splicing maps of *P. tetrazona*

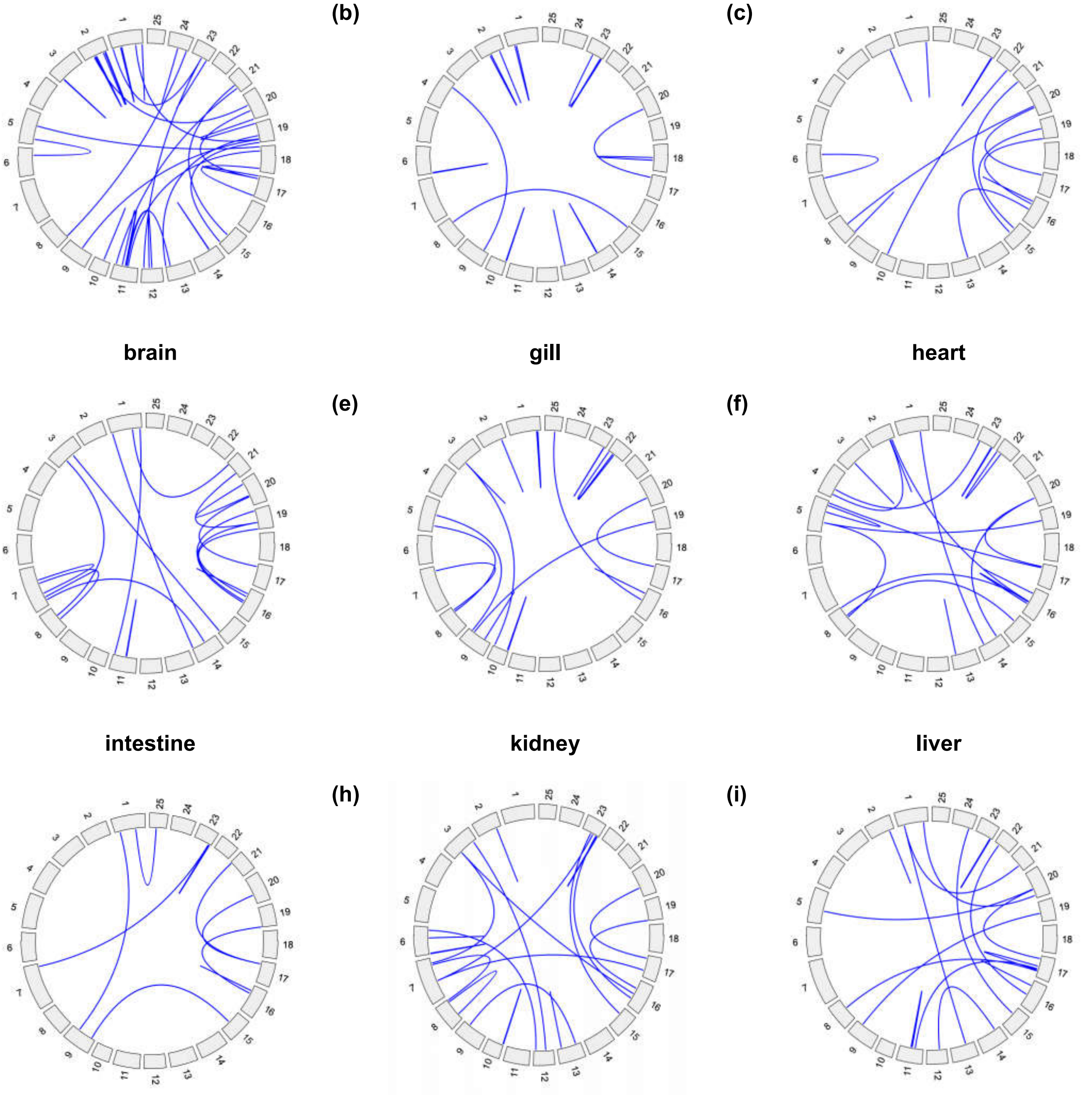

(a) brain **(b)** gill **(c)** heart

(d) intestine **(e)** kidney **(f)** liver

(g) muscle **(h)** skin **(i)** spleen

A blue line links two reads from different genomic loci, representing a *trans*-splicing event.

Supplementary Fig. 56. ***Trans*-splicing maps of common carp**

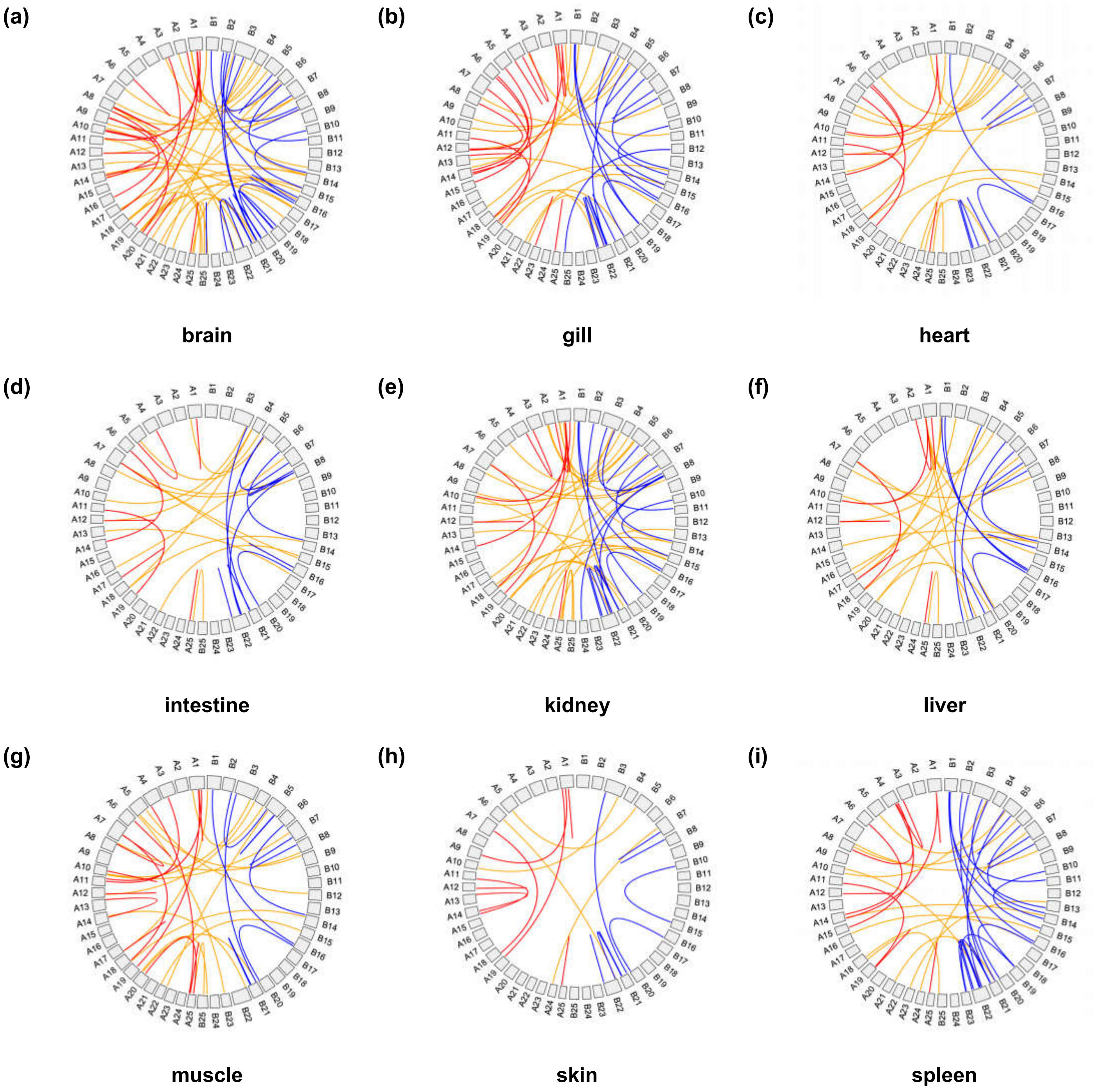

A red line represents a TS event generated from two transcripts located in different loci of the A subgenome. A blue line represents a TS event from two loci in the B subgenome and orange lines symbolize TS events occurring between A and B subgenomes.

Supplementary Fig. 57. ***Trans*-splicing maps of goldfish**

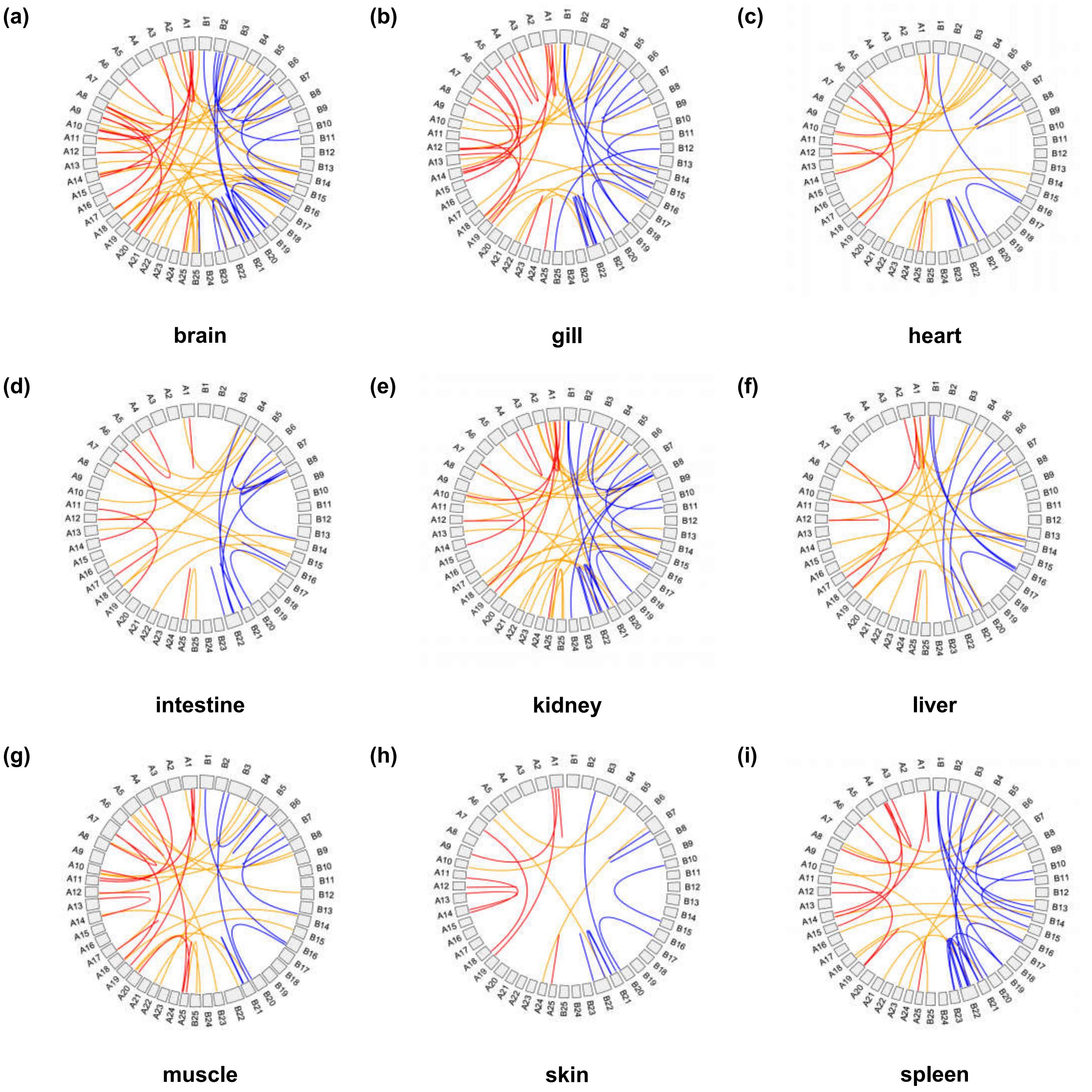

A red line represents a TS event generated from two transcripts located in different loci of the A subgenome. A blue line represents a TS event from two loci in the B subgenome and orange lines symbolize TS events occurring between A and B subgenomes.

Supplementary Fig. 58. *Trans*-splicing event numbers in nine tissues of all genes from the common carp A, common carp B, goldfish A, goldfish B, *P. guichenoti*, and *P. tetrazona*

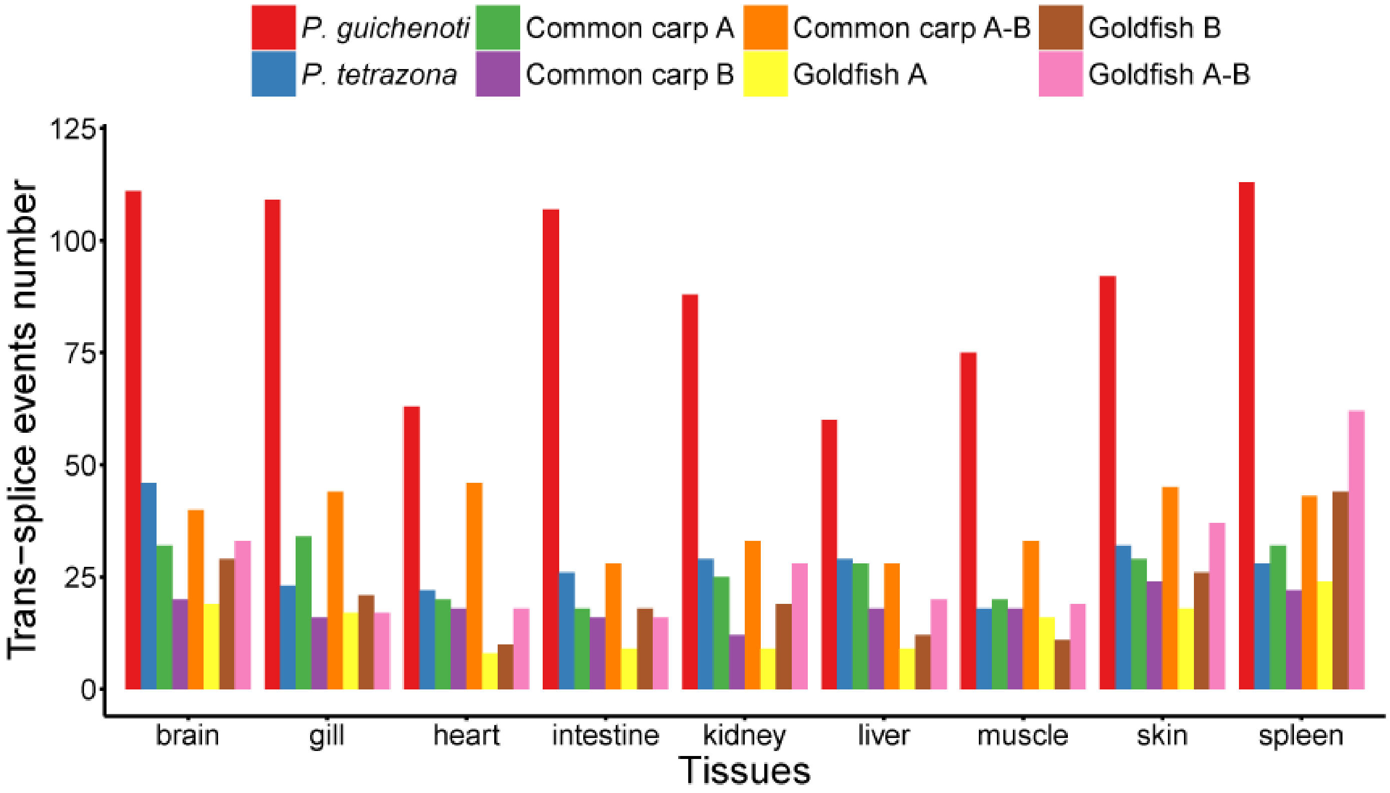

Supplementary Fig. 59. Sequence identities and coverages of long reads and de novo assembled transcripts used to validate TS events

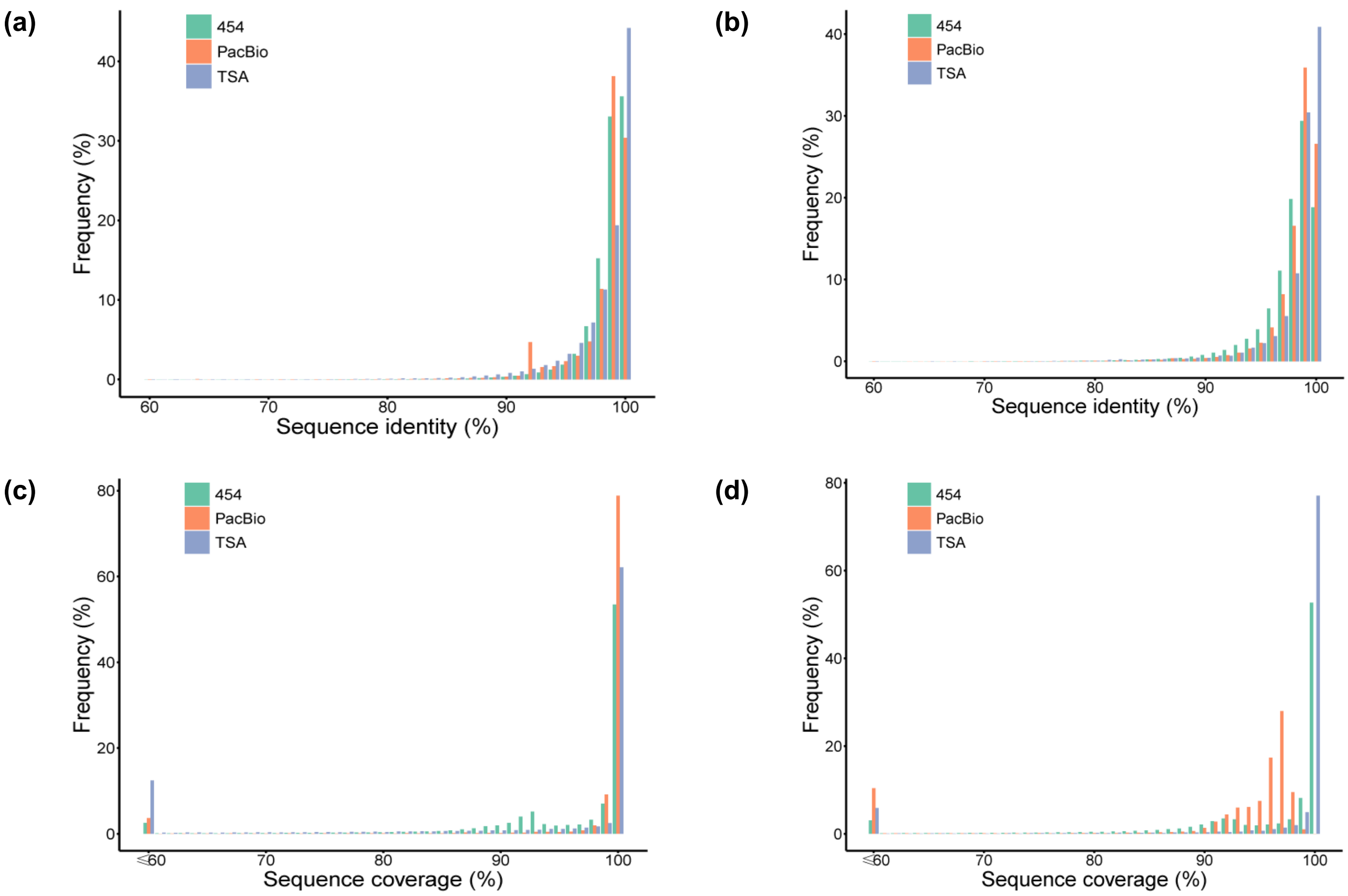

The sequence identity distributions of 454 RNA-seq reads, Pacbio Iso-seq reads, and TSA sequences of the common carp (a) and goldfish (b) aligned to the corresponding reference genomes, respectively. The sequence coverage distributions of 454 RNA-seq reads, Pacbio Iso-seq reads, and TSA sequences of common carp (c) and goldfish (d) aligned to the corresponding reference genomes.

Supplementary Fig. 60. Dosage compensation effect of the tetraploid homoeologues

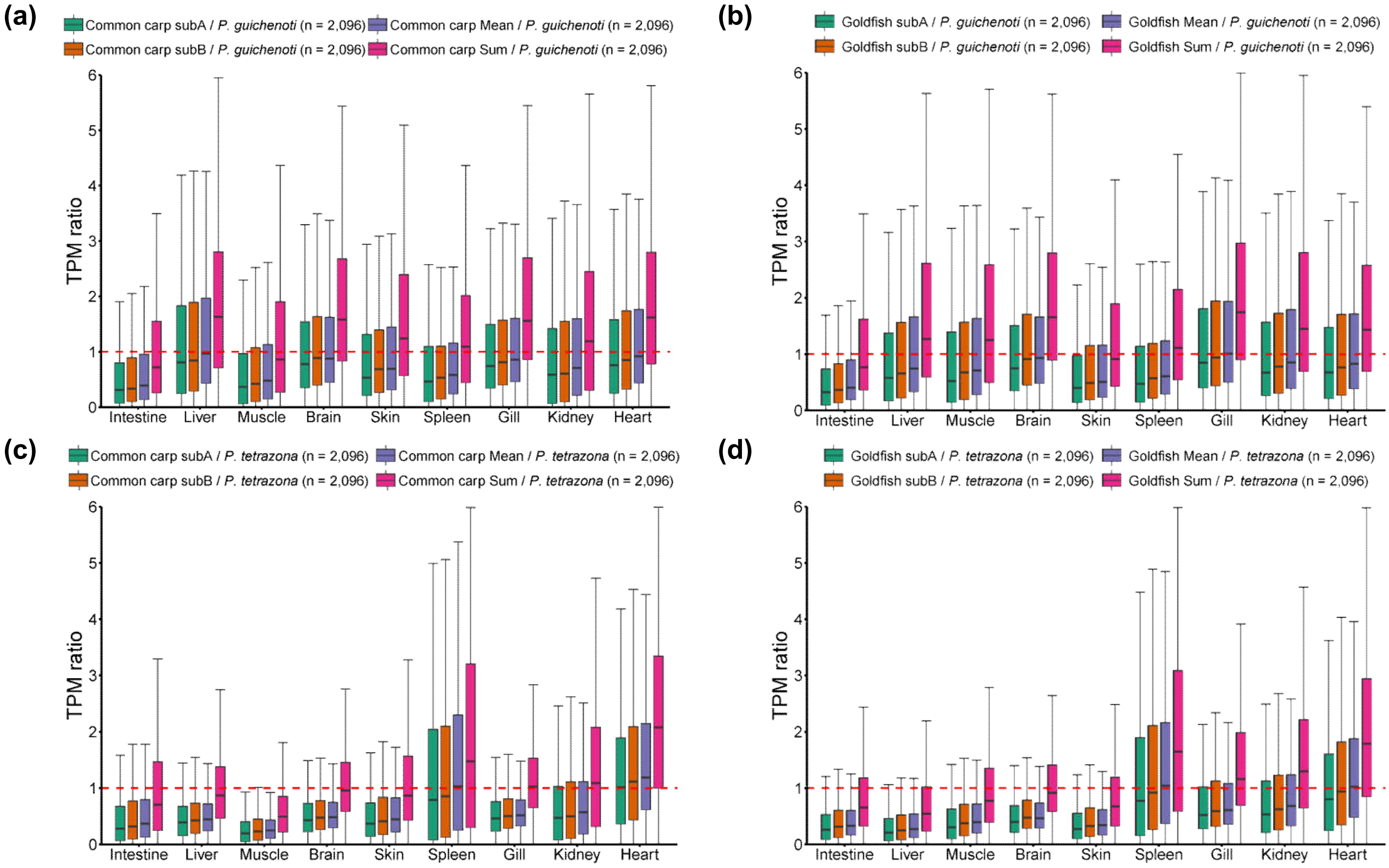

Compared to diploid orthologs, the tetraploid homoeologues had lowered gene expression level. Compared with the expression levels of the *P. guichenoti* orthologues, figures (a) and (b) depicts the expression levels of the homoeologues (green and orange boxplots), the levels of pseudo-ancestral genes (purple boxplots), and the mean levels of homoeologues (pink boxplots) in the common carp and goldfish, respectively. Compared with the expression levels of the *P. tetrazona* orthologues, figures (c) and (d) depicts the expression levels of the homoeologues, the levels of pseudo-ancestral genes, and the mean levels of homoeologues in common carp and goldfish, respectively. The definitions of the boxplots and whiskers are consistent with those in Fig. 3a. The n values in the brackets represent the expression comparison numbers.

Supplementary Fig. 61. The expression correlation between homoeologues across nine tissues in the common carp and goldfish

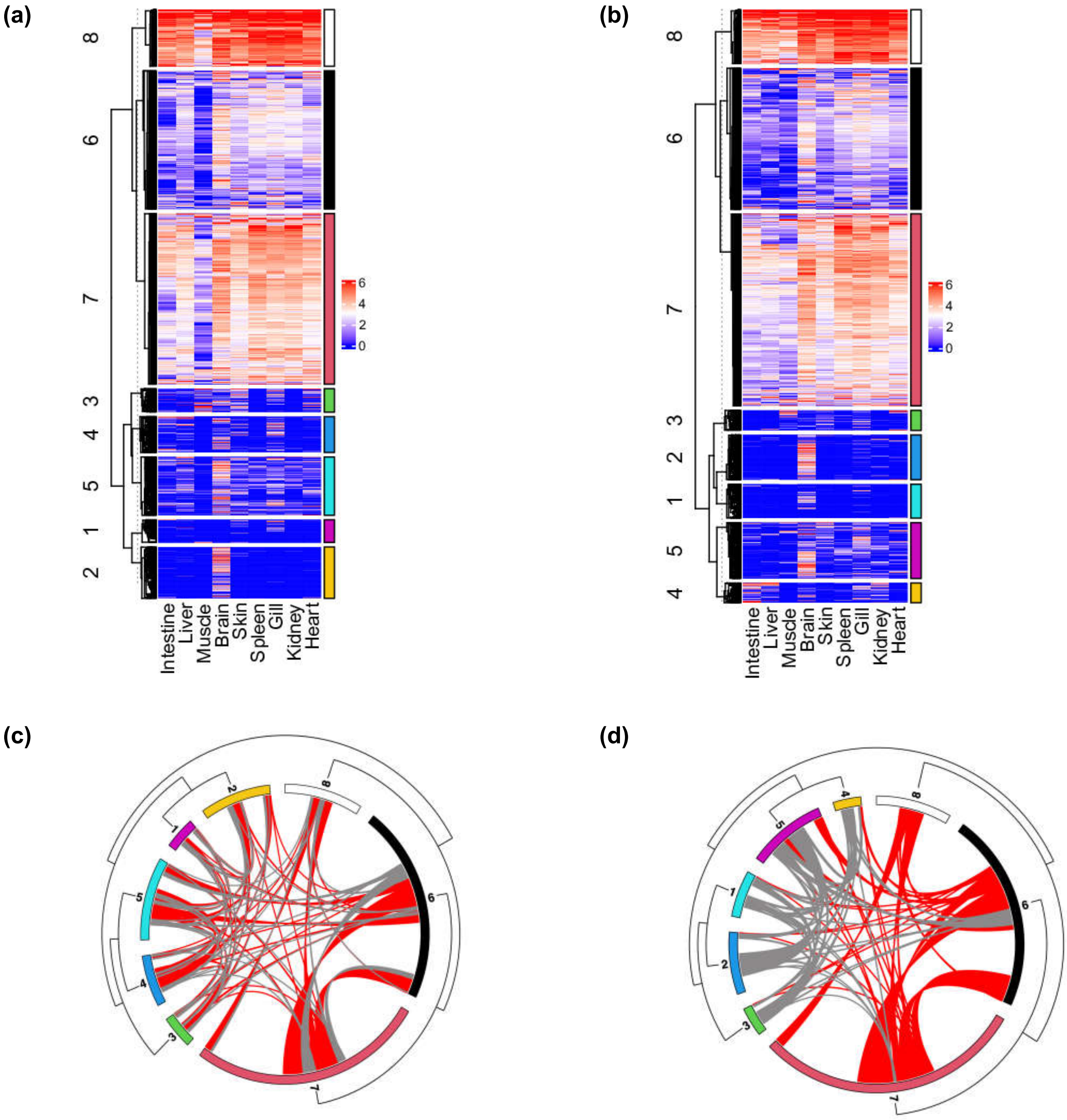

In common carp (a) and goldfish (b), 4,192 homoeologues from 2,096 pairs are clustered into eight groups based on their expression patterns in tissue profiles. Circos plots indicate the homoeologue assignments to eight groups in common carp (c) and goldfish (d). The red lines link homoeologues in different groups with significantly correlated expression, and the grey lines connect homoeologues in different groups without significant expression correlation.

Supplementary Fig. 62. The expression dominance towards the tetraploid B subgenomes in nine tissues

(a)

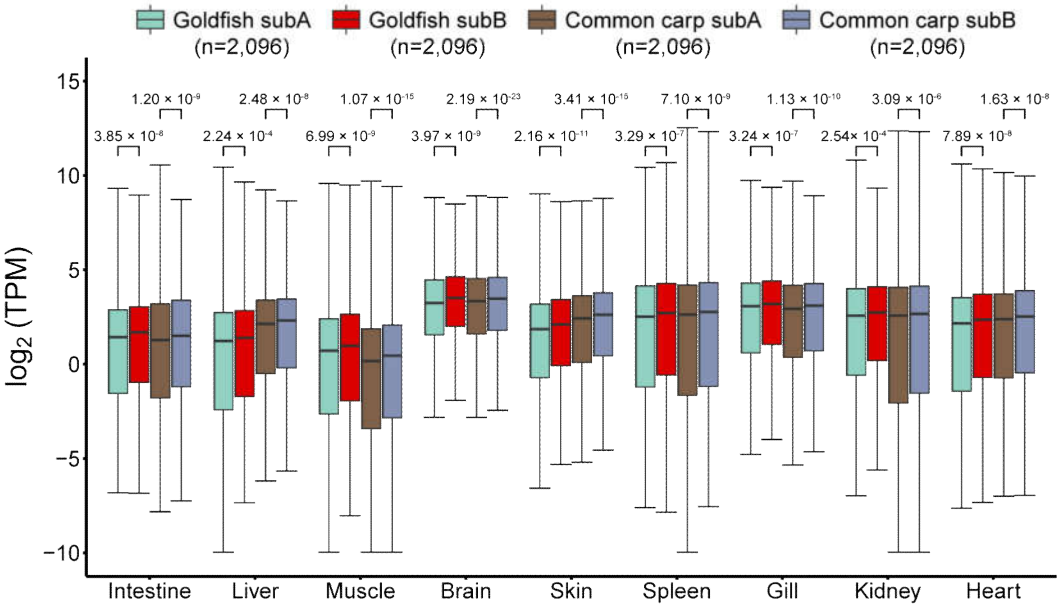

(b)

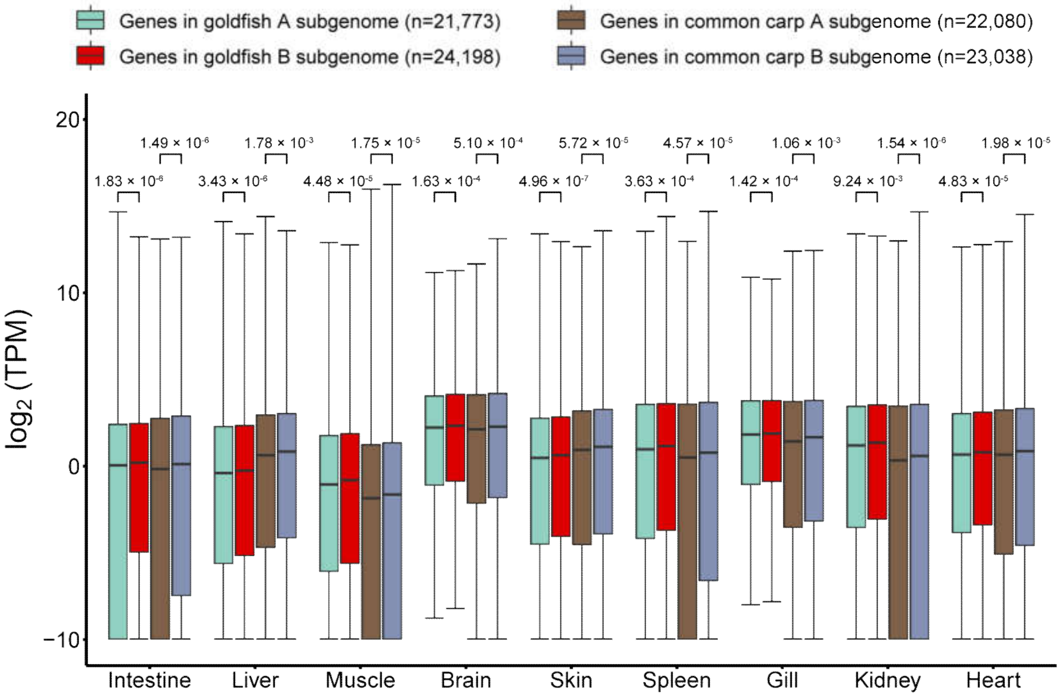

(a) In general, the expression levels of 2,096 subB genes (represented with n values) are significantly higher than those of 2,096 subA copies in all tissues using two-sided Wilcoxon test with paired sample option. (b) All genes in the B subgenome have significantly higher expression levels than the genes from the A subgenome in nine tissues using two-sided Mann-Whitney *U* test. The n values showed the compared gene numbers in four subgenomes. The definition of the boxplots and whiskers in (a) and (b) are consistent with those in Fig. 3a.

Supplementary Fig. 63. Expression correlations and distances between the common carp genes and their *P. guichenoti* (*P. tetrazona*) orthologs across nine tissues

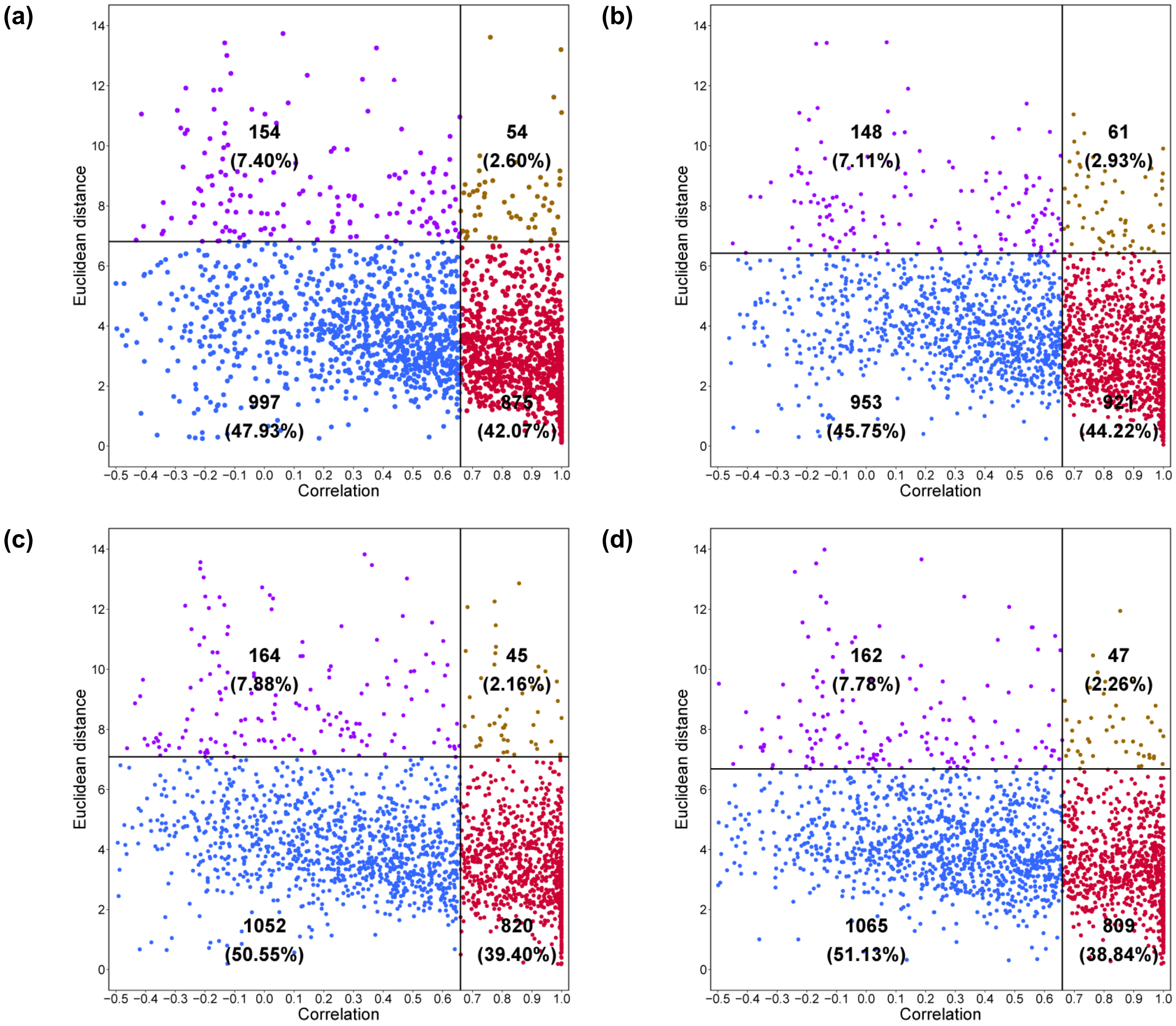

Dotplots of expression correlation (x axis) and Euclidean distance (y axis) between orthologous pairs of common carp subA genes and *P. guichenoti* (a), common carp subB genes and *P. guichenoti* (b), common carp subA genes and *P. tetrazona* (c), common carp subB genes and *P. tetrazona* (d). Each box lists the number and the percentage of orthologous pairs. The meanings of the upper left box (purple), lower left box (blue), upper right box (brown), and lower right box (red), are the same to those in Fig. 4c.

Supplementary Fig. 64. Expression correlation and distance between the goldfish homoeologues and their *P. guichenoti* (*P. tetrazona*) orthologs across nine tissues

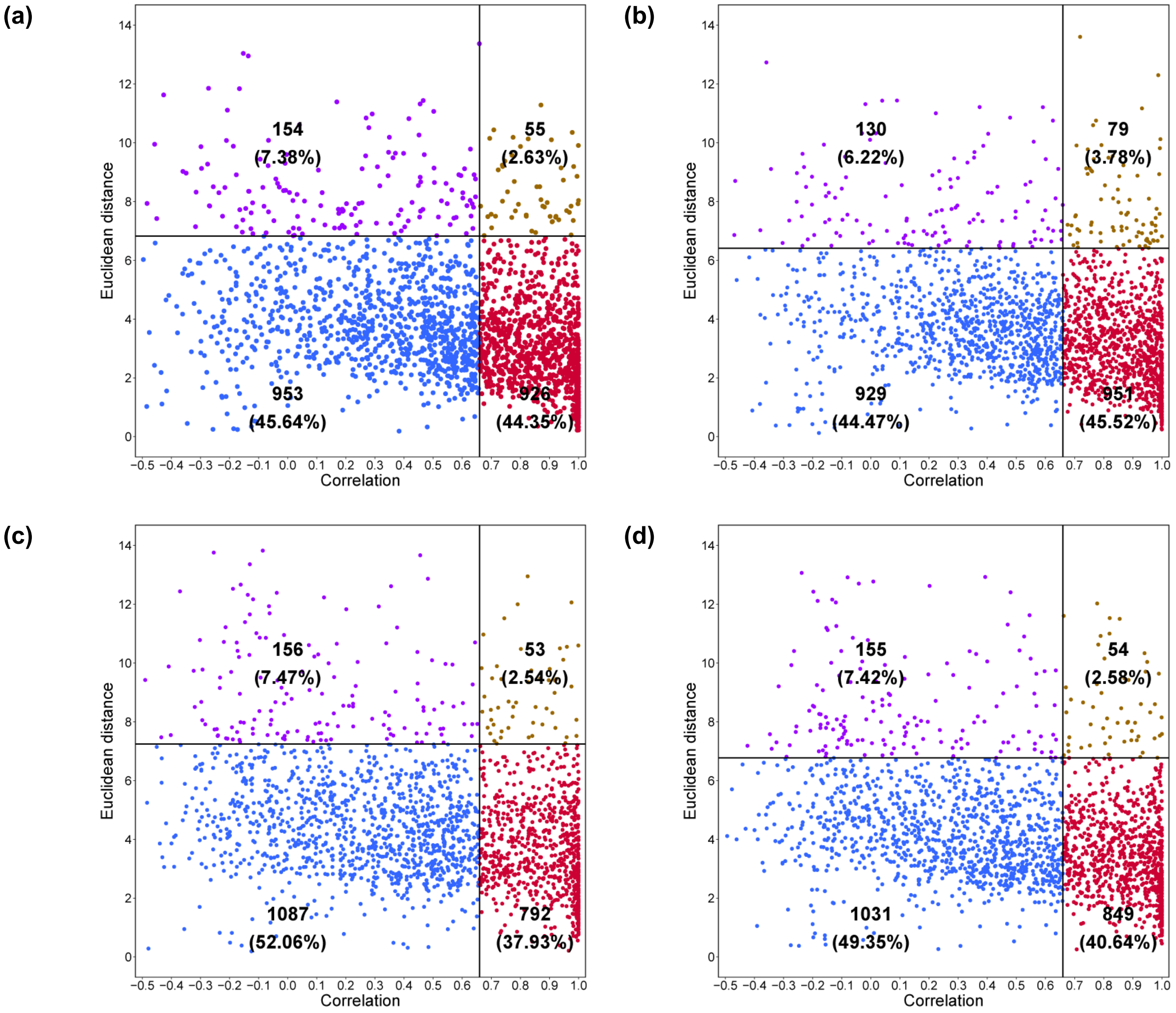

Dotplots of expression correlation (x axis) and Euclidean distance (y axis) between orthologous pairs in goldfish subA genes and *P. guichenoti* (a), goldfish subB genes and *P. guichenoti* (b), goldfish subA genes and *P. tetrazona* (c), goldfish subB genes and *P. tetrazona* (d). Each box lists the number and the percentage of the orthologous pairs. The meanings of the upper left box (purple), lower left box (blue), upper right box (brown), and lower right box (red), are the same to those in Fig. 4c.

Supplementary Fig. 65. ***Ka/Ks*** **levels of the co-expressed group, sub-functionalized group, non-functionalized group, and neo-functionalized group across tissues and conditions in common carp**

(a)

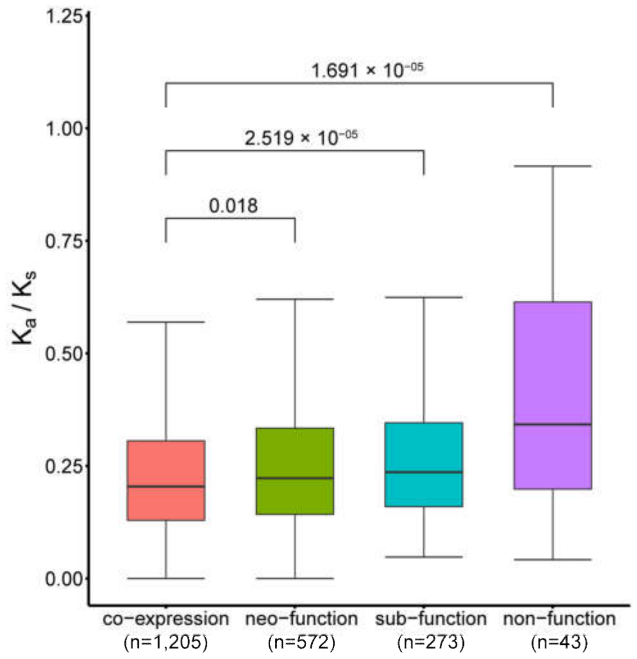

(b)

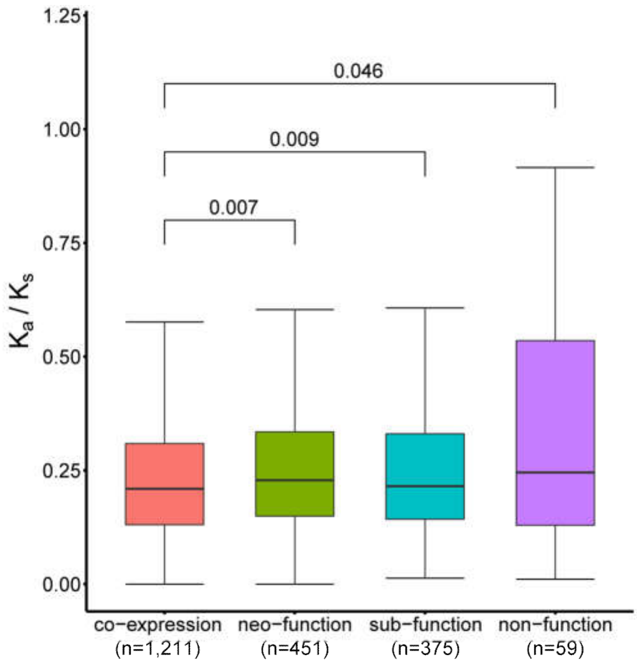

The *Ka/Ks* levels of the co-expressed group were significantly lower than those of the sub-functionalized group, non-functionalized group, and neo-functionalized group in tissues (a) and conditions (b) using two-sided Mann-Whitney *U* test. P values are shown on the lines connecting the compared groups. The n values represent the gene numbers in four groups. The definitions of the boxplots and whiskers in (a) and (b) are consistent with those in Fig. 3a.

Supplementary Fig. 66. ***Ka/Ks*** **levels of the co-expressed group, sub-functionalized group, non-functionalized group, and neo-functionalized group across tissues and conditions in goldfish**

(a)

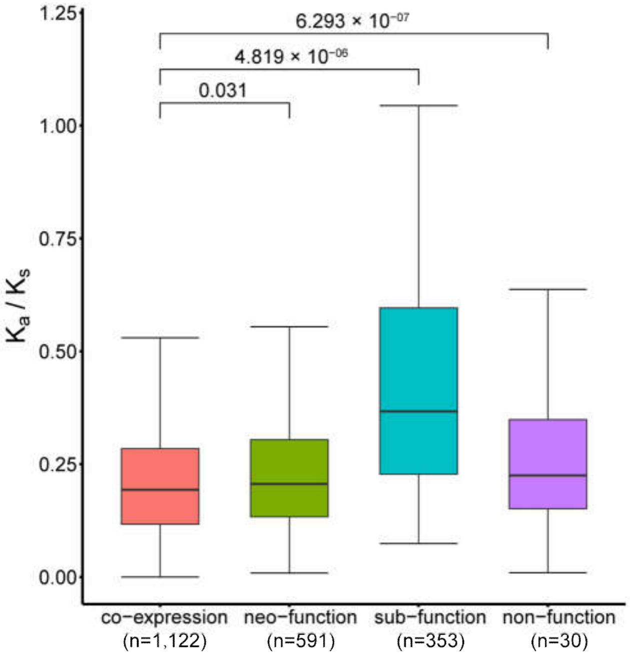

(b)

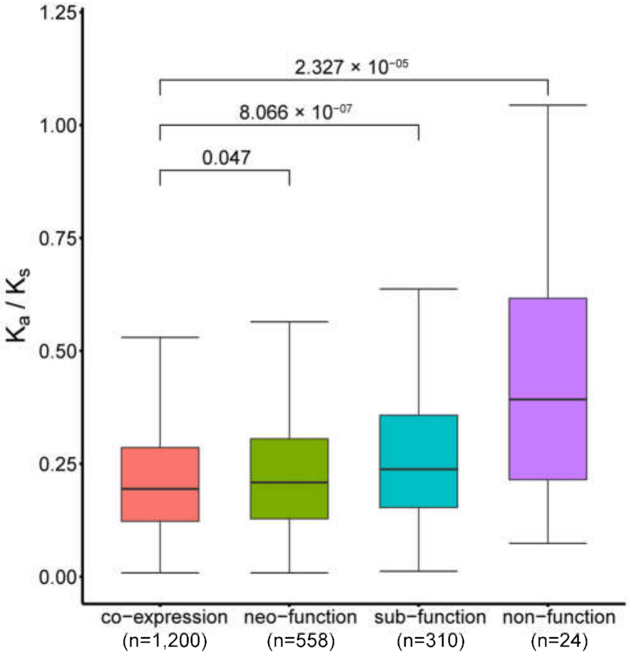

The *Ka/Ks* levels of the co-expressed group were significantly lower than those of sub-functionalized group, non-functionalized group, and neo-functionalized group in goldfish tissues (a) and conditions (b) using two-sided Mann-Whitney *U* test. The P values are shown on the lines connecting the compared groups. The n values represent the gene numbers in four groups. The definitions of the boxplots and whiskers are consistent with those in Fig. 3a.

Supplementary Fig. 67. The expression correlation between the homoeologues across nine conditions in common carp and goldfish

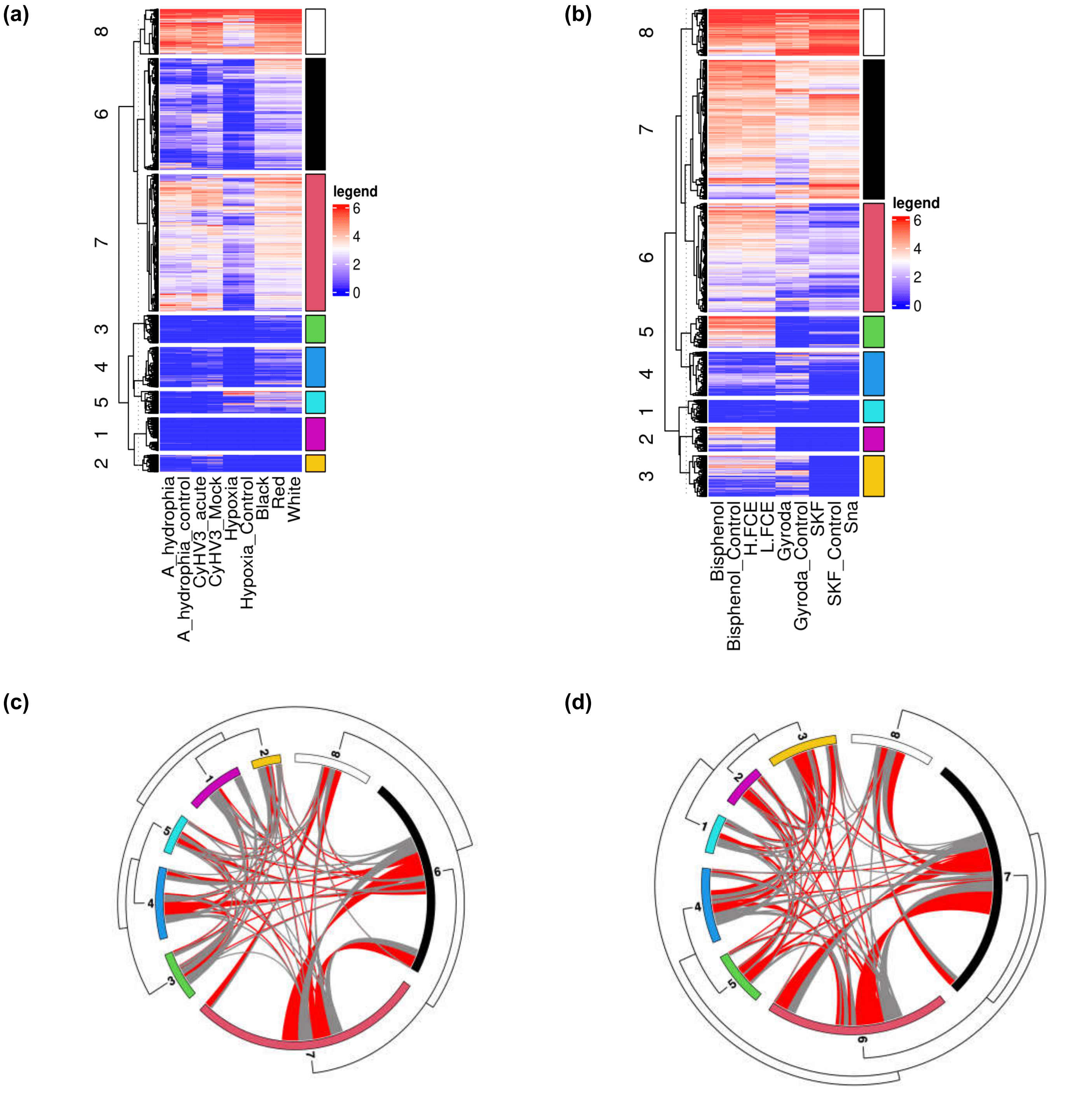

In common carp (a) and goldfish (b), 4,192 homoeologues from 2,096 pairs were clustered into eight groups based on their expression patterns in condition profiles. Circos plots indicate the homoeologue assignments to eight groups in common carp (c) and goldfish (d). The red lines link homoeologues in different groups with significantly correlated expression, and grey lines connect homoeologues in different groups without significant expression correlation.

Supplementary Fig. 68. Expression dominance towards the subB genes in nine conditions of common carp

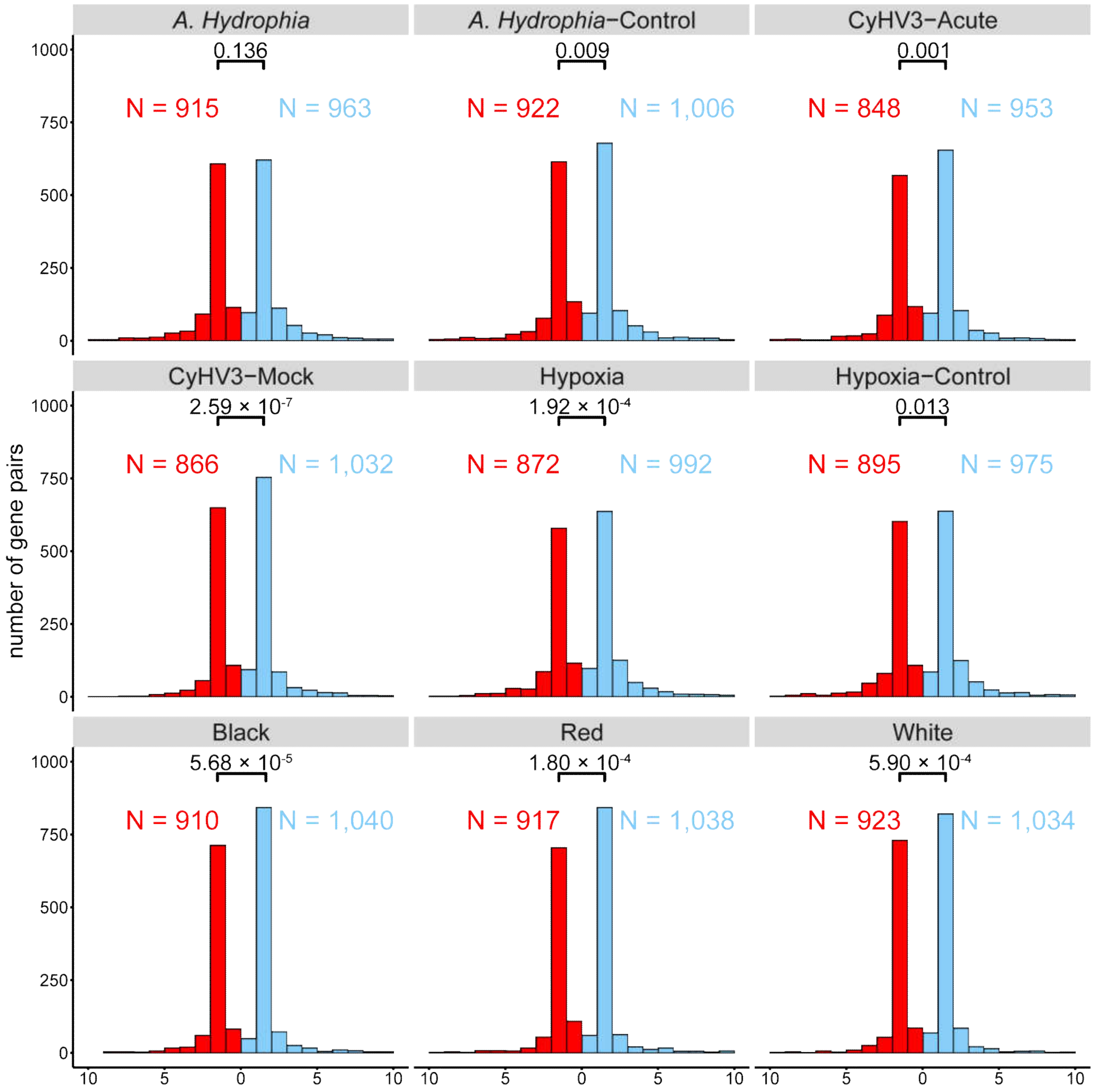

Expression histograms of homoeologues in nine conditions of common carp show the degree of expression differences. The N values indicate the numbers of the dominant subA genes (red bars) and subB genes (blue bars). The P values are computed with two-sided Chi-square test and shown on the lines connecting the compared groups.

Supplementary Fig. 69. Expression dominance towards the subB genes in nine conditions of goldfish

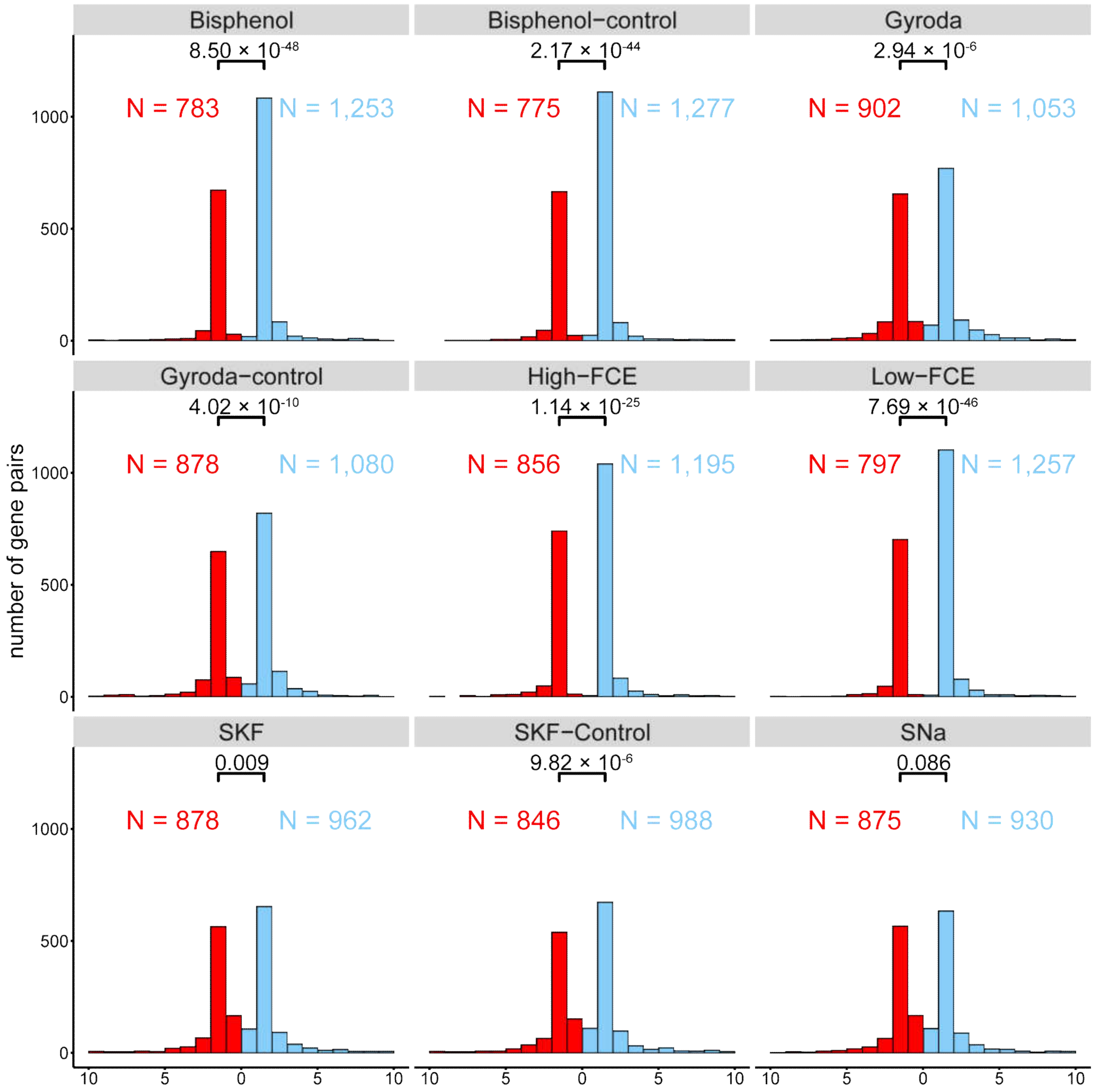

Expression histograms of homoeologues in nine conditions of goldfish show the degree of expression differences. The N values indicate the numbers of the dominant subA genes (red bars) and subB genes (blue bars). The P values are computed with two-sided Chi-square tests and shown on the lines connecting the compared groups.

Supplementary Fig. 70. Expression correlations and distances between the common carp homoeologues across nine conditions

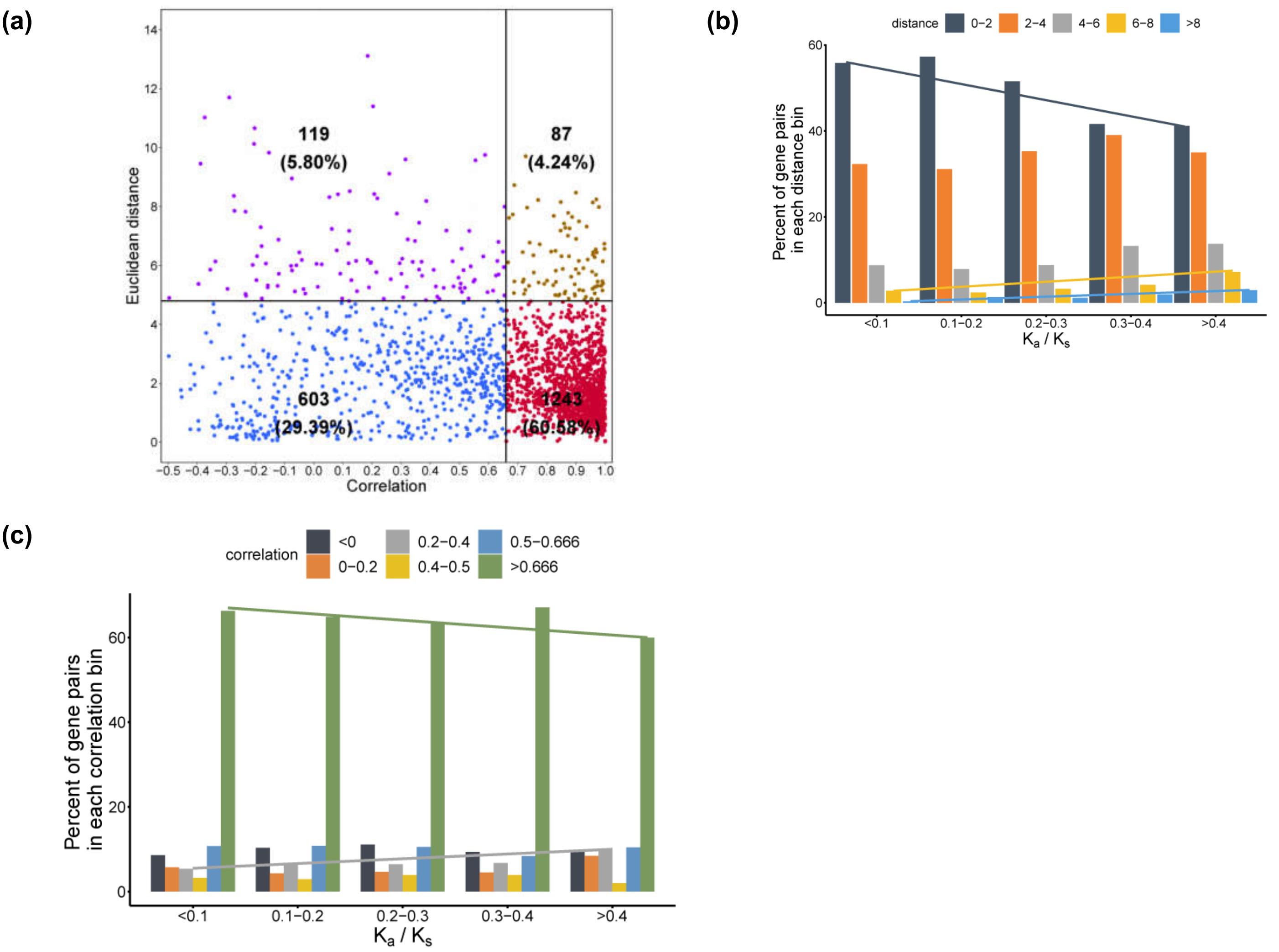

(a) Dotplot of expression correlation (x axis) and Euclidean distance (y axis) between common carp homoeologues across nine conditions. Each box lists the number and percentage of homoeologous pairs. The meanings of the upper left box (purple), lower left box (blue), upper right box (brown), and lower right box (red), are the same to those in Fig. 4c. (b) Euclidean distance distribution in different *Ka/Ks* groups. (c) Expression correlation distribution in different *Ka/Ks* groups.

Supplementary Fig. 71. The expression correlations and distances between the goldfish homoeologues across nine conditions

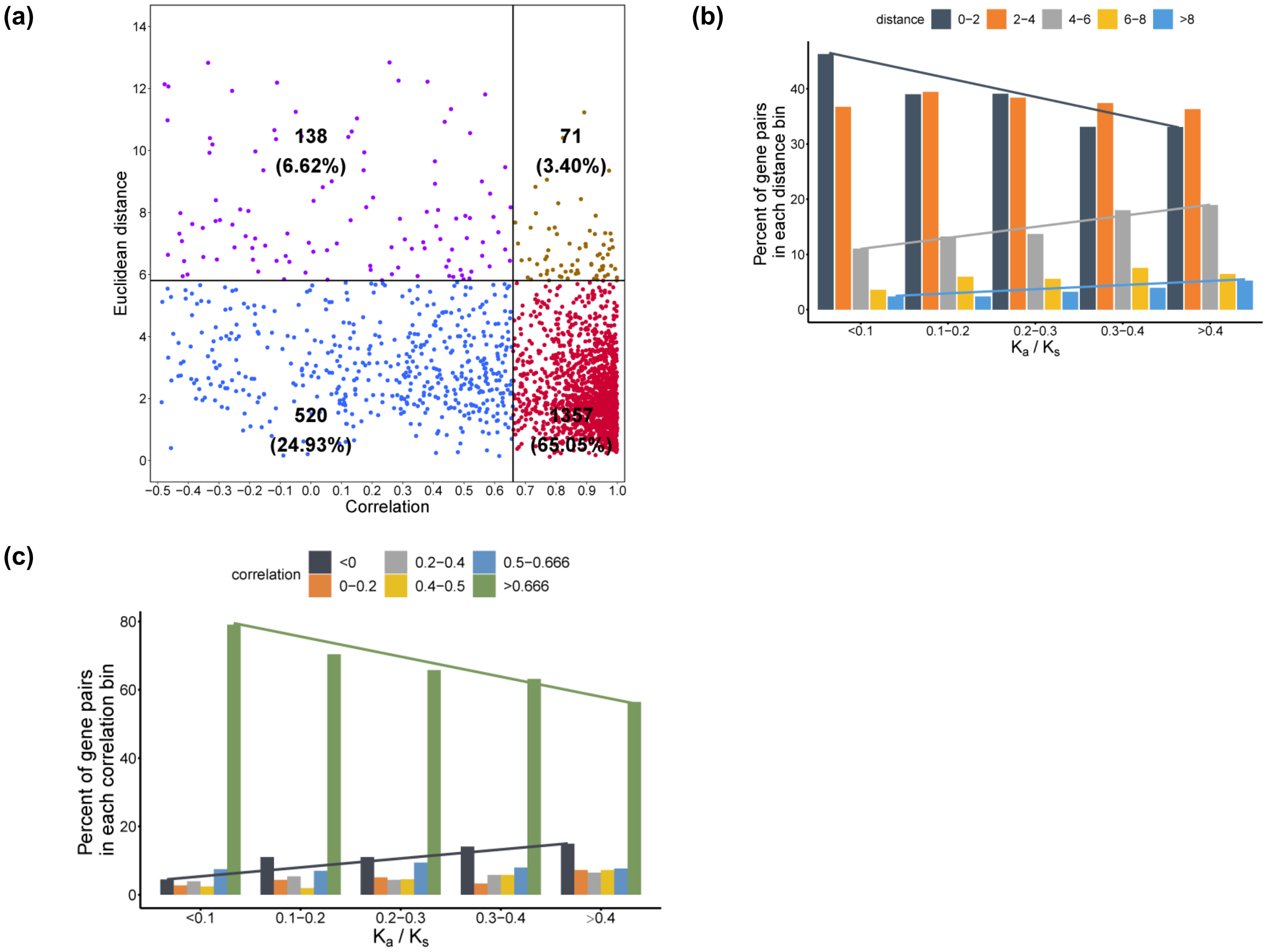

(a) Dot plot of expression correlation (x axis) and Euclidean distance (y axis) between goldfish homoeologous across nine conditions. Each box lists the number and percentage of homoeologous pairs. The meanings of the upper left box (purple), lower left box (blue), upper right box (brown), and lower right box (red), are the same to those in Fig. 4c. (b) Euclidean distance distribution in different *Ka/Ks* groups. (c) Expression correlation distribution in different *Ka/Ks* groups.

Supplementary Fig. 72. Statistics of co-expressed, sub-functionalized, neo-functionalized, and non-functionalized groups of 2,096 homoeologous pairs in nine tissues and nine conditions of common carp and goldfish

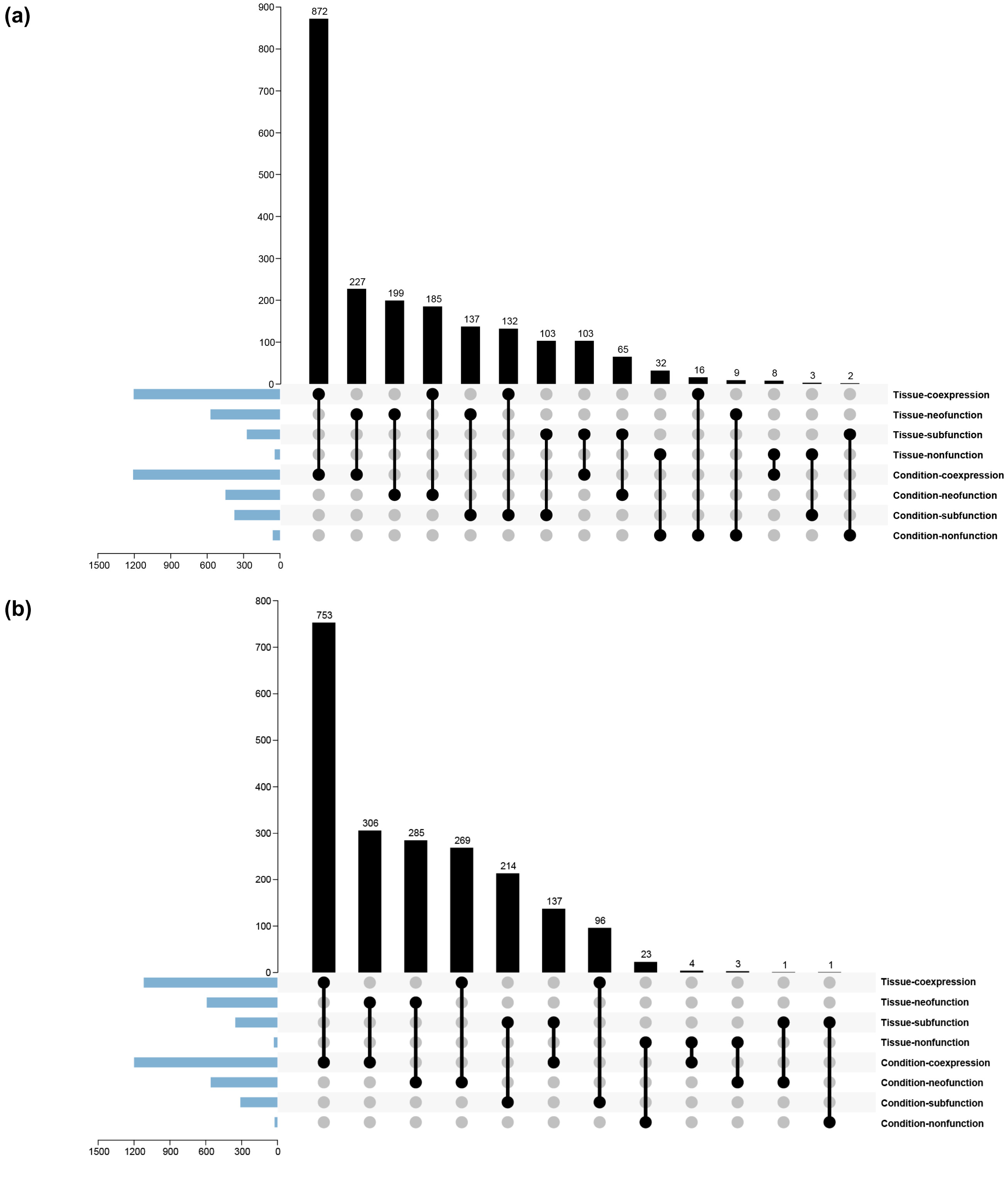

Figure (a) and (b) show the overlapping numbers among four functionalization types across tissues and conditions in the common carp and goldfish, respectively.

Supplementary Fig. 73. Genome-wide distribution of common carp DEGs in different experiments comparisons

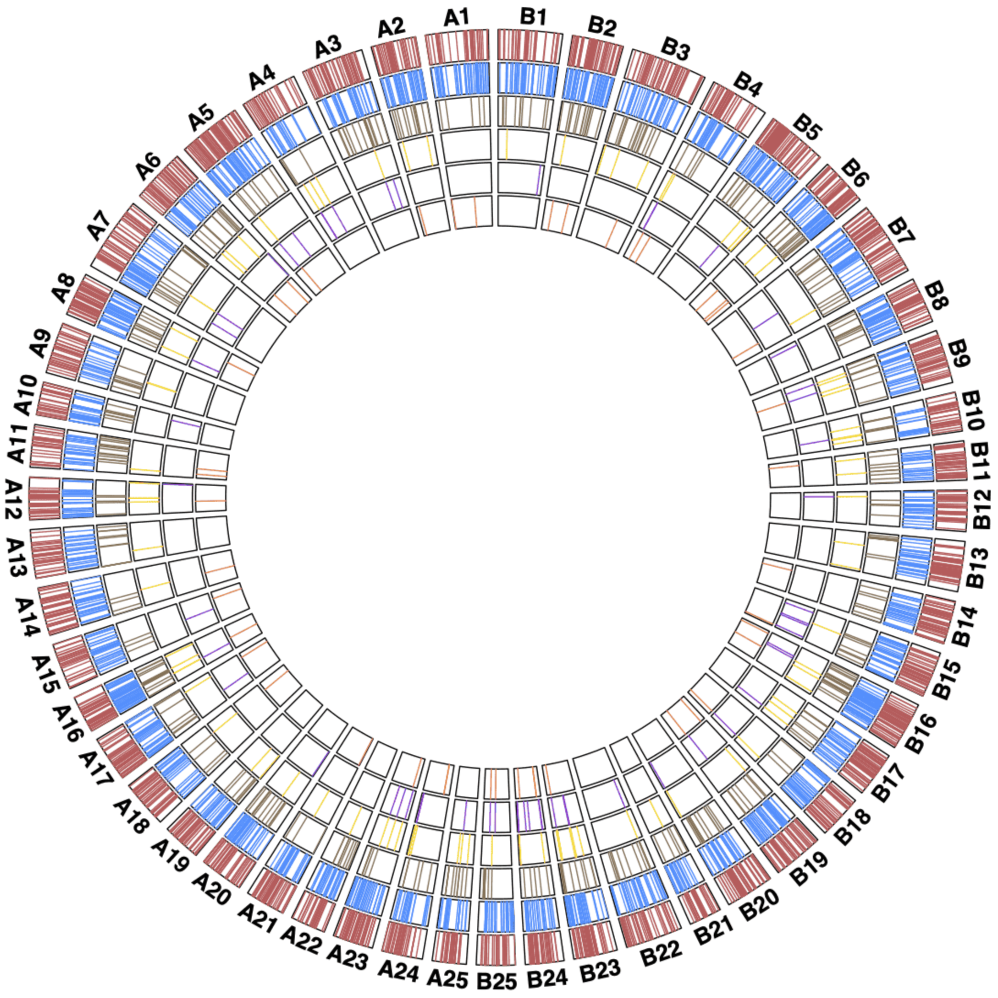

Track 1 (from the outer ring): the DEGs identified by comparing of CyHV3 infection group with control. Track 2: the DEGs identified by comparing *A. hydrophila* infection group and control. Track 3: the DEGs identified by comparing hypoxia treatment group with control. Tracks 4, 5, and 6: the DEGs identified by comparing black skin with red skin, comparing black skin with white skin, and comparing red skin with white skin, respectively.

Supplementary Fig. 74. Genome-wide distribution of goldfish DEGs in different condition comparisons

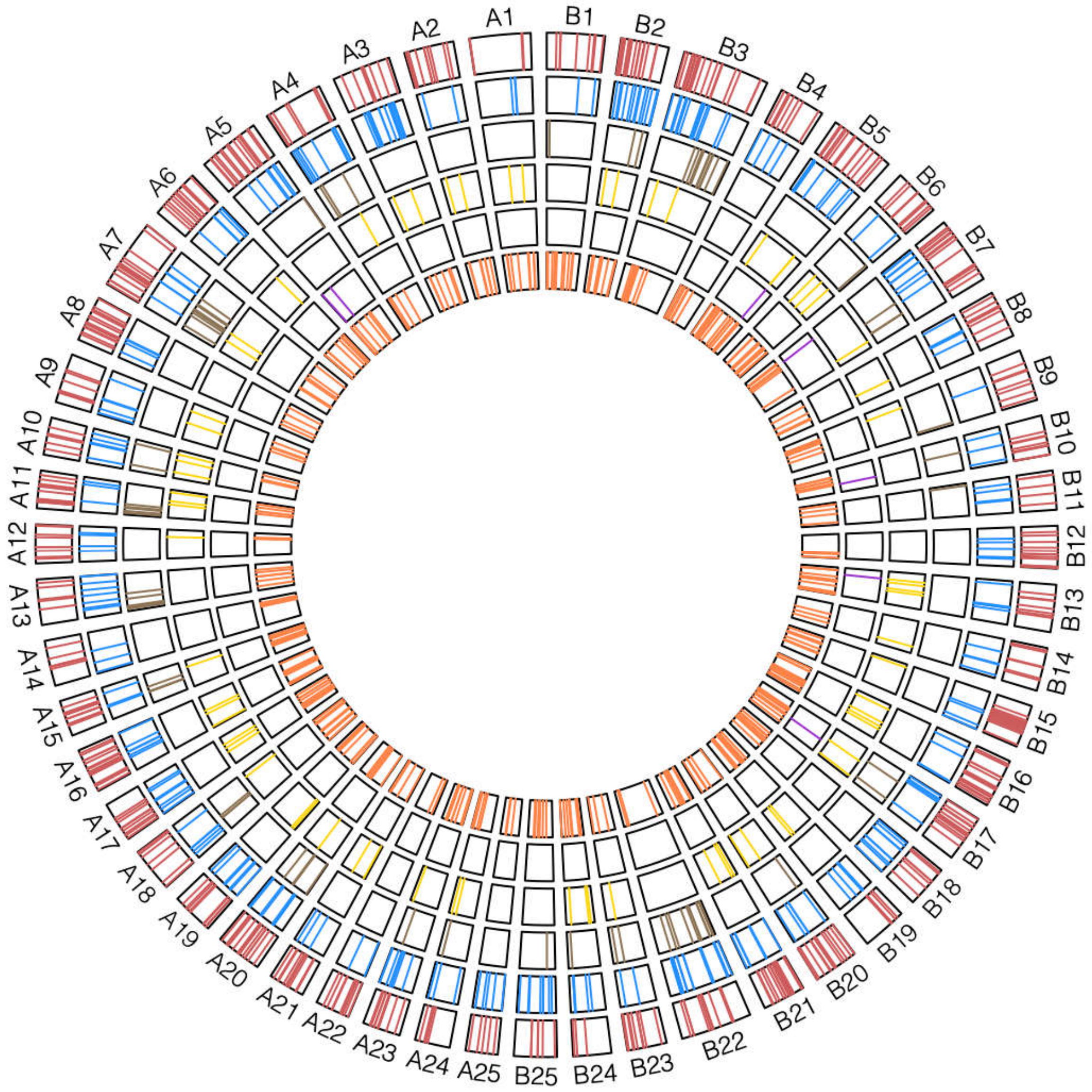

Track 1 (from the outer ring): the DEGs identified by comparing of gyrodactylus infection group with control. Track 2: the DEGs identified by comparing bisphenol treatment group and control. Track 3: the DEGs identified by comparing high FCE group with low FCE group. Tracks 4, 5, and 6: the DEGs identified by comparing control with SNa treatment, comparing control and SKF treatment, and comparing SNa treatment with SKF treatment, respectively.

Supplementary Fig. 75. GO enrichment of common carp DEGs in hypoxia treatment

(a)

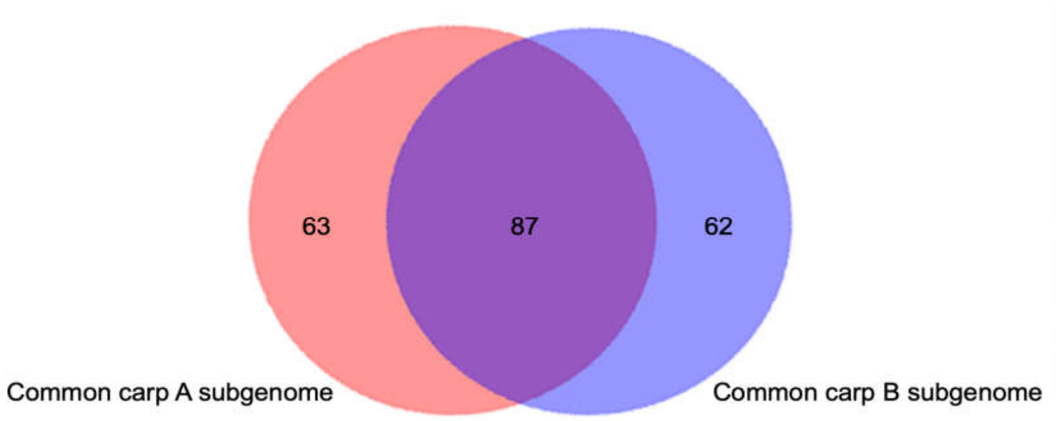

(b)

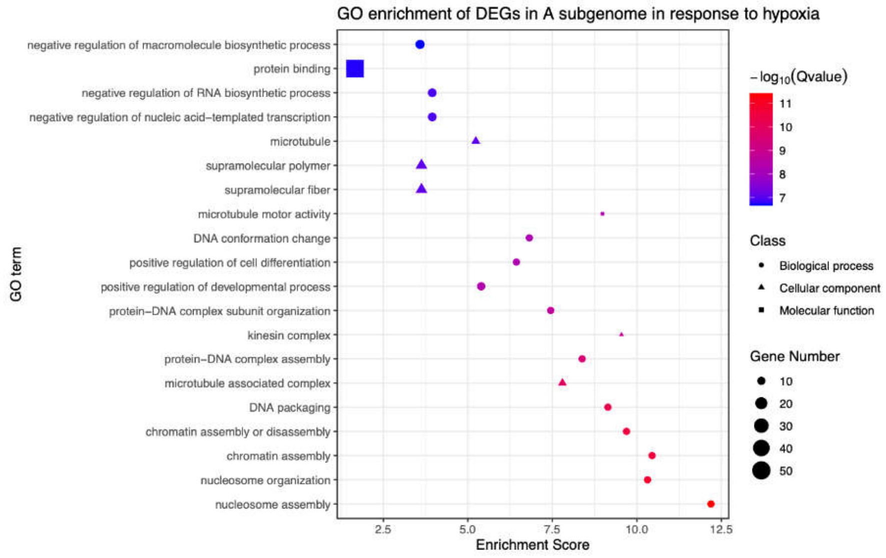

(c)

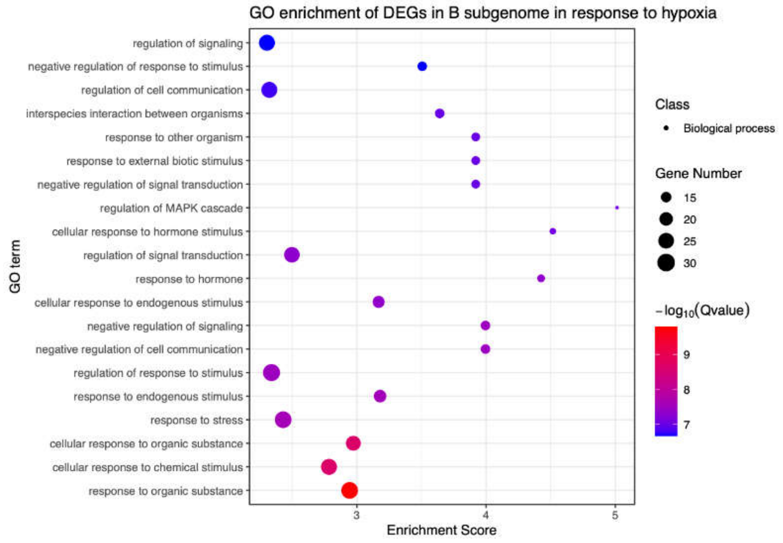

The venn diagram (a) shows the shared GO terms and specific GO terms enriched by DEGs in the common carp A and B subgenomes. The top 20 significantly enriched GO terms by DEGs in the A and B subgenome are listed in (b) and (c), respectively.

Supplementary Fig. 76. GO enrichment of common carp DEGs in CyHV-3 infection

(a)

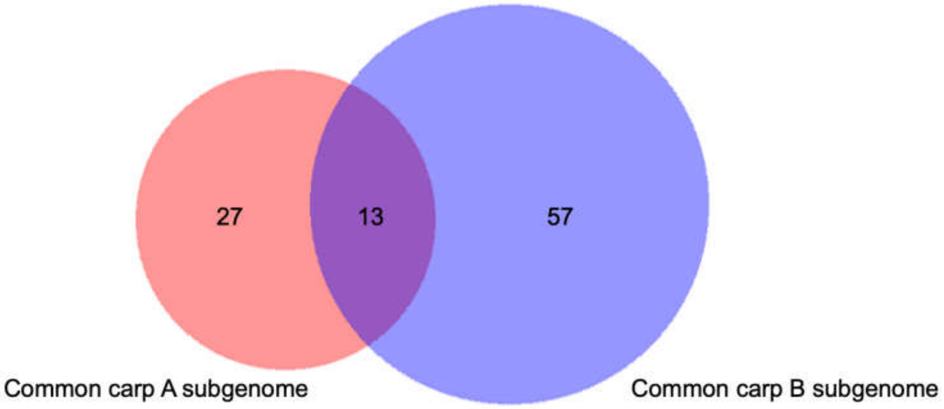

(b)

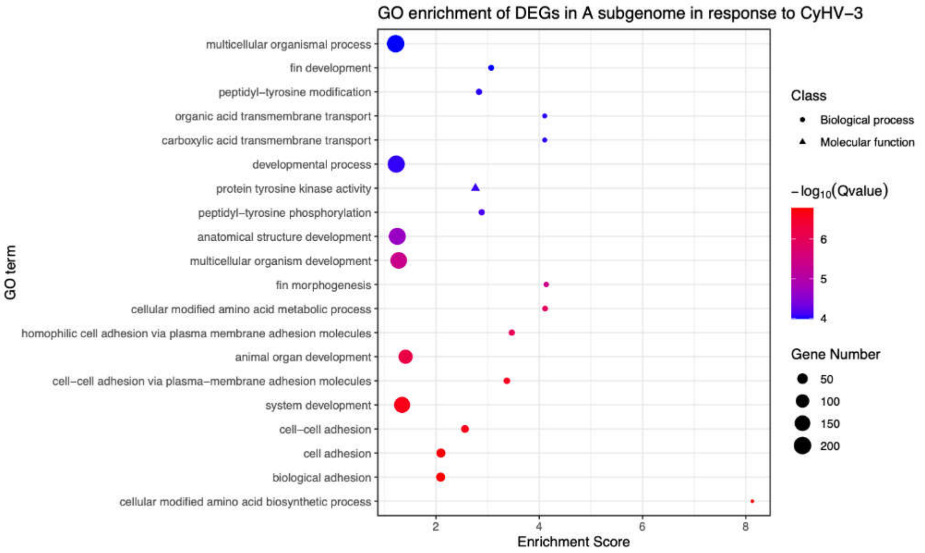

(c)

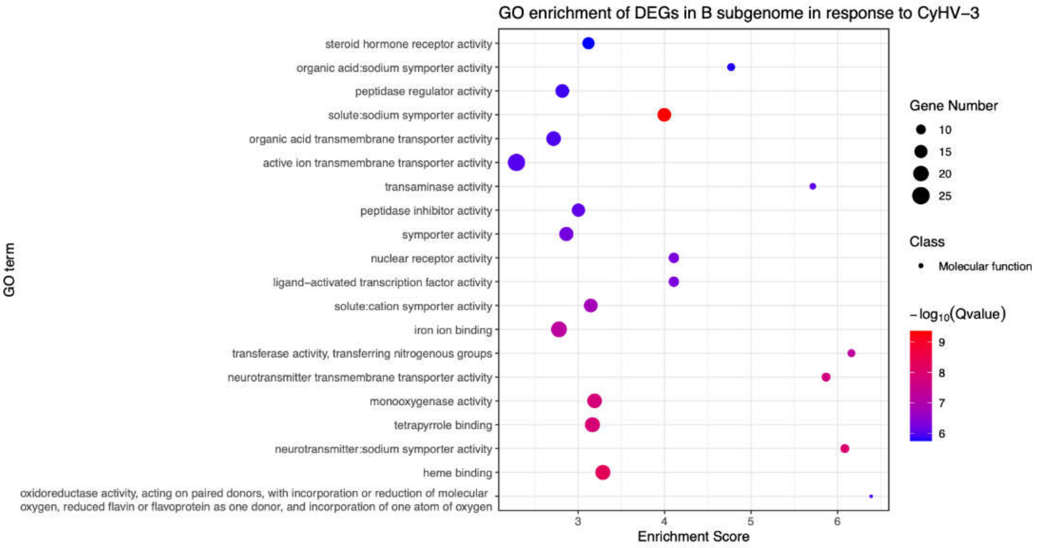

The venn diagram (a) shows the shared GO terms and specific GO terms enriched by DEGs in the common carp A and B subgenomes. The top 20 significantly enriched GO terms by DEGs in the A and B subgenome are listed in (b) and (c), respectively.

Supplementary Fig. 77. GO enrichment of common carp DEGs in *A. hydrophila* infection

(a)

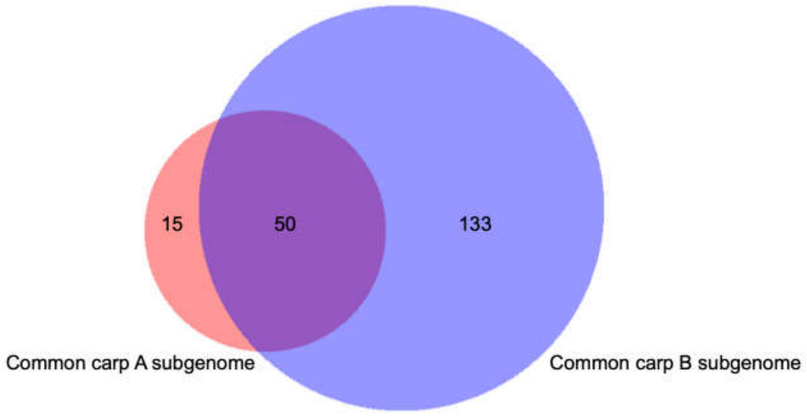

(b)

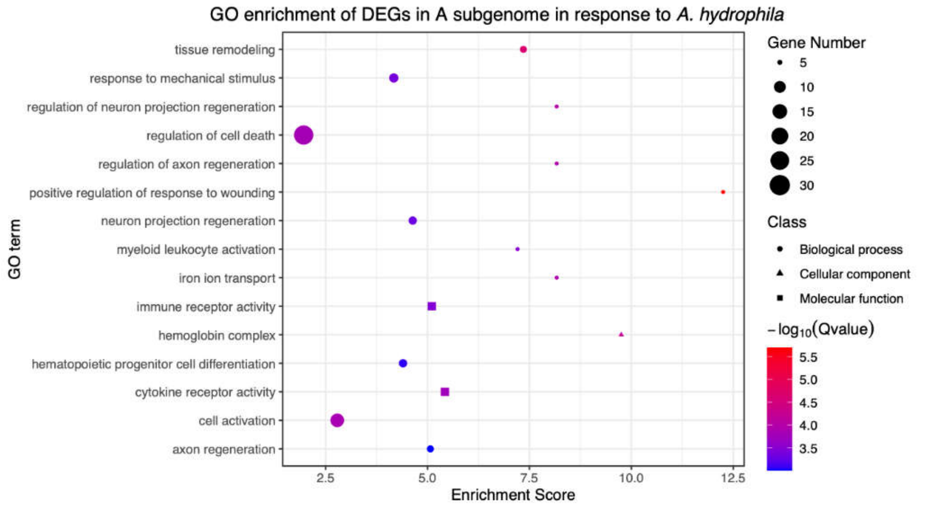

(c)

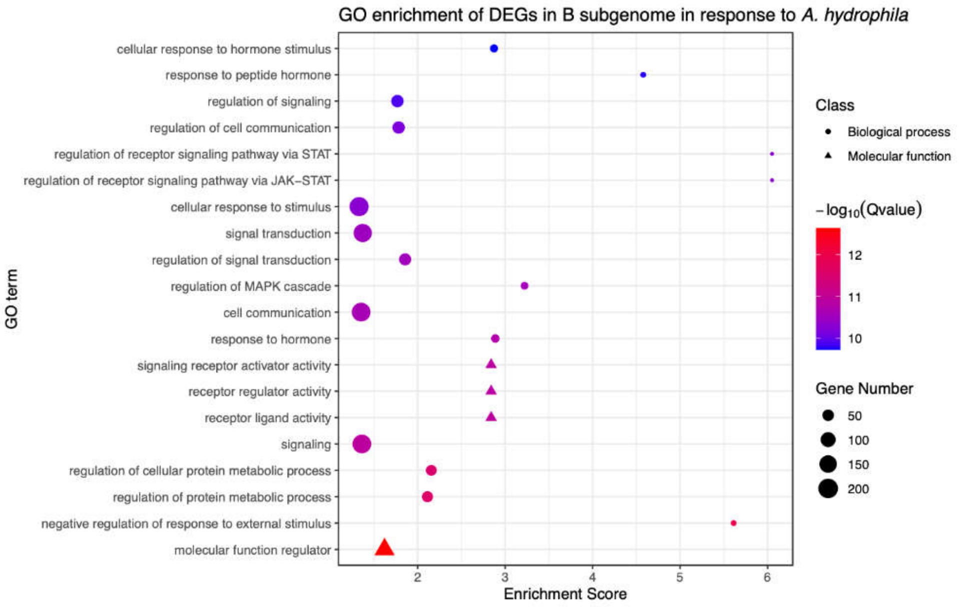

The venn diagram (a) shows the shared GO terms and specific GO terms enriched by DEGs in the common carp A and B subgenomes. The top significantly enriched GO terms by DEGs in the A and B subgenome are listed in (b) and (c), respectively.

Supplementary Fig. 78. GO enrichment of common carp DEGs in comparison of red skin and white skin

(a)

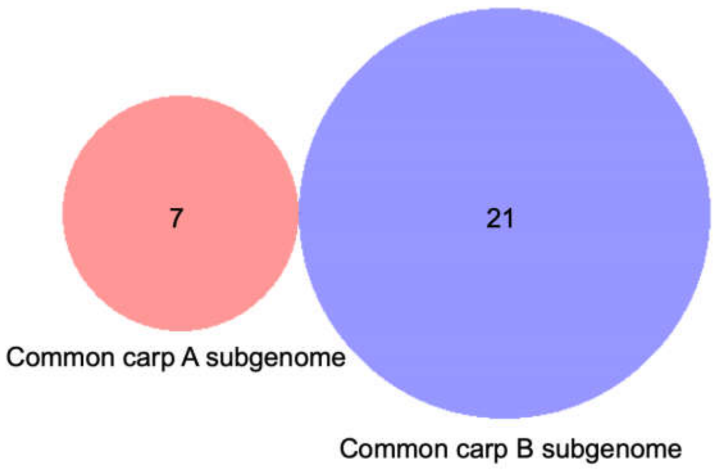

(b)

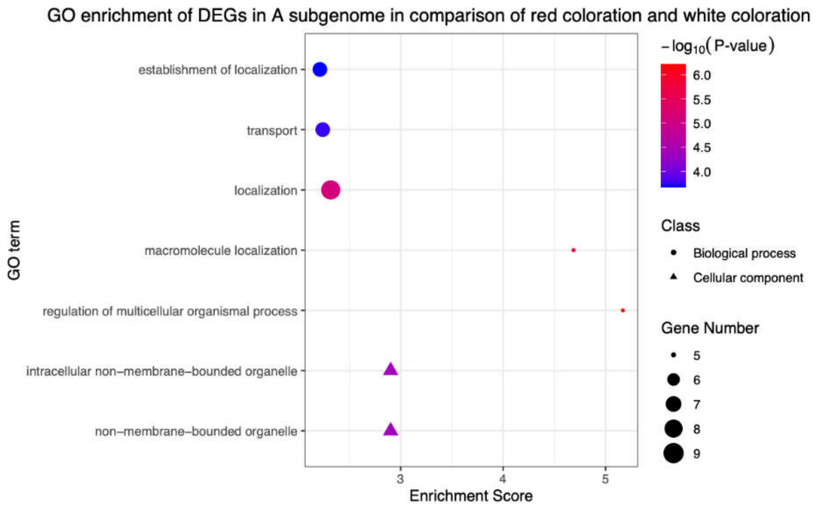

(c)

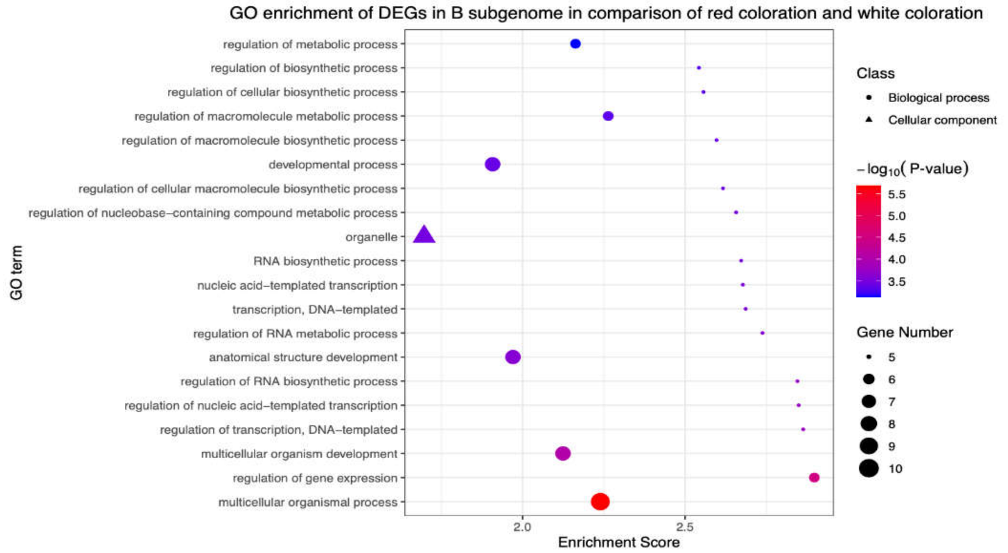

The venn diagram (a) shows the specific GO terms enriched by DEGs in the common carp A and B subgenomes.

The top significantly enriched GO terms by DEGs in the A and B subgenome are listed in (b) and (c), respectively.

Supplementary Fig. 79. GO enrichment of common carp DEGs in the other two skin comparisons

(a)

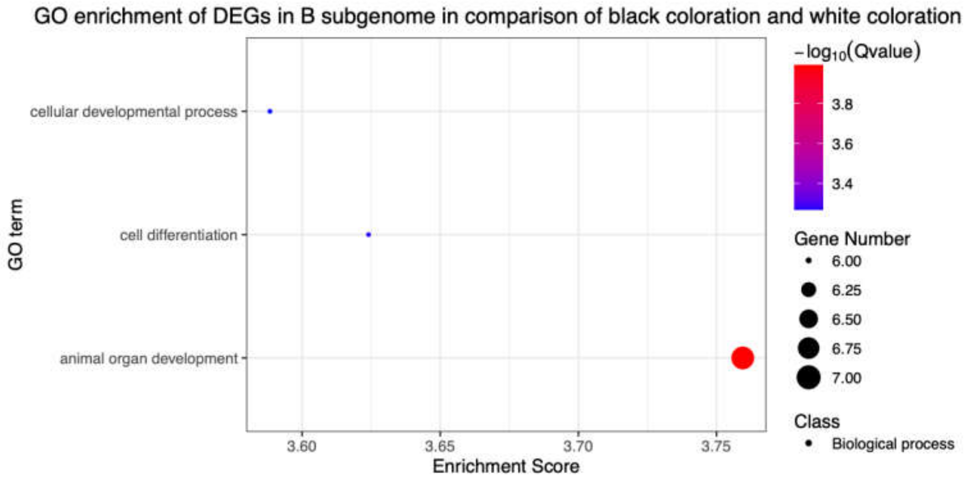

(b)

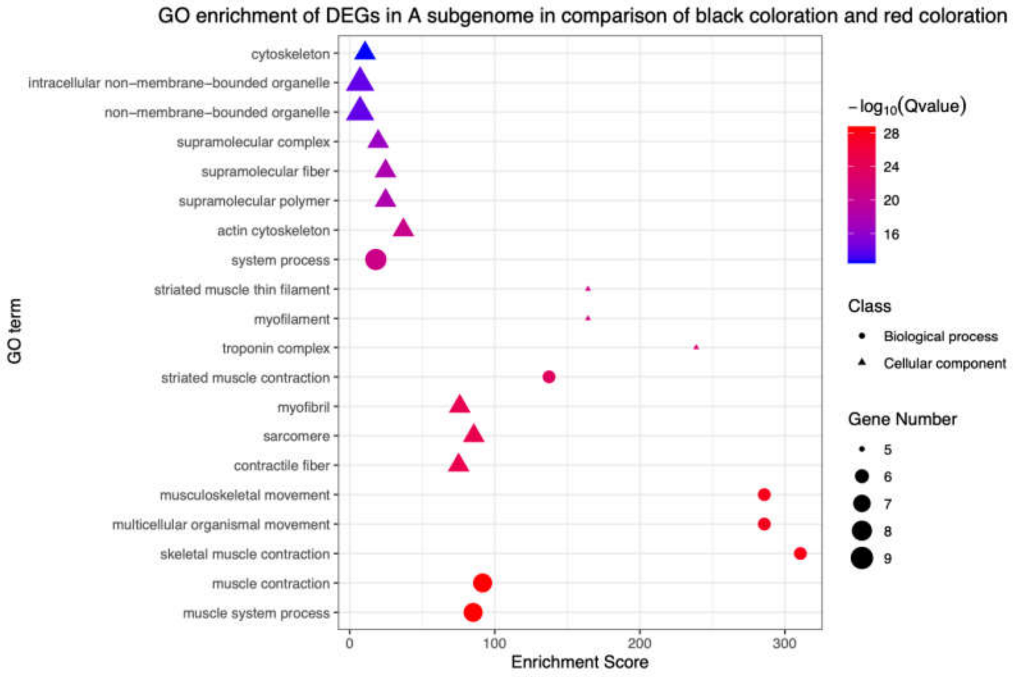

In comparison of black skin and white skin, only DEGs in the B subgenome had enriched GO terms (a). In comparison of black coloration and red coloration, only DEGs in the A subgenome had enriched GO terms (b).

Supplementary Fig. 80. GO enrichment of goldfish DEGs in bisphenol treatment

(a)

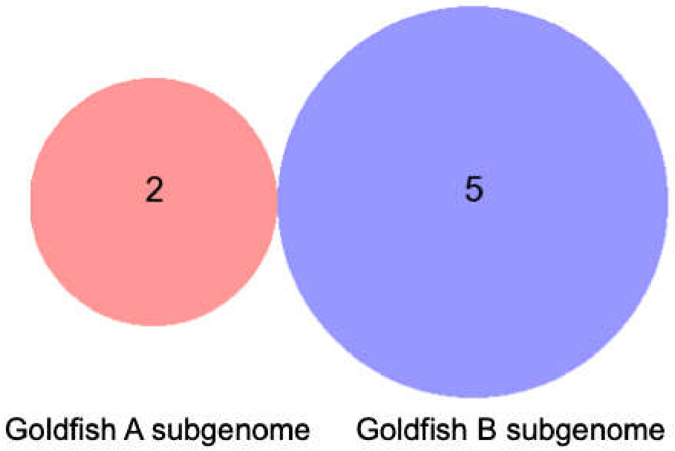

(b)

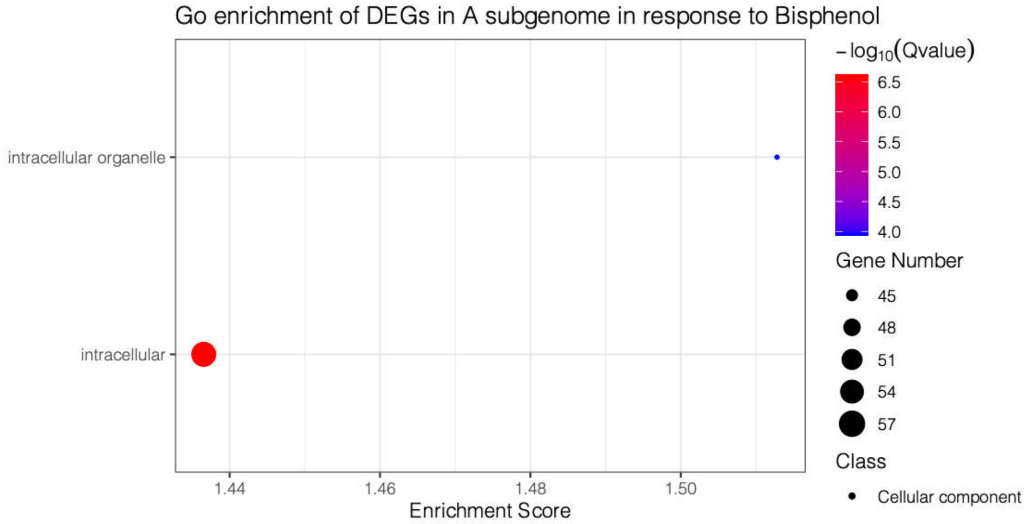

(c)

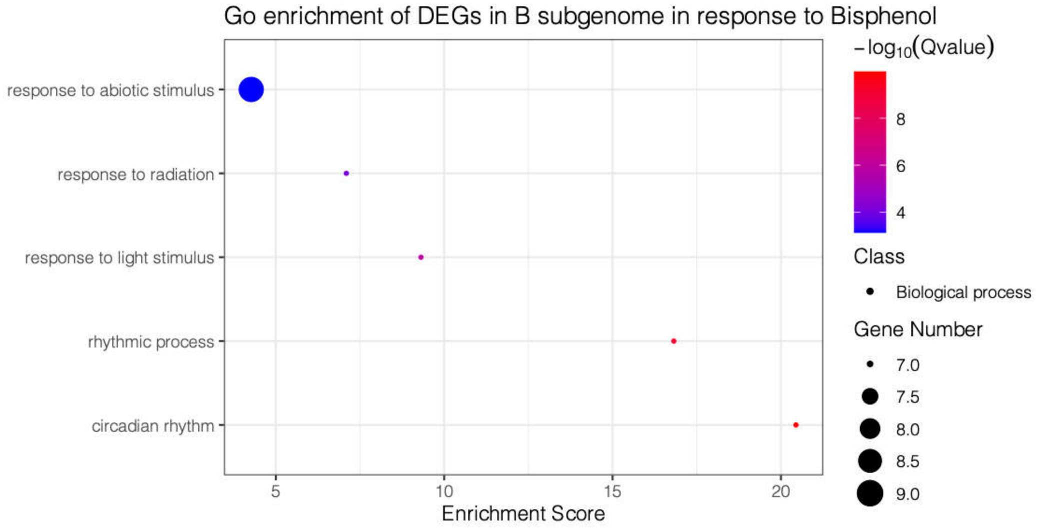

The venn diagram (a) shows the shared GO terms and specific GO terms enriched by DEGs in the goldfish A and B subgenomes. The significantly enriched GO terms by DEGs in the A and B subgenomes are listed in (b) and (c), respectively.

Supplementary Fig. 81. GO enrichment of goldfish DEGs in gyrodactylus infection

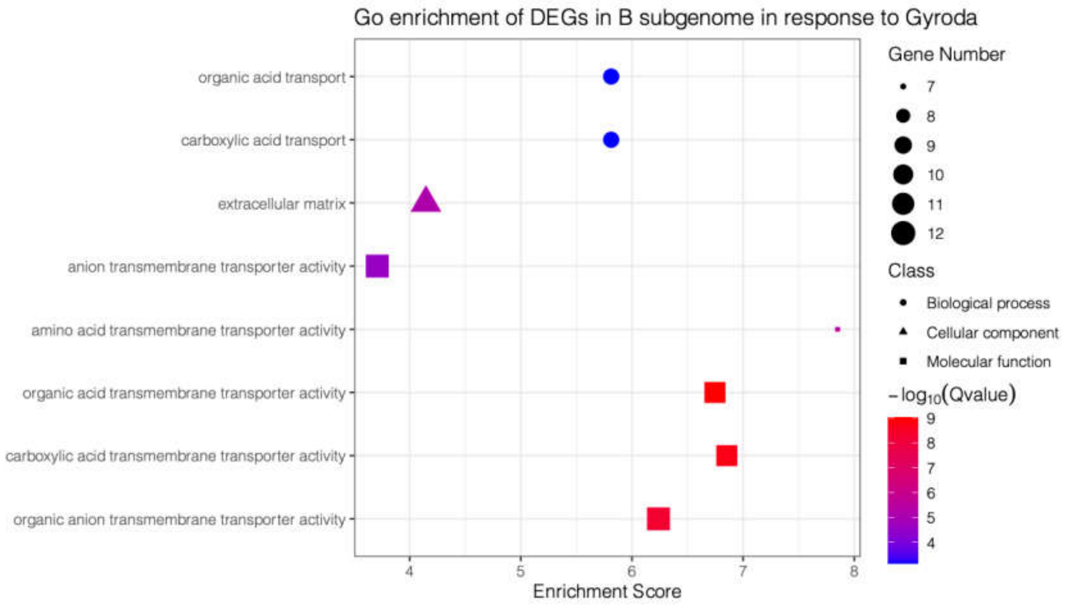

In comparison of gyrodactylus infected group and control group, only DEGs in the goldfish B subgenome had enriched GO terms.

Supplementary Fig. 82. GO enrichment of goldfish DEGs in different FCE groups

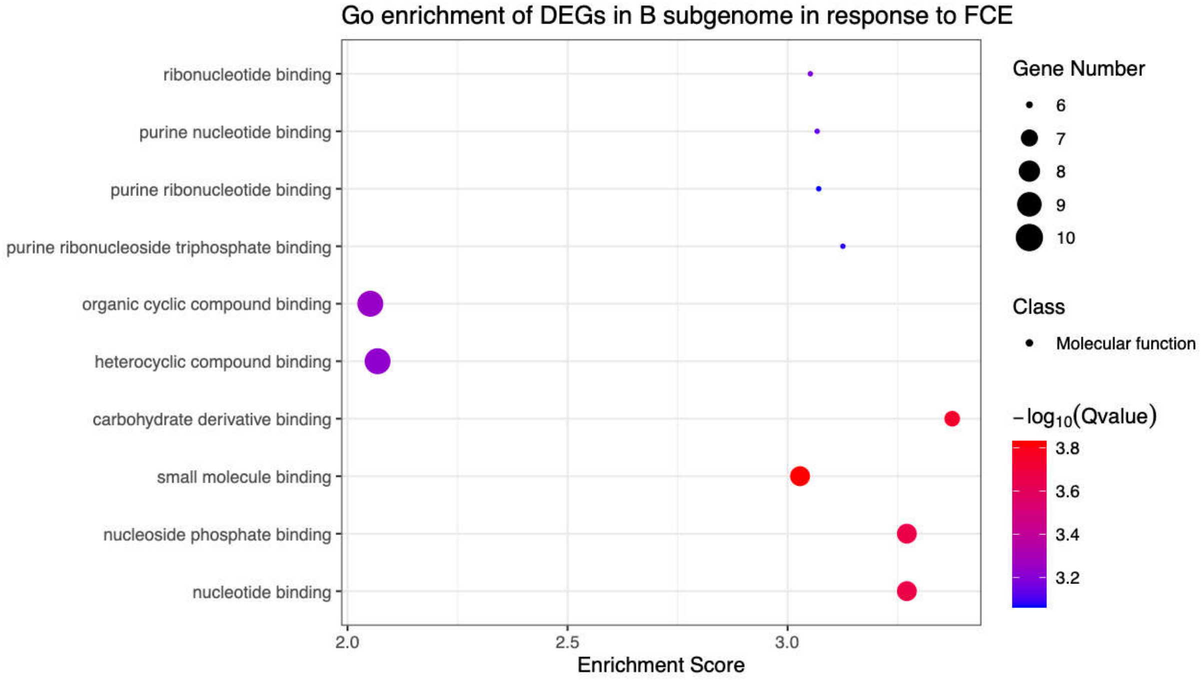

In comparison of different feed conversion efficiency (FCE) groups, only DEGs in the goldfish B subgenome had enriched GO terms.

Supplementary Fig. 83. GO enrichment of goldfish DEGs in SNa treatment group

(a)

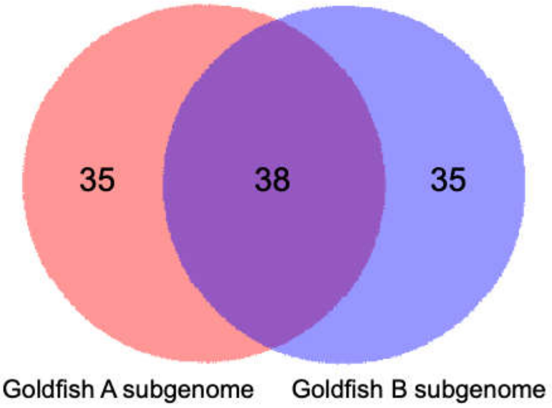

(b)

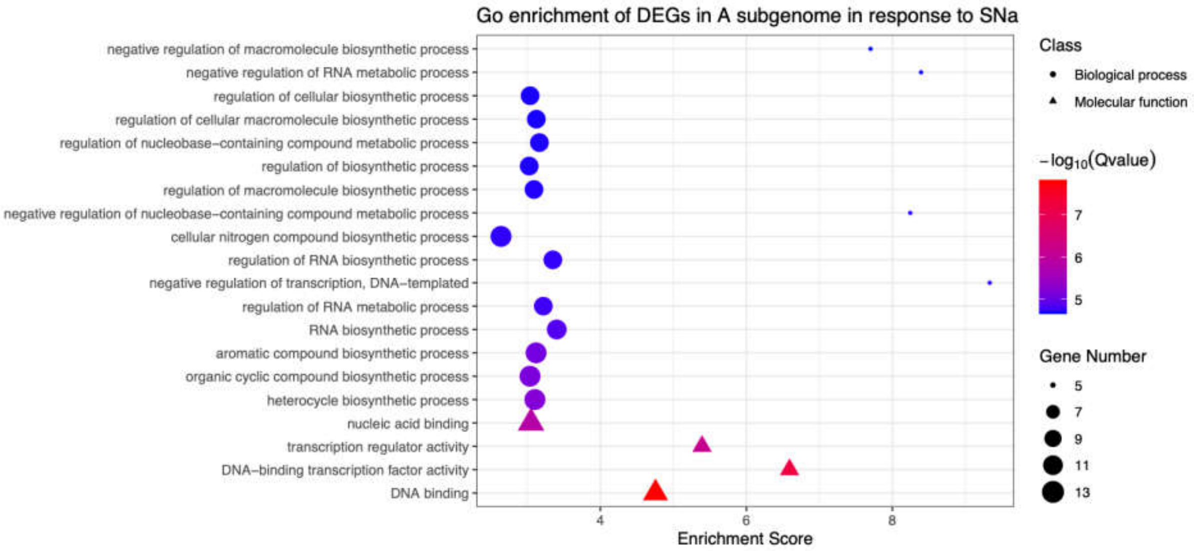

(c)

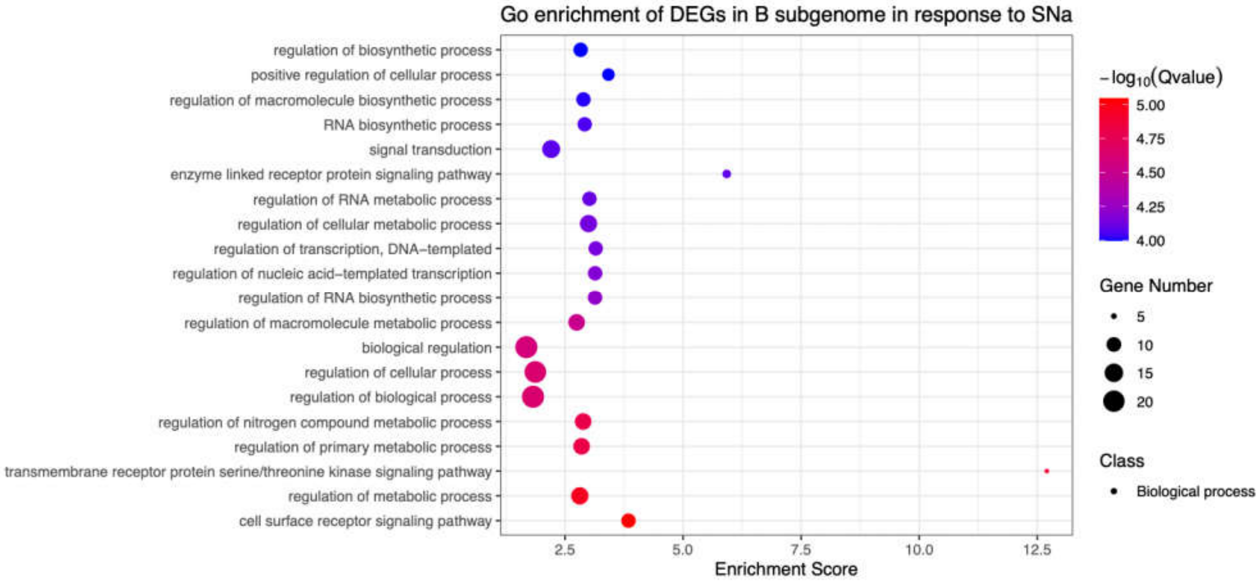

The venn diagram (a) shows the specific GO terms enriched by DEGs in the goldfish A and B subgenomes. The top 20 significantly enriched GO terms by DEGs in the A and B subgenome are listed in (b) and (c), respectively.

Supplementary Fig. 84. GO enrichment of goldfish DEGs between SNa treatment group and SKF treatment group

(a)

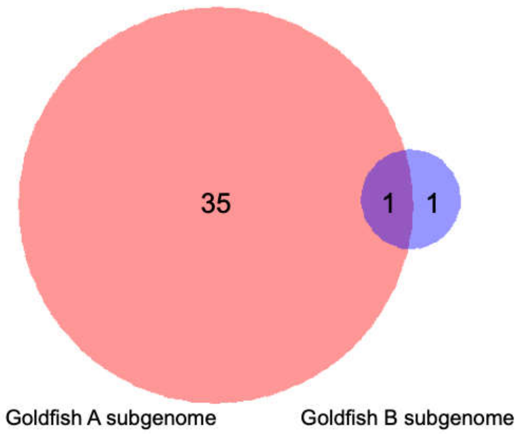

(b)

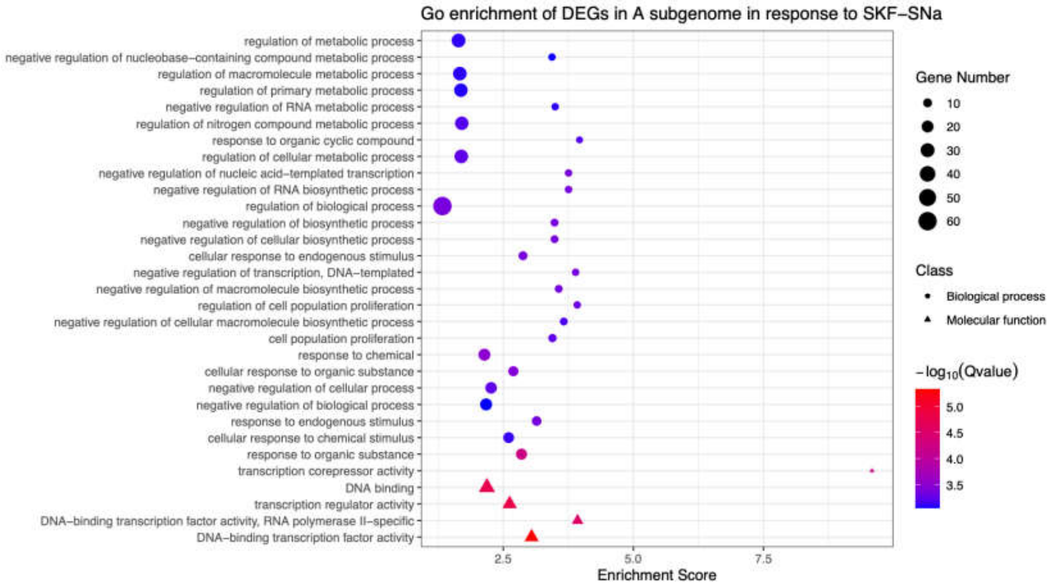

(c)

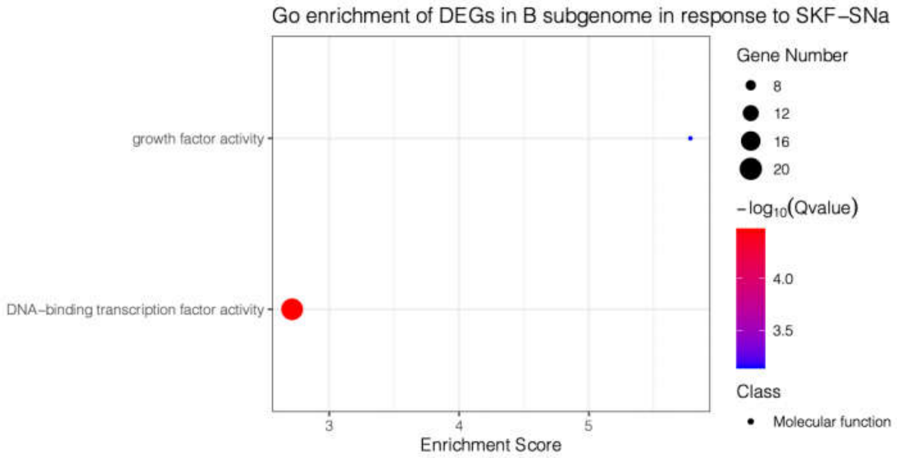

The venn diagram (a) shows the shared GO terms and specific GO terms enriched by DEGs in the goldfish A and B subgenomes. The significantly enriched GO terms by DEGs in the A and B subgenome are listed in (b) and (c), respectively.

Supplementary Fig. 85. GO enrichment of the PSGs of two common carp strains identified by using π and ZF$_{ST}$

(a)

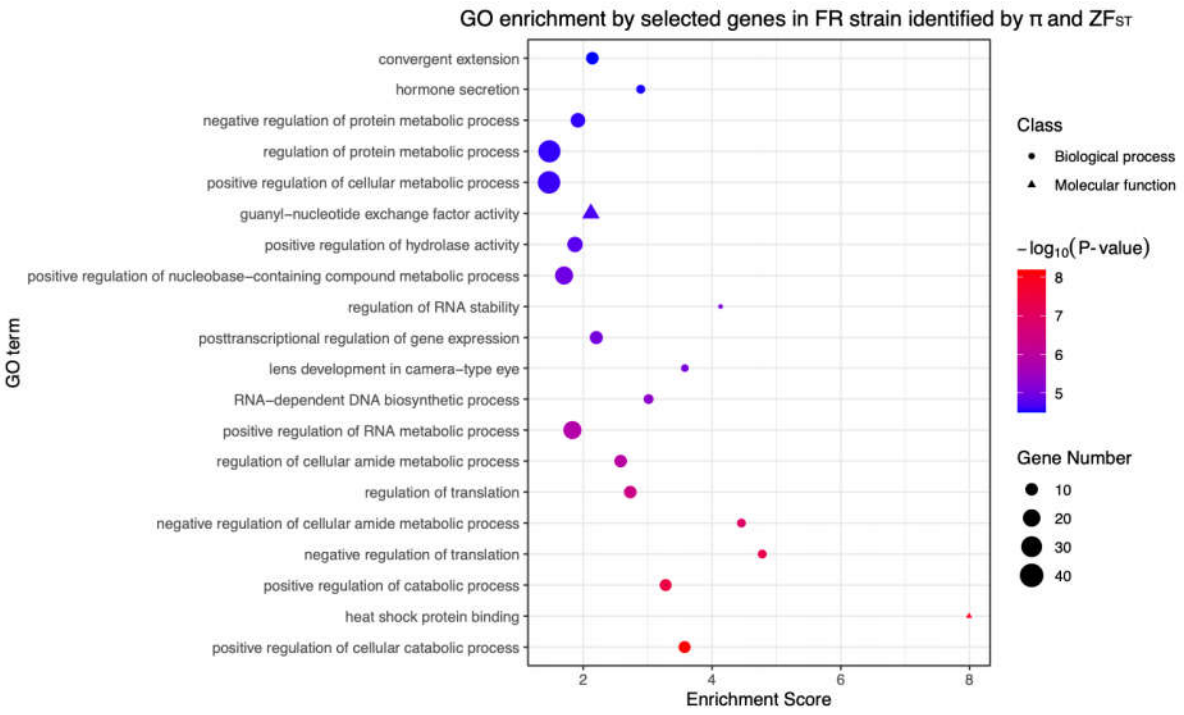

(b)

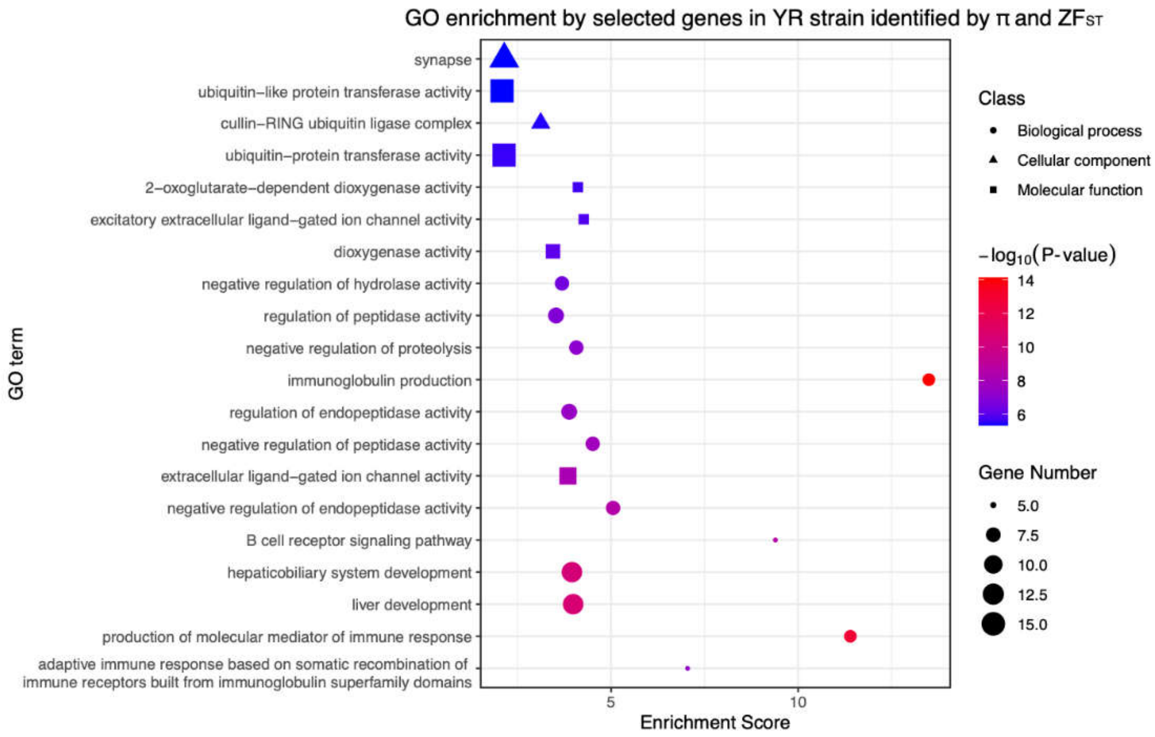

The top significantly enriched GO terms by the PSGs in the FR strain and in YR strain are listed in (a) and (b), respectively.

Supplementary Fig. 86. The re-sequencing mapping ratio, depth, and coverage of three common carp trains

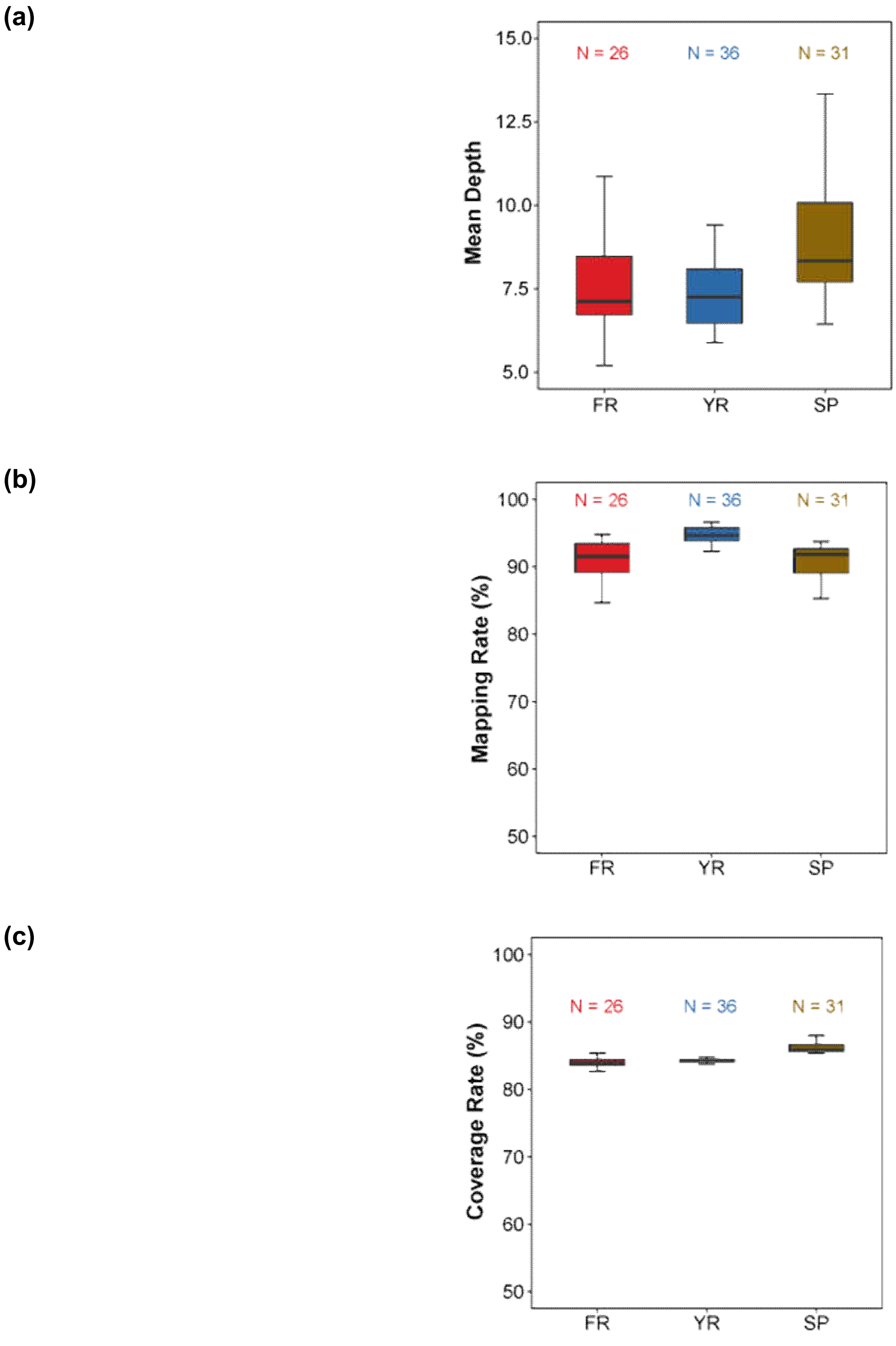

The definitions of the boxplots and whiskers are consistent with those in Fig. 3a. The N values represent the re-sequenced individual numbers of three common carp strains.

Supplementary Fig. 87. The venn diagrams of SNPs/Indels identified in three strains

(a)

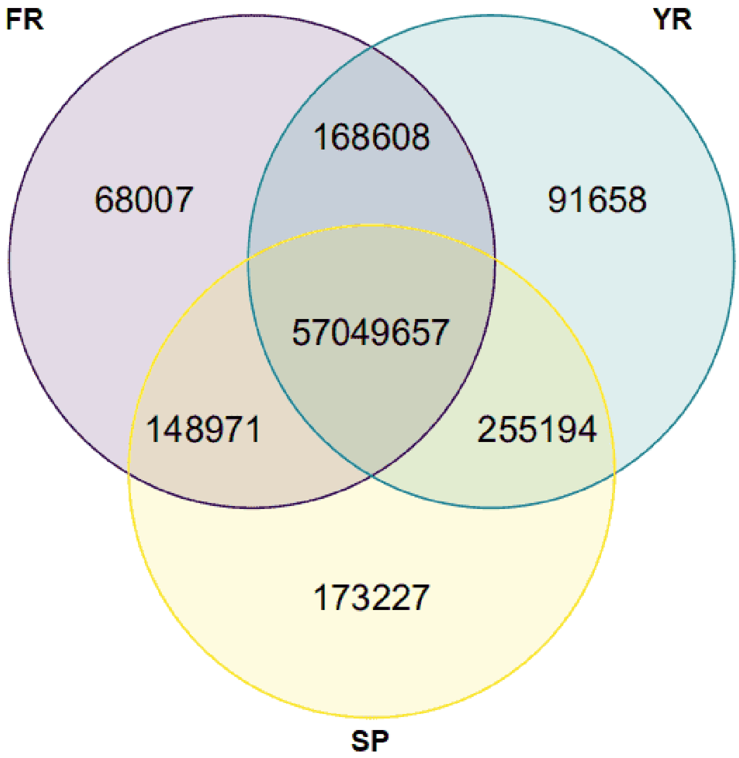

(b)

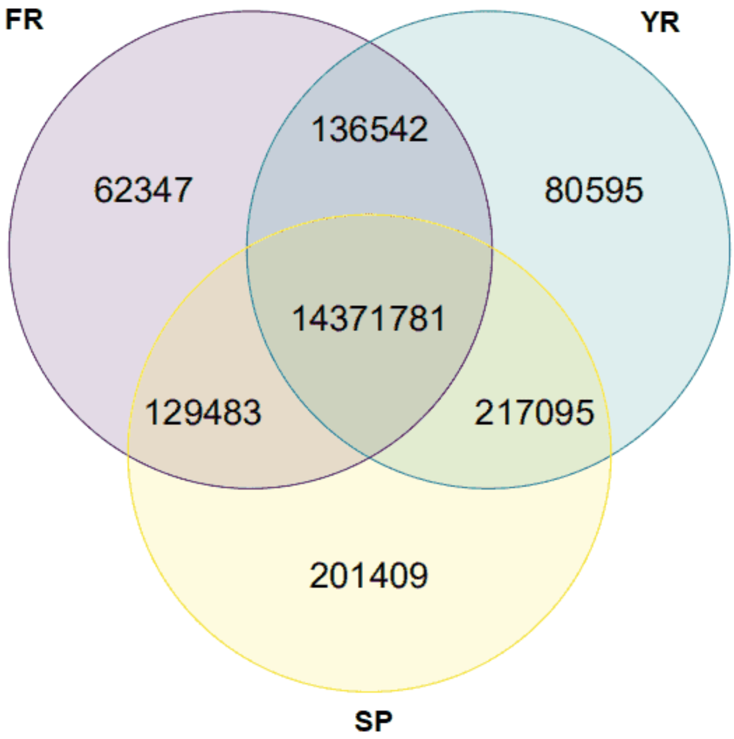

The venn diagrams of SNPs (a) and Indels (b) identified in FR, YR and SP strains.

Supplementary Fig. 88. SNP patterns in the common carp genome

(a)

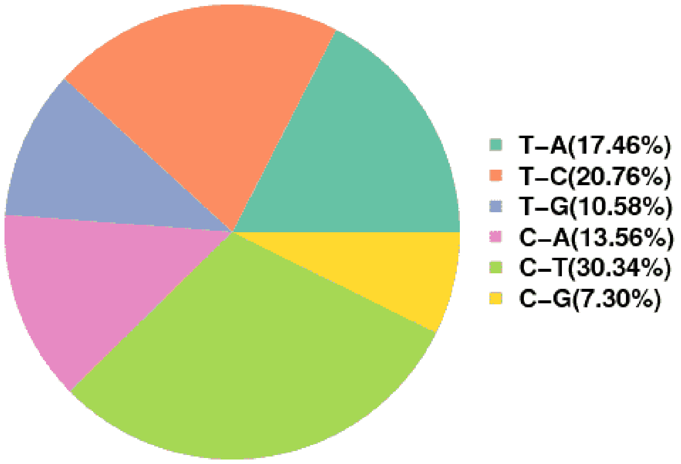

(b)

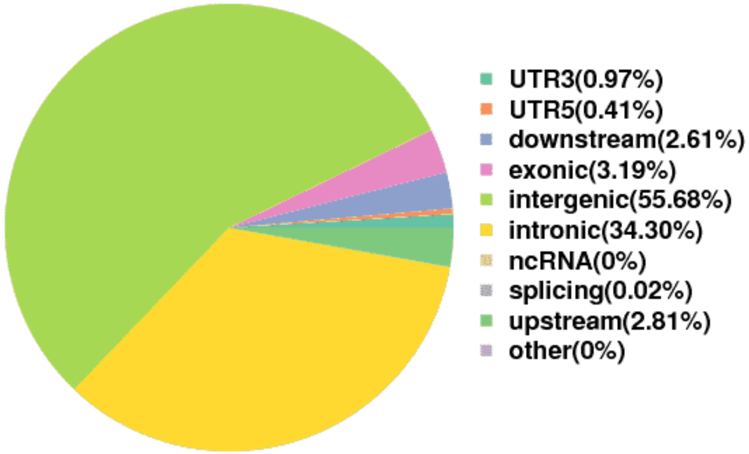

(c)

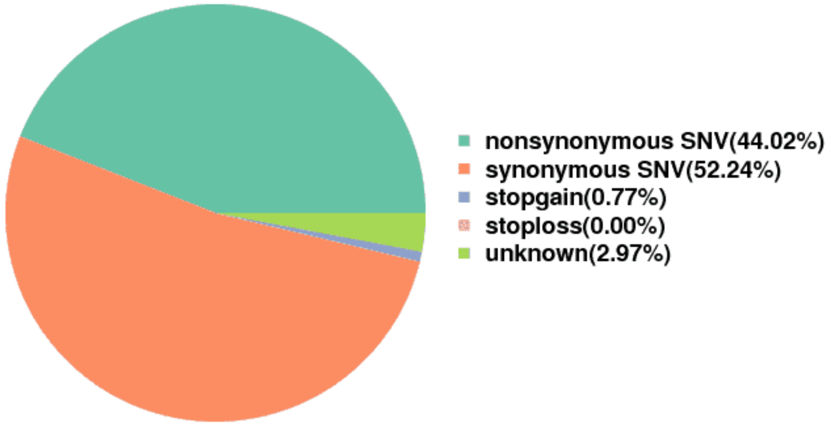

The figures (a) (b) and (c) show the proportions of different types of SNPs, genomic location distribution and functional annotation of SNPs.

Supplementary Fig. 89. Indels distribution in the common carp genome

(a)

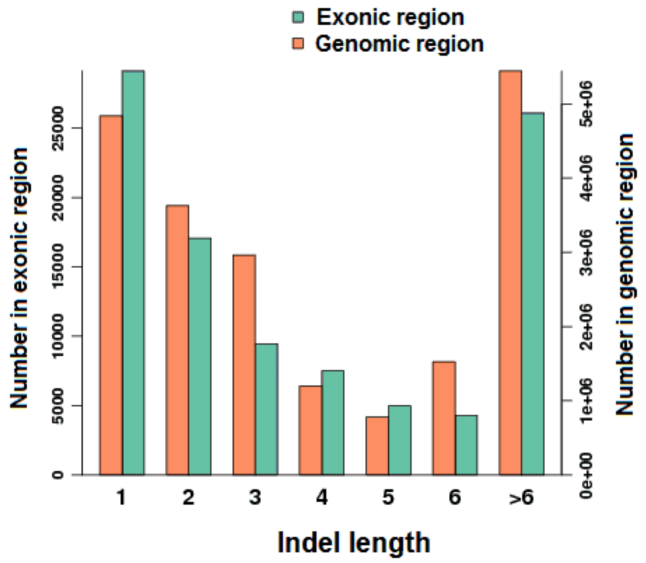

(b)

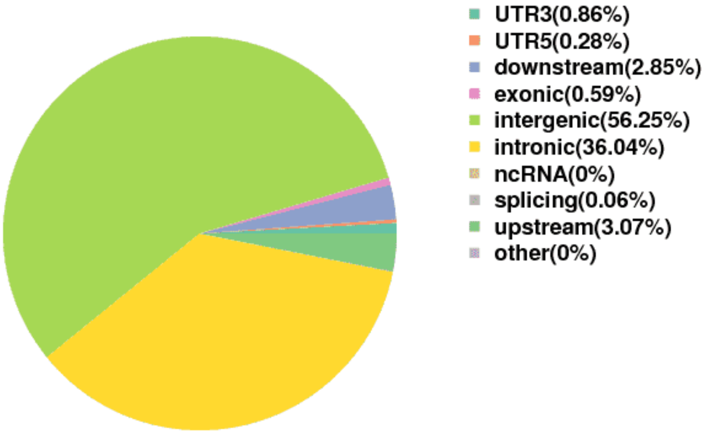

(c)

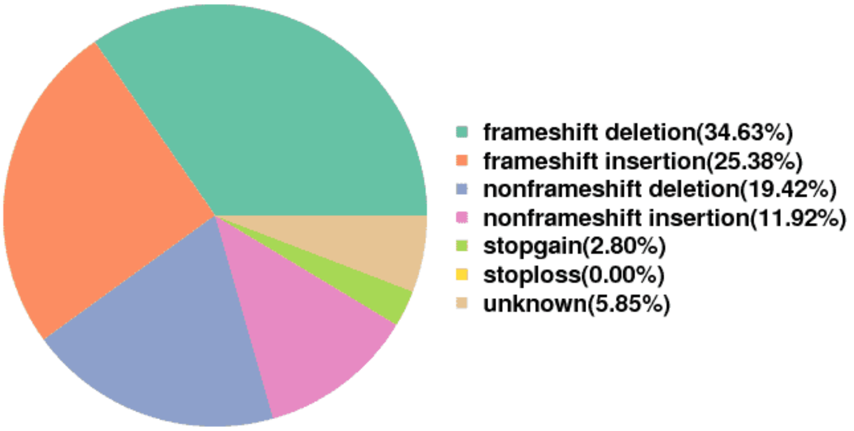

The figures (a) (b) and (c) show the proportions of different types of Indels, genomic location distribution and functional annotation of Indels.

References

1. Marçais, G. & Kingsford, C. A fast, lock-free approach for efficient parallel counting of occurrences of k-mers. *Bioinformatics* **27**, 764-770 (2011).
2. Ruan, J. & Li, H. Fast and accurate long-read assembly with wtdbg2. *Nature Methods* **17**, 155-158 (2020).
3. Kajitani, R. *et al.* Efficient de novo assembly of highly heterozygous genomes from whole-genome shotgun short reads. *Genome Research* **24**, 1384-1395 (2014).
4. Vaser, R., Sovic, I., Nagarajan, N. & Sikic, M. Fast and accurate de novo genome assembly from long uncorrected reads. *Genome Research* **27**, 737-746 (2017).
5. Walker, B.J. *et al.* Pilon: an integrated tool for comprehensive microbial variant detection and genome assembly improvement. *PloS one* **9**, e112963-e112963 (2014).
6. Chakraborty, M., Baldwin-Brown, J.G., Long, A.D. & Emerson, J.J. Contiguous and accurate de novo assembly of metazoan genomes with modest long read coverage. *Nucleic Acids Research* **44**, e147-e147 (2016).
7. Langmead, B. & Salzberg, S.L. Fast gapped-read alignment with Bowtie 2. *Nature Methods* **9**, 357 (2012).
8. Wingett, S. *et al.* HiCUP: pipeline for mapping and processing Hi-C data. *F1000Research* **4**, 1310-1310 (2015).
9. Burton, J.N. *et al.* Chromosome-scale scaffolding of de novo genome assemblies based on chromatin interactions. *Nature Biotechnology* **31**, 1119 (2013).
10. Cui, W. *et al.* Embryonic development and phylogenetic analysis of Puntius tetrazona. *Journal of fisheries of China* **44**, 1286-1295 (2020).
11. Gu, R., Xu, G., Wen, H. & Hua, D. Karyotypic analysis and cellular DNA contents of Paracanthobrama guichenoti in Lake Taihu. *Journal of Fisheries of China* **33**, 9-14 (2009).
12. Xu, P. *et al.* Genome sequence and genetic diversity of the common carp, *Cyprinus carpio*. *Nature Genetics* **46**, 1212-1219 (2014).
13. Kolder, I.C. *et al.* A full-body transcriptome and proteome resource for the European common carp. *BMC Genomics* **17**, 701 (2016).
14. Xu, P. *et al.* The allotetraploid origin and asymmetrical genome evolution of the common carp Cyprinus carpio. *Nature Communications* **10**, 4625 (2019).
15. Jain, C., Koren, S., Dilthey, A., Phillippy, A.M. & Aluru, S. A fast adaptive algorithm for computing whole-genome homology maps. *Bioinformatics* **34**, i748-i756 (2018).
16. Xu, P. *et al.* Genomic insight into the common carp (Cyprinus carpio) genome by sequencing analysis of BAC-end sequences. *BMC Genomics* **12**, 188 (2011).
17. Kent, W.J. BLAT--the BLAST-like alignment tool. *Genome Research* **12**, 656-664 (2002).
18. Kim, D., Langmead, B. & Salzberg, S.L. HISAT: a fast spliced aligner with low memory requirements. *Nature methods* **12**, 357-360 (2015).
19. Chen, D. *et al.* The evolutionary origin and domestication history of goldfish (Carassius auratus). *Proceedings of the National Academy of Sciences* **117**, 29775 (2020).
20. Wu, T.D. & Watanabe, C.K. GMAP: a genomic mapping and alignment program for mRNA and EST sequences. *Bioinformatics* **21**, 1859-1875 (2005).
21. Bolger, A.M., Lohse, M. & Usadel, B. Trimmomatic: a flexible trimmer for Illumina sequence data. *Bioinformatics* **30**, 2114-2120 (2014).
22. Cox, M.P., Peterson, D.A. & Biggs, P.J. SolexaQA: At-a-glance quality assessment of Illumina second-generation sequencing data. *BMC bioinformatics* **11**, 485 (2010).
23. Pertea, M. *et al.* StringTie enables improved reconstruction of a transcriptome from RNA-seq reads. *Nature Biotechnology* **33**, 290-295 (2015).
24. Li, H. & Durbin, R. Fast and accurate short read alignment with Burrows-Wheeler transform. *Bioinformatics* **25**, 1754-1760 (2009).
25. David, L., Blum, S., Feldman, M.W., Lavi, U. & Hillel, J. Recent Duplication of the Common Carp (Cyprinus carpio L.) Genome as Revealed by Analyses of Microsatellite Loci. *Molecular Biology and Evolution* **20**, 1425-1434 (2003).
26. Kumar, S., Stecher, G., Suleski, M. & Hedges, S.B. TimeTree: A Resource for Timelines, Timetrees, and Divergence Times. *Molecular Biology and Evolution* **34**, 1812-1819 (2017).
27. Session, A.M. *et al.* Genome evolution in the allotetraploid frog Xenopus laevis. *Nature* **538**, 336-343 (2016).
28. Chen, Z. *et al.* De novo assembly of the goldfish (Carassius auratus) genome and the evolution of genes after whole-genome duplication. *Science Advances* **5**, eaav0547 (2019).
29. Haas, B.J. *et al.* De novo transcript sequence reconstruction from RNA-seq using the Trinity platform for reference generation and analysis. *Nature Protocols* **8**, 1494-512 (2013).
30. Wang, Y. *et al.* MCScanX: a toolkit for detection and evolutionary analysis of gene synteny and collinearity. *Nucleic acids research* **40**, e49-e49 (2012).
31. Langham, R.J. *et al.* Genomic duplication, fractionation and the origin of regulatory novelty. *Genetics* **166**, 935-945

(2004).
32. Force, A. *et al.* Preservation of duplicate genes by complementary, degenerative mutations. *Genetics* **151**, 1531-1545 (1999).
33. Teshima, K.M. & Innan, H. Neofunctionalization of duplicated genes under the pressure of gene conversion. *Genetics* **178**, 1385-1398 (2008).
34. Kon, T. *et al.* The Genetic Basis of Morphological Diversity in Domesticated Goldfish. *Current Biology* **30**, 2260-2274.e6 (2020).
35. Howe, K. *et al.* The zebrafish reference genome sequence and its relationship to the human genome. *Nature* **496**, 498-503 (2013).
36. Anderson, J.L. *et al.* Multiple Sex-Associated Regions and a Putative Sex Chromosome in Zebrafish Revealed by RAD Mapping and Population Genomics. *PLOS ONE* **7**, e40701 (2012).
37. Dong, Z., Nguyen, N.H. & Zhu, W. Genetic evaluation of a selective breeding program for common carp Cyprinus carpio conducted from 2004 to 2014. *BMC genetics* **16**, 94-94 (2015).
38. Simakov, O. *et al.* Deeply conserved synteny resolves early events in vertebrate evolution. *Nature Ecology and Evolution* **4**, 820-830 (2020).
39. Berthelot, C. *et al.* The rainbow trout genome provides novel insights into evolution after whole-genome duplication in vertebrates. *Nature Communications* **5**, 3657 (2014).
40. Lien, S. *et al.* The Atlantic salmon genome provides insights into rediploidization. *Nature* **533**, 200-205 (2016).
41. Du, K. *et al.* The sterlet sturgeon genome sequence and the mechanisms of segmental rediploidization. *Nature Ecology and Evolution* **4**, 841-852 (2020).
42. Kurtz, S. *et al.* Versatile and open software for comparing large genomes. *Genome biology* **5**, R12-R12 (2004).
