# Supplementary material for: Parallel subgenome structure and divergent expression evolution of allo-tetraploid common carp and goldfish
Source: Nat Genet. 2021 Sep 30;53(10):1493–503. doi: 10.1038/s41588-021-00933-9 (PMC8492472; doi:10.1038/s41588-021-00933-9)
Supplement: Supplementary file 2 — Reporting Summary [file 41588_2021_933_MOESM2_ESM.pdf]

nature research

Corresponding author(s): Jiong-Tang Li

Last updated by author(s): Jun 23, 2021

# Reporting Summary

Nature Research wishes to improve the reproducibility of the work that we publish. This form provides structure for consistency and transparency in reporting. For further information on Nature Research policies, see our Editorial Policies and the Editorial Policy Checklist.

## Statistics

For all statistical analyses, confirm that the following items are present in the figure legend, table legend, main text, or Methods section.

| n/a | Confirmed | |
|---|---|---|
| ☐ | ☒ | The exact sample size (*n*) for each experimental group/condition, given as a discrete number and unit of measurement |
| ☐ | ☒ | A statement on whether measurements were taken from distinct samples or whether the same sample was measured repeatedly |
| ☐ | ☒ | The statistical test(s) used AND whether they are one- or two-sided<br>*Only common tests should be described solely by name; describe more complex techniques in the Methods section.* |
| ☒ | ☐ | A description of all covariates tested |
| ☐ | ☒ | A description of any assumptions or corrections, such as tests of normality and adjustment for multiple comparisons |
| ☐ | ☒ | A full description of the statistical parameters including central tendency (e.g. means) or other basic estimates (e.g. regression coefficient) AND variation (e.g. standard deviation) or associated estimates of uncertainty (e.g. confidence intervals) |
| ☐ | ☒ | For null hypothesis testing, the test statistic (e.g. *F*, *t*, *r*) with confidence intervals, effect sizes, degrees of freedom and *P* value noted<br>*Give P values as exact values whenever suitable.* |
| ☒ | ☐ | For Bayesian analysis, information on the choice of priors and Markov chain Monte Carlo settings |
| ☒ | ☐ | For hierarchical and complex designs, identification of the appropriate level for tests and full reporting of outcomes |
| ☐ | ☒ | Estimates of effect sizes (e.g. Cohen's *d*, Pearson's *r*), indicating how they were calculated |

*Our web collection on statistics for biologists contains articles on many of the points above.*

## Software and code

Policy information about availability of computer code

**Data collection**

PacBio reads and Nanopore data were collected on PacBio Sequel instrument and Nanopore platform, respectively. Hi-C data and pair-end reads were collected from the Illumina HiSeq platform. For common carp, the published genome-seq reads and RNA-seq data of different aquatic traits were download from NCBI SRA database.

**Data analysis**

All the softwares used for analysis have been described in the Online Methods as well as Supplementary Methods. All software used in this study included: Trimmomatic v0.35, SolexaQA v3.7.1, Jeffyfish v2.2, wtdbg2 v2.4, racon v1.3.1, pilon v1.22, quickmerge v0.3, SSPACE v3.0, Platanus v1.2.4, LR_Gapcloser v1.0, Bowtie2 v2.3.5.1, HiCUP v0.6.1, HiC-Pro v2.11.1, BLAT v35X1, Lachesis v1.0, BWA v0.7.17, Samtools v0.1.19, HISAT2 v2.1.0, HiCPlotter v0.8.1, RepeatModeler v1.0.11, RepeatMasker v4.0.7, Mashmap v2, LTR_finder v1.07, MISA v2.1, Fgenesh v3.1.1, GeneWise v2.4.1, StringTie v1.3.5, TransDecoder v5.5.0, KOBAS v2.0, Blast2GO v5.2, BUSCO v3.1.0, Orthofinder v2.3.11, Mafft v7.453, pal2nal v14, IQ-tree v1.6.12, KaKs_calculator v2.0, Lastz v1.02.00, axtToChain v385, chainToAxt v385, axtToMaf v385, roast v3.3, MCScanX v2017Jan4, STAR v2.7.3, STAR-fusion v1.7.0, R v3.5.2, DESeq2 v1.30.0, TBtools v1.046, GATK v3.8, ANNOVAR v2015Dec14, EIGENSOFT v7.2.1, Admixture v1.3.0, PopLDdecay v3.40, VCFtools v0.1.16, Phybase v1.5, GMAP v2018-03-11, Jeffyfish v2.2.10, and SMRTLink 6.0.0.47841.

For manuscripts utilizing custom algorithms or software that are central to the research but not yet described in published literature, software must be made available to editors and reviewers. We strongly encourage code deposition in a community repository (e.g. GitHub). See the Nature Research guidelines for submitting code & software for further information.

## Data

Policy information about availability of data

All manuscripts must include a data availability statement. This statement should provide the following information, where applicable:

- Accession codes, unique identifiers, or web links for publicly available datasets
- A list of figures that have associated raw data
- A description of any restrictions on data availability

The raw genome and transcriptome sequencing data of three species were deposited in the Genome Sequence Archive (GSA)101 in BIG Data Center102 (accession numbers CRA002435, CRA002449, and CRA002464) and the SRA database (project numbers PRJNA684670, PRJNA684766, and PRJNA684636), respectively. The genome resequencing data of three common carp strains were available in both the GSA (accession numbers CRA002466, CRA002415, and CRA002463) and the SRA (project numbers PRJNA684795, PRJNA684797, and PRJNA684676).The assemblies of three genomes were available in both the Genome Warehouse102 in BIG data Center (accession numbers GWHALNJ00000000, GWHACFJ00000000, and GWHACFI00000000) and the Bioproject database (project number PRJNA682709, PRJNA686690, and PRJNA683758). The mRNA sequences, protein sequences, and function annotations of four fish are available at figshare (doi:10.6084/m9.figshare.13886912). Genome assemblies of goldfish were download from GeneBank database with accession number shown in Supplementary Table 3. RNA-seq data of goldfish were downloaded from SRA database and accession number listed in Supplementary Table 7.

# Field-specific reporting

Please select the one below that is the best fit for your research. If you are not sure, read the appropriate sections before making your selection.

☒ Life sciences ☐ Behavioural & social sciences ☐ Ecological, evolutionary & environmental sciences

For a reference copy of the document with all sections, see nature.com/documents/nr-reporting-summary-flat.pdf

# Life sciences study design

All studies must disclose on these points even when the disclosure is negative.

| | |
|---|---|
| Sample size | No statistical methods were used to determine sample size. For each species, one individual was collected for genome assembly. For RNA-sequencing, six samples per species were sampled. A total of 31 individuals of SP strain, 36 individuals of YR strain, and 26 individuals of FR strain were used for genome re-sequencing. |
| Data exclusions | No data was excluded. |
| Replication | The relevant analysis to replication in this study included RNA-seq data and construction of phylogenetic gene trees. The RNA-seq data per tissue was generated with three biological replicates and all attempts at replication were successful. The phylogenetic gene tree was constructed using IQ-tree with 1,000 bootstrap replicates and the consensus tree shown excellent reproducibility which presented in Fig. 5a and Supplementary Fig. 14. The results in the study are reproducible using the raw sequencing data provided. |
| Randomization | Fish individuals for genome sequencing, re-sequencing, and RNA-sequencing were sampled randomly by species or strains. |
| Blinding | Blinding was not relevant for this study. Genomic and transcriptomic characteristic of interest in this study are not influenced by blinding. |

# Reporting for specific materials, systems and methods

We require information from authors about some types of materials, experimental systems and methods used in many studies. Here, indicate whether each material, system or method listed is relevant to your study. If you are not sure if a list item applies to your research, read the appropriate section before selecting a response.

## Materials & experimental systems

| n/a | Involved in the study | |
|---|---|---|
| ☒ | ☐ | Antibodies |
| ☒ | ☐ | Eukaryotic cell lines |
| ☒ | ☐ | Palaeontology and archaeology |
| ☐ | ☒ | Animals and other organisms |
| ☒ | ☐ | Human research participants |
| ☒ | ☐ | Clinical data |
| ☒ | ☐ | Dual use research of concern |

## Methods

| n/a | Involved in the study | |
|---|---|---|
| ☒ | ☐ | ChIP-seq |
| ☒ | ☐ | Flow cytometry |
| ☒ | ☐ | MRI-based neuroimaging |

## Animals and other organisms

Policy information about studies involving animals; ARRIVE guidelines recommended for reporting animal research

| | |
|---|---|
| Laboratory animals | Each individual from common carp (var. 'Songpu'), P. guichenoti, and P. tetrazona used for whole genome sequencing was healthy, female, and mature. For RNA-sequencing, six samples (three females and three males, healthy, and mature) per species were sampled. Three common carp strains, including the YR strain, SP strain, and FR strain, were used for genome re-sequencing (unsexed and one year old). |
| Wild animals | Our study did not sample wild animals. |
| Field-collected samples | Our study did not include field-collected samples. |
| Ethics oversight | The welfare and use of animals in this study was done following the recommendations for scientific purposes set up by the Animal Care and Use Committee of Chinese Academy of Fishery Sciences. |

Note that full information on the approval of the study protocol must also be provided in the manuscript.
