# Supplementary material for: Parallel subgenome structure and divergent expression evolution of allo-tetraploid common carp and goldfish
Source: Nat Genet. 2021 Sep 30;53(10):1493–503. doi: 10.1038/s41588-021-00933-9 (PMC8492472; doi:10.1038/s41588-021-00933-9)
Supplement: Supplementary file 3 — Supplementary Tables 1–37. [file 41588_2021_933_MOESM3_ESM.pdf]

TABLE OF CONTENTS

Supplementary Table 1. Summaries of sequencing data of common carp and two diploid fish 3

Supplementary Table 2. Mapping ratios of RNA-seq reads from nine tissues of three species 4

Supplementary Table 3. Statistics of the common carp genome assemblies 5

Supplementary Table 4. The ratios of RNA-seq alignment to various common carp genome assemblies 6

Supplementary Table 5. Functional annotations of protein-coding genes of four species 7

Supplementary Table 6. BUSCO evaluation of the genes of three fish 8

Supplementary Table 7. The published RNA-seq data used in this study 9

Supplementary Table 8. BUSCO evaluation of different genome annotations from various goldfish assemblies 10

Supplementary Table 9. In common carp, subA and subB gene numbers in the A and B subgenomes 11

Supplementary Table 10. Goldfish subA and subB gene numbers in A and B subgenomes 12

Supplementary Table 11. Depths of *P. guichenoti* reads and *P. tetrazona* reads aligned to different subgenomes 13

Supplementary Table 12. Comparison of subgenome notations in the studies of various goldfish and common carp .. 14

Supplementary Table 13. Repeat contents in the common carp subgenomes 15

Supplementary Table 14. Repeat contents in the goldfish subgenomes 16

Supplementary Table 15. Repeat contents in *P. guichenoti* and *P. tetrazona* 17

Supplementary Table 16. Different types of ARs in six genomes 18

Supplementary Table 17. Gene collinearities among six genomes 19

Supplementary Table 18. Gene collinearities in chr4 among four tetraploid subgenomes 20

Supplementary Table 19. Comparing the exchanged genes and the collinear blocks 21

Supplementary Table 20. Retention and loss of ARs in the genomes of common carp and goldfish 22

Supplementary Table 21. Retention of conserved AGs in different genomes 23

Supplementary Table 22. Median *Ka/Ks* values of genes in the *P. tetrazona*, common carp A, B, goldfish A and B subgenomes with *P. guichenoti* as references 24

Supplementary Table 23. Median *Ka/Ks* values of the subA genes in the A subgenomes and of subB genes in the B subgenomes 25

Supplementary Table 24. In common carp, median *Ka/Ks* values of the exchanged subB genes and the hosted subA genes in the A subgenome 26

Supplementary Table 25. In common carp, *Ka/Ks* values of the exchanged subA genes and the hosted subB genes in the B subgenome 27

Supplementary Table 26. In goldfish, median *Ka/Ks* values of the exchanged subB genes and hosted subA genes in

the A subgenome 28
Supplementary Table 27. In goldfish, *Ka/Ks* values of the exchanged subA genes and the hosted subB genes in the B subgenome...29
Supplementary Table 28. The number of genome-wide genes participating in TS events..30
Supplementary Table 29. Validating the TS events using Pacbio, Illumina and 454 reads 31
Supplementary Table 30. Median TPM values of the homoeologues, pseudo-ancestral homoeologous genes, the *P. guichenoti* genes and the *P. tetrazona* genes 32
Supplementary Table 31. Co-transcription of the homoeologous pairs in nine tissues and nine conditions 33
Supplementary Table 32. Statistics of co-expressed, neo-F, sub-F, and non-F groups of 2,096 homoeologous pairs in nine tissues and nine conditions 34
Supplementary Table 33. The inconsistence among the co-expressed, neo-F, sub-F, and non-F groups of 2,096 homoeologous pairs in nine tissues and nine conditions...35
Supplementary Table 34. Differentially expressed homoeologues in different comparisons...36
Supplementary Table 35. Differentially expressed homoeologous pairs in multiple condition comparisons 37
Supplementary Table 36. Genome-wide DEGs in multiple condition comparisons 38
Supplementary Table 37. Diversity levels of the FR strain and the YR strain..39
References 40

Supplementary Table 1. Summaries of sequencing data of common carp and two diploid fish

	Platform	Insert size	Clean pairs	Total bases (Gb)	Coverage (X)
C. carpio	Illumina	250 bp	135,383,144	20.5	12.1
		300 bp	66,804,549	18.4	10.9
		500 bp	162,302,770	35.7	21.1
		2 K bp	36,606,575	5.8	3.4
		3 K bp	128,373,771	25.7	15.2
		5 K bp	44,473,217	4.4	2.6
		8 K bp	125,711,537	25.1	14.8
	Illumina	350 bp	66,661,327	20	11.8
	Hi-C		383,143,303	114.9	68
	Pacbio		3,178,039	31.37	18.5
	Nanopore		4,920,986	12.41	7.3
	Total			314.28	185.7
P. guichenoti	Illumina	250 bp	182,887,007	54.87	50.3
	Hi-C		330,138,524	99.04	90.8
	Nanopore		3,469,994	51.31	47.1
	Total			205.22	188.2
P. tetrazona	Illumina	250 bp	201,213,668	60.36	82.7
	Hi-C		349,826,035	104.9	143.7
	Nanopore		2,696,840	42.98	58.8
	Total			218.24	285.2

Supplementary Table 2. Mapping ratios of RNA-seq reads from nine tissues of three species

Species	Tissue	Cleaned pairs	Alignment ratio (%)
Common carp	Liver	69,211,656	93.13
	Muscle	61,973,128	96.19
	Intestine	72,456,131	92.71
	Brain	80,438,831	93.57
	Spleen	61,315,543	92.21
	Skin	75,612,388	94.43
	Gill	75,212,421	92.84
	Heart	68,855,264	87.28
	Kidney	69,446,147	92.76
	Total	634,521,509	92.79
P. guichenoti	Liver	64,575,571	91.45
	Muscle	85,751,629	92.71
	Intestine	80,752,219	91.79
	Brain	75,862,972	92.71
	Spleen	88,866,374	91.73
	Skin	63,326,135	91.94
	Gill	86,677,421	91.42
	Heart	101,793,641	92.25
	Kidney	71,558,976	91.98
	Total	719,164,938	92
P. tetrazona	Liver	78,565,579	92.95
	Muscle	76,441,557	94.08
	Intestine	71,852,295	85.74
	Brain	72,358,707	93.82
	Spleen	64,320,718	94.76
	Skin	77,023,296	93.63
	Gill	78,781,149	92.18
	Heart	89,114,067	93.4
	Kidney	84,171,201	93.1
	Total	692,628,569	92.51

Supplementary Table 3. Statistics of the common carp genome assemblies

	Genome Size (Mb)	Genome size without gaps (Mb)	Contig N50 (Mb)	Contigs number	Total sizes of chromosomes (Mb)	Gap number
GCA_000951615.2[1]	1,713	1,670	0.075	53,088	875	57,939
GCA_001270105.1[2]	1,380	1,361	0.007	427,338	-	479,190
GCA_004011575.1[3]	1,423	1,391	0.060	184,435	1,260	109,782
GCA_004011595.1[3]	1,457	1,407	0.036	316,365	1,240	93,212
GCA_004011555.1[3]	1,415	1,359	0.094	53,446	1,300	58,464
Current	1,681	1,673	1.554	20,528	1,531	13,694

Supplementary Table 4. The ratios of RNA-seq alignment to various common carp genome assemblies

	**RNA-seq alignment ratio**
GCA_000951615.2[1]	85.78%
GCA_001270105.1[2]	90.28%
GCA_004011575.1[3]	86.43%
GCA_004011595.1[3]	85.32%
GCA_004011555.1[3]	89.13%
Current	92.79%

Supplementary Table 5. Functional annotations of protein-coding genes of four species

**Database**	**Common carp**	**Goldfish**	***P. guichenoti***	***P. tetrazona***
GO	31,439	32,134	16,937	16,560
KEGG	26,126	29,537	14,045	13,468
KOG	27,217	27,397	14,697	14,220
NR	45,562	45,492	23,792	21,716
NT	47,669	48,142	24,103	21,804
Swiss-Prot	37,951	38,249	20,583	19,860
All annotated	47,687	48,159	24,122	21,869
In chromosomes	45,118	46,257	21,889	19,767
Total genes	47,925	48,857	24,284	21,943

Supplementary Table 6. BUSCO evaluation of the genes of three fish

Species	Complete BUSCOs		Fragmented BUSCOs	Missing BUSCOs	Total BUSCO actinopterygii genes
	Complete and single-copy BUSCOs	Complete and duplicated BUSCOs			
Common carp	1,708 (37.3%)	2,520 (55.0%)	138 (3.0%)	218 (4.7%)	4,584 (100%)
P. guichenoti	3,330 (91.5%)	130 (3.6%)	47 (1.3%)	133 (3.6%)	3,640 (100%)
P. tetrazona	3,467 (95.2%)	75 (2.1%)	27 (0.7%)	71 (2.0%)	3,640 (100%)

Supplementary Table 7. The published RNA-seq data used in this study

Species		Comparison	Group	Accession number in Bioproject or SRA or GSA
Common carp	Condition comparison	Hypoxia stress treatment	Hypoxia treatment	PRJNA512071
			Control group	
		CyHV3 infection	Acute infection	PRJNA314552
			Mock infection	
		Aeromonas hydrophila infection	Treatment group	PRJNA315069
			Control group	
		Skin coloration	Black skin	PRJNA508277
			Red skin	
			White skin	
	Validating TS events		Polished Pacbio Iso-seq dataset	SRR8791143
			TSA dataset	GFWU00000000.1
			454 long read dataset	PRJNA79467, PRJNA82763, PRJNA88151, PRJNA257337
Goldfish	Tissue profiling		Liver	CRR046311, CRR046312
			Muscle	SRR8712980, SRR8712981, SRR8712983
			Intestine	SRR10358004
			Brain	CRR046301, CRR046302
			Spleen	CRR053075, CRR053076
			Skin	SRR10358011, SRR10358024
			Gill	SRR7749872, SRR7749875
			Heart	SRR7749871, SRR10358005
			Kidney	CRR046309, CRR046310
	Condition comparison	Gyrodactylus infection experiment	Infection group	PRJNA564889
			Control group	
		Feed conversion efficiency (FCE) comparison	High FCE group	PRJNA433432
			Low FCE group	
		Bisphenol A Treatment	Treatment group	PRJNA514986
			Control group	
		Secretoneurin A treatment	sectretoneurin A (SNa) treatment group	PRJNA415604
			selective dopamine D1 receptor (SKF) treatment group	
			Control group	
	Validating TS events		Unpolished Pacbio Iso-seq data	SRR9924704, SRR9924705, SRR11585445, SRR10382503, SRR10382504, SRR10382505
			TSA dataset	GBZM00000000.1
			454 long read dataset	SRR609300, SRR1535130, SRR609301, SRR609302

Supplementary Table 8. BUSCO evaluation of different genome annotations from various goldfish assemblies

Assemblies	Gene number	Complete BUSCOs		Fragmented BUSCOs	Missing BUSCOs	Total BUSCO actinopterygii genes
		Complete and single-copy	Complete and duplicated			
Chen, Z *et al.* 2019[4]	80,065	1,689 (36.8%)	2,544 (55.5%)	241 (5.3%)	110 (2.4%)	4,584 (100%)
Luo, J *et al*. 2020[5]	43,144	2,289 (49.9%)	2,093 (45.7%)	71 (1.5%)	131 (2.9%)	4,584 (100%)
Chen, D. *et al.* 2020[6]	56,251	2,064 (45.0%)	2,280 (49.7%)	92 (2.0%)	148 (3.3%)	4,584 (100%)
This study	48,857	2,361 (51.5%)	2,124 (46.3%)	66 (1.4%)	33 (0.8%)	4,584 (100%)

Supplementary Table 9. In common carp, subA and subB gene numbers in the A and B subgenomes

A subgenome chromosome *			B subgenome chromosome *		
No.	subA gene number	subB gene number	No.	subA gene number	subB gene number
A1	70	2	B1	2	70
A2	97	10	B2	7	100
A3	100	1	B3	2	97
A4	78	3	B4	2	79
A5	104	6	B5	6	104
A6	99	1	B6	1	90
A7	114	5	B7	5	115
A8	93	0	B8	0	91
A9	78	1	B9	2	78
A10	60	4	B10	4	61
A11	87	3	B11	3	86
A12	57	4	B12	4	57
A13	105	1	B13	1	104
A14	50	3	B14	1	55
A15	71	2	B15	1	72
A16	113	2	B16	2	118
A17	92	1	B17	1	93
A18	73	3	B18	3	72
A19	97	3	B19	5	97
A20	78	1	B20	1	81
A21	53	1	B21	1	54
A22	41	1	B22	0	41
A23	87	2	B23	2	88
A24	50	1	B24	1	51
A25	58	1	B25	1	57

* Genes located on the scaffolds were excluded.

Supplementary Table 10. Goldfish subA and subB gene numbers in A and B subgenomes

A subgenome chromosome *			B subgenome chromosome *		
No.	subA gene number	subB gene number	No.	subA gene number	subB gene number
A1	69	2	B1	3	69
A2	98	6	B2	6	101
A3	99	1	B3	1	99
A4	76	2	B4	3	77
A5	105	3	B5	3	107
A6	98	1	B6	1	97
A7	117	4	B7	5	115
A8	90	0	B8	0	91
A9	78	1	B9	1	78
A10	60	4	B10	4	62
A11	89	2	B11	1	89
A12	59	3	B12	3	56
A13	103	1	B13	2	105
A14	54	1	B14	0	55
A15	70	1	B15	2	70
A16	119	2	B16	2	118
A17	93	1	B17	1	94
A18	71	3	B18	3	70
A19	100	4	B19	4	100
A20	81	2	B20	2	80
A21	55	1	B21	1	55
A22	42	2	B22	1	43
A23	86	1	B23	2	89
A24	50	1	B24	1	51
A25	55	2	B25	1	57

* Genes located on the scaffolds were excluded.

Supplementary Table 11. Depths of *P. guichenoti* reads and *P. tetrazona* reads aligned to different subgenomes

Common carp	Mean depth of *P. guichenoti* reads*	Mean depth of *P. tetrazona* reads	Goldfish	Mean depth of *P. guichenoti* reads	Mean depth of *P. tetrazona* reads
A1 / B1	13.27 / 9.13	6.23 / 18.90	A1 / B1	10.80 / 8.86	4.72 / 17.27
A2 / B2	12.84 / 10.91	6.70 / 21.57	A2 / B2	12.42 / 10.89	5.68 / 18.47
A3 / B3	12.50 / 10.11	7.41 / 15.78	A3 / B3	11.69 / 12.00	6.27 / 11.84
A4 / B4	11.09 / 13.00	5.14 / 16.01	A4 / B4	10.79 / 12.07	4.62 / 15.39
A5 / B5	10.44 / 10.52	6.24 / 22.91	A5 / B5	8.76 / 8.60	5.85 / 17.03
A6 / B6	12.77 / 10.28	6.89 / 23.27	A6 / B6	8.21 / 9.62	6.27 / 20.33
A7 / B7	11.24 / 11.06	6.48 / 21.32	A7 / B7	10.22 / 9.72	5.88 / 19.12
A8 / B8	13.03 / 12.41	7.49 / 21.87	A8 / B8	11.14 / 9.33	6.17 / 17.70
A9 / B9	12.09 / 11.29	6.62 / 22.35	A9 / B9	9.64 / 9.20	6.73 / 19.39
A10 / B10	11.82 / 16.04	6.60 / 22.94	A10 / B10	11.08 / 9.17	6.81 / 18.22
A11 / B11	13.40 / 11.87	7.21 / 24.97	A11 / B11	10.52 / 11.11	6.32 / 19.14
A12 / B12	12.31 / 10.50	7.21 / 22.47	A12 / B12	8.84 / 7.99	6.41 / 17.32
A13 / B13	10.55 / 14.27	6.51 / 25.09	A13 / B13	9.15 / 10.14	5.89 / 20.29
A14 / B14	12.75 / 11.02	8.74 / 21.50	A14 / B14	10.97 / 9.25	6.37 / 20.43
A15 / B15	12.25 / 12.62	6.48 / 21.00	A15 / B15	10.16 / 11.22	6.38 / 17.71
A16 / B16	10.49 / 10.57	6.56 / 22.85	A16 / B16	9.16 / 10.75	6.38 / 17.96
A17 / B17	13.55 / 11.25	7.56 / 23.74	A17 / B17	9.36 / 10.20	6.80 / 19.68
A18 / B18	13.18 / 12.49	6.65 / 23.02	A18 / B18	11.84 / 11.15	7.05 / 18.56
A19 / B19	15.33 / 12.40	6.48 / 21.86	A19 / B19	11.54 / 9.42	7.18 / 18.88
A20 / B20	11.82 / 12.52	6.41 / 22.07	A20 / B20	8.45 / 13.16	6.31 / 18.69
A21 / B21	15.02 / 13.17	11.34 / 19.59	A21 / B21	10.39 / 12.28	6.68 / 17.17
A22 / B22	10.68 / 8.75	5.93 / 12.63	A22 / B22	9.13 / 12.03	6.85 / 9.26
A23 / B23	12.68 / 12.48	7.04 / 24.07	A23 / B23	11.42 / 11.67	7.40 / 21.35
A24 / B24	11.74 / 11.74	6.96 / 21.27	A24 / B24	13.89 / 9.42	6.22 / 18.28
A25 / B25	9.27 / 11.28	6.17 / 19.45	A25 / B25	10.17 / 10.86	7.05 / 14.58

* Depth of all non-overlapping blocks in one chromosome. The depth of one block = read number * read length / 1 kb.

Supplementary Table 12. Comparison of subgenome notations in the studies of various goldfish and common carp

Common carp	Xu *et al.* 2019[3]	A subgenome (633 Mb)	B subgenome (671 Mb)
	Current study	A subgenome (745 Mb)	B subgenome (785 Mb)
Goldfish	Kon *et al.* 2020[7]	S subgenome (573 Mb)	L subgenome (701 Mb)
	Luo *et al.* 2020[5]	P subgenome (719 Mb)	M subgenome (781 Mb)
	Chen *et al.* 2020[6]	B subgenome (861 Mb)	A subgenome (793 Mb)
	Current study	A subgenome (793 Mb)	B subgenome (861 Mb)

Supplementary Table 13. Repeat contents in the common carp subgenomes

	Common carp genome		A subgenome		B subgenome	
	Length	Percentage	Length	Percentage	Length	Percentage
Retro-elements	117,792,730	7.01	55,792,684	7.48	56,866,894	7.24
SINEs	5,662,370	0.34	2,536,842	0.34	2,567,849	0.33
Penelope	335,214	0.02	129,041	0.02	158,649	0.02
LINEs	70,994,938	4.22	29,659,579	3.97	29,735,692	3.79
L2/CR1/Rex	54,310,801	3.23	22,734,921	3.05	23,247,310	2.96
R1/LOA/Jockey	2,655,105	0.16	983,331	0.13	1,105,382	0.14
R2/R4/NeSL	437,489	0.03	219,324	0.03	168,804	0.02
RTE/Bov-B	1,463,779	0.09	666,636	0.09	660,755	0.084
L1/CIN4	5,376,779	0.32	2,191,967	0.29	2,134,039	0.27
LTR elements	57,457,118	3.42	23,596,263	3.16	24,563,353	3.13
BEL/Pao	4,166,078	0.25	1,689,793	0.23	1,618,223	0.2
Ty1/Copia	208,035	0.01	70,504	0.01	91,379	0.01
Gypsy/DIRS1	3,4625,668	2.06	14,237,597	1.91	14,825,705	1.89
Retroviral	3,685,147	0.22	1,373,550	0.18	1,448,770	0.18
DNA transposons	193,596,666	11.51	82,181,115	11.02	93,224,685	11.87
hobo-Activator	34,254,732	2.04	14,183,556	1.9	16,739,149	2.13
Tc1-IS630-Pogo	46,860,165	2.79	20,779,746	2.78	22,044,890	2.81
PiggyBac	6,136,294	0.36	2,450,192	0.33	2,751,787	0.35
Tourist/Harbinger	9,071,470	0.54	3,915,175	0.53	4,233,018	0.54
Other	1,091,794	0.06	449,475	0.06	526,162	0.07
Unclassified	260,477,055	15.49	109,865,486	14.73	119,173,658	15.17
Total interspersed repeats	588,188,147	34.98	247,839,285	33.23	269,265,237	34.29
Small RNA	946,134	0.06	343,786	0.05	405,147	0.05
Satellites	15,870,492	0.94	7,205,565	0.97	7,527,118	0.96
Simple repeats	60,586,113	3.6	27,992,855	3.75	28,298,093	3.6
Low complexity	8,308,641	0.49	3,831,033	0.51	3,893,686	0.49
Total repeats	673,899,527	40.08	287,212,524	38.5	309,389,281	39.4

Supplementary Table 14. Repeat contents in the goldfish subgenomes

	goldfish whole genome		A subgenome		B subgenome	
	Length	Percentage	Length	Percentage	Length	Percentage
Retro-elements	159,198,091	9.15	73,317,324	9.24	74,160,681	8.61
SINEs	6,104,345	0.35	2,883,896	0.36	2,932,803	0.34
Penelope	260,540	0.015	115,940	0.015	128,707	0.015
LINEs	78,505,890	4.51	35,612,365	4.49	36,671,886	4.26
L2/CR1/Rex	62,185,120	3.57	28,433,488	3.59	29,159,057	3.39
R1/LOA/Jockey	2,650,726	0.15	1,049,785	0.13	1,305,205	0.15
R2/R4/NeSL	426,180	0.024	206,065	0.026	1,305,205	0.15
RTE/Bov-B	2,138,629	0.12	945,958	0.12	993,003	0.12
L1/CIN4	4,138,787	0.24	1,870,903	0.24	1,925,830	0.22
LTR elements	74,587,856	4.29	34,821,063	4.39	34,555,992	4.01
BEL/Pao	5,273,726	0.30	2,209,518	0.28	2,551,266	0.30
Ty1/Copia	318,554	0.018	152,575	0.019	139,897	0.016
Gypsy/DIRS1	45,581,181	2.62	21,466,568	2.71	20,975,659	2.44
Retroviral	2,871,379	0.165	1,289,125	0.16	1,310,955	0.15
DNA transposons	195,346,337	11.23	87,445,423	11.03	97,040,672	11.27
hobo-Activator	36,740,790	2.11	16,338,837	2.06	18,340,571	2.13
Tc1-IS630-Pogo	37,930,816	2.18	17,607,428	2.22	18,479,549	2.15
PiggyBac	4,926,036	0.28	2,256,468	0.28	2,371,525	0.28
Tourist/Harbinger	9,269,578	0.53	4,170,628	0.53	4,597,689	0.53
Other	795,405	0.046	381,787	0.048	369,372	0.043
Unclassified	255,050,911	14.66	115,508,852	14.57	123,766,634	14.38
Total interspersed repeats	609,595,339	35.04	276,271,599	34.84	294,967,987	34.26
Small RNA	826,925	0.048	326,008	0.041	412,086	0.048
Satellites	12,718,002	0.73	5,831,078	0.74	6,324,525	0.73
Simple repeats	51,607,990	2.97	23,715,694	2.99	25,250,074	2.93
Low complexity	6,171,919	0.35	2,815,833	0.36	3,068,100	0.36
Total repeats	680,920,175	39.14	308,960,212	38.96	330,022,772	38.33

Supplementary Table 15. Repeat contents in *P. guichenoti* and *P. tetrazona*

	P. guichenoti		*P. tetrazona*	
	**Length**	**Percentage**	**Length**	**Percentage**
Retro-elements	111,947,317	10.29	41,965,681	5.74
SINEs	1,497,418	0.14	305,551	0.04
Penelope	147,272	0.01	93,285	0.01
LINEs	60,189,518	5.53	17,858,055	2.44
L2/CR1/Rex	45,803,304	4.21	12,166,863	1.66
R1/LOA/Jockey	774,029	0.07	128,535	0.02
R2/R4/NeSL	185,752	0.02	57,857	0.007
RTE/Bov-B	4,029,731	0.37	1,770,363	0.24
L1/CIN4	1,349,745	0.12	1,197,554	0.16
LTR elements	50,260,381	4.62	23,802,075	3.26
BEL/Pao	1,883,481	0.17	1,054,122	0.144
Ty1/Copia	115,341	0.01	50,685	0.007
Gypsy/DIRS1	25,380,900	2.33	13,782,074	1.89
Retroviral	5,659,630	0.52	2,492,684	0.34
DNA transposons	213,879,929	19.66	46,205,602	6.32
hobo-Activator	23,037,871	2.12	8,367,969	1.14
Tc1-IS630-Pogo	84,833,139	7.8	8,649,425	1.18
PiggyBac	3,762,452	0.35	285,301	0.04
Tourist/Harbinger	15,209,215	1.4	1,833,390	0.25
Other	1,866,549	0.17	54,194	0.007
Unclassified	202,422,532	18.61	79,980,092	10.94
Total interspersed repeats	528,249,778	48.56	168,151,375	23.01
Small RNA	995,256	0.09	2,434,888	0.33
Satellites	8,308,627	0.76	4,430,804	0.61
Simple repeats	51,396,740	4.72	33,081,585	4.52
Low complexity	3,580,297	0.33	2,871,688	0.39
Total repeats	592,530,698	54.46	210,970,340	28.86

Supplementary Table 16. Different types of ARs in six genomes

	Consistent ARs	Inversed ARs	Translocated ARs	Total ARs
P. guichenoti	102,139 (60.8%)	45,780 (27.3%)	19,895 (11.9%)	167,814
Common carp A	170,653 (84.4%)	23,721 (11.7%)	7,748 (3.8%)	202,122
Common carp B	178,252 (84.4%)	25,979 (12.3%)	6,958 (3.3%)	211,189
Goldfish A	177,752 (86.1%)	21,282 (10.3%)	7,451 (3.6%)	206,485
Goldfish B	188,524 (84.0%)	28,393 (12.6%)	7,648 (3.4%)	224,565
P. tetrazona	172,828 (76.1%)	29,861 (13.1%)	24,431 (10.8%)	227,120

Supplementary Table 17. Gene collinearities among six genomes

	Comparison	Consistent	Inversion	Translocation	Total
Common carp	A *vs* B	11,572 (72.9%)	3,120 (19.6%)	1,185 (7.5%)	15,883
	P. tetrazona vs A	9,341 (68.1%)	3,298 (24%)	1,077 (7.9%)	13,716
	P. tetrazona vs B	9,706 (67.4%)	3,458 (24%)	1,244 (8.6%)	14,408
	P. guichenoti vs A	7,301 (53.3%)	5,400 (39.5%)	990 (7.2%)	13,691
	P. guichenoti vs B	7,559 (52.7%)	5,727 (39.9%)	1,059 (7.4%)	14,345
Goldfish	A *vs* B	10,910 (75.8%)	2,500 (17.4%)	991 (6.9%)	14,401
	P. tetrazona vs A	8,954 (68.3%)	3,082 (23.5%)	1,080 (8.2%)	13,116
	P. tetrazona vs B	9,603 (66.0%)	3,752 (25.8%)	1,205 (8.3%)	14,560
	P. guichenoti vs A	6,897 (53.0%)	5,202 (40.0%)	918 (7.1%)	13,017
	P. guichenoti vs B	7,762 (54.0%)	5,680 (39.5%)	920 (6.4%)	14,362

Supplementary Table 18. Gene collinearities in chr4 among four tetraploid subgenomes

Comparison	Collinear pairs
Common carp chrA4 (912) *vs* chrB4 (1,039)	600
Goldfish chrA4 (983) *vs* chrB4 (939)	382
Goldfish chrA4 (983) *vs* common carp chrA4 (912)	399
Goldfish chrB4 (939) *vs* common carp chrB4 (1,039)	551

Supplementary Table 19. Comparing the exchanged genes and the collinear blocks

		Non-syntenic block	Syntenic block			Total
			Consistent	Inversion	Translocation	
Common carp	exchanged subA genes to B subgenome	10	40	12	0	62
	exchanged subB genes to A subgenome	4	40	13	1	58
Goldfish	exchanged subA genes to B subgenome	12	33	6	0	51
	exchanged subB genes to A subgenome	14	33	6	0	53

Supplementary Table 20. Retention and loss of ARs in the genomes of common carp and goldfish

	**Type**	**Conserved ARs**
Common carp	Lost ARs in both subgenomes	7,004 (2.5%)
	Retained ARs in both subgenomes	144,387 (52.3%)
	Retained ARs in only A subgenome	57,735 (20.9%)
	Retained AR in only B subgenome	66,802 (24.2%)
Goldfish	Lost ARs in both subgenomes	4,073 (1.5%)
	Retained ARs in both subgenomes	159,195 (57.7%)
	Retained AR in only A subgenome	47,290 (17.1%)
	Retained AR in only B subgenome	65,370 (23.7%)
P. guichenoti		167,814 (60.8%)
P. tetrazona		227,120 (82.3%)
	Total ARs	275,928

Supplementary Table 21. Retention of conserved AGs in different genomes

	Type	Conserved AG number	Families including AGs	Background gene number	*P* value*
Common carp	A subgenome	13,585 (61.5%#)	9,654	22,080	1.37×10^{-73}
	B subgenome	13,806 (59.9%)	9,821	23,038	4.42×10^{-50}
Goldfish	A subgenome	13,895 (63.4%)	9,504	21,902	1.77×10^{-109}
	B subgenome	15,345 (63.0%)	10,213	24,355	5.46×10^{-106}
P. tetrazona		11,622 (53.0%)	8,663	21,943	
P. guichenoti		10,974 (45.2%)	8,392	24,284	

#: the proportion of retained AGs in comparison with all genes in the corresponding genome.

*: Compared to the AG retention rates in the *P. tetrazona* with two-sided Chi-square test.

Supplementary Table 22. Median *Ka/Ks* values of genes in the *P. tetrazona*, common carp A, B, goldfish A and B subgenomes with *P. guichenoti* as references

Chromosome	Medians of genes in *P. tetrazona*	Common carp A subgenome		Common carp B subgenome		P value#	Goldfish A subgenome		Goldfish B subgenome		P value#
		Median	P*	Median	P*		Median	P*	Median	P*	
1	0.22	0.22	0.10	0.20	0.32	0.59	0.21	0.13	0.22	0.27	0.83
2	0.19	0.20	0.19	0.21	0.05	0.49	0.19	0.37	0.18	0.48	0.74
3	0.19	0.20	0.10	0.19	0.17	0.76	0.20	0.07	0.18	0.32	0.38
4	0.19	0.19	0.05	0.18	0.22	0.44	0.19	0.15	0.18	0.28	0.66
5	0.22	0.23	0.03	0.22	0.12	0.40	0.22	0.10	0.22	0.16	0.84
6	0.20	0.23	0.01	0.20	0.17	0.18	0.21	0.07	0.20	0.20	0.62
7	0.20	0.21	0.03	0.22	0.07	0.57	0.21	0.06	0.20	0.41	0.33
8	0.20	0.23	0.01	0.22	0.02	0.77	0.21	0.03	0.19	0.13	0.46
9	0.23	0.24	0.13	0.21	0.52	0.45	0.24	0.28	0.20	0.89	0.43
10	0.22	0.21	0.46	0.18	0.92	0.34	0.20	0.41	0.18	0.89	0.39
11	0.18	0.18	0.03	0.16	0.25	0.31	0.18	0.07	0.16	0.35	0.40
12	0.18	0.20	0.03	0.18	0.12	0.53	0.20	0.05	0.18	0.16	0.68
13	0.21	0.23	0.04	0.21	0.24	0.44	0.23	0.12	0.21	0.19	0.87
14	0.20	0.17	0.26	0.19	0.29	0.92	0.20	0.11	0.18	0.45	0.45
15	0.18	0.18	0.03	0.18	0.09	0.55	0.18	0.01	0.19	0.06	0.49
16	0.21	0.22	0.08	0.20	0.18	0.77	0.21	0.17	0.20	0.26	0.86
17	0.22	0.22	0.13	0.23	0.16	0.88	0.20	0.37	0.22	0.42	0.79
18	0.20	0.21	0.07	0.18	0.36	0.37	0.20	0.21	0.17	0.78	0.37
19	0.20	0.22	0.08	0.20	0.26	0.49	0.21	0.05	0.21	0.15	0.65
20	0.20	0.21	0.03	0.20	0.18	0.45	0.19	0.17	0.20	0.42	0.66
21	0.20	0.21	0.23	0.19	0.49	0.64	0.19	0.34	0.19	0.54	0.76
22	0.19	0.19	0.18	0.17	0.85	0.19	0.19	0.24	0.15	0.57	0.08
23	0.19	0.21	0.04	0.19	0.18	0.56	0.21	0.21	0.18	0.45	0.56
24	0.19	0.22	0.19	0.21	0.28	0.81	0.21	0.23	0.19	0.38	0.72
25	0.19	0.21	0.13	0.22	0.17	0.91	0.22	0.17	0.20	0.22	0.83

*:Two-sided Mann-Whitney *U* test comparing the homoeologues and their *P. tetrazona* orthologs); #: Two-sided Mann-Whitney *U* test comparing the homoeologues.

Supplementary Table 23. Median *Ka/Ks* values of the subA genes in the A subgenomes and of subB genes in the B subgenomes

	Common carp			Goldfish		
Chromosome Number	Median *Ka/Ks* values of subA genes in A subgenome	Median *Ka/Ks* values of subB genes in B subgenome	Mann-Whitney *U* test P value#	Median *Ka/Ks* values of subA genes in A subgenome	Median *Ka/Ks* values of subB genes in B subgenome	Mann-Whitney *U* test P value#
1	0.220	0.197	0.528	0.219	0.219	0.676
2	0.192	0.210	0.613	0.190	0.176	0.634
3	0.194	0.178	0.732	0.204	0.182	0.338
4	0.190	0.170	0.497	0.190	0.178	0.595
5	0.227	0.223	0.475	0.219	0.222	0.873
6	0.237	0.201	0.170	0.213	0.202	0.609
7	0.215	0.224	0.499	0.208	0.201	0.351
8	0.228	0.222	0.769	0.215	0.193	0.459
9	0.245	0.221	0.471	0.240	0.205	0.436
10	0.187	0.171	0.352	0.193	0.181	0.453
11	0.176	0.161	0.360	0.171	0.162	0.420
12	0.208	0.182	0.482	0.201	0.182	0.566
13	0.235	0.211	0.444	0.230	0.211	0.796
14	0.159	0.183	0.943	0.200	0.177	0.458
15	0.184	0.177	0.641	0.182	0.188	0.569
16	0.215	0.204	0.738	0.210	0.204	0.864
17	0.225	0.226	0.907	0.202	0.217	0.809
18	0.208	0.185	0.368	0.200	0.174	0.329
19	0.216	0.201	0.452	0.208	0.214	0.577
20	0.210	0.202	0.435	0.187	0.196	0.611
21	0.209	0.192	0.590	0.187	0.188	0.706
22	0.187	0.170	0.233	0.179	0.165	0.130
23	0.198	0.188	0.564	0.206	0.181	0.548
24	0.219	0.214	0.820	0.220	0.195	0.691
25	0.214	0.221	0.973	0.217	0.198	0.889

#: Two-sided Mann-Whitney *U* test comparing the homoeologues.

Supplementary Table 24. In common carp, median *Ka/Ks* values of the exchanged subB genes and the hosted subA genes in the A subgenome

	Median *Ka/Ks* values of subA genes in A subgenome	Median *Ka/Ks* values of subB genes in A subgenome	Mann-Whitney *U* test value *
1	0.220	0.250&	NA
2	0.192	0.278	0.254
3	0.194	0.235&	NA
4	0.190	0.297&	NA
5	0.227	0.161	0.301
6	0.237	0.249&	NA
7	0.215	0.252	0.957
8	0.228	NA#	NA
9	0.245	0.138&	NA
10	0.187	0.326	0.298
11	0.176	0.287	0.559
12	0.208	0.180	0.988
13	0.235	0.074&	NA
14	0.159	0.376&	NA
15	0.184	0.115&	NA
16	0.215	0.179&	NA
17	0.225	0.313&	NA
18	0.208	0.150	0.852
19	0.216	0.170	0.227
20	0.210	0.422&	NA
21	0.209	0.137&	NA
22	0.187	NA#	NA
23	0.198	0.350&	NA
24	0.219	0.094&	NA
25	0.214	0.249&	NA

*: Two-sided Mann-Whitney *U* test comparing the genes in the A subgenome.

#: No exchanged gene for calculating Ka/Ks value.

&: there were at most two exchanged genes in the A subgenome. We cannot perform two-sided Mann-Whitney *U* test.

Supplementary Table 25. In common carp, *Ka/Ks* values of the exchanged subA genes and the hosted subB genes in the B subgenome

	Median *Ka/Ks* values of subB genes in B subgenome	Median *Ka/Ks* values of subA genes in B subgenome	Mann-Whitney *U* test P value*
1	0.197	0.182[&]	NA
2	0.210	0.215	0.954
3	0.178	0.213[&]	NA
4	0.170	0.272	0.120
5	0.223	0.231	0.963
6	0.201	0.203[&]	NA
7	0.224	0.189	0.667
8	0.222	NA[#]	NA
9	0.221	0.079[&]	NA
10	0.171	0.332	0.104
11	0.161	0.288	0.099
12	0.182	0.182	0.988
13	0.211	0.089[&]	NA
14	0.183	0.238	0.461
15	0.177	0.261[&]	NA
16	0.204	0.149[&]	NA
17	0.226	0.403[&]	NA
18	0.185	0.158	0.903
19	0.201	0.113	0.096
20	0.202	0.373[&]	NA
21	0.192	0.101[&]	NA
22	0.170	0.341[&]	NA
23	0.188	0.328[&]	NA
24	0.214	0.093[&]	NA
25	0.221	0.360[&]	NA

*: Two-sided Mann-Whitney *U* test comparing the genes in the B subgenome.

[#]: No exchanged gene for calculating Ka/Ks value.

[&]: There were at most two exchanged genes in the B subgenome. Therefore, we cannot perform two-sided Mann-Whitney *U* test.

Supplementary Table 26. In goldfish, median *Ka/Ks* values of the exchanged subB genes and hosted subA genes in the A subgenome

	Median *Ka/Ks* values of subA genes in A subgenome	Median *Ka/Ks* values of subB genes in A subgenome	Mann-Whitney *U* test P value *
1	0.219	0.292	0.612
2	0.190	0.182	0.983
3	0.204	0.261[&]	NA
4	0.190	0.257	0.223
5	0.219	0.110	0.390
6	0.213	0.213[&]	NA
7	0.208	0.232	0.757
8	0.215	NA[#]	NA
9	0.240	0.097[&]	NA
10	0.193	0.323	0.298
11	0.171	0.673[&]	NA
12	0.201	0.175	0.974
13	0.230	0.250[&]	NA
14	0.200	NA[#]	NA
15	0.182	0.112[&]	NA
16	0.210	0.137[&]	NA
17	0.202	0.341[&]	NA
18	0.200	0.145	0.742
19	0.208	0.157	0.606
20	0.187	0.316[&]	NA
21	0.187	0.555[&]	NA
22	0.179	0.132[&]	NA
23	0.206	0.292[&]	NA
24	0.220	0.084[&]	NA
25	0.217	0.248[&]	NA

*: Two-sided Mann-Whitney *U* test comparing the genes in the A subgenome.

[#]: No exchanged gene for calculating Ka/Ks value.

[&]: There were at most two exchanged genes in A subgenome. Therefore, we cannot perform two-sided Mann-Whitney *U* test.

Supplementary Table 27. In goldfish, *Ka/Ks* values of the exchanged subA genes and the hosted subB genes in the B subgenome

	Median *Ka/Ks* values of subB genes in B subgenome	Median *Ka/Ks* values of subA genes in B subgenome	Mann-Whitney *U* test P value*
1	0.219	0.157[&]	NA
2	0.176	0.180	0.583
3	0.182	0.157[&]	NA
4	0.178	0.380[&]	NA
5	0.222	0.155	0.646
6	0.202	0.188[&]	NA
7	0.201	0.242	0.718
8	0.193	NA[#]	NA
9	0.205	0.120[&]	NA
10	0.181	0.441	0.055
11	0.162	0.441[&]	NA
12	0.182	0.121	0.417
13	0.211	0.088[&]	NA
14	0.177	0.197[&]	NA
15	0.188	0.136[&]	NA
16	0.204	0.147[&]	NA
17	0.217	0.408[&]	NA
18	0.174	0.140	0.708
19	0.214	0.143	0.315
20	0.196	0.285[&]	NA
21	0.188	0.328[&]	NA
22	0.165	0.323[&]	NA
23	0.181	0.306[&]	NA
24	0.195	0.075[&]	NA
25	0.198	0.317[&]	NA

*: Two-sided Mann-Whitney *U* test comparing the genes in the B subgenome.

[#]: No exchanged gene for calculating *Ka/Ks* value.

[&]: There were at most two exchanged genes in the B subgenome. Therefore, we cannot perform Mann-Whitney *U* test.

Supplementary Table 28. The number of genome-wide genes participating in TS events

	**Genome**	**Genes involved in TS**
2,096 sextuplets	Common carp subA	38 (1.81%)
	Common carp subB	41 (1.96%)
	Goldfish subA	12 (0.57%)
	Goldfish subB	11 (0.52%)
	P. tetrazona	30 (1.43%)
	P. guichenoti	83 (3.96%)
All genes	Common carp A subgenome	408 (1.85%)
	Common carp B subgenome	400 (1.73%)
	Goldfish A subgenome	179 (0.82%)
	Goldfish B subgenome	193 (0.79%)
	P. tetrazona genome	290 (1.32%)
	P. guichenoti genome	864 (3.55%)

Supplementary Table 29. Validating the TS events using Pacbio, Illumina and 454 reads

	TS events	Validate TS events				TS events with coding potentials
		PacBio data	TSA sequences	454 read	All	
Common carp	496*	46	48	8	87	79
Goldfish	292	102	23	9	112	111

* We identified 496 TS events in common carp, including 461 between chromosomes and 35 between chromosomes and scaffolds.

Supplementary Table 30. Median TPM values of the homoeologues, pseudo-ancestral homoeologous genes, the *P. guichenoti* genes and the *P. tetrazona* genes

	Tissues	subA genes	subB genes	Mean	pseudo-ancestral genes	*P. guichenoti* genes	*P. tetrazona* genes
Common carp	Brain	23.62	24.94	24.28	48.56	33.89	46.20
	Gill	22.84	23.2	23.02	46.04	34.55	46.26
	Heart	17.47	20.32	18.89	37.79	24.63	19.92
	Intestine	19.22	19.48	19.35	38.70	36.03	35.36
	Kidney	21.43	22.57	22.00	44.00	35.28	30.89
	Liver	14.43	15.23	14.83	29.65	21.45	31.32
	Muscle	15.12	20.81	17.96	35.93	32.12	32.78
	Skin	19.83	22.79	21.31	42.62	31.13	35.69
	Spleen	22.37	23.41	22.89	45.78	37.76	27.87
Goldfish	Brain	20.75	24.35	22.55	45.10	33.89	46.20
	Gill	19.51	20.56	20.04	40.08	34.55	46.26
	Heart	16.69	19.03	17.86	35.72	24.63	19.92
	Intestine	12.66	15.19	13.92	27.84	36.03	35.36
	Kidney	25.47	26.00	25.73	51.47	35.28	30.89
	Liver	12.94	16.58	14.76	29.52	21.45	31.32
	Muscle	10.66	13.89	12.27	24.55	32.12	32.78
	Skin	16.66	18.17	17.41	34.83	31.13	35.69
	Spleen	23.00	27.16	25.08	50.16	37.76	27.87

Supplementary Table 31. Co-transcription of the homoeologous pairs in nine tissues and nine conditions

Common carp	Co-transcription in nine tissues		Co-transcription in nine conditions	
	**Type**	**Pair number**	**Type**	**Pair number**
	At least one tissue	2,071	At least one condition	2,023
	At least two tissues	1,993	At least two conditions	1,972
	At least three tissues	1,916	At least three conditions	1,916
	At least four tissues	1,846	At least four conditions	1,855
	At least five tissues	1,770	At least five conditions	1,789
	At least six tissues	1,703	At least six conditions	1,722
	At least seven tissues	1,630	At least seven conditions	1,643
	At least eight tissues	1,567	At least eight conditions	1,567
	In all tissues	1,451	In all conditions	1,459
**Goldfish**	At least one tissue	2,084	At least one condition	2,094
	At least two tissues	2,045	At least two conditions	2,086
	At least three tissues	1,993	At least three conditions	2,071
	At least four tissues	1,931	At least four conditions	2,055
	At least five tissues	1,866	At least five conditions	1,959
	At least six tissues	1,794	At least six conditions	1,882
	At least seven tissues	1,725	At least seven conditions	1,707
	At least eight tissues	1,644	At least eight conditions	1,617
	In all tissues	1,521	In all conditions	1,520

Supplementary Table 32. Statistics of co-expressed, neo-F, sub-F, and non-F groups of 2,096 homoeologous pairs in nine tissues and nine conditions

Species	Type	Nine tissues			Nine conditions		
		Total	Consistent*	Inconsistent	Total	Consistent	Inconsistent
Common carp	Co-expression	1,205	872 (72.4%)	333 (27.6%)	1,211	872 (72.0%)	339 (28.0%)
	Neo-functionalization	572	199 (34.8%)	373 (65.2%)	451	199 (44.1%)	252 (55.9%)
	Sub-functionalization	273	103 (37.7%)	170 (62.3%)	375	103 (27.5%)	272 (72.5%)
	Non-functionalization	43	32 (74.4%)	11 (25.6%)	59	32 (54.2%)	27 (45.8%)
Goldfish	Co-expression	1,122	753 (67.1%)	369 (32.9%)	1,200	753 (62.7%)	447 (37.2%)
	Neo-functionalization	591	285 (48.2%)	306 (51.8%)	558	285 (51.1%)	273 (48.9%)
	Sub-functionalization	353	214 (60.6%)	139 (39.4%)	310	214 (69.0%)	96 (31.0%)
	Non-functionalization	30	23 (76.7%)	7 (23.3%)	24	23 (95.8%)	1 (4.2%)

*: if one pair was classified into the same type of group in both tissue expression analysis and condition expression analysis, it was considered to be consistent. Or else, it was inconsistent.

Supplementary Table 33. The inconsistence among the co-expressed, neo-F, sub-F, and non-F groups of 2,096 homoeologous pairs in nine tissues and nine conditions

	Inconsistent type	Pair number
Common carp	Tissue coexpression and condition neo-functionalization	185
	Tissue coexpression and condition sub-functionalization	132
	Tissue coexpression and condition non-functionalization	16
	Tissue sub-functionalization and condition coexpression	103
	Tissue neo-functionalization and condition coexpression	227
	Tissue non-functionalization and condition coexpression	8
Goldfish	Tissue coexpression and condition neo-functionalization	269
	Tissue coexpression and condition sub-functionalization	96
	Tissue coexpression and condition non-functionalization	0
	Tissue sub-functionalization and condition coexpression	137
	Tissue neo-functionalization and condition coexpression	306
	Tissue non-functionalization and condition coexpression	4

Supplementary Table 34. Differentially expressed homoeologues in different comparisons

		DEGs in subA genes	DEGs in subB genes	P value
Common carp	Anti-hypoxia#	36	34	0.8111
	Anti-CyHV3#	134	146	0.4733
	Anti-*A. hydrophila*#	90	103	0.3494
	Black against Red*	0	0	NA
	Black against White*	6	6	1
	Red against White*	4	5	0.2482
Goldfish	Anti-Bisphenol*	15	13	0.8501
	Anti-Gyrodactylus#	34	37	0.7218
	High FCE *vs* Low FCE*	2	1	1
	Control against SKF*	0	1	1
	Control against SNa*	10	5	0.3009
	SNa against SKF#	28	31	0.6961

#: Two-sided Chi-square test; *: Two-sided Fisher exact test.

Supplementary Table 35. Differentially expressed homoeologous pairs in multiple condition comparisons

		One UPG	One DPG	Both UPG	Both DPG	One UPG and the other DPG	Total pair
Common carp	Anti-hypoxia	28	6	18	0	0	52
	Anti-CyHV3	132	34	38	18	1	223
	Anti-*Aeromonas hydrophila*	44	83	5	26	2	160
	Black *vs* Red	0	0	0	0	0	0
	Black *vs* White	1	3	1	3	0	8
	Red *vs* White	0	6	1	2	0	9
Goldfish	Anti-Bisphenol	9	15	2	0	0	26
	Anti-Gyrodactylus	43	16	5	1	0	65
	High FCE *vs* Low FCE	3	0	0	0	0	3
	Control *vs* SKF	1	0	0	0	0	1
	Control *vs* SNa	0	13	0	1	0	14
	SNa *vs* SKF	6	41	1	5	0	53

DPG: down-upregulated gene; UPG: up-regulated gene

Supplementary Table 36. Genome-wide DEGs in multiple condition comparisons

		UPG in A subgenome	UPGs in B subgenome	P value	DPGs in A subgenome	DPGs in B subgenome	P value
Common carp	Anti-hypoxia	159	178	0.5531#	44	48	0.9130#
	Anti-CyHV3	693	787	0.1036#	283	295	1#
	Anti-*Aeromonas hydrophila*	281	316	0.3795#	482	571	0.0406#
	Black *vs* Red	0	1	1.0000*	11	5	0.1817*
	Black *vs* White	1	3	0.6472*	11	13	0.9202*
	Red *vs* White	8	4	0.3473*	13	22	0.2197#
Goldfish	Bisphenol treatment	72	80	1#	65	79	0.6515#
	Anti-Gyrodactylus	150	168	0.9899#	91	91	0.5223#
	High FCE *vs* Low FCE	16	18	1#	21	22	0.9673#
	Control *vs* SKF	0	2	0.5014*	2	3	1*
	Control *vs* SNa	0	2	0.5014*	35	34	0.6606#
	SNa *vs* SKF	37	46	0.6903#	116	150	0.2428#

DPG: down-regulated gene; UPG: up-regulated gene; #: Two-sided Chi-square test; *: Two-sided Fisher exact test.

Supplementary Table 37. Diversity levels of the FR strain and the YR strain

	**FR strain**		**YR strain**	
	Mean	S.D.	Mean	S.D.
π	$1.57x10^{-3}$	$8.9x10^{-4}$	$1.99\ x10^{-3}$	$1.07\ x10^{-3}$
θw	$1.18\ x10^{-3}$	$5.6\ x10^{-4}$	$1.39\ x10^{-3}$	$6.44\ x10^{-4}$
Tajima'D	1.14	0.41	1.48	0.39
FuLi'D	1.85	0.21	2.26	0.16
FuLi'F	1.88	0.29	2.32	0.26

References

1. Xu, P. *et al.* Genome sequence and genetic diversity of the common carp, *Cyprinus carpio*. *Nature Genetics* **46**, 1212-1219 (2014).
2. Kolder, I.C.R.M. *et al.* A full-body transcriptome and proteome resource for the European common carp. *BMC Genomics* **17**, 701 (2016).
3. Xu, P. *et al.* The allotetraploid origin and asymmetrical genome evolution of the common carp *Cyprinus carpio*. *Nature Communications* **10**, 4625 (2019).
4. Chen, Z. *et al.* Y. De novo assembly of the goldfish (*Carassius auratus*) genome and the evolution of genes after whole-genome duplication. *Science Advances* **5**, eaav0547 (2019).
5. Luo, J. *et al.* From asymmetrical to balanced genomic diversification during rediploidization: Subgenomic evolution in allotetraploid fish. *Science Advances* **6**, eaaz7677 (2020).
6. Chen, D. *et al.* The evolutionary origin and domestication history of goldfish (*Carassius auratus*). *Proceedings of the National Academy of Sciences* **117**, 29775 (2020).
7. Kon, T. *et al.* The Genetic Basis of Morphological Diversity in Domesticated Goldfish. *Current Biology* **30**, 2260-2274.e6 (2020).
